# Cost effective, experimentally robust differential-expression analysis for human/mammalian, pathogen and dual-species transcriptomics

**DOI:** 10.1099/mgen.0.000320

**Published:** 2019-12-18

**Authors:** Amol C. Shetty, John Mattick, Matthew Chung, Carrie McCracken, Anup Mahurkar, Scott G. Filler, Claire M. Fraser, David A. Rasko, Vincent M. Bruno, Julie C. Dunning Hotopp

**Affiliations:** ^1^​ Institute for Genome Sciences, School of Medicine, University of Maryland, Baltimore, MD 21201, USA; ^2^​ Department of Microbiology and Immunology, School of Medicine, University of Maryland, Baltimore, MD 21201, USA; ^3^​ Division of Infectious Diseases, Lundquist Institute for Biomedical Innovation at Harbor-UCLA Medical Center, Torrance, CA 90502, USA; ^4^​ David Geffen School of Medicine at UCLA, Los Angeles, CA 90502, USA; ^5^​ Department of Medicine, School of Medicine, University of Maryland, Baltimore, MD 21201, USA; ^6^​ Greenebaum Cancer Center, University of Maryland, Baltimore, MD 21201, USA

**Keywords:** sequencing, transcriptomics, RNA-Seq, dual species RNA-Seq

## Abstract

As sequencing read length has increased, researchers have quickly adopted longer reads for their experiments. Here, we examine 14 pathogen or host–pathogen differential gene expression data sets to assess whether using longer reads is warranted. A variety of data sets was used to assess what genomic attributes might affect the outcome of differential gene expression analysis including: gene density, operons, gene length, number of introns/exons and intron length. No genome attribute was found to influence the data in principal components analysis, hierarchical clustering with bootstrap support, or regression analyses of pairwise comparisons that were undertaken on the same reads, looking at all combinations of paired and unpaired reads trimmed to 36, 54, 72 and 101 bp. Read pairing had the greatest effect when there was little variation in the samples from different conditions or in their replicates (e.g. little differential gene expression). But overall, 54 and 72 bp reads were typically most similar. Given differences in costs and mapping percentages, we recommend 54 bp reads for organisms with no or few introns and 72 bp reads for all others. In a third of the data sets, read pairing had absolutely no effect, despite paired reads having twice as much data. Therefore, single-end reads seem robust for differential-expression analyses, but in eukaryotes paired-end reads are likely desired to analyse splice variants and should be preferred for data sets that are acquired with the intent to be community resources that might be used in secondary data analyses.

## Data Summary

1. The human only Cold Spring Harbor Laboratory (CSHL) ENCODE data set [1] was downloaded from ftp://hgdownload.cse.ucsc.edu/goldenPath/hg19/encodeDCC/wgEncodeCshlLongRnaSeq/.
2. The data from mice vaginas infected with *Candida albicans* [2] were downloaded from the National Center for Biotechnology Information (NCBI) SRA (sequence read archive), accession number SRP057050 (url – https://trace.ncbi.nlm.nih.gov/Traces/sra/?study=SRP057050).
3. The data from *Aspergillus fumigatus* cells in contact with human cells [3] were downloaded from the NCBI SRA, accession number PRJNA399754 (url – https://www.ncbi.nlm.nih.gov/bioproject/399754).
4. The data from a strand-specific library from a study comparing *C. albicans* cells in contact with human cells with those in media [4] were downloaded from the NCBI SRA, accession number SRP011085 (url – https://trace.ncbi.nlm.nih.gov/Traces/sra/?study=SRP011085).
5. The data from *C. albicans* in culture media [4] were downloaded from the NCBI SRA, accession number SRP011085 (url – https://trace.ncbi.nlm.nih.gov/Traces/sra/?study=SRP011085).
6. The data from *Escherichia coli* grown in different media [5] were downloaded from the NCBI SRA, accession number SRP056578 (url – https://trace.ncbi.nlm.nih.gov/Traces/sra/?study=SRP056578).
7. The data from the *Ixodes scapularis* cell line ISE6 differentially infected with *Ehrlichia chaffeensis* strains Arkansas and Heartland were downloaded from the NCBI SRA, accession number SRP040023 (url – https://trace.ncbi.nlm.nih.gov/Traces/sra/?study=SRP040023).
8. The data from the canine cell line differentially infected with *Ehrlichia chaffeensis* strains Arkansas and Heartland were downloaded from the NCBI SRA, accession number SRP040027 (url – https://trace.ncbi.nlm.nih.gov/Traces/sra/?study=SRP040027).
9. The data from a strand-specific library from *Wolbachia* endosymbiont *w*Bm from adult male and adult female *Brugia malayi* filarial nematodes [6] were downloaded from the NCBI SRA, accession number SRP068711 (url – https://trace.ncbi.nlm.nih.gov/Traces/sra/?study=SRP068711).
10. The data from a strand-specific library from *B. malayi* filarial nematodes [6] were downloaded from the NCBI SRA, accession number SRP068692 (url – https://trace.ncbi.nlm.nih.gov/Traces/sra/?study=SRP068692).
11. The data from *Helicobacter pylori* in culture with human N87 cells at 2 and 24 h where Agilent SureSelect was used to capture the transcriptome were downloaded from the NCBI SRA, accession number SRP102958 (url – https://trace.ncbi.nlm.nih.gov/Traces/sra/?study=SRP102958).
12. The data from human N87 cells infected with *H. pylori* for 2 and 24 h were downloaded from the NCBI SRA, accession number SRP102958 (url –https://trace.ncbi.nlm.nih.gov/Traces/sra/?study=SRP102958).
13. The data from a strand-specific library from *Pseudomonas aeruginosa* in stationary and exponential phases [7] were downloaded from the NCBI SRA, accession number PRJEB24688 (url – https://www.ebi.ac.uk/ena/data/view/PRJEB24688).
14. The data from a strand-specific library from *P. aeruginosa* isolates [8] were downloaded from the NCBI SRA, accession number GSE83773 (url – https://www.ncbi.nlm.nih.gov/geo/query/acc.cgi?acc=GSE83773).

**Impact Statement**

As sequencing technologies improve, sequencing costs decrease and read lengths increase. We examine host–pathogen interaction studies to assess whether using these longer reads is warranted, given their increased cost relative to using the same number of shorter reads. To this end, we compared the use of various read lengths and read pairing for 14 diverse host–pathogen data sets with varying genomic attributes including: gene density, operons, gene length, number of introns/exons, G+C content and intron length. For organisms with many introns, 72 bp reads may provide benefit, but 54 bp reads provide robust results. Likewise, for data sets that will be community resources, paired ends are likely desired to enable their use in other studies, but for differential-expression analyses, single-end reads yield robust results.

## Introduction

As sequencing throughput has increased and sequencing costs have decreased, measuring differential expression of genes using sequence data has become an increasingly powerful, effective and popular approach [9, 10]. The method has been applied successfully to two or more organisms simultaneously [11, 12], where it is often referred to as dual species or multispecies RNA sequencing (RNA-Seq)/transcriptomics. While there are several derivations, typically, a randomly sheared sequencing library is constructed from cDNA synthesized from the RNA samples of interest [13, 14]. Following the sequencing of millions of reads from these libraries, the reads are mapped to a reference and the transcript abundance is measured by counting the reads or sequencing depth underlying each transcript [14, 15]. A normalized version of this number that accounts for numerous factors, including the gene length, total number of reads sequenced and/or total number of reads mapping, is then used to compare the samples of interest and to identify genes that are differentially expressed [14, 15].

The most common platform used today for such analyses is the Illumina HiSeq, which currently generates millions of bases of sequencing data in the form of single-end or paired-end reads that are each tens to hundreds of bases in length for a few thousand pounds or less. This platform has been through frequent updates yielding longer reads and decreasing costs per bp. As read lengths have increased, many researchers have quickly used the increased read lengths, assuming it can only lead to better results. However, despite decreasing costs per bp, ultimately the longer reads often mean an increased cost per read and typically fewer reads are sequenced for the same cost. While this leads to the same sequencing depth, it results in fewer independent measurements at each position. For example, a shift from 50 bp paired-end reads to 150 bp paired-end reads leads to a 66 % reduction in the number of reads sequenced to obtain the same sequencing depth. The decreased number of reads can actually result in reduced statistical power, since a single read will contribute to the sequencing depth at a larger number of positions.

One alternate approach is to sequence the same number of overall base pairs, but use shorter paired reads. Such an approach would yield more sequence reads underlying each transcript and, therefore, more independent measurements at each position. For example, the use of 50 bp paired-end reads as opposed to 100 bp paired-end reads would lead to a 200 % increase in the number of reads sequenced to obtain the same sequencing depth. Alternatively, one could sequence the same number of reads at a short depth to save money that could be used to interrogate other conditions, sequence more replicates or perform validation experiments. As of November 2019, one academic sequencing lab (http://genomics.umn.edu/nextgen-novaseq.php) advertised sequencing 50 bp paired-end reads on the Illumina NovaSeq SP flow cell for $6.07 (£4.93; £1=$1.23) per million reads, while 150 bp paired-end reads were $9.23 (£7.50) per million reads. As such, significant savings (34 % savings) can be obtained by generating 50 bp paired-end reads instead of 150 bp paired-end reads. Another alternative would be to sequence single reads, as opposed to paired reads. However, both read length and read pairing are expected to influence the accuracy of read mapping, which is the crucial first step in any RNA-Seq analysis pipeline. Furthermore, these factors may influence the analysis of various genomes differently. For example, paired reads may be more beneficial in a genome with a large number of paralogous genes, gene families and/or repeats. This was examined through an analysis of various read lengths and pairing status (paired versus unpaired) for a human transcriptome data set that concluded that 50 bp single-end reads could be used reliably for differential-expression analysis, but that splice detection required longer, paired reads [1].

However, what works best in human data sets may not always be best for other organisms. Therefore, and given the caveats described above, we sought to investigate the influence of read length and read pairing on differential-expression analysis across a variety of genomes of various complexity including: (a) genome size, (b) presence/absence of introns, (c) length of introns, (d) number of introns per gene, (e) number of genes and (f) percentage of genes transcribed (Table S1, available with the online version of this article). In several instances, we have increased the complexity to include sequencing data that contain both an invertebrate or vertebrate animal host and an associated bacterial pathogen or endosymbiont. Ultimately, the goal was to identify the most appropriate and most cost-effective sequencing strategy based on the intrinsic properties of the genome(s) being analysed. In this way, the available resources can be appropriately distributed in order to maximize the number of biological replicates for the conditions being examined, while maintaining the greatest quality results. We find little to no effect of reducing read length or pairing status on differential-expression analyses, such that shorter read lengths and single-end reads may be the most cost-effective means to generate differential-expression data.

## Methods

### Reference genomes

The human, mouse, canine, *Ixodes* and *Aspergillus* reference genomes and annotations were downloaded from Ensembl; the *Ehrlichia chaffeensis *, *Escherichia coli *, *Helicobacter pylori *, *Pseudomonas aeruginosa* and *w*Bm reference files were downloaded from the National Center for Biotechnology Information (NCBI); the *Candida albicans* reference files were downloaded from the *Candida* Genome Database (http://www.candidagenome.org); and the *Brugia malayi* reference files were downloaded from WormBase (https://www.wormbase.org) (Table S1). The fasta genomic sequences were indexed using SAMtools (v. 0.1.19) [16]. The GTF/GFF reference annotations were used to extract genomic coordinates for the genes, exons and introns using BEDTools (v. 2.17.0) [17].

### Sequencing data used

The 101 or 151 bp paired-end sequencing reads for each sample (Table S2) were trimmed from the 3′ end of the sequence read to generate 36, 54, 72 and 101 bp reads using the FASTX-Toolkit (http://hannonlab.cshl.edu/fastx_toolkit) generating two separate fastq files consisting of first-in-pair reads and second-in-pair reads that were compressed for downstream analysis.

### Reference-based alignment

The sequencing reads were aligned to their respective reference genome fasta sequence using the TopHat splice-aware aligner (v. 2.012) [18] for eukaryotic data or Bowtie aligner (v. 0.12.9) [19] for prokaryotic data allowing for a maximum of two mismatches per aligned read, an inner mate distance of 200 bp and discarding reads that aligned to more than 20 genomic loci. For the subset also examined with bwa, bwa mem v0.7.17 [20] was run using all default settings and a seed length of 23 bp. The alignment files were sorted, indexed and converted between BAM and SAM formats using SAMtools (v. 0.1.19) [16]. The alignment files were used to compute the total number of reads per sample, the number of reads that aligned to the reference genome, the number of reads that mapped once to the genome and the number of reads that mapped to >1 but <20 genomic loci (Table S2). The percentage of fragments for paired end reads and reads for single end reads that mapped to exons, introns, genes and intergenic regions of the genome were computed based on coordinates from the respective annotation files in GTF/GFF format.

### Read counts per kb of the gene length per million mapped reads (RPKM) calculations

The number of reads/fragments that mapped to each gene was calculated from the BAM alignments using HTSeq (v. 0.5.4) [21] and further normalized for sequencing library depth and gene length to estimate the RPKM or fragments per kbp of the gene length per million mapped reads (FPKM) for each gene for each set of fastq files.

### Hierarchical clustering and principal components analysis (PCA)

The raw counts from HTSeq were further normalized using DESeq (v. 1.10.1) [22] in R (v. 2.15.2) [23]. Genes with low read counts across all samples for a data set were excluded from downstream analysis. The final set of normalized gene expression values for each gene for each sample within a data set was used to compute a Euclidean distance matrix between every pair of samples that was used to generate a heat map cluster with pvclust with 1000 bootstraps. Eigen vectors were calculated with the PCA package in R to determine the first and second principal components (PCs) that illustrate the vectors with the largest variance in the data set.

The final set of normalized gene expression values for each gene for each sample within a data set was used to test for differential gene expression between the two conditions using the ‘negative binomial’ test incorporated within DESeq (v. 1.10.1) [22] in R (v. 2.15.2) [23]). The final results were then filtered to determine significant differentially expressed genes using a <5 % false discovery rate (FDR), greater than twofold change, and a >10th percentile of mean normalized gene expression distribution within the data set.

## Results

### Design and data set selection

We examined RNA-Seq data from 14 studies to test the effect of read length and read pairing on gene expression data from a wide set of pathogen or host–pathogen samples (Table S1). This included eukaryotic and prokaryotic genomes; organisms of varying genome size and varying numbers of genes; organisms with and without introns; organisms of varying intron length; organisms with varying numbers of exons per gene; bacteria of varying G+C content; and data from single organisms compared to those from mixtures of organisms with an emphasis on host–pathogen systems (Table S1). All of the data sets used were generated as 101 or 151 bp paired-end reads. Data were trimmed from the 3′-end of the read to generate 36, 54, 72 and 101 bp data sets. The first read in the pair was analysed separately from the second read in the pair when single-end reads were analysed.

To examine the influence of read length and pairing at many steps, analyses were undertaken on multiple data sets. Mapping statistics were calculated from the Bowtie alignments. PCA and hierarchical clustering were undertaken on the DESeq normalized read counts for each individual replicate in each biological condition (Additional Files S1–S28). Scatterplots were used to examine differential-expression results obtained with DESeq (Additional Files S29–S30).

### Read mapping as a function of read length

**Fig. 1.**
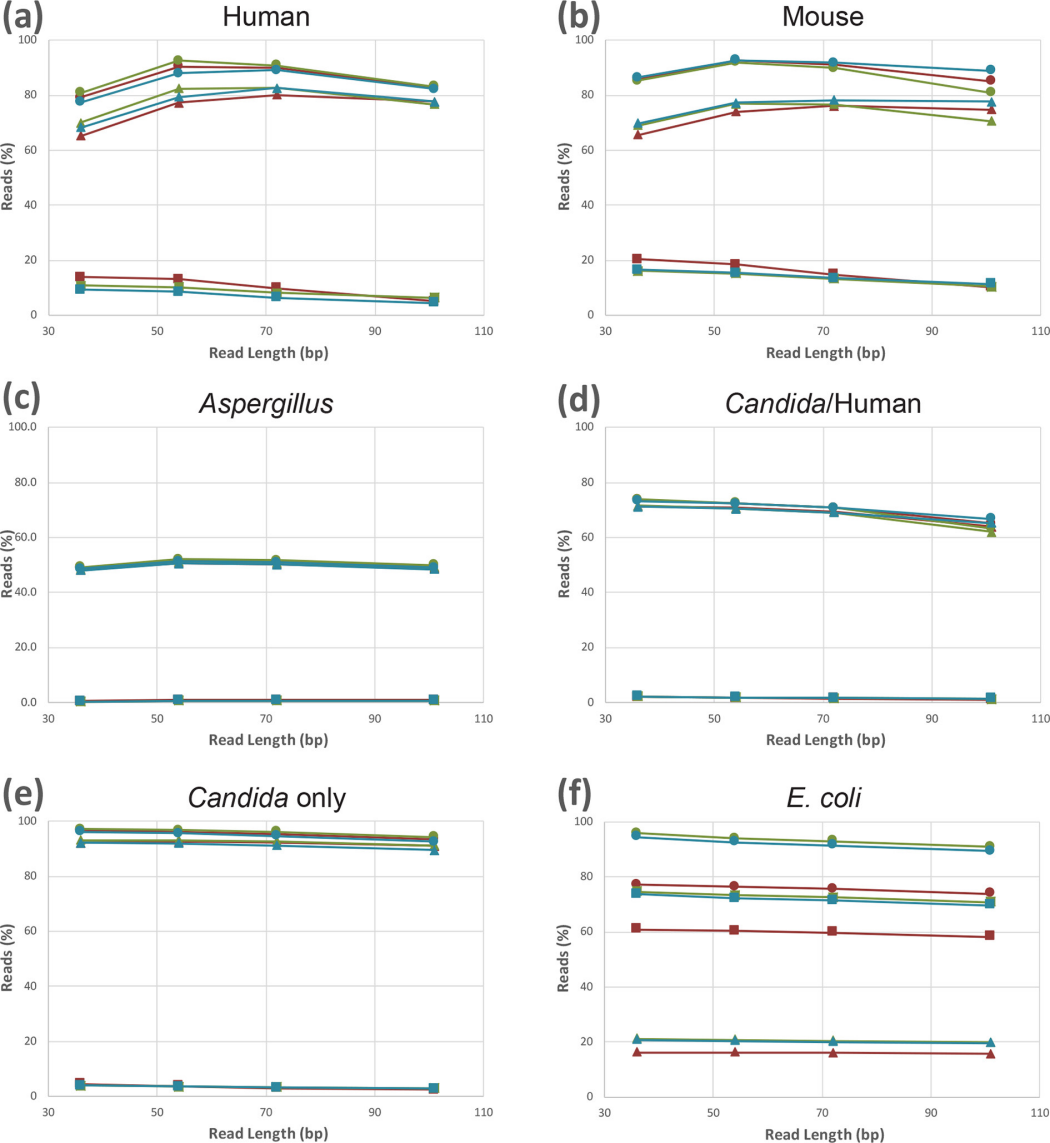
The percentage of reads mapping (circles), reads mapping uniquely (triangles) and reads not mapping uniquely (squares) are compared for 36, 54, 72 and 101 bp reads for the human (a), mouse (b), *Aspergillus* (c), *Candida*/host (d), *Candida* only (e) and *E. coli* (f) data sets. Results are compared for mappings with the paired reads (red), only the first read in the pair (green) and only the second read in the pair (blue).

The number of reads mapping is dependent upon the number of mismatches allowed, as well as the uniqueness of the sequence, both of which are expected to vary by read length as well as the aligner used. We expect that fewer 36 bp reads will map uniquely since a greater proportion will multi-map, and we expect that fewer 101 bp reads will map because of the accumulation of sequencing errors, which increases with read length. As expected in six representative data sets, in half of the cases fewer reads map uniquely for 36 bp and 101 bp for both paired- and single-end reads, relative to the 54 and 72 bp equivalents (Fig. 1a, b, c). The number of multi-map reads that do not uniquely map decreases as a function of read length (Fig. 1a, b, squares). However, in organisms with smaller genomes that have no introns (i.e. *E. coli *) or a limited number of introns (i.e. *C. albicans*), increasing read length leads to decreasing mapped read counts (Fig. 1d, e, f).

In this subset of six data sets, the greatest proportion of multi-mapping reads were found in *E. coli *﻿, followed by mouse and human. Unlike the eukaryotic data sets analysed where polyadenylated RNA can be enriched and sequenced, the *E. coli* data, despite having rRNA depleted with a commercial kit, retained a sizable proportion of rRNA that was sequenced. Given that there are seven copies of the rRNA in the reference genome used for mapping [24], a large number of multi-mapping reads were expected. Therefore, as expected, >99 % of reads mapping to the rRNA genes were multi-mapping reads and, on average, 78 % of the mapped reads mapped to the rRNA genes. The increase in multi-mapping reads in human and mouse is expected given their genome size and composition. In both humans and mice, the paired-end reads yielded slightly more multiple hits than the single-end reads, which we attribute to how the aligner handles multi-mapping reads.

### PCA of read length

**Fig. 2.**
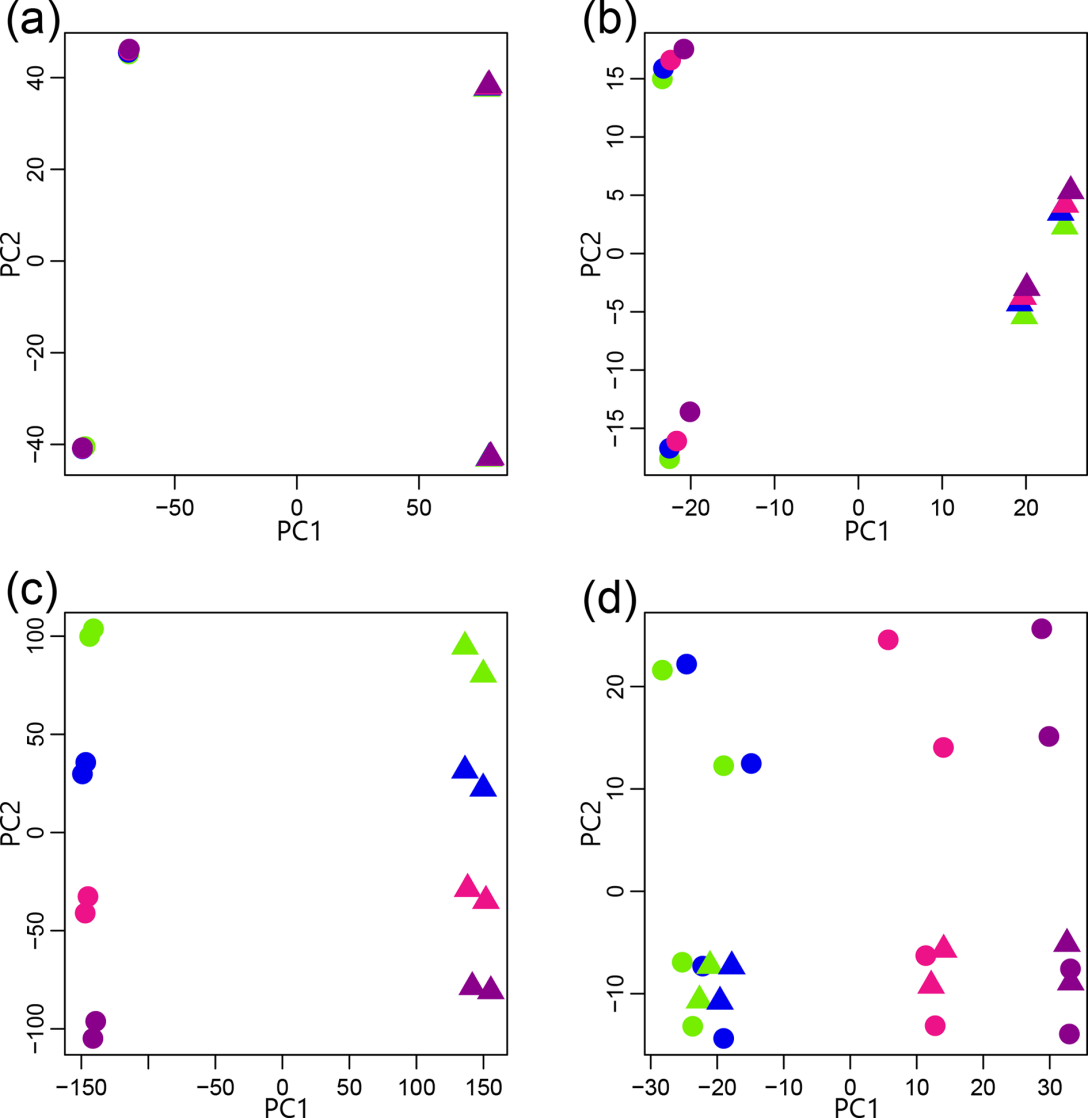
A PCA was undertaken for a vector representing data for the different read lengths (green, 36 bp; blue, 54 bp; magenta, 72 bp; purple, 101 bp), replicates and biological conditions. Four representative results are illustrated with *E. coli* paired-end data (circle, DMEM; triangle, LB) (a), *Candida/*human first-in-pair single-end reads (circle, 5h_c; triangle, 5h_oc) (b), CSHL ENCODE human first-in-pair single-end reads (circle, IMR-90; triangle, NHD) (c) and *Wolbachia* paired-end reads (circle, adult females; triangle, adult males) (d). All PCA plots for read length are provided in Additional Files S1–S12 and pairing statuses are provided in Additional Files S13–S24.

If read length is of no consequence, we would expect the first PC to separate the data based on biological condition and the second PC to separate the data based on replicates. Furthermore, we would expect all of the read lengths derived from the same data to be tightly grouped. This was observed for *E. coli* and both of the *P. aeruginosa* data sets (Fig. 2a **,** Additional Files S6, S13 and S14), where the read length seems to be inconsequential.

However, the data from the 11 other comparisons resulted in different patterns with read length having a greater role. The reads from the *Candida*/human data set demonstrate similar PC1 and PC2, but the spread of the data points suggests that read length may have some influence on the data (Fig. 2b **,** Additional File S4). This was also observed for the paired-end reads from the human/Cold Spring Harbor Laboratory (CSHL) data set and all of the *Aspergillus*, *Candida*, tick, canine and human with *Helicobacter* data regardless of pairing status (Additional Files S1, S3, S4, S5, S7, S8 and S12). In this case, the biological samples still cluster best with each other, as do their replicates.

The influence of read length is more pronounced in the single-end reads from the CSHL data set, which were separated on the first PC by biological replicate but were separated by read length on the second PC (Fig. 2c **,** Additional File S1). This suggests that there were greater distinctions in data with varying read lengths than there were in the replicates. This was also true for both the paired-end reads and single-end reads from the mouse with *Candida*, the *Helicobacter* with human and the *B. malayi* data (Additional Files S2, S10 and S11). In this case, the read length exerts a bigger effect, but the biological replicates still cluster best with each other. However, the sample replicates have less variation than is introduced by varying the read length.

The influence of read length is most pronounced in the *Wolbachia* data, which were separated on the first PC by read length and by samples in the second PC (Fig. 2d **,** Additional File S9). The samples from the two biological conditions and their replicates were intermixed in the second PC. This suggests that there are greater distinctions in the data of varying read lengths than there were in the biological samples or their replicates. In other words, the samples and their replicates have less variation than is introduced by varying the read length. In all cases, when read length does divide the data, it is always distinguished from decreasing to increasing read length along the axis, as opposed to a random order.

### Hierarchical clustering as a function of read length

**Fig. 3.**
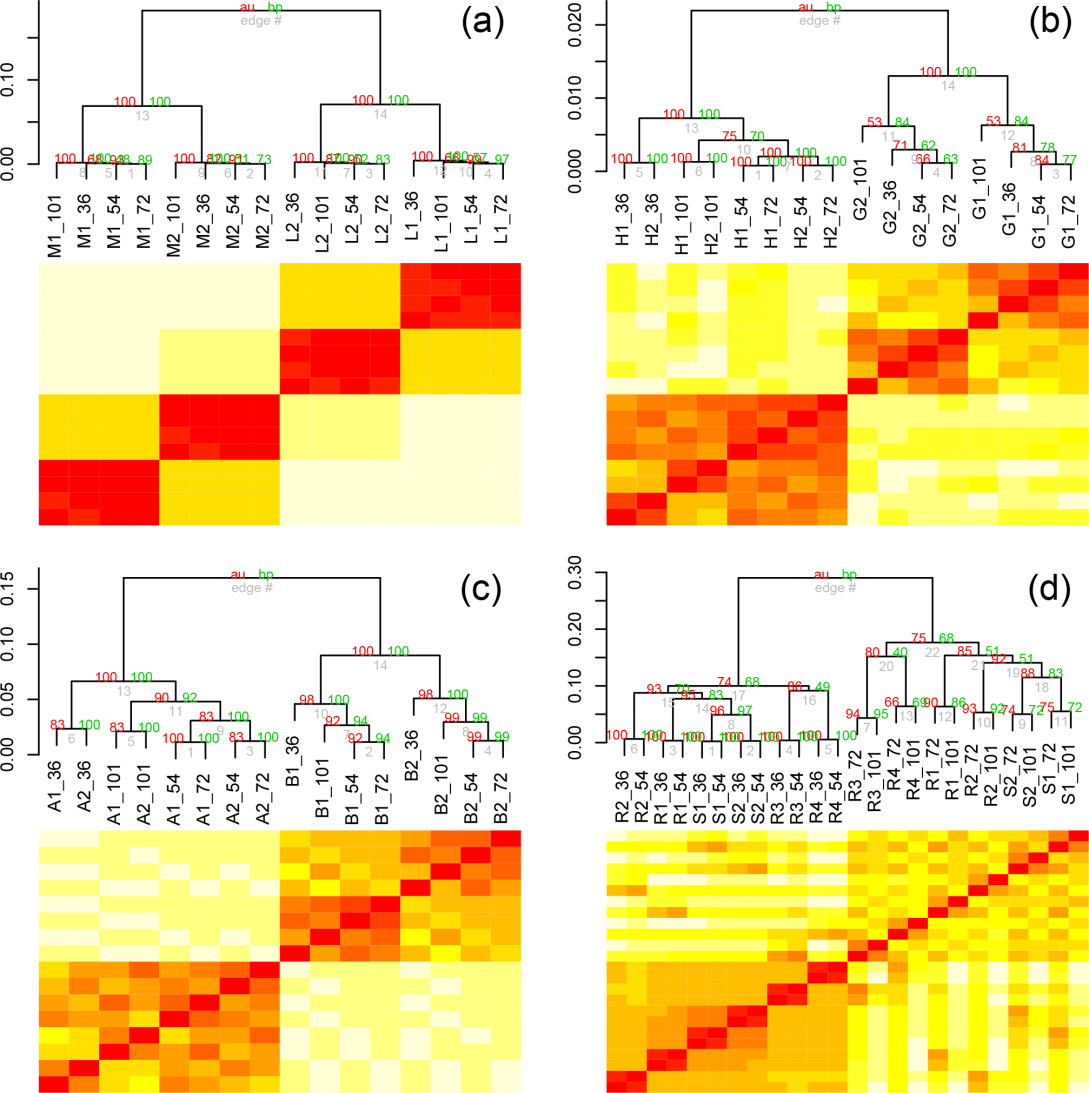
Hierarchical clustering using pvclust for bootstrap support was undertaken for a vector representing data for each sample at different read lengths. Samples are labelled according to the key in Table S2, followed by the read length (36, 54, 72 and 101 bp). Four representative results are illustrated here with *E. coli* paired-end data (a), *Candida/*human first-in-pair single-end reads (b), ENCODE human first-in-pair single-end reads (c) and *Wolbachia* paired-end reads (d). In the *E. coli* data, read length did not affect the clustering of the data, while the largest effect of read length was observed with the *Wolbachia* data. All hierarchical clustering plots for read length are provided in Additional Files S1–12 and pairing status are provided in Additional Files S13–24.

In numerous cases, hierarchical clustering (complete clustering, correlation distance) of the data sets with statistical support (AU, approximately unbiased, and BP, bootstrap probability) is consistent with the PCA. In the *E. coli* data, when the PCA reveals data clustering by biological condition and then replication, but not by read length, the heat map and dendrogram show similar, well-supported (confidence ≥80 %) clustering (Fig. 3a, Additional File S6). In the instances where the PCA revealed that read length had some influence, the hierarchical clustering mirrored that (Fig. 3b, c **,** Additional Files S1–5, S7–9, S10–12). Read length had the greatest influence with the *Wolbachia* data, where data clustered primarily by read length (Fig. 3d, Additional Files S2 and S9).

### Log-fold change of differentially expressed genes as a function of read length

**Fig. 4.**
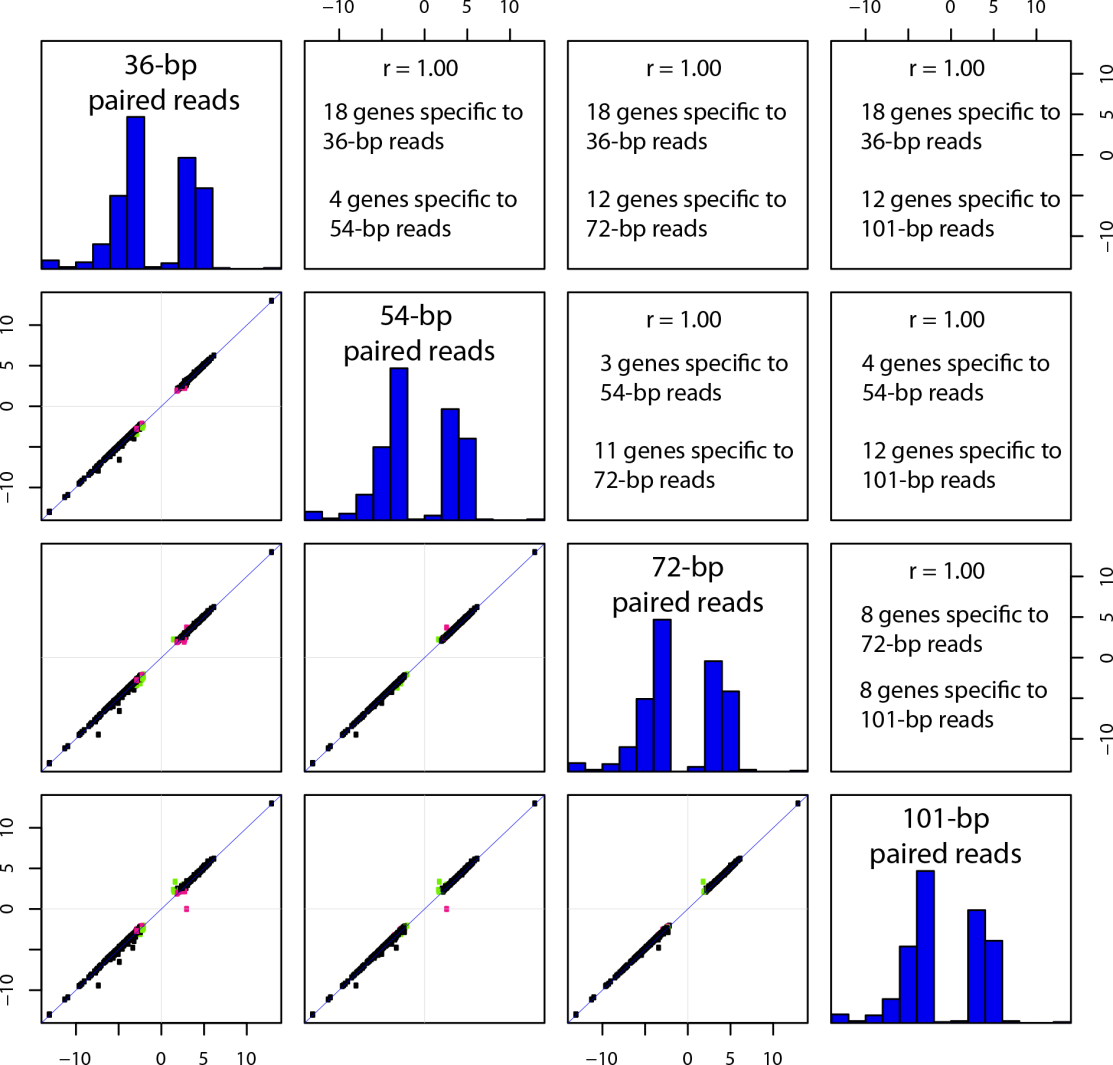
The differentially expressed genes identified in *E. coli* (L vs M) using an adjusted *P* value (FDR) cut-off ≤0.05 for paired-end reads at varying read lengths within a data set were compared using Pearson’s correlation implemented in the R statistical tool and illustrated as a matrix of scatterplots. The diagonal represents the histogram of log-transformed fold-changes within the comparison. The lower plots represent the correlation between comparisons with singleton differentially expressed genes identified for comparisons on the *x*-axis (pink) and *y*-axis (green). Genes with FDR >0.05 in both comparisons are not shown. The upper portion of the plot lists the corresponding Pearson’s correlation coefficient and the number of singleton differentially expressed genes identified in each comparison.

For all comparisons, the log-fold change of differentially expressed genes between the two conditions correlates well across all pairwise comparisons of read length for single-end and paired-end reads, with R^2^ values ranging from 0.63 to 1.0 (mean, 0.94; median, 0.97) (Table S3, Additional File S29). Remarkably, all such pairwise comparisons with *E. coli* yield R^2^ values of 0.99 or 1.00 (Fig. 4 **,** Additional File S29), as did those for *H. pylori* and one of the *P. aeruginosa* data sets, suggesting that 36 bp reads yield the same results as 101 bp reads. Overall though, comparisons that include 36 bp reads have lower correlation values relative to those from the longer read length data (Table S3). The highest correlations are found in comparisons of the closest read lengths (e.g. 54 bp vs 72 bp and 72 bp vs 101 bp) (Table S3).

A slightly different result is observed when focusing on genes found to be differentially regulated at one read length, but not found to be differentially regulated at another read length, referred to as singletons. In this case, the 54 bp versus 72 bp comparison typically outperformed all other comparisons (Table S4, Additional File S29). The next best comparisons were the other two groupings of similar sizes, 36 bp versus 54 bp and 72 bp versus 101 bp (Table S4, Additional File S29).

### PCA of read pairing

**Fig. 5.**
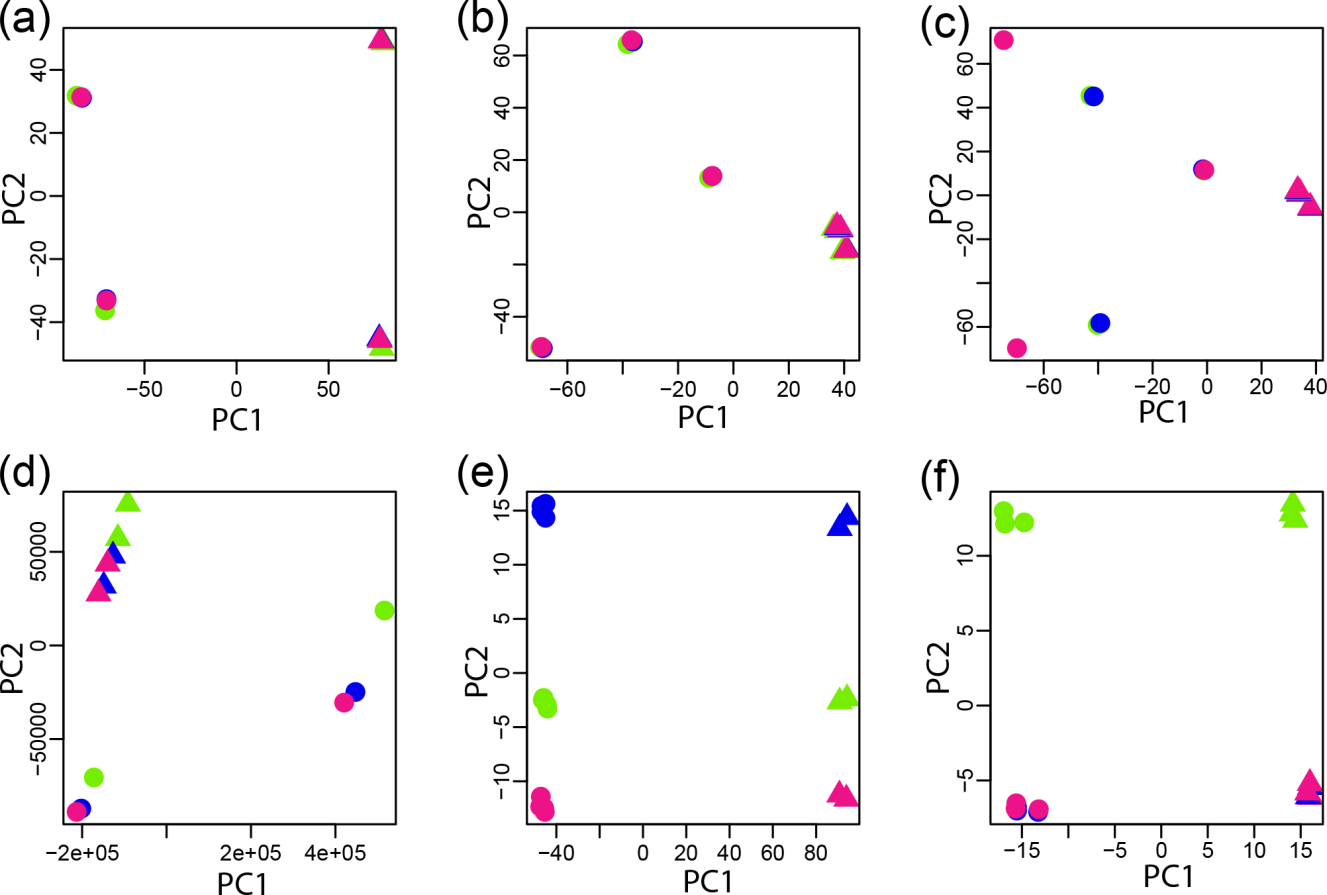
A PCA was undertaken for a vector representing data for the different pairing statuses (paired end, green; first-in-pair single end, blue; second-in-pair single end, pink) for the biological samples and their replicates. Six representative results are illustrated with 72 bp *E. coli* data for minimal media (circles) and rich media (triangles) (a), 72 bp data for ticks differentially infected with *E. chaffeensis* strain Arkansas (circle) and strain Heartland (triangle) (b), 101 bp data for ticks differentially infected with *E. chaffeensis* strain Arkansas (circle) and strain Heartland (triangle) (c), 101 bp *Candida* data from rhr2_comp (circle) and rh2_exp (triangle) (d), 72 bp *B. malayi* data from adult females (circle) and adult males (triangle) (e) and 72 bp *H *. *pylori* data from a 24 hour time point (circle) and a 2 hour time point (triangle) (f). All PCA plots for read length are provided in Additional Files S1–12 and pairing status are provided in Additional Files S13–24.

Read pairing is expected to exert influences in many of the same ways as read length. If read pairing is of no consequence, data from paired- and single-end reads should be more similar to one another than to samples from other biological conditions or replicates in a PCA plot. As such, we expect the first PC to resolve biological samples while the second resolves replicates. This was observed for data from the *E. coli* data sets where read pairing had no effect for each of the four read lengths examined (Fig. 5a **,** Additional File S20). In the other data sets, five other patterns were observed where in all cases at least paired-end reads were different than single-end reads.

In all but the 101 bp reads for tick cells infected with *Ehrlichia *, biological samples separate on PC1, but PC2 does not separate the samples on replicate (Fig. 5b **,** Additional File S21). The same is seen for canine cells infected with *Ehrlichia chaffeensis* and human cells infected with *H. pylori* (Additional Files S22 and S26). However, the 101 bp tick data show a difference between paired and unpaired data for only two of the six samples, while all other read lengths and samples show no difference (Fig. 5c **,** Additional File S21).

In the human ENCODE data, the *Wolbachia* endosymbiont of *B. malayi* data, and several of the other data sets, a similar pattern is observed except that the differentially paired data are more loosely grouped (Additional Files S15, S16, S17, S18, S19 and S23). In this case, while the replicates do not define the clustering, the pairing status also does not. Frequently, but not always, this dispersion of the data results in the two single-end data sets grouping together and separate from the paired-end data as is observed for the 101 bp *Candida* only data (Fig. 5d). Typically, but not always, the effect was greatest in the shortest reads (i.e. a larger dispersion between paired- and single-end 32 bp reads than for paired- and single-end 101 bp reads).

In the *B. malayi* data and both *P. aeruginosa* data sets, PC1 separates the biological replicates, but PC2 separates the paired and unpaired data with the single-end data being different from each other (Fig. 5e **,** Additional Files S24, S27 and S28). These data are from strand-specific (or directional) libraries and this could explain why the unpaired data sets are different from one another. Examining the other strand-specific libraries also showed dissimilarity in the unpaired reads (Additional Files S18 and S23) that was not observed in other samples.

In the *H. pylori* data, PC1 separates the biological replicates, but PC2 separates the paired and unpaired data with the single-end data clustering together (Fig. 5f **,** Additional File S25). This is the only sample from an Agilent SureSelect capture library, and it is possible that this leads to a strong difference in pairing status.

### Hierarchical clustering as a function of read pairing

Hierarchical clustering of the data sets largely supports the PCA for read pairing when there are strong AU/BP values. For *E. coli *, hierarchical clustering separates first by biological condition and then replication, but not by read pairing, which is well supported by the AU/BP values (100%) (Additional File S20). The instances where the PCA showed the greatest influence of read pairing also showed the greatest variation in hierarchical clustering, including the 101 bp tick reads (Additional File S21) where two data sets of paired-end reads are quite different than their unpaired data counterparts. The *B. malayi* hierarchical clustering and similarity matrix illustrates that the data between the replicates are so similar that it may not be surprising that the PCA and hierarchical clustering separates the samples by pairing status (Additional File S24).

### Log-fold change of differentially expressed genes as a function of pairing status

For all comparisons, the log-fold change of differentially expressed genes between the two conditions correlates well across all pairwise comparisons of pairing status regardless of the length of the reads, with R^2^ values ranging from 0.49 to 1.0 (mean, 0.92; median, 0.96) (Table S5, Additional File S30). Even when the PCA identified a difference in the single-end reads, for example in the *P. aeruginosa* comparisons, there is a strong correlation between the genes called differentially expressed and their values. Overall though, comparisons of the single-end reads with each other have lower correlation values relative to those from the longer read length data (Table S5, Additional File S30).

### Effect of aligner

The effect of the read aligner was tested using the *E. coli* and *Wolbachia* data sets by comparing the results above that were obtained with Bowtie with those from bwa mem (Additional Files S31–S36). These two data sets were selected since they represented the extremes of the results presented above with *E. coli* data not being affected upon changing read length or pairing status, whereas both read length and pairing influence the results with the *Wolbachia* data. There was virtually no difference between the two aligners for the *E. coli* data set. For the *Wolbachia* data set, there was still an effect of read length, but it was smaller with bwa mem than with Bowtie. As such, bwa mem may handle varying read lengths and pairing statuses better than Bowtie.

### No discernible effect from the sequencing centre

While most of the data was generated by Maryland Genomics within the Institute for Genome Sciences at the University of Maryland School of Medicine (USA), publicly available data sets were included from three other groups. No differences were found that could be attributed to the sequencing centre. Two of the external data sets were selected to be similar data sets, both being strand-specific data generated from RNA from monocultures of the same species. There was no discernible difference between the two.

## Discussion

### Effect of differential expression levels

**Fig. 6.**
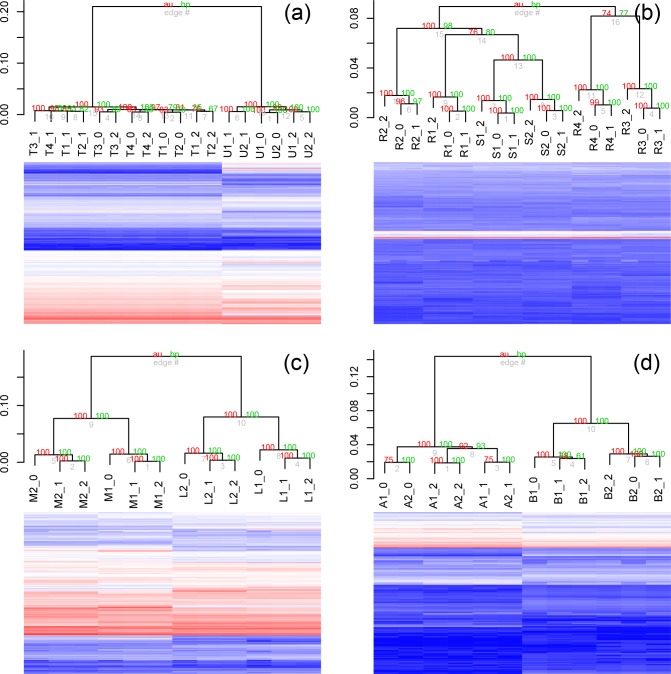
Hierarchical clustering using pvclust for bootstrap support was undertaken for a vector representing data for each sample at different read lengths with heatmaps illustrating the DESeq normalized read counts of the samples. Samples are labelled according to the key in Table S2, followed by the read length (36, 54, 72 and 101 bp). Four representative results are illustrated here with *w*Bm paired-end data (a), *B. malayi* paired-end data (b), ENCODE human paired-end data (c) and *E. coli* paired-end data (d). Little variation is seen in the biological samples and their replicates from *Wolbachia,* as opposed to *E. coli,* which likely explains why the read length has a strong effect in the *w*Bm data relative to the *E. coli* data.

The data for the *Wolbachia* endosymbiont of *w*Bm were the only data to cluster by read length, instead of biological sample. These data were from *Wolbachia* from whole adult male and female *B. malayi*, which are nematodes/roundworms. Few differences in differential expression were observed between *Wolbachia* endosymbionts from male soma and female germline cells of a related nematode, *Onchocerca ochengi* [25]. In the *w*Bm study, our analysis visually shows little variation between samples (Fig. 6a), and suggests that the variation between the biological samples and their replicates is less than the variation introduced by truncating the reads. In other data sets analysed here, when samples clustered by read length in the replicates, the samples also have little variation in the replicates. For example, the *Brugia* paired-end data and the ENCODE paired-end data both showed clustering on read length in the replicates, but resolved the biological samples. In both cases, there were significant differences in the biological samples, but very little variation between the replicates (Fig. 6b, c). In contrast, the *E. coli* data, which showed little influence of read length in the replicates, had larger variation between the biological conditions and their biological replicates (Fig. 6d). This result suggests that read length has the greatest influence between samples that are highly similar. This suggests that computational truncation of reads could be used as a low cost post hoc method to measure the variation in samples.

We compared data sets that represented a diverse array of genomic complexity to assess what attributes might affect the outcome. Our selection included genomes with high gene density, genomes with operons, genomes with long genes, genomes with many introns/exons, genomes with differing G+C content and genomes with long introns. We did not observe any obvious patterns associated with these criteria.

### Best read length

Overall, in all but *w*Bm, as discussed above, samples clustered by biological replicate rather than read length, suggesting that data from a variety of read lengths can yield robust data. However, data changed in most cases based on read lengths and pairing status, such that different data lengths and types should not be combined whenever possible (e.g. in secondary data analysis and meta-analyses).

For *E. coli *, all read lengths resolved the biological samples and their replicates correctly, suggesting that 36 bp reads could be a viable option. However, 36 bp reads performed differently than the other length reads in most other comparisons. Additionally, the number of multi-map reads that do not map uniquely decreases as a function of read length. This suggests that the use of 36 bp reads should probably be minimized despite the decreased cost. As read length increased, fewer reads map, likely owing to the known accumulation of errors in long reads, suggesting that 101 bp reads are not ideal in many cases. In all cases, 54 and 72 bp reads performed best in terms of mapping percentage. It is possible that algorithms can better accommodate sequencing errors through parameterization or further development making 101 bp reads more desirable in the future. But in these studies, with this analysis pipeline, 54 bp and 72 bp reads seem to be most desirable. They are also typically most congruent with the best correlation between them. When including the cost difference, 54 bp is likely best overall, particularly for genomes with no introns or few introns, but 72 bp reads may be desirable for genomes with many more introns; this differs slightly from the previously published recommendation of using 50 bp reads [1].

### Best pairing status

Overall, all samples clustered by biological replicate rather than pairing status, suggesting that paired and unpaired data can both yield robust data. Even for the strand-specific libraries, when there was a difference between the single-end reads, the differential-expression analysis for data sets of differing pairing status were well correlated. Unpaired reads have half as many bases of paired reads, yet in many of the data sets, the results from paired and unpaired read data sets gave nearly identical results. This demonstrates that the number of bases sequenced with a pair is essentially inconsequential. This is expected, since a single read and a pair of reads count the same in a fragment count based analysis. This suggests that there is little advantage in these cases in sequencing the second read. However, in other cases, the read pairing did result in differences. In directly comparing the differential expression, results of paired-end reads consistently yielded better correlations.

To our surprise, read pairing did not substantially increase the mapping percentage, which is the major argument for using read pairs, since the second read should help resolve the placement of reads in repetitive regions. Importantly, this study did not focus on an analysis of splice variants, but a prior study showed paired-end reads were advantageous for studying splice variants [1]. Overall, we recommend that data sets generated as community resources that might be repurposed and reused include paired-end reads, but when resources are limiting, single ends appear to yield robust results similar to paired ends for differential-expression analysis.

## Data bibliography

1. ENCODE, ftp://hgdownload.cse.ucsc.edu/goldenPath/hg19/encodeDCC/wgEncodeCshlLongRnaSeq/ (2015).

2. Bruno *et al.*, NCBI SRA (sequence read archive), accession number SRP057050 (2015).

3. Bruno *et al.*, NCBI SRA, accession number PRJNA399754 (2015).

4. Liu *et al.*, NCBI SRA, accession number SRP011085 (2015).

5. Hazen *et al.*, NCBI SRA, accession number SRP056578 (2015).

6. Lin *et al.*, NCBI SRA, accession number SRP040023 (2018).

7. Line *et al.*, NCBI SRA, accession number SRP040027 (2018).

8. Chung *et al.*, NCBI SRA, accession number SRP068711 (2018).

9. Chung *et al.*, NCBI SRA, accession number SRP068692 (2018).

10. Robinson *et al.*, NCBI SRA, accession number SRP102958 (2018).

11. Rossi *et al.*, NCBI SRA, accession number PRJEB24688 (2019).

12. Gifford *et al.*, Gene Expression Omnibus, accession number GSE83773 (2019).

## Supplementary Data

Supplementary material 1

Supplementary material 2
