# Supplementary material for: Cost effective, experimentally robust differential-expression analysis for human/mammalian, pathogen and dual-species transcriptomics
Source: Microb Genom. 2019 Dec 18;6(1):e000320. doi: 10.1099/mgen.0.000320 (PMC7067034; doi:10.1099/mgen.0.000320)
Supplement: Supplementary material 1 [file mgen-6-320-s001.pdf]

Additional File 1. Compendium of figures for Encode CSHL comparisons of IMR-90 v. NHD cells with results separated by read pairing status. A heatmap with hierarchical clustering with statistical support is shown on page 1 with the condition denoted according to letter code from Supplementary Table 2, followed by the replicate designation and the read length. A PCA plot is shown on page 2 where the conditions are denoted by the shape (circle, IMR-90; triangle, NHD) and the read length by the color (green, 36 bp; blue, 54 bp; magenta, 72 bp; purple, 101 bp). On both pages, results are shown in the three panels for (A) paired end reads, (B) first-in-pair single end reads, and (C) second-in-pair single reads.

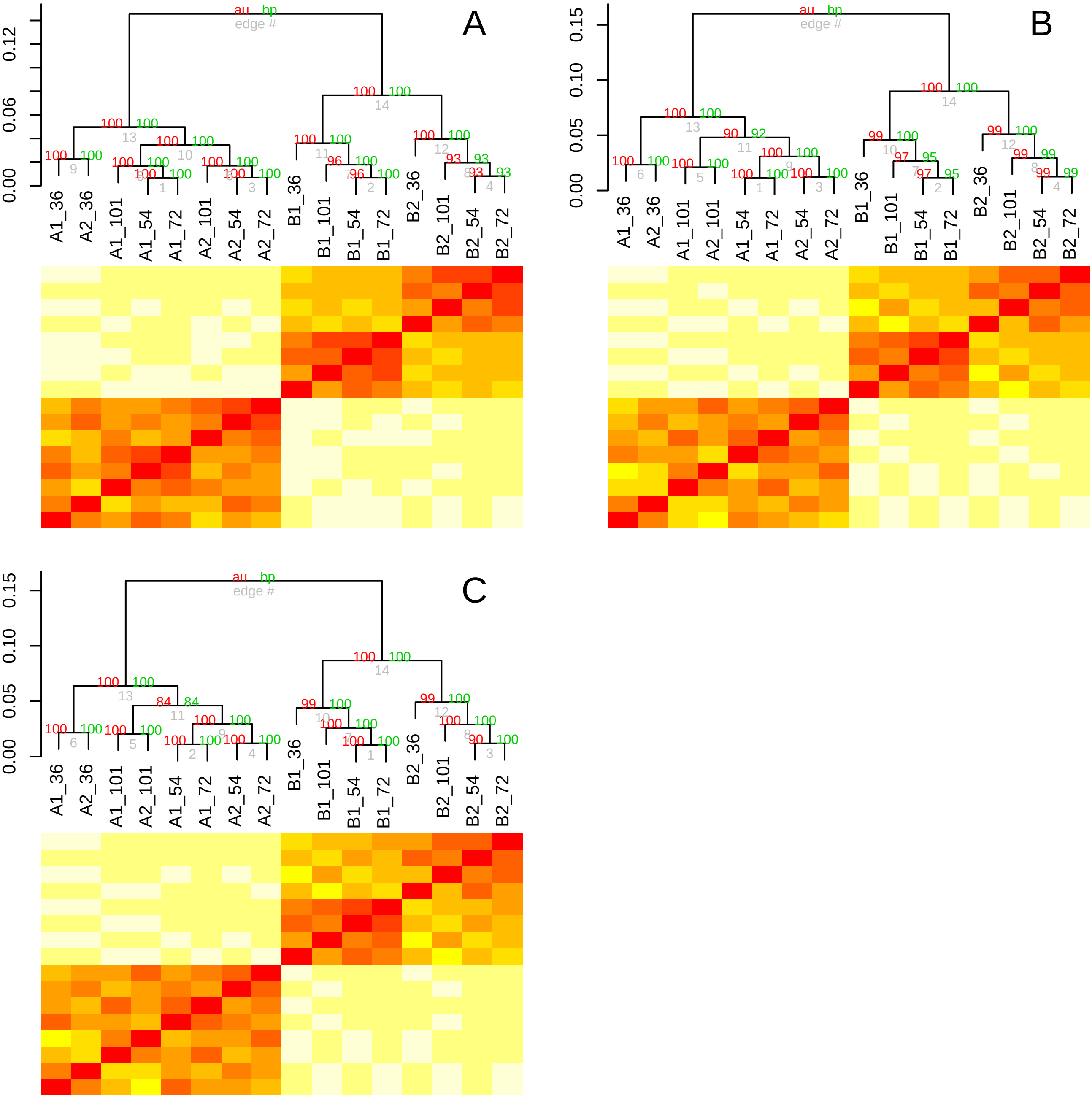
A
B
C
au
bp
edge #
0.00
0.06
0.12
0.05
0.10
0.15
A1_36
A2_36
A1_101
A2_101
A1_54
A1_72
A2_54
A2_72
B1_36
B1_101
B1_54
B1_72
B2_36
B2_101
B2_54
B2_72

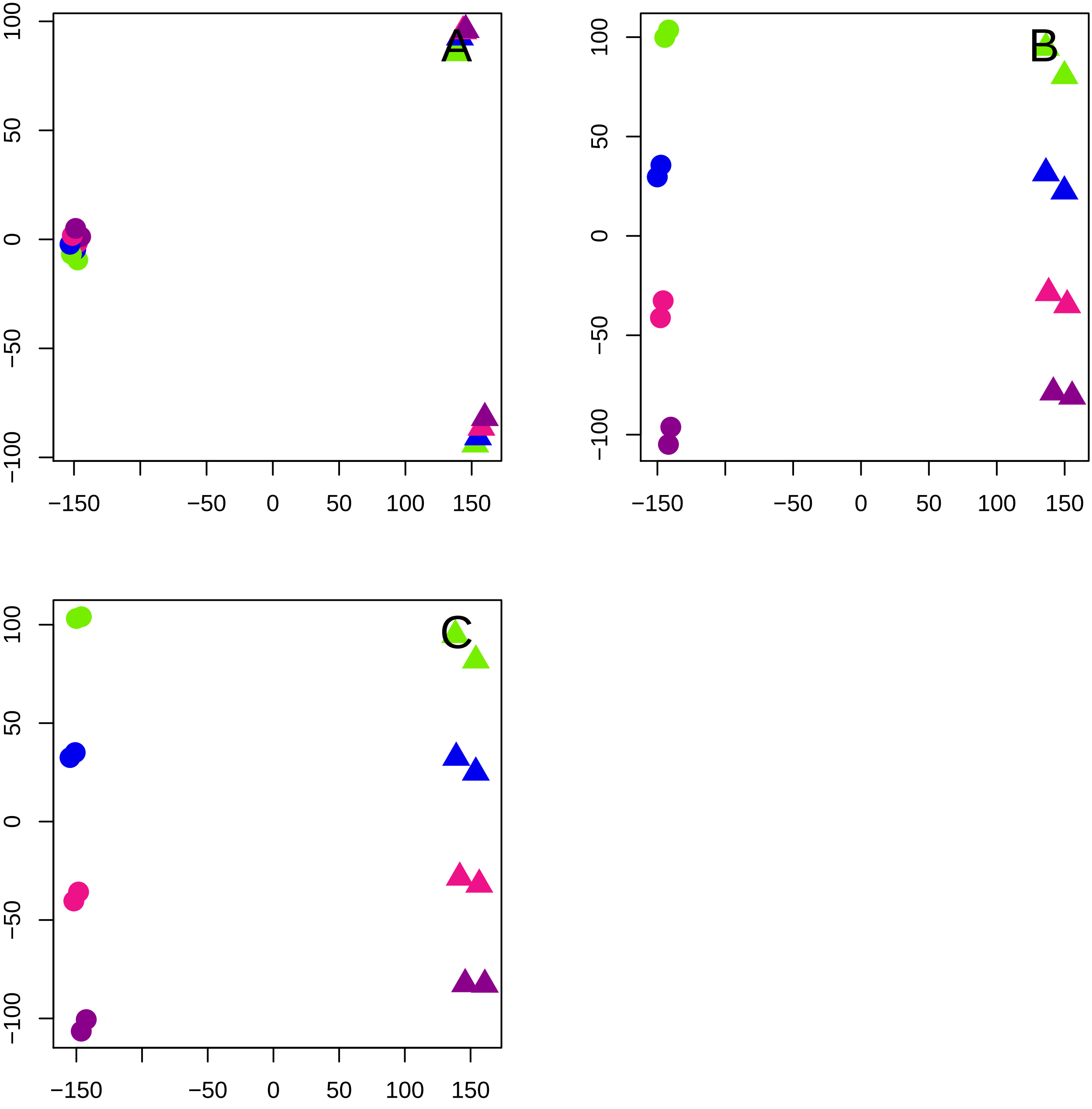

A
B
C
100
50
0
−50
−100
−150
−50
0
50
100
150

Additional File 2. Compendium of figures for data from *Candida*-infected mouse vaginas with results separated by read pairing status. A heatmap with hierarchical clustering with statistical support is shown on page 1 with the condition denoted according to letter code from Supplementary Table 2, followed by the replicate designation and the read length. A PCA plot is shown on page 2 where the conditions are denoted by the shape (circle, CA_d3; triangle, naïve_d3) and the read length by the color (green, 36 bp; blue, 54 bp; magenta, 72 bp; purple, 101 bp). On both pages, results are shown in the three panels for (A) paired end reads, (B) first-in-pair single end reads, and (C) second-in-pair single reads.

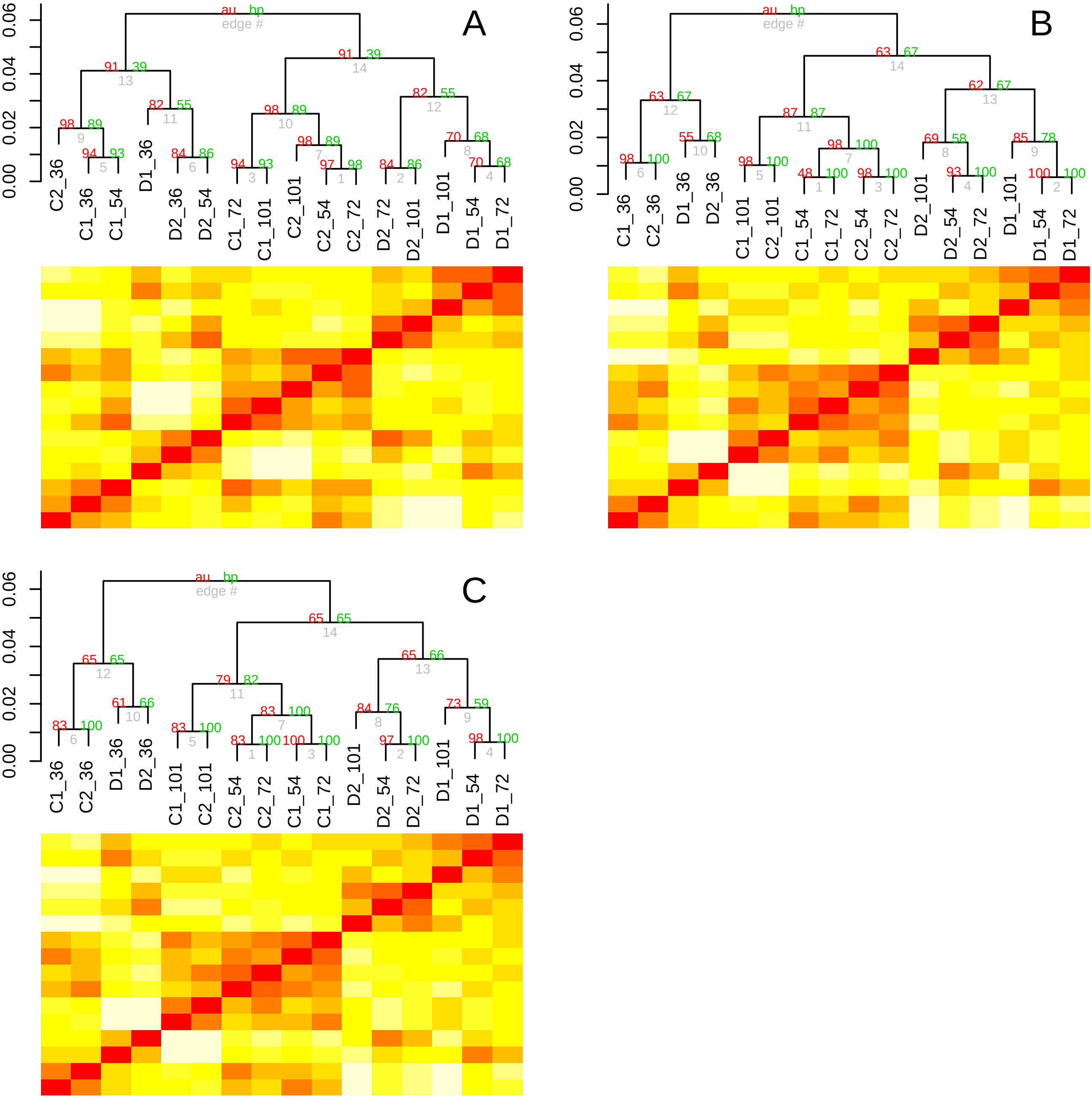
A
B
C
au
bp
edge #
0.00
0.02
0.04
0.06
C2_36
C1_36
C1_54
D1_36
D2_36
D2_54
C1_72
C1_101
C2_101
C2_54
C2_72
D2_72
D2_101
D1_101
D1_54
D1_72
C1_36
C2_36
D1_36
D2_36
C1_101
C2_101
C1_54
C1_72
C2_54
C2_72
D2_101
D2_54
D2_72
D1_101
D1_54
D1_72
C1_36
C2_36
D1_36
D2_36
C1_101
C2_101
C2_54
C2_72
C1_54
C1_72
D2_101
D2_54
D2_72
D1_101
D1_54
D1_72

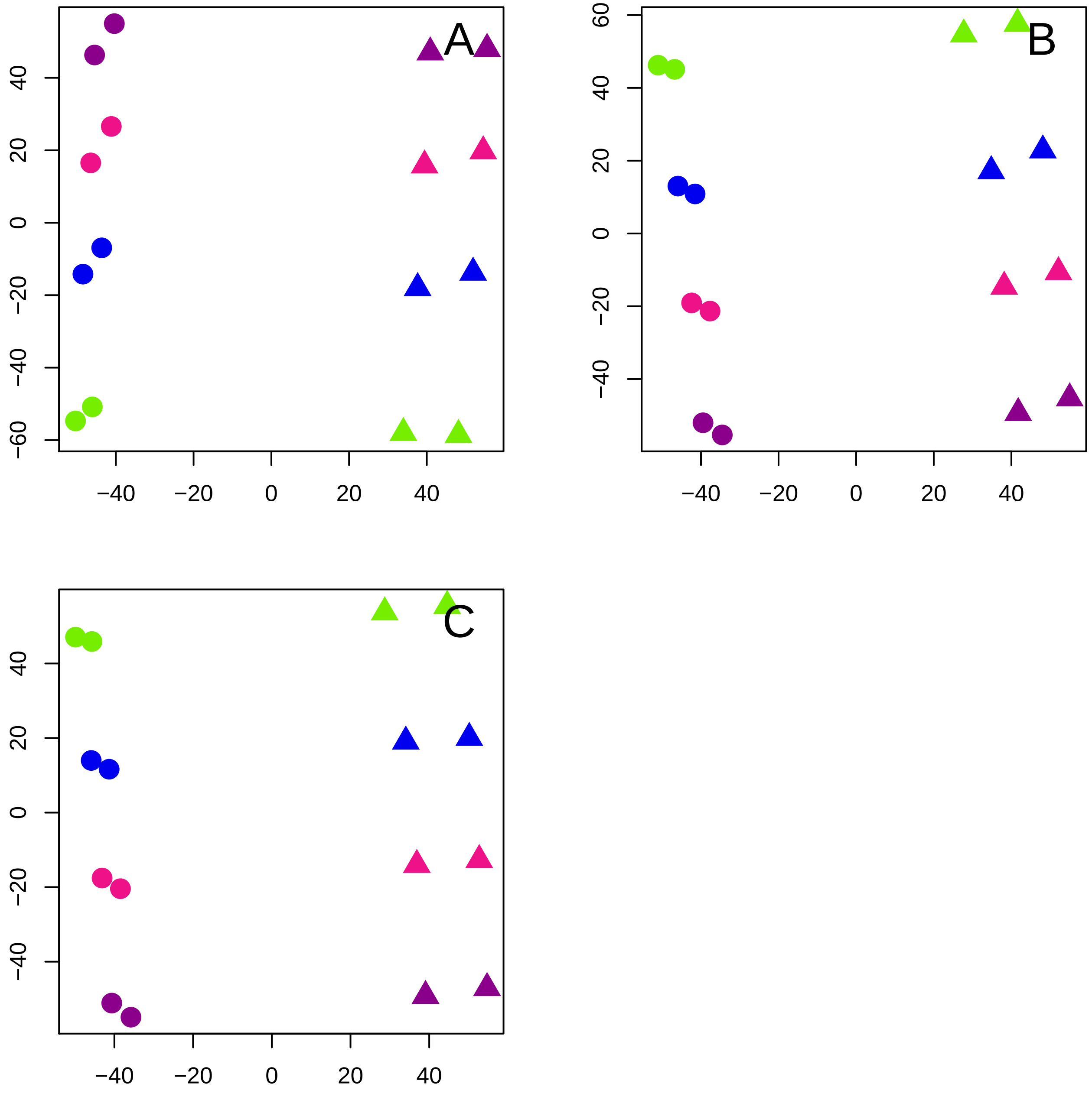
A
B
C
−60
−40
−20
0
20
40
60

Additional File 3. Compendium of figures for *A. fumigatus* data with results separated by read pairing status. A heatmap with hierarchical clustering with statistical support is shown on page 1 with the condition denoted according to letter code from Supplementary Table 2, followed by the replicate designation and the read length. A PCA plot is shown on page 2 where the conditions are denoted by the shape (circle, 1_6h_AF293; triangle, 4_6h_AF293) and the read length by the color (green, 36 bp; blue, 54 bp; magenta, 72 bp; purple, 101 bp). On both pages, results are shown in the three panels for (A) paired end reads, (B) first-in-pair single end reads, and (C) second-in-pair single reads.

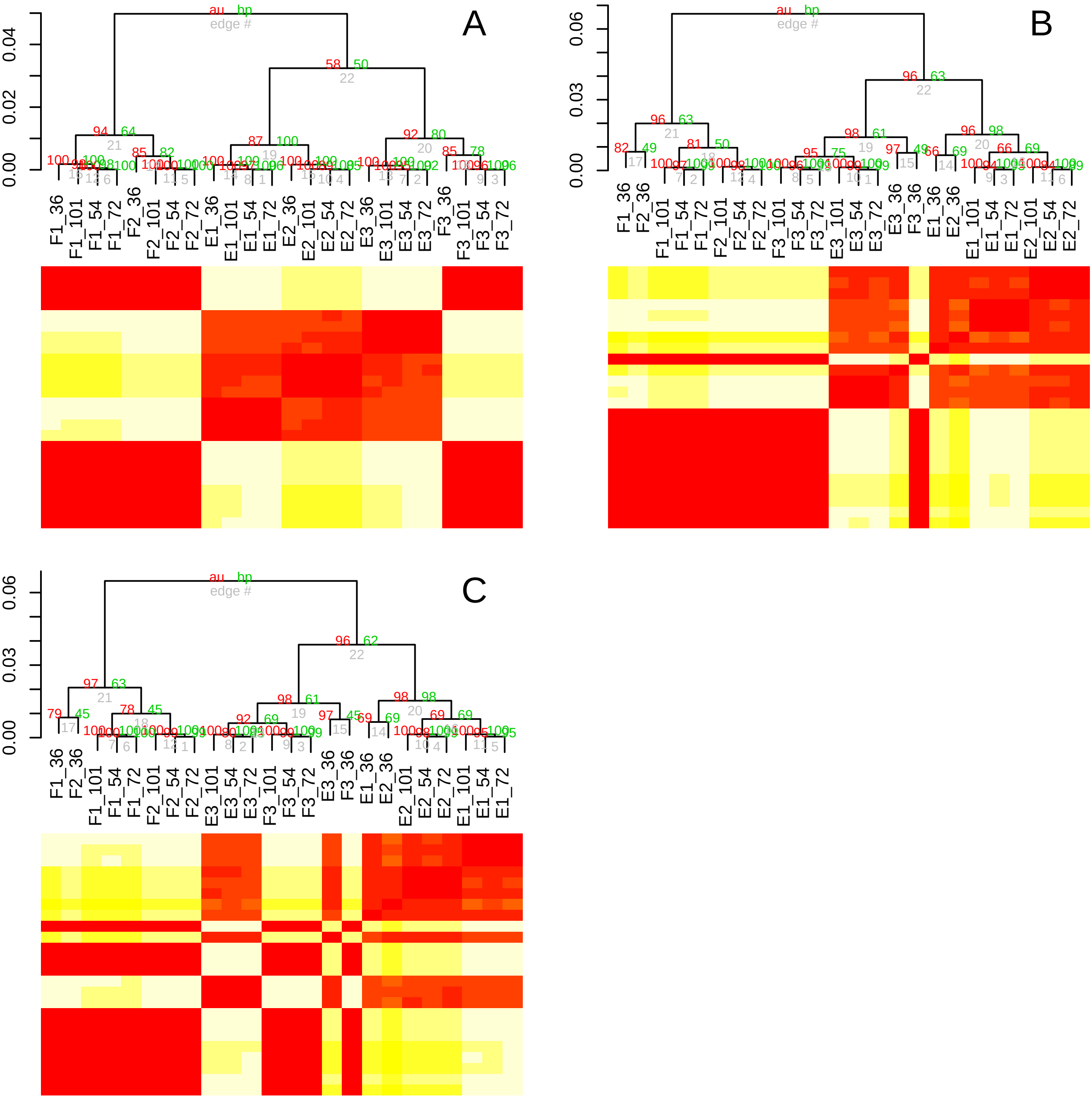
A
B
C
au
bp
edge #
0.00
0.02
0.04
0.03
0.06
F1_36
F1_101
F1_54
F1_72
F2_36
F2_101
F2_54
F2_72
E1_36
E1_101
E1_54
E1_72
E2_36
E2_101
E2_54
E2_72
E3_36
E3_101
E3_54
E3_72
F3_36
F3_101
F3_54
F3_72

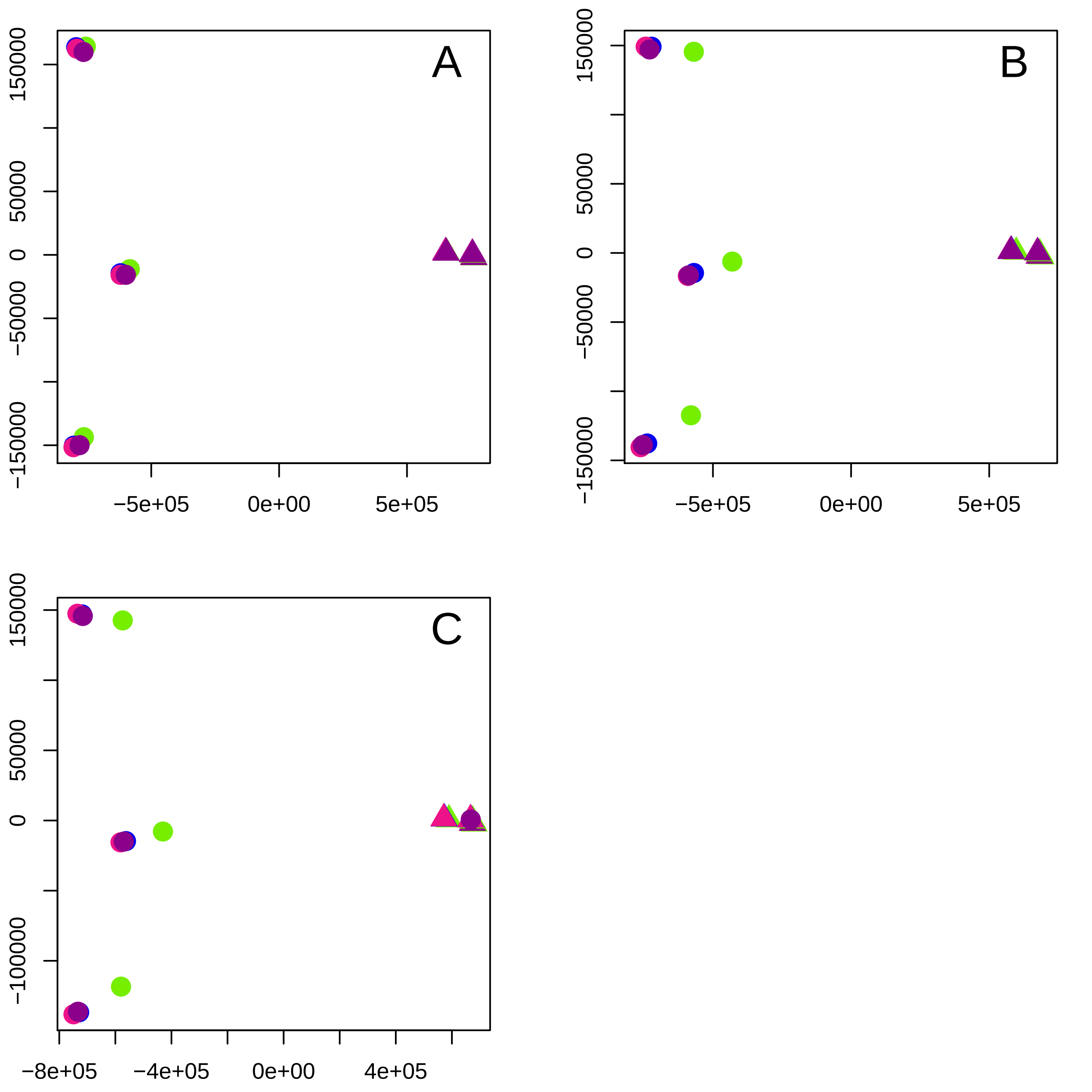
A
B
C
150000
50000
0
−50000
−150000
−100000
−5e+05
0e+00
5e+05
−8e+05
−4e+05
4e+05

Additional File 4. Compendium of figures for *Candida*-human data with results separated by read pairing status. A heatmap with hierarchical clustering with statistical support is shown on page 1 with the condition denoted according to letter code from Supplementary Table 2, followed by the replicate designation and the read length. A PCA plot is shown on page 2 where the conditions are denoted by the shape (circle, 5h_c; triangle, 5h_oc) and the read length by the color (green, 36 bp; blue, 54 bp; magenta, 72 bp; purple, 101 bp). On both pages, results are shown in the three panels for (A) paired end reads, (B) first-in-pair single end reads, and (C) second-in-pair single reads.

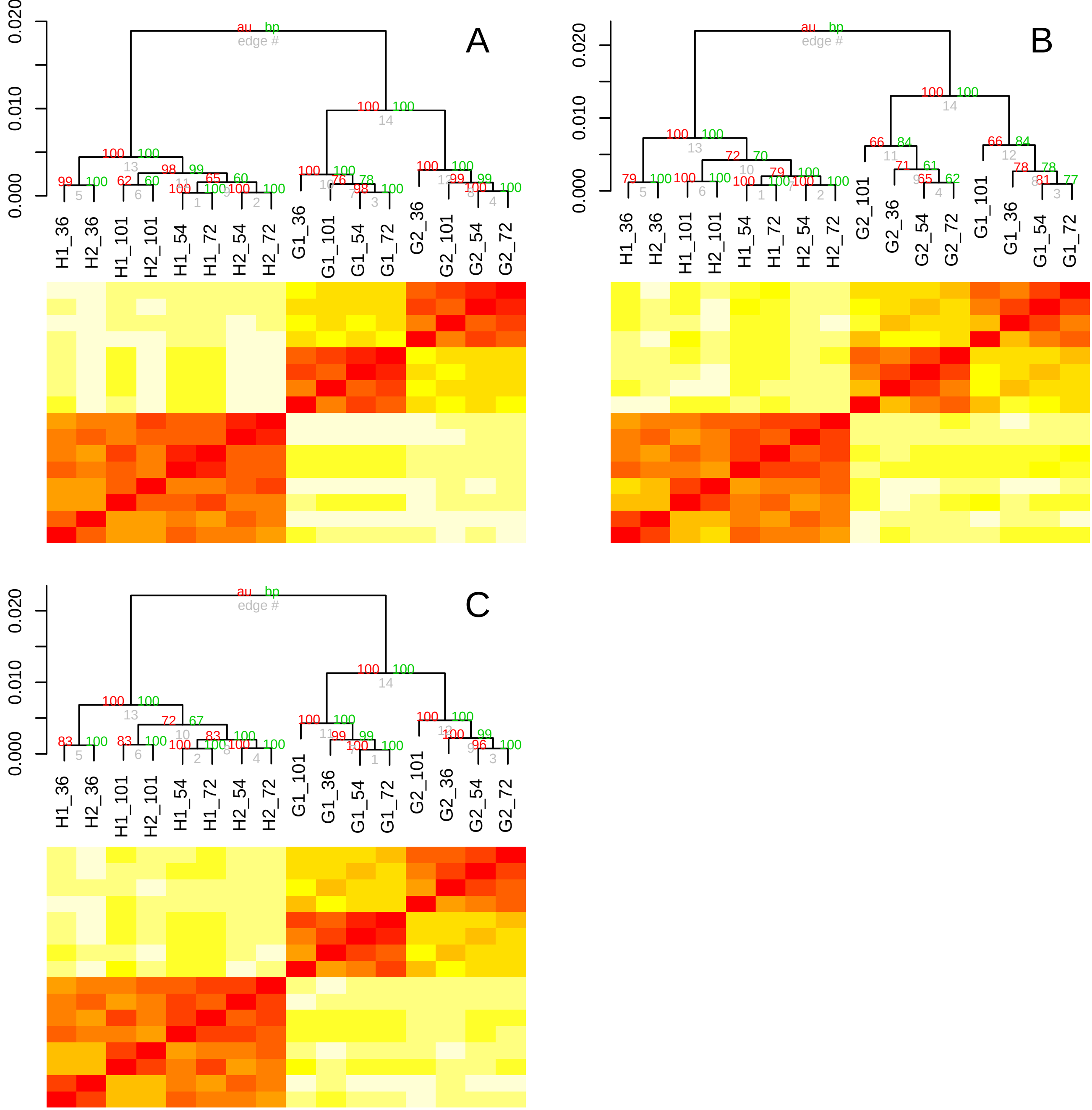
A
B
C
au
bp
edge #
0.000
0.010
0.020
H1_36
H2_36
H1_101
H2_101
H1_54
H1_72
H2_54
H2_72
G1_36
G1_101
G1_54
G1_72
G2_36
G2_101
G2_54
G2_72

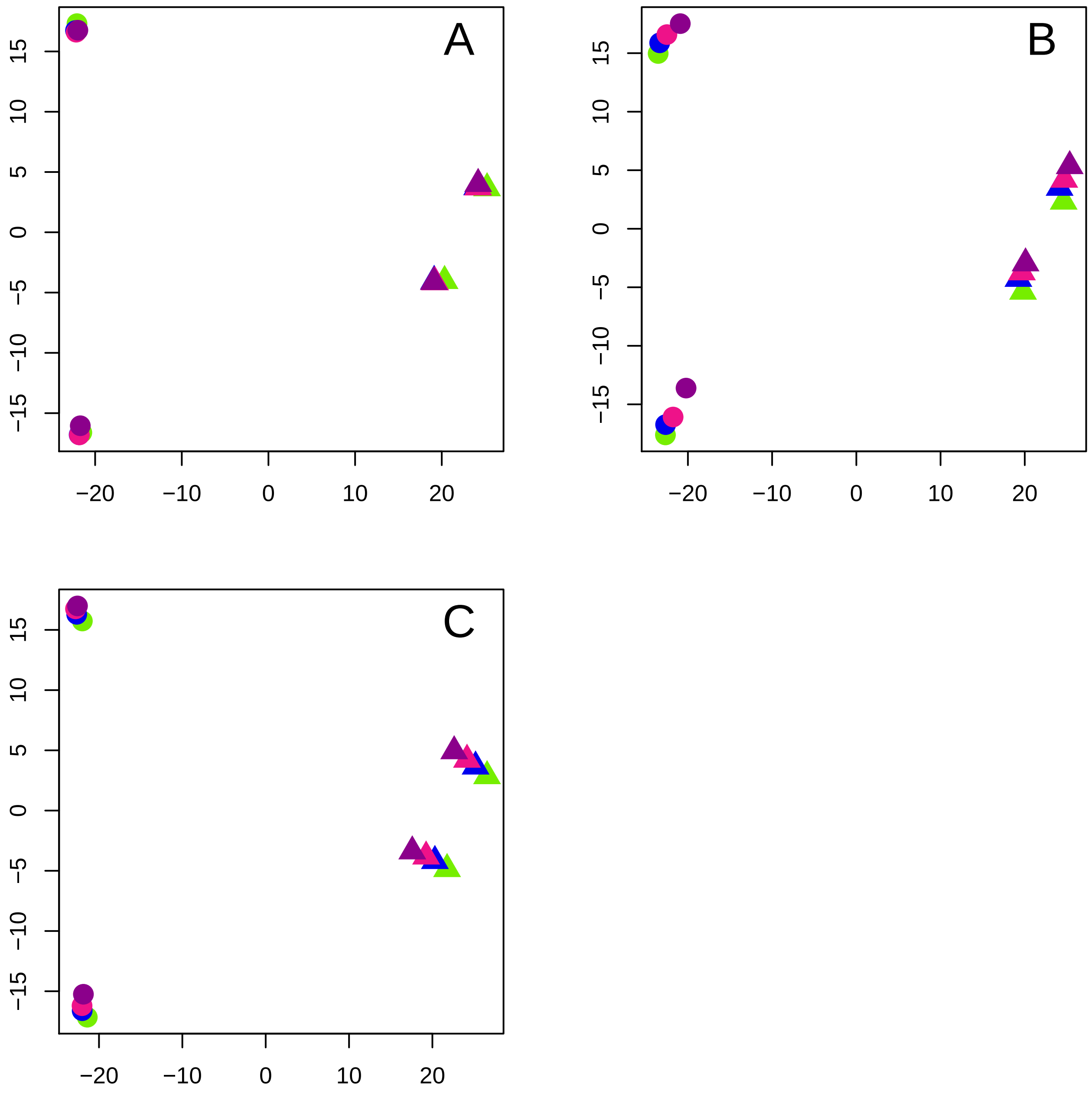
A
B
C
15
10
5
0
−5
−10
−15
−20
−10
0
10
20

Additional File 5. Compendium of figures for *Candida*-only data with results separated by read pairing status. A heatmap with hierarchical clustering with statistical support is shown on page 1 with the condition denoted according to letter code from Supplementary Table 2, followed by the replicate designation and the read length. A PCA plot is shown on page 2 where the conditions are denoted by the shape (circle, rhr2_comp; triangle, rhr2_del) and the read length by the color (green, 36 bp; blue, 54 bp; magenta, 72 bp; purple, 101 bp). On both pages, results are shown in the three panels for (A) paired end reads, (B) first-in-pair single end reads, and (C) second-in-pair single reads.

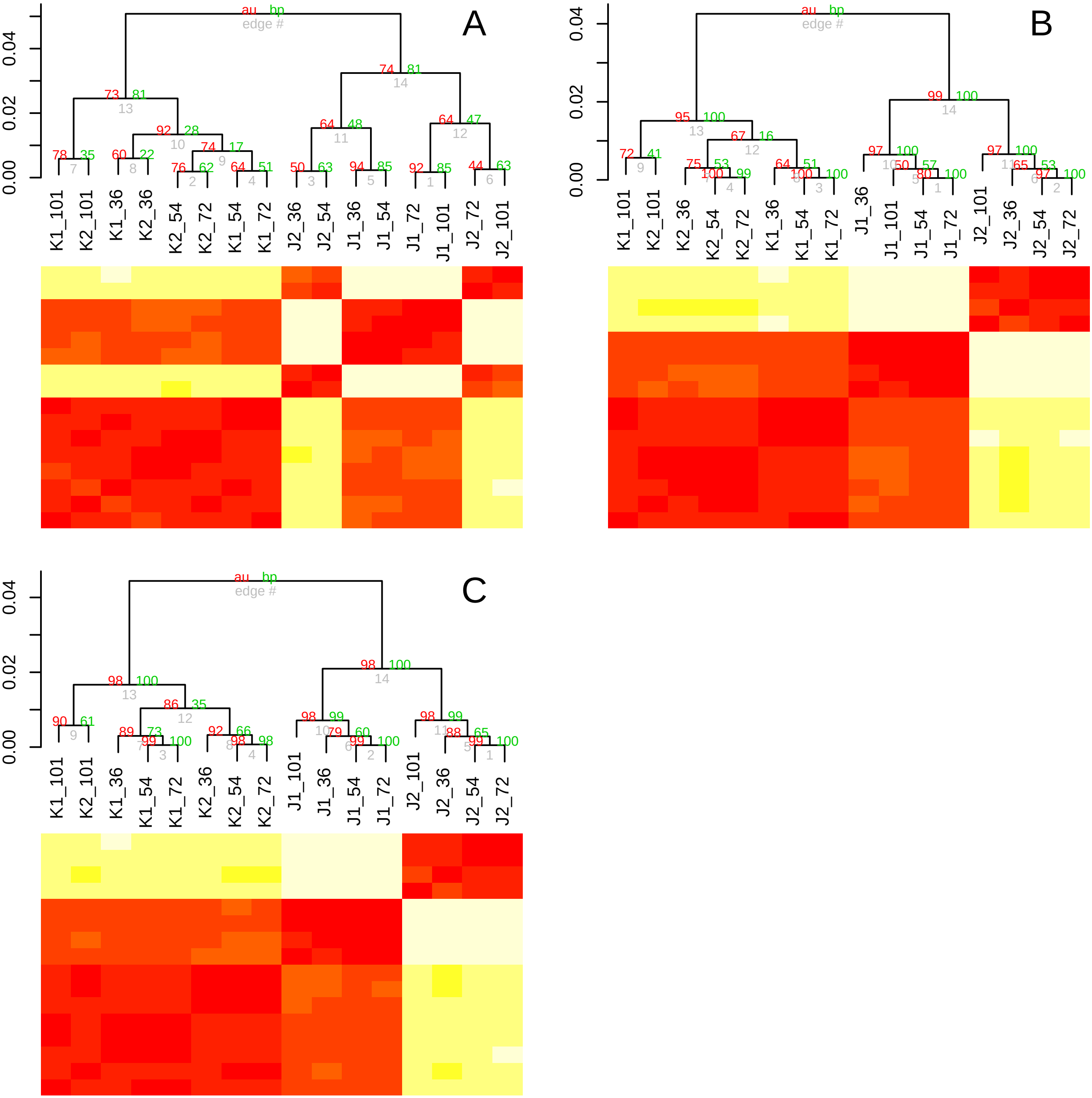
A
B
C
0.00
0.02
0.04
au
bp
edge #
K1_101 K2_101 K1_36 K2_36 K2_54 K2_72 K1_54 K1_72 J2_36 J2_54 J1_36 J1_54 J1_72 J1_101 J2_72 J2_101
K1_101 K2_101 K2_36 K2_54 K2_72 K1_36 K1_54 K1_72 J1_36 J1_101 J1_54 J1_72 J2_101 J2_36 J2_54 J2_72
K1_101 K2_101 K1_36 K1_54 K1_72 K2_36 K2_54 K2_72 J1_101 J1_36 J1_54 J1_72 J2_101 J2_36 J2_54 J2_72

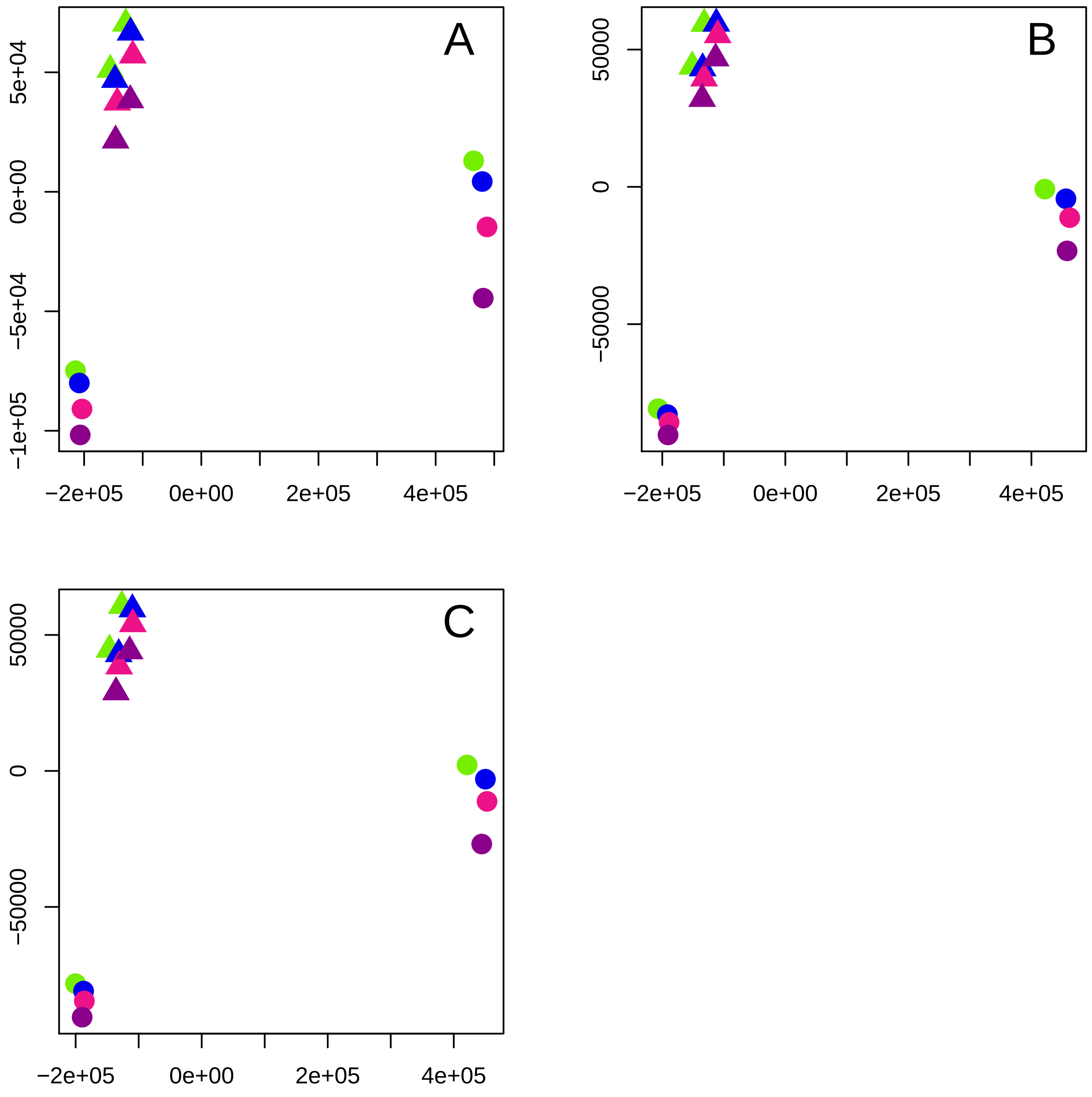
A
B
C
5e+04
0e+00
−5e+04
−1e+05
50000
0
−50000
−2e+05
0e+00
2e+05
4e+05

Additional File 6. Compendium of figures for *Es. coli* data with results separated by read pairing status. A heatmap with hierarchical clustering with statistical support is shown on page 1 with the condition denoted according to letter code from Supplementary Table 2, followed by the replicate designation and the read length. A PCA plot is shown on page 2 where the conditions are denoted by the shape (circle, DMEM; triangle, LB) and the read length by the color (green, 36 bp; blue, 54 bp; magenta, 72 bp; purple, 101 bp). On both pages, results are shown in the three panels for (A) paired end reads, (B) first-in-pair single end reads, and (C) second-in-pair single reads.

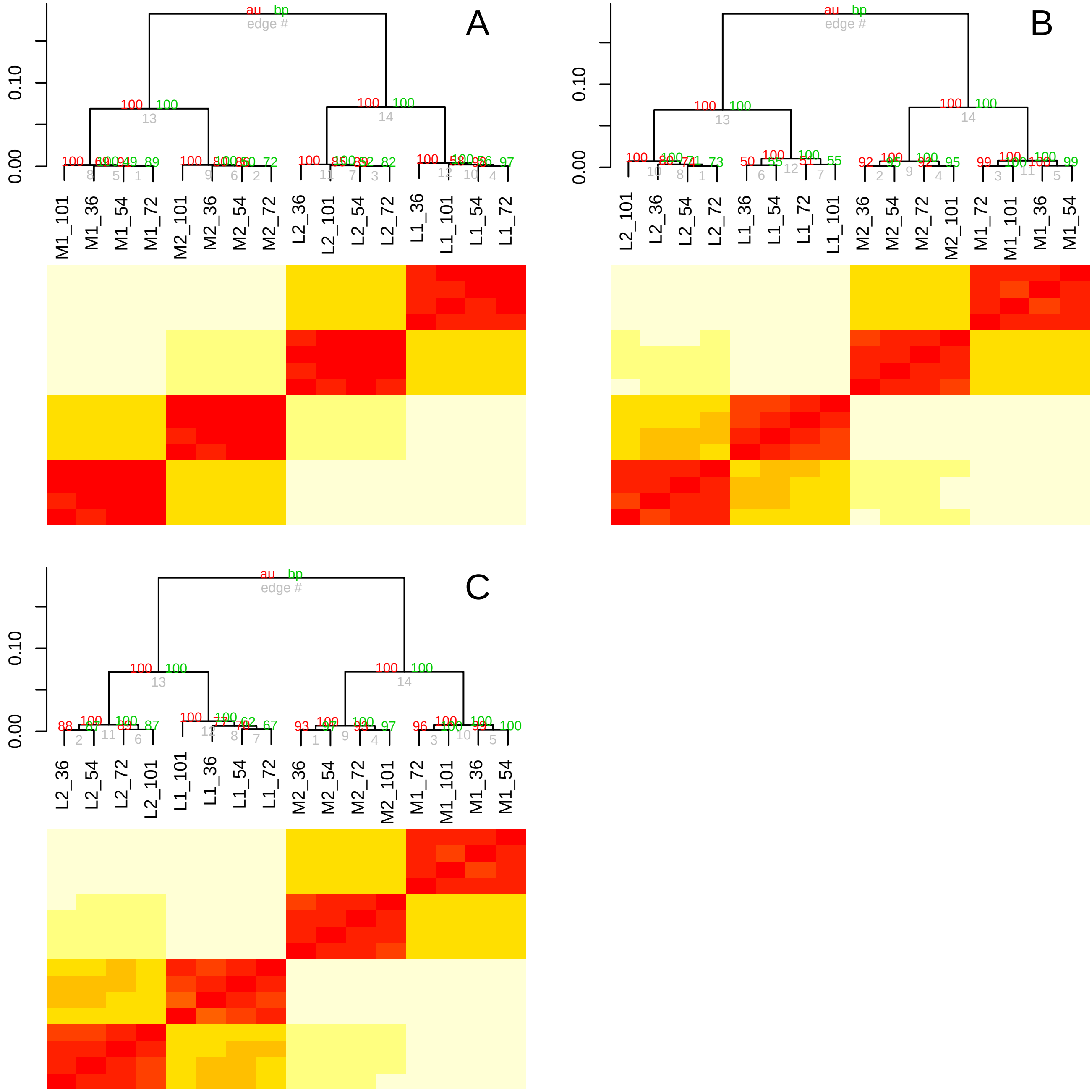

A
au bp
edge #
0.00
0.10
M1_101
M1_36
M1_54
M1_72
M2_101
M2_36
M2_54
M2_72
L2_36
L2_101
L2_54
L2_72
L1_36
L1_101
L1_54
L1_72
B
au bp
edge #
0.00
0.10
L2_101
L2_36
L2_54
L2_72
L1_36
L1_54
L1_72
L1_101
M2_36
M2_54
M2_72
M2_101
M1_72
M1_101
M1_36
M1_54
C
au bp
edge #
0.00
0.10
L2_36
L2_54
L2_72
L2_101
L1_101
L1_36
L1_54
L1_72
M2_36
M2_54
M2_72
M2_101
M1_72
M1_101
M1_36
M1_54

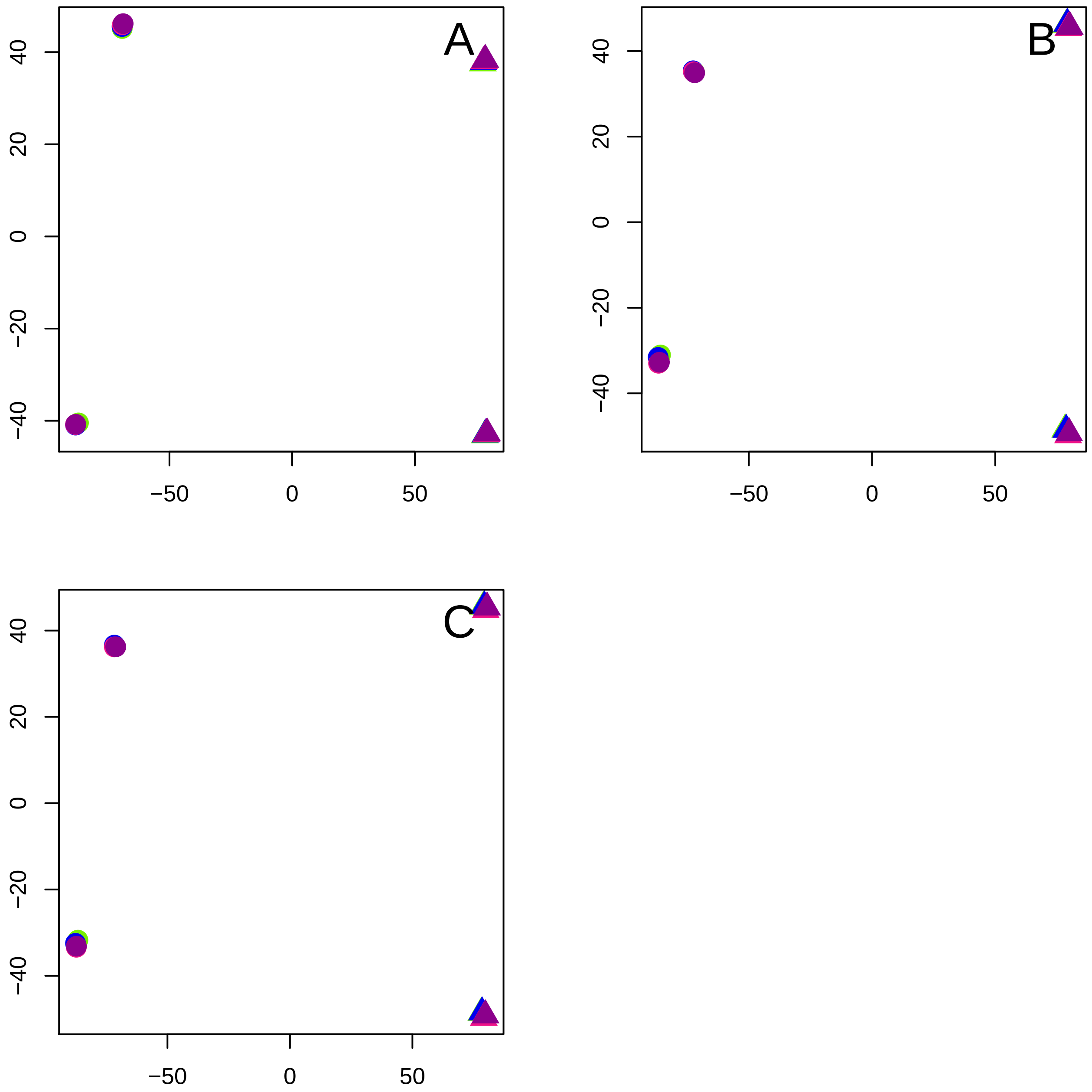
A
B
C
−50
0
50
40
20
0
−20
−40

Additional File 7. Compendium of figures for data from *Ixodes scapularis* cell lines with differential presence of *Eh. chaffeensis* strains with results separated by read pairing status. A heatmap with hierarchical clustering with statistical support is shown on page 1 with the condition denoted according to letter code from Supplementary Table 2, followed by the replicate designation and the read length. A PCA plot is shown on page 2 where the conditions are denoted by the shape and the read length by the color (green, 36 bp; blue, 54 bp; magenta, 72 bp; purple, 101 bp). On both pages, results are shown in the three panels for (A) paired end reads, (B) first-in-pair single end reads, and (C) second-in-pair single reads.

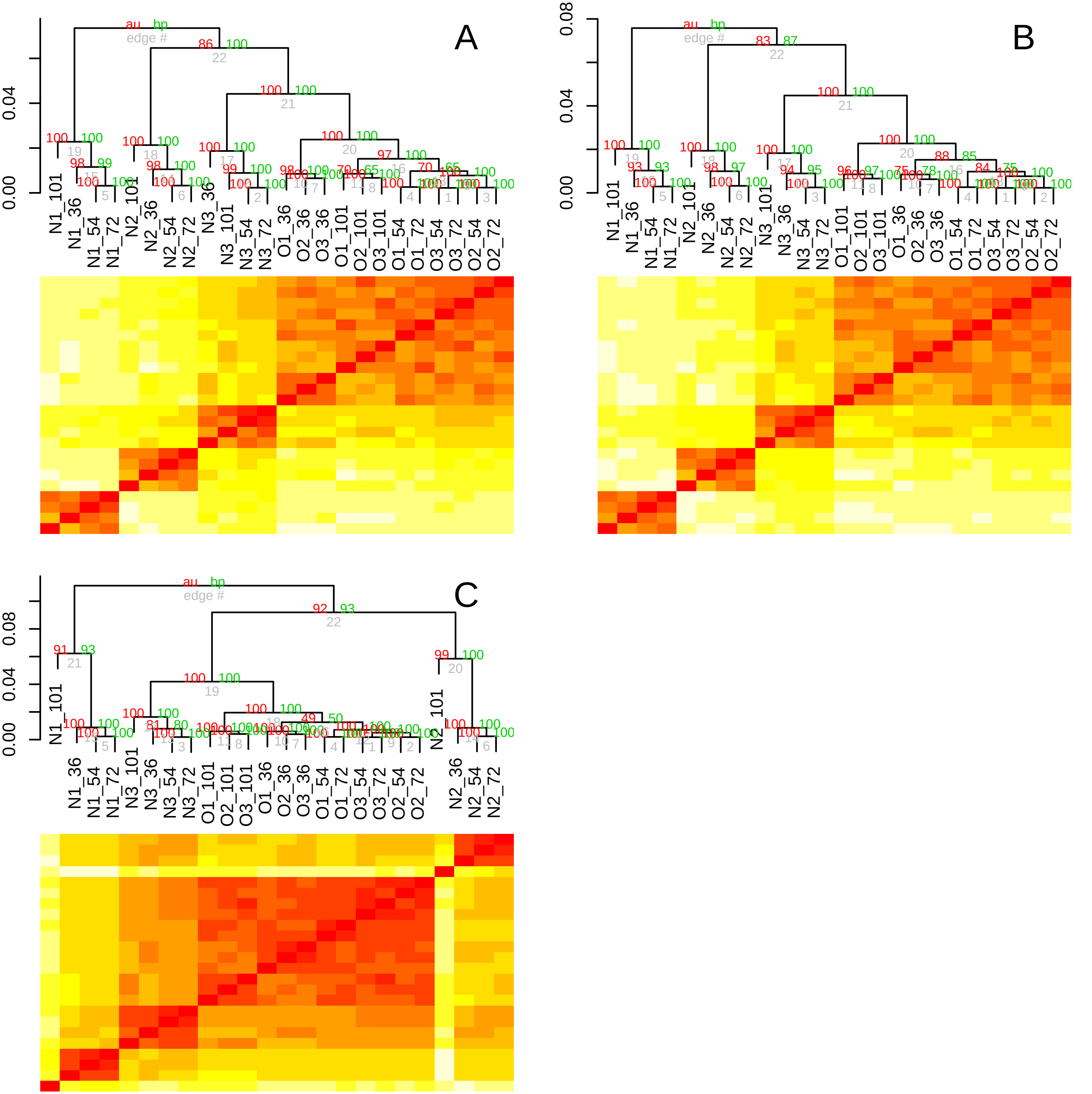
A
B
C
au
bp
edge #
0.00
0.04
0.08
N1_101
N1_36
N1_54
N1_72
N2_101
N2_36
N2_54
N2_72
N3_101
N3_36
N3_54
N3_72
O1_101
O2_101
O3_101
O1_36
O2_36
O3_36
O1_54
O1_72
O3_54
O3_72
O2_54
O2_72

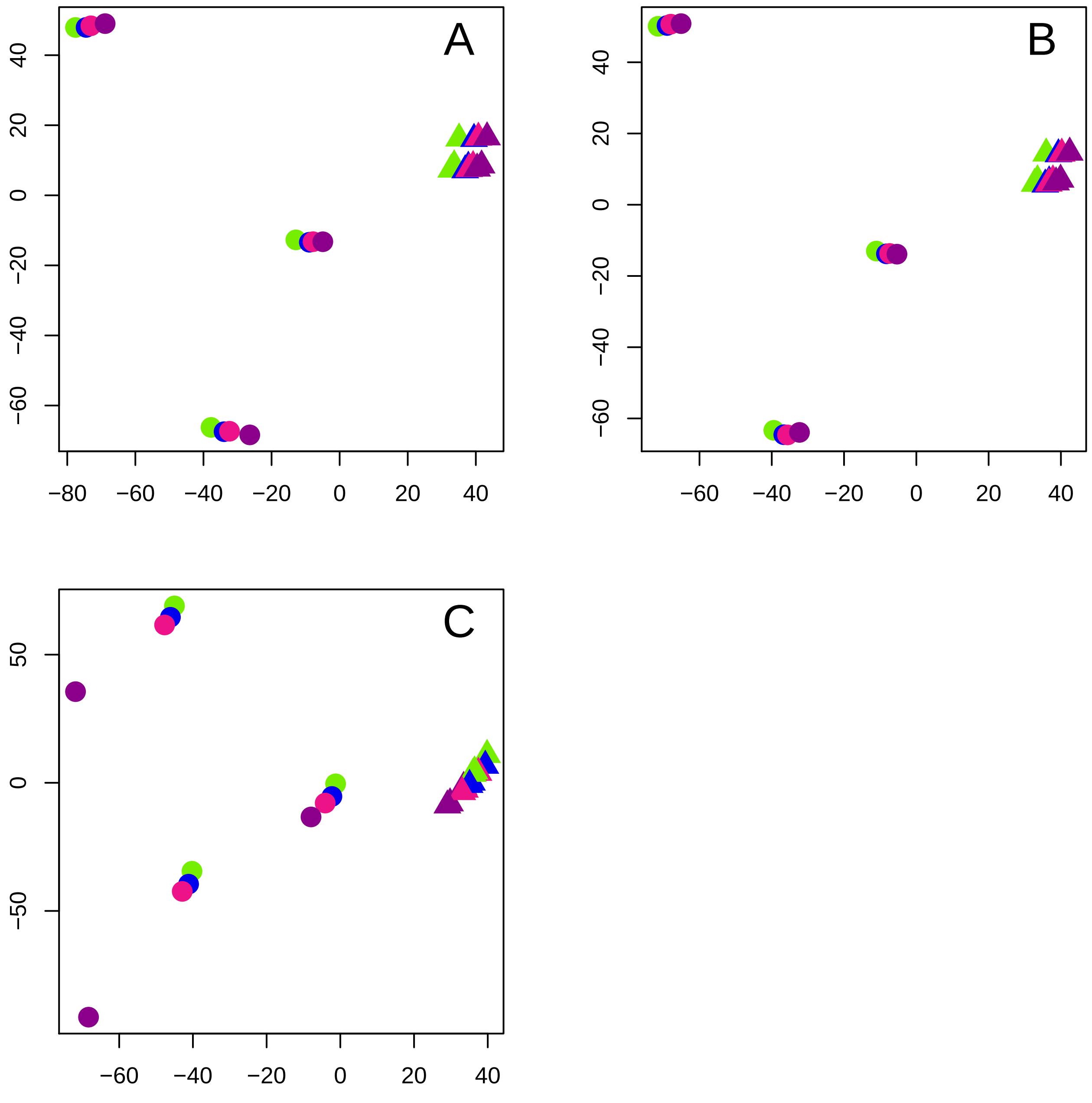
A
B
C
−80
−60
−40
−20
0
20
40
50
−50

Additional File 8. Compendium of figures for data from canine cell lines with differential presence of *Eh. chaffeensis* strains with results separated by read pairing status. A heatmap with hierarchical clustering with statistical support is shown on page 1 with the condition denoted according to letter code from Supplementary Table 2, followed by the replicate designation and the read length. A PCA plot is shown on page 2 where the conditions are denoted by the shape and the read length by the color (green, 36 bp; blue, 54 bp; magenta, 72 bp; purple, 101 bp). On both pages, results are shown in the three panels for (A) paired end reads, (B) first-in-pair single end reads, and (C) second-in-pair single reads.

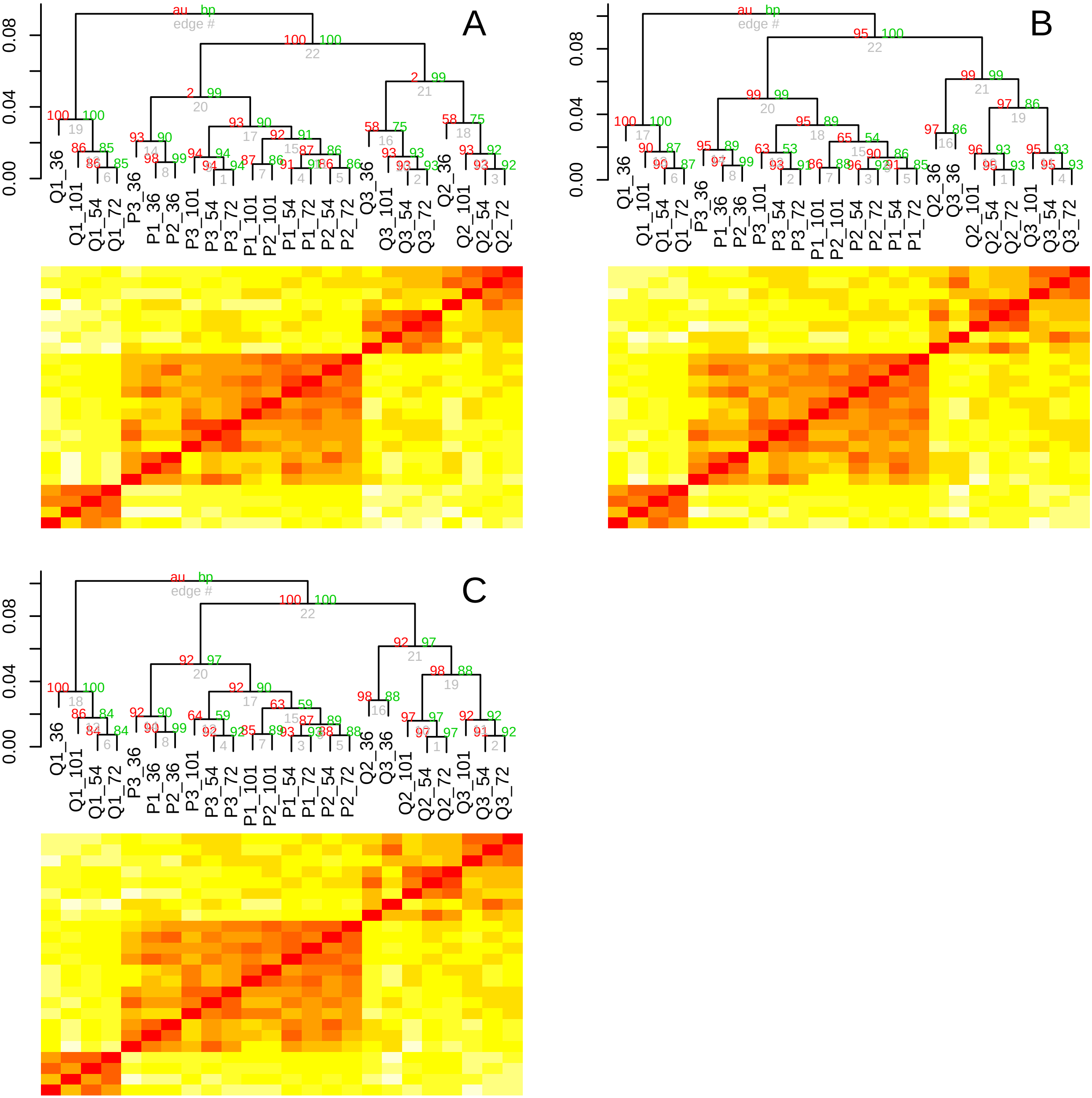
A
B
C
au
bp
edge #
0.00
0.04
0.08
Q1_36
Q1_101
Q1_54
Q1_72
P3_36
P1_36
P2_36
P3_101
P3_54
P3_72
P1_101
P2_101
P1_54
P1_72
P2_54
P2_72
Q3_36
Q3_101
Q3_54
Q3_72
Q2_36
Q2_101
Q2_54
Q2_72

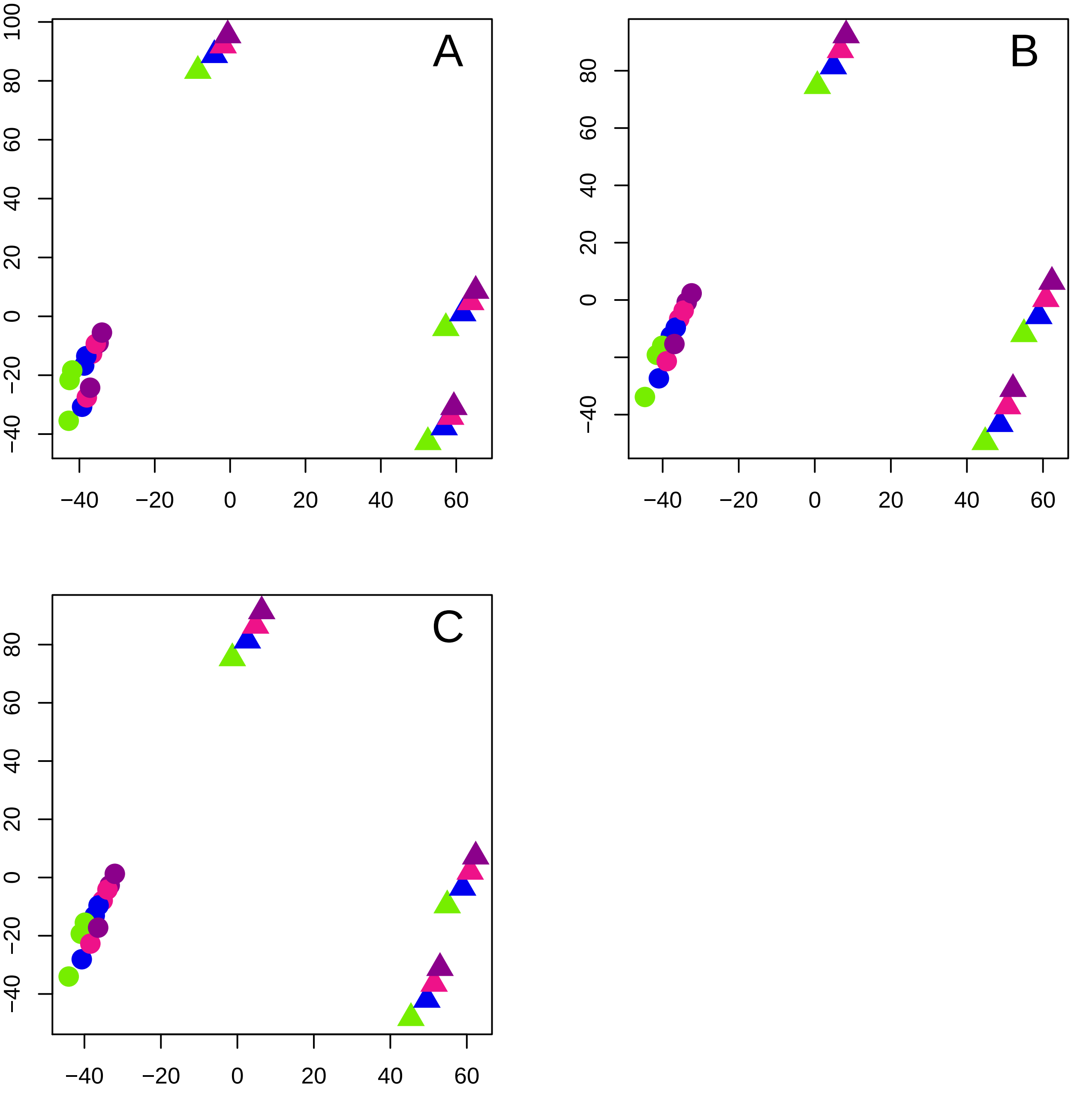
A
B
C
100
80
60
40
20
0
−20
−40
−20
0
20
40
60

Additional File 9. Compendium of figures for data from the *Wolbachia* endosymbiont *w*Bm in adult male and adult female *B. malayi* hosts with results separated by read pairing status. A heatmap with hierarchical clustering with statistical support is shown on page 1 with the condition denoted according to letter code from Supplementary Table 2, followed by the replicate designation and the read length. A PCA plot is shown on page 2 where the conditions are denoted by the shape and the read length by the color (green, 36 bp; blue, 54 bp; magenta, 72 bp; purple, 101 bp). On both pages, results are shown in the three panels for (A) paired end reads, (B) first-in-pair single end reads, and (C) second-in-pair single reads.

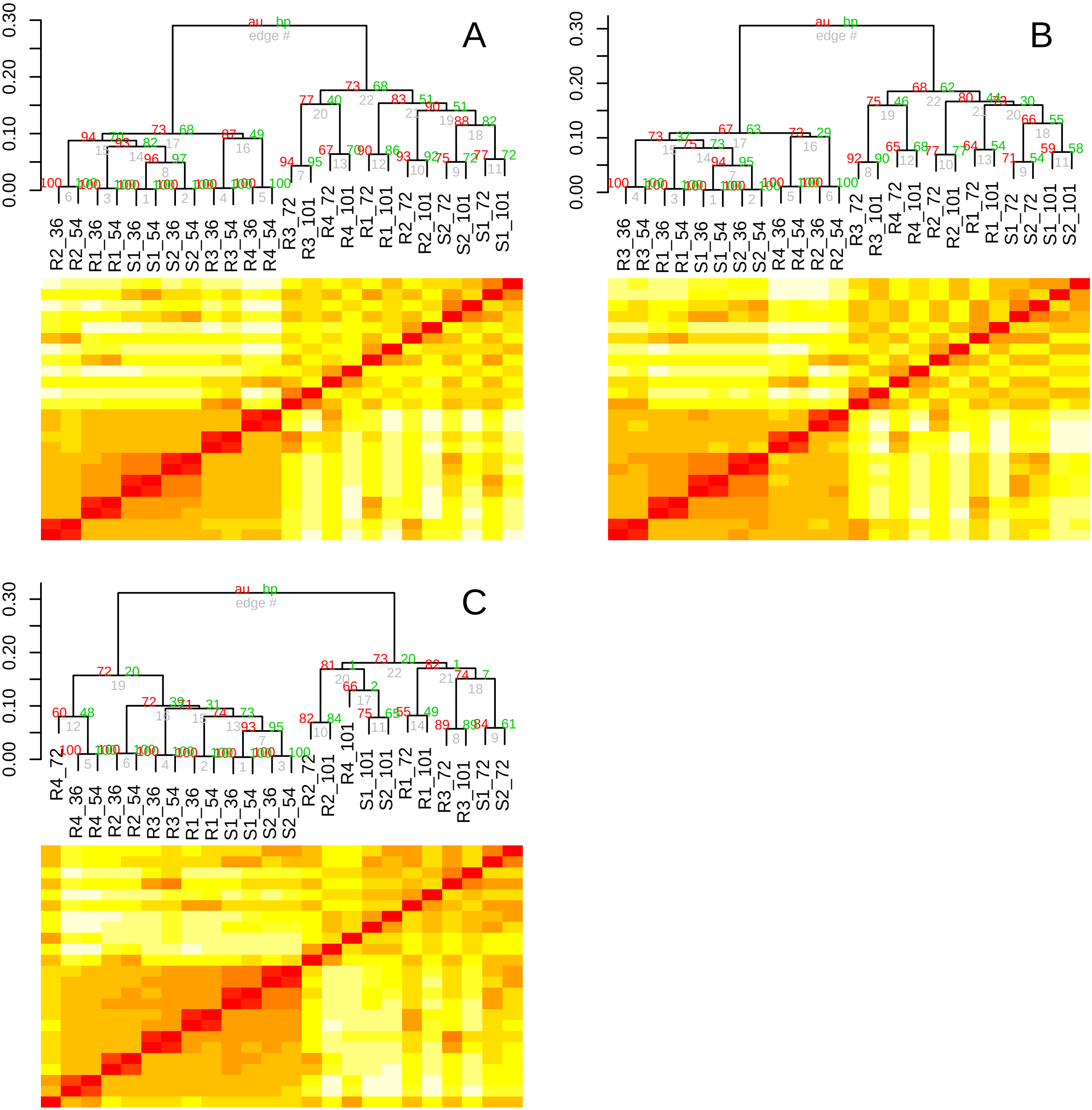
A
B
C
au bp
edge #
0.00 0.10 0.20 0.30
R2_36 R2_54 R1_36 R1_54 S1_36 S1_54 S2_36 S2_54 R3_36 R3_54 R4_36 R4_54 R3_72 R3_101 R4_72 R4_101 R1_72 R1_101 R2_72 R2_101 S2_72 S2_101 S1_72 S1_101
R3_36 R3_54 R1_36 R1_54 S1_36 S1_54 S2_36 S2_54 R4_36 R4_54 R2_36 R2_54 R3_72 R3_101 R4_72 R4_101 R2_72 R2_101 R1_72 R1_101 S1_72 S2_72 S1_101 S2_101
R4_72 R4_36 R4_54 R2_36 R2_54 R3_36 R3_54 R1_36 R1_54 S1_36 S1_54 S2_36 S2_54 R2_72 R2_101 R4_101 S1_101 S2_101 R1_72 R1_101 R3_72 R3_101 S1_72 S2_72

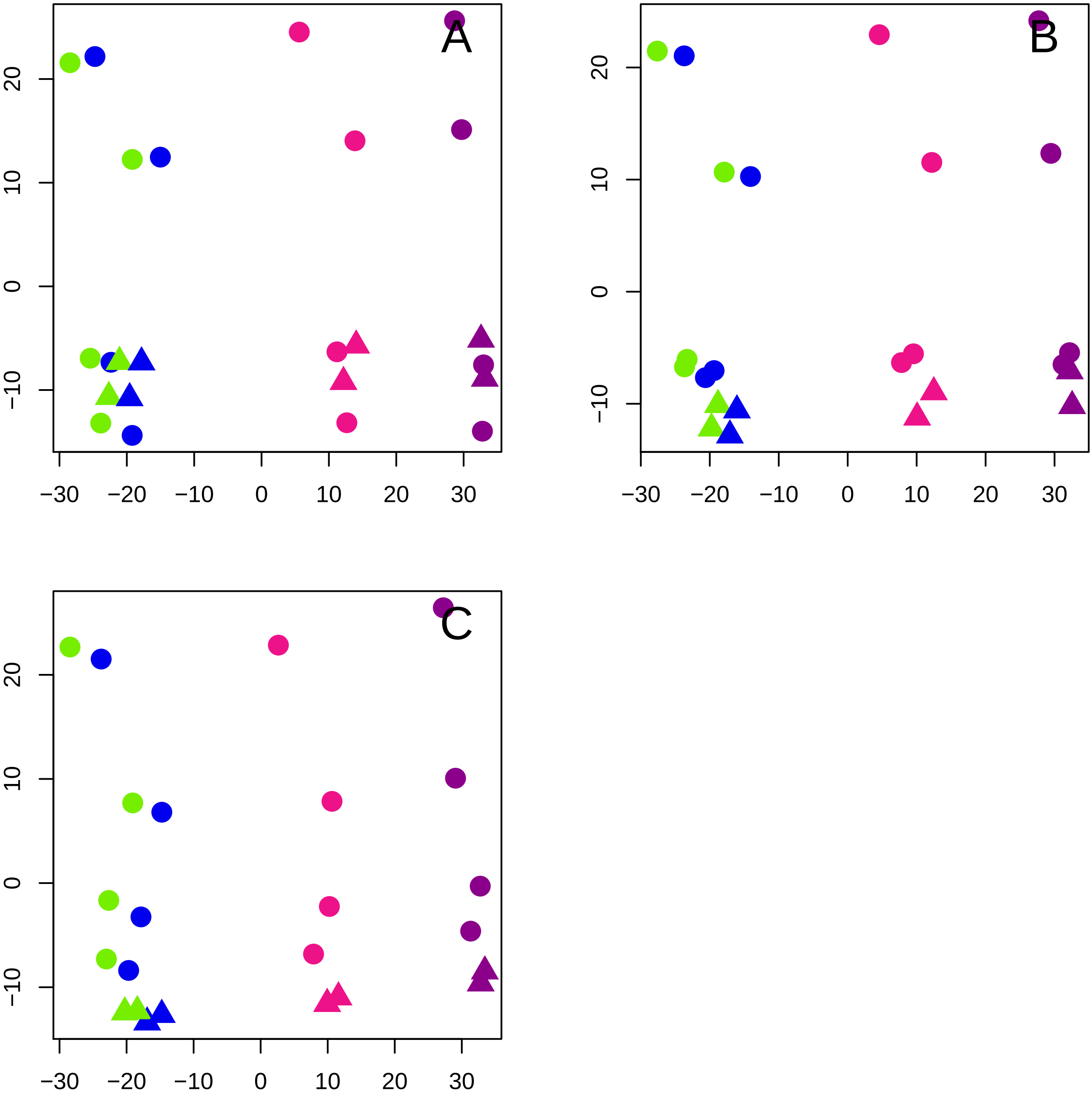
A
B
C
−30
−20
−10
0
10
20
30
−10
0
10
20

Additional File 10. Compendium of figures for data from adult male and adult female *B. malayi* with results separated by read pairing status. A heatmap with hierarchical clustering with statistical support is shown on page 1 with the condition denoted according to letter code from Supplementary Table 2, followed by the replicate designation and the read length. A PCA plot is shown on page 2 where the conditions are denoted by the shape and the read length by the color (green, 36 bp; blue, 54 bp; magenta, 72 bp; purple, 101 bp). On both pages, results are shown in the three panels for (A) paired end reads, (B) first-in-pair single end reads, and (C) second-in-pair single reads.

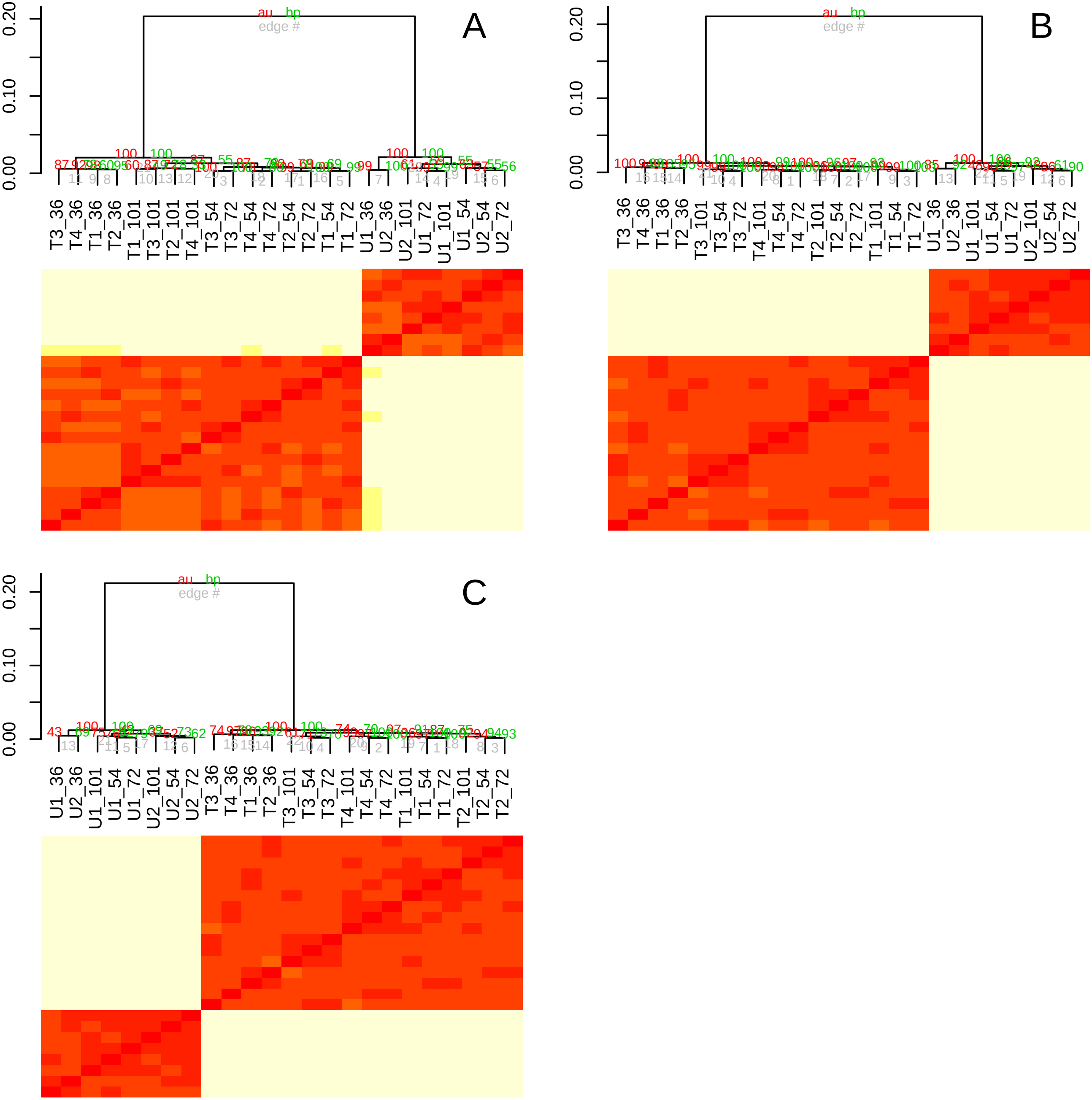

A
B
C
au bp
edge #
0.00
0.10
0.20
T3_36 T4_36 T1_36 T2_36 T1_101 T3_101 T2_101 T4_101 T3_54 T3_72 T4_54 T4_72 T2_54 T2_72 T1_54 T1_72 U1_36 U2_36 U2_101 U1_72 U1_101 U1_54 U2_54 U2_72
T3_36 T4_36 T1_36 T2_36 T3_101 T3_54 T3_72 T4_101 T4_54 T4_72 T2_101 T2_54 T2_72 T1_101 T1_54 T1_72 U1_36 U2_36 U1_101 U1_54 U1_72 U2_101 U2_54 U2_72
U1_36 U2_36 U1_101 U1_54 U1_72 U2_101 U2_54 U2_72 T3_36 T4_36 T1_36 T2_36 T3_101 T3_54 T3_72 T4_101 T4_54 T4_72 T1_101 T1_54 T1_72 T2_101 T2_54 T2_72

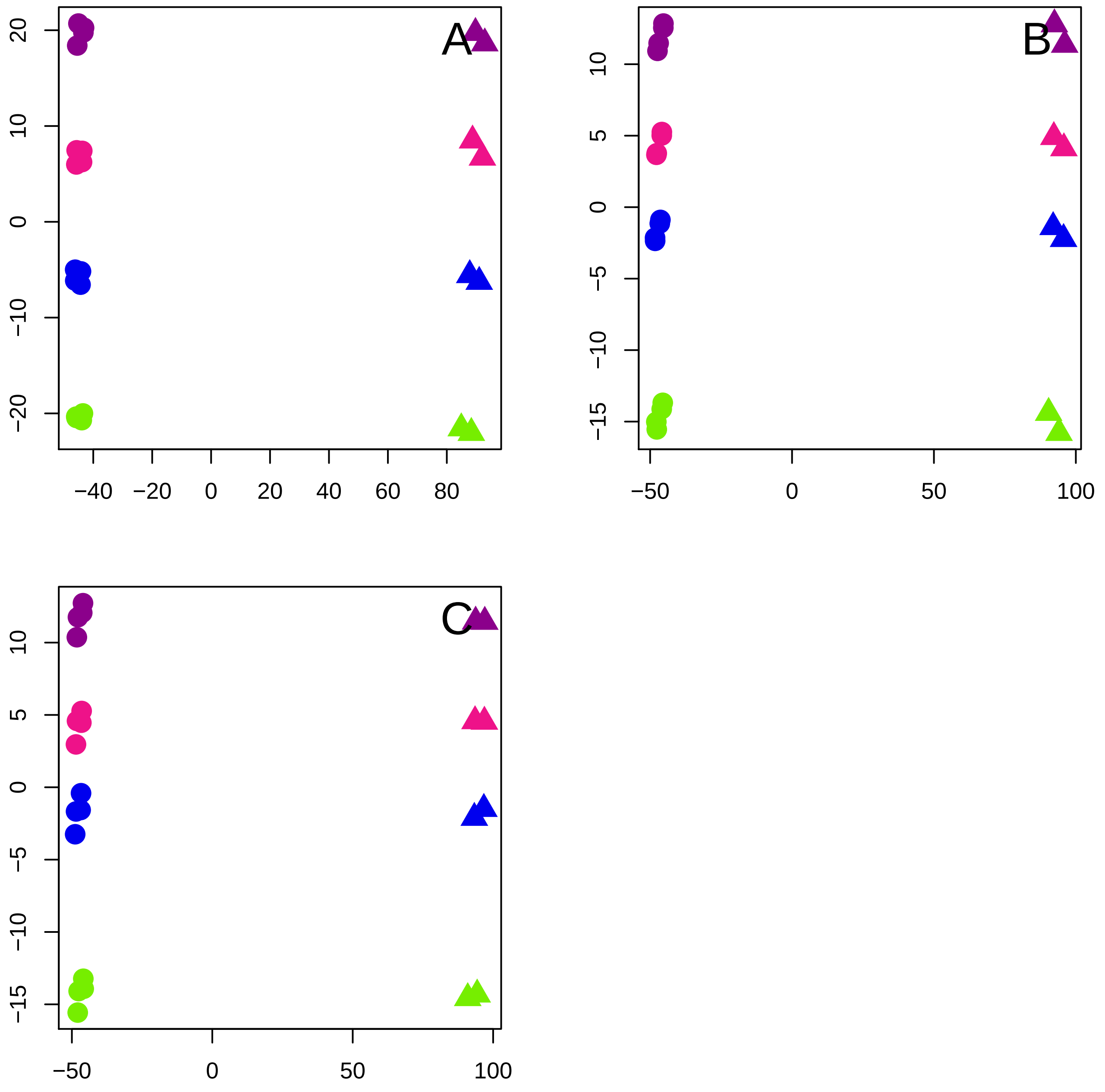
A
B
C
20
10
0
−10
−20
−40
−20
0
20
40
60
80
5
−5
−15
−50
50
100

Additional File 11. Compendium of figures for data from *H. pylori* at two time points post infection of a human cell line with results separated by read pairing status. A heatmap with hierarchical clustering with statistical support is shown on page 1 with the condition denoted according to letter code from Supplementary Table 2, followed by the replicate designation and the read length. A PCA plot is shown on page 2 where the conditions are denoted by the shape and the read length by the color (green, 36 bp; blue, 54 bp; magenta, 72 bp; purple, 101 bp). On both pages, results are shown in the three panels for (A) paired end reads, (B) first-in-pair single end reads, and (C) second-in-pair single reads.

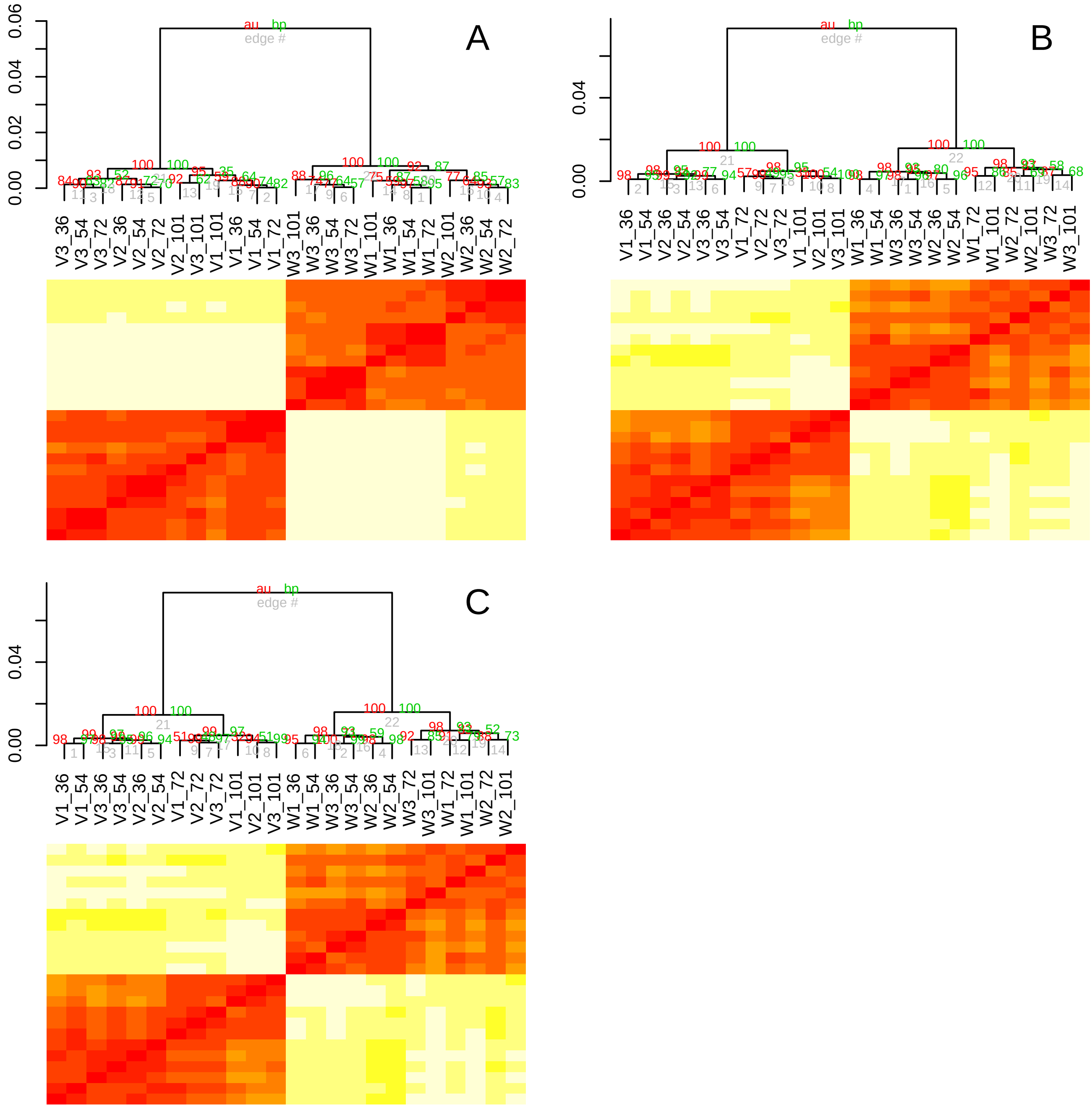

A
B
C
au
bp
edge #
0.00
0.02
0.04
0.06
V3_36 V3_54 V3_72 V2_36 V2_54 V2_72 V2_101 V3_101 V1_101 V1_36 V1_54 V1_72 W3_101 W3_36 W3_54 W3_72 W1_101 W1_36 W1_54 W1_72 W2_101 W2_36 W2_54 W2_72
V1_36 V1_54 V2_36 V2_54 V3_36 V3_54 V1_72 V2_72 V3_72 V1_101 V2_101 V3_101 W1_36 W1_54 W3_36 W3_54 W2_36 W2_54 W1_72 W1_101 W2_72 W2_101 W3_72 W3_101
V1_36 V1_54 V3_36 V3_54 V2_36 V2_54 V1_72 V2_72 V3_72 V1_101 V2_101 V3_101 W1_36 W1_54 W3_36 W3_54 W2_36 W2_54 W3_72 W3_101 W1_72 W1_101 W2_72 W2_101

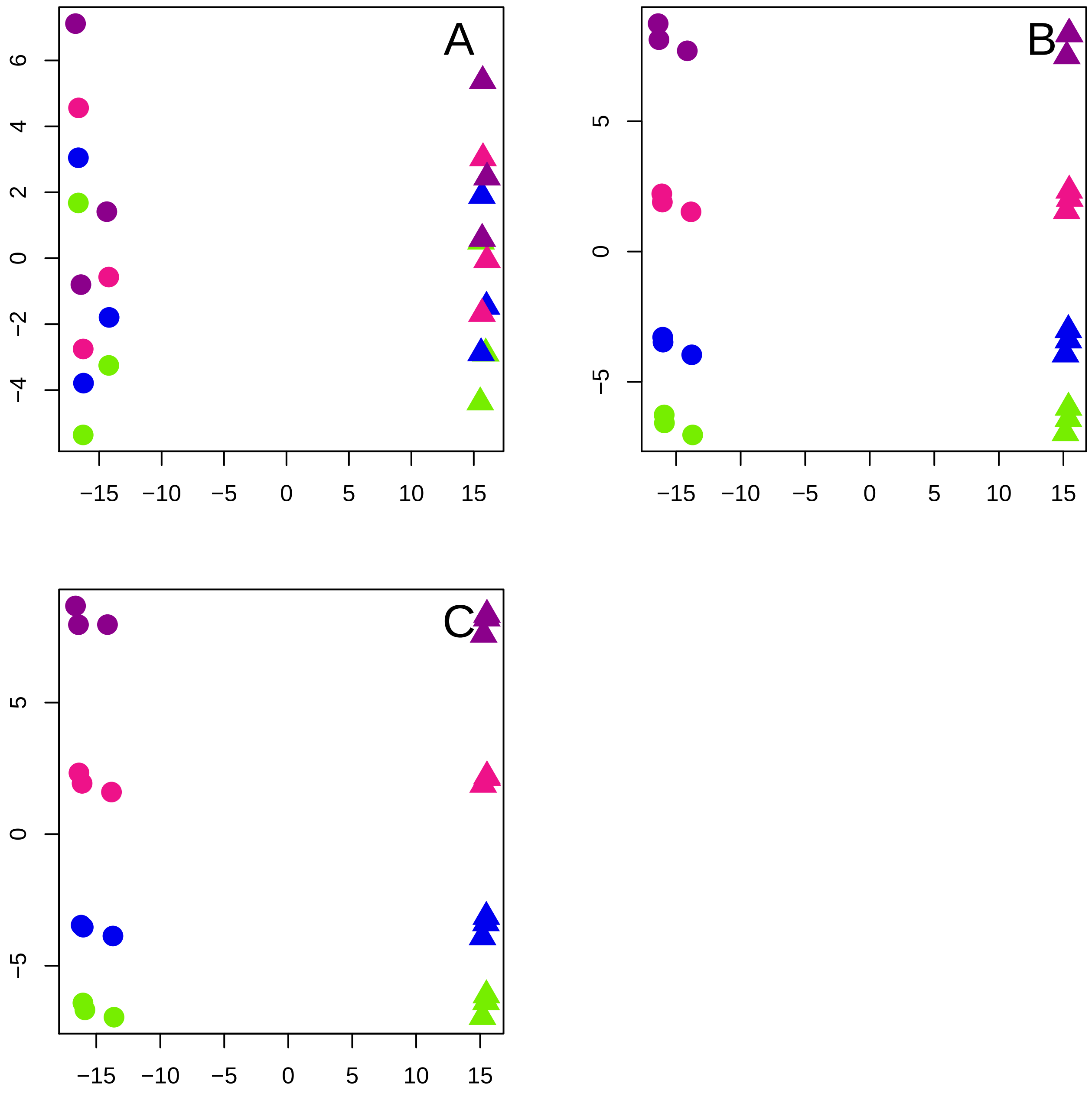
A
B
C
−15
−10
−5
0
5
10
15
6
4
2
0
−2
−4
5
0
−5

Additional File 12. Compendium of figures for data from human cells infected with *H. pylori* at two time points with results separated by read pairing status. A heatmap with hierarchical clustering with statistical support is shown on page 1 with the condition denoted according to letter code from Supplementary Table 2, followed by the replicate designation and the read length. A PCA plot is shown on page 2 where the conditions are denoted by the shape and the read length by the color (green, 36 bp; blue, 54 bp; magenta, 72 bp; purple, 101 bp). On both pages, results are shown in the three panels for (A) paired end reads, (B) first-in-pair single end reads, and (C) second-in-pair single reads.

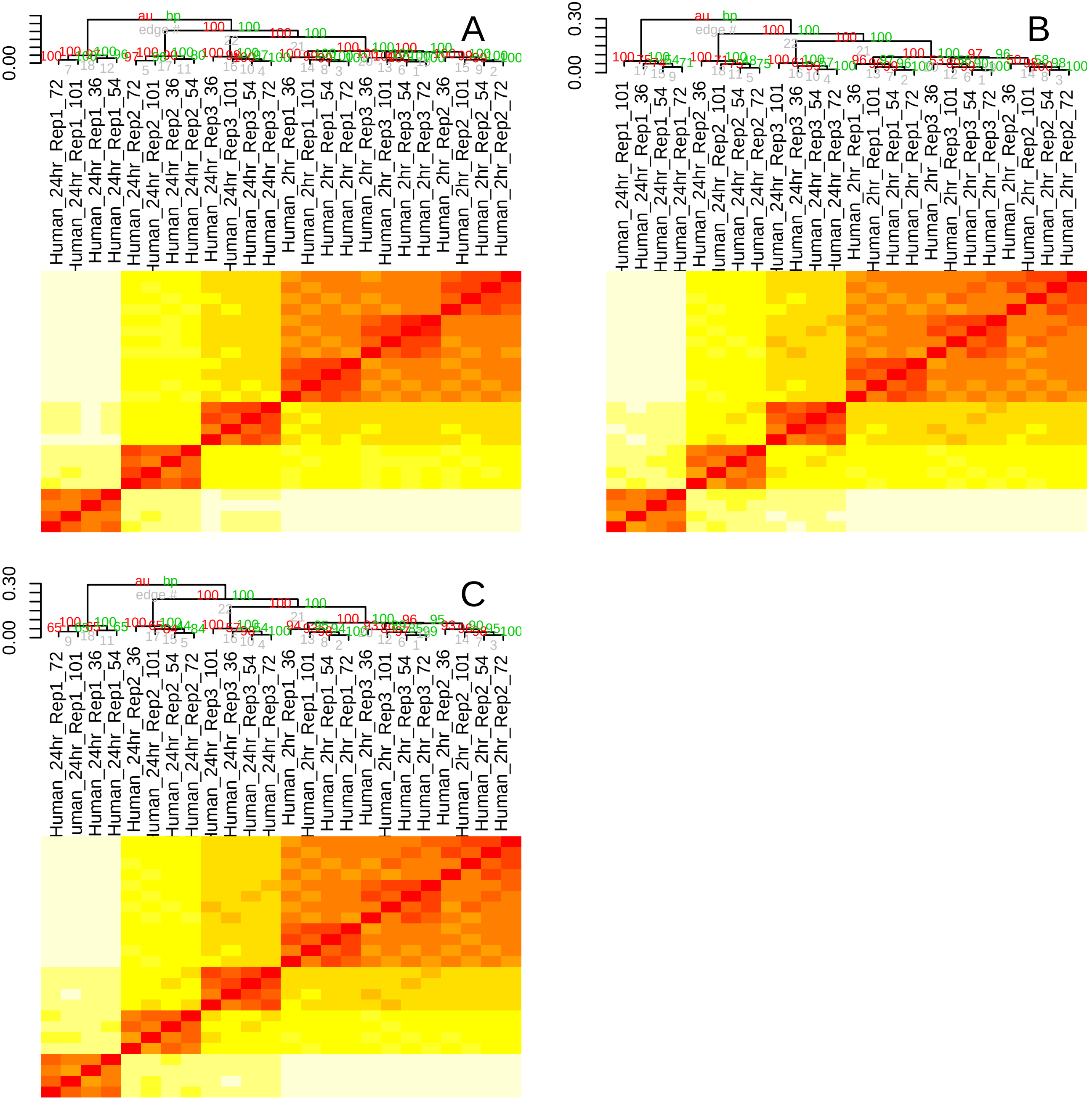
A
B
C
au
bp
edge #
0.00
0.30
Human_24hr_Rep1_72
Human_24hr_Rep1_101
Human_24hr_Rep1_36
Human_24hr_Rep1_54
Human_24hr_Rep2_72
Human_24hr_Rep2_101
Human_24hr_Rep2_36
Human_24hr_Rep2_54
Human_24hr_Rep3_36
Human_24hr_Rep3_101
Human_24hr_Rep3_54
Human_24hr_Rep3_72
Human_2hr_Rep1_36
Human_2hr_Rep1_101
Human_2hr_Rep1_54
Human_2hr_Rep1_72
Human_2hr_Rep3_36
Human_2hr_Rep3_101
Human_2hr_Rep3_54
Human_2hr_Rep3_72
Human_2hr_Rep2_36
Human_2hr_Rep2_101
Human_2hr_Rep2_54
Human_2hr_Rep2_72

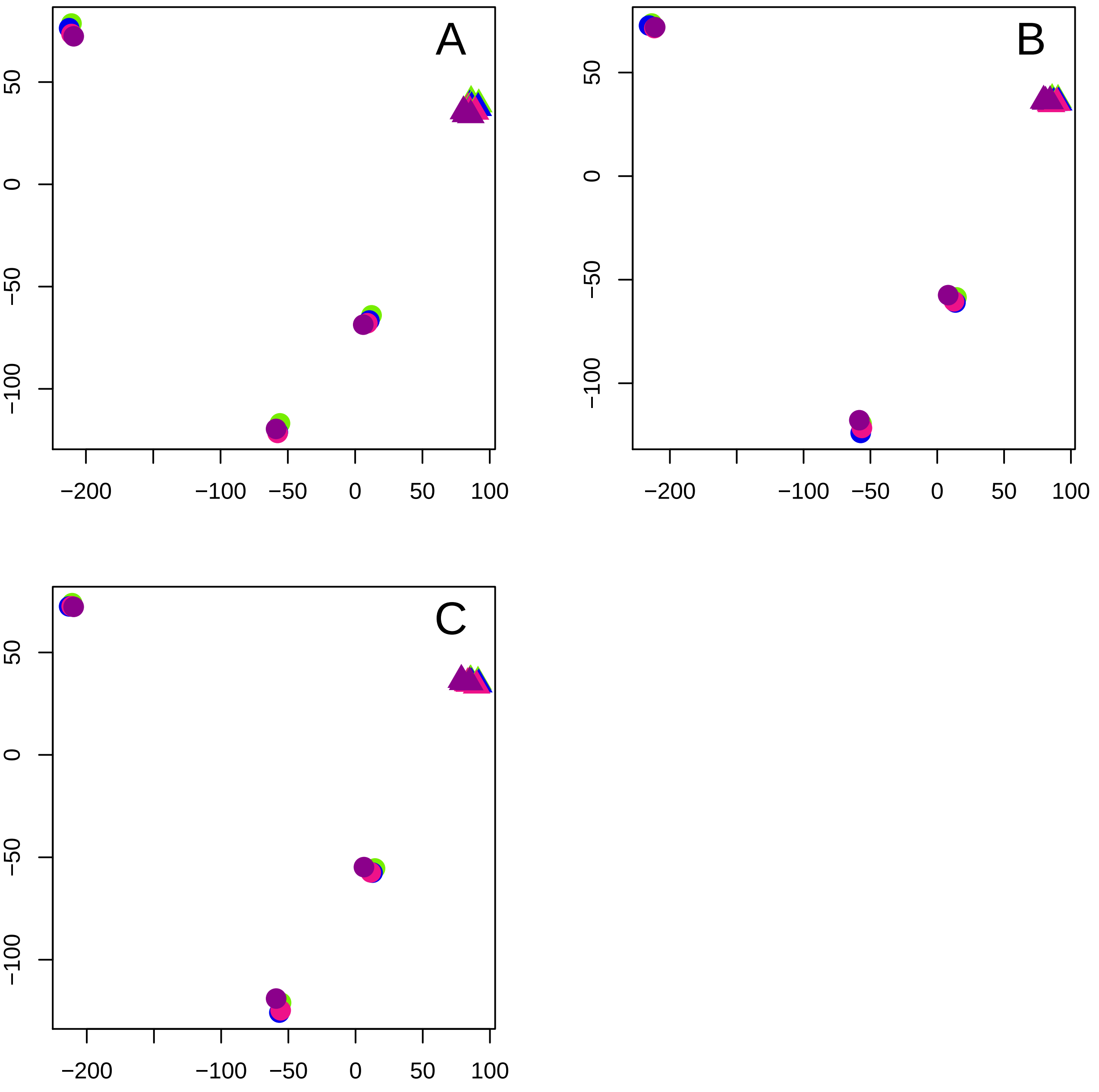
A
B
C
50
0
−50
−100
−200
−100
−50
0
50
100

Additional File 13. Compendium of figures for data from *P. aeruginosa* in stationary and exponential phase with results separated by read pairing status. A heatmap with hierarchical clustering with statistical support is shown on page 1 with the condition from Supplementary Table 2, followed by the replicate designation and the read length. A PCA plot is shown on page 2 where the conditions are denoted by the shape and the read length by the color (green, 36 bp; blue, 54 bp; magenta, 72 bp; purple, 101 bp). On both pages, results are shown in the three panels for (A) paired end reads, (B) first-in-pair single end reads, and (C) second-in-pair single reads.

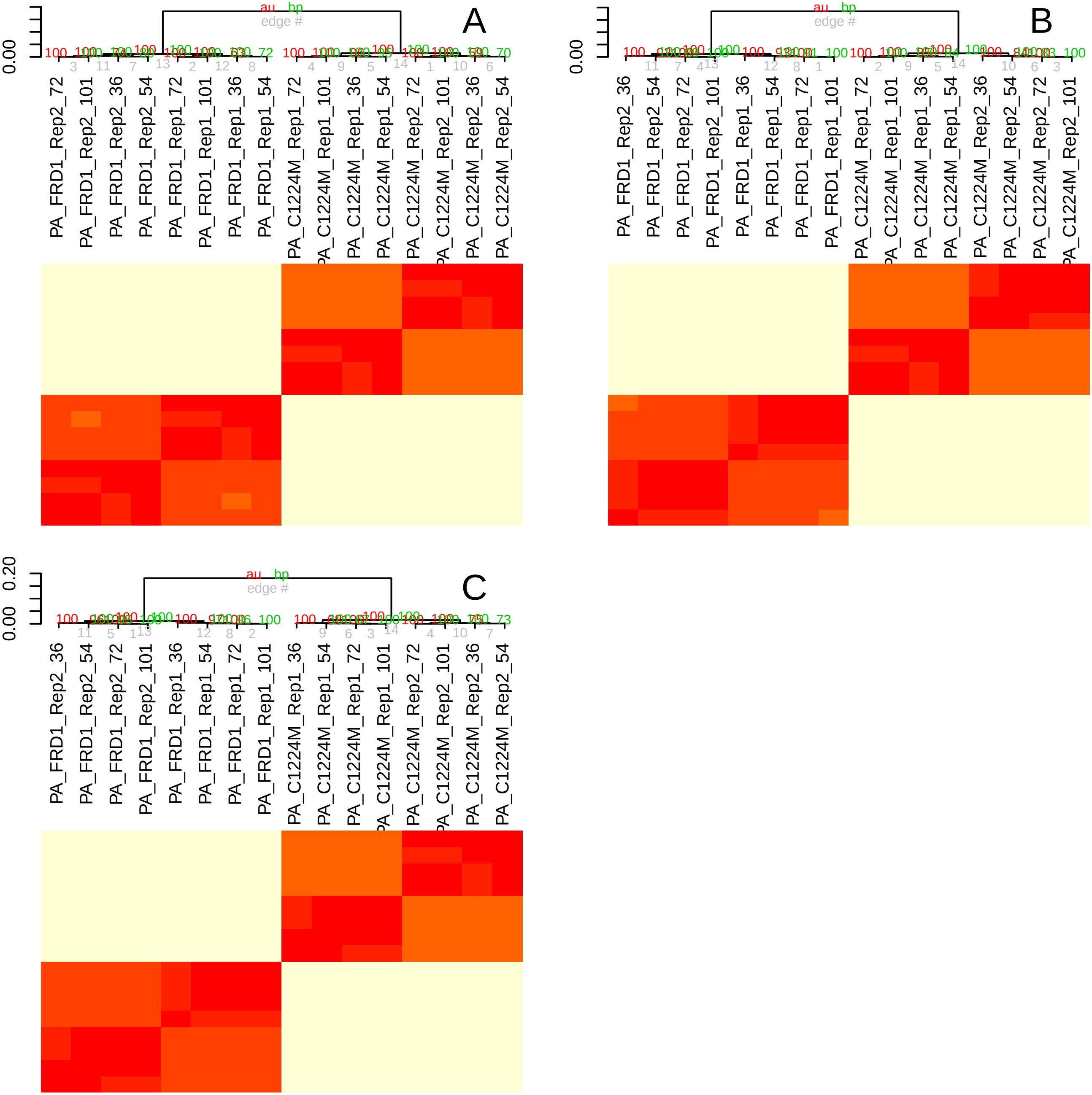
A
B
C
au
bp
edge #
0.00
0.20
PA_FRD1_Rep2_72
PA_FRD1_Rep2_101
PA_FRD1_Rep2_36
PA_FRD1_Rep2_54
PA_FRD1_Rep1_72
PA_FRD1_Rep1_101
PA_FRD1_Rep1_36
PA_FRD1_Rep1_54
PA_C1224M_Rep1_72
PA_C1224M_Rep1_101
PA_C1224M_Rep1_36
PA_C1224M_Rep1_54
PA_C1224M_Rep2_72
PA_C1224M_Rep2_101
PA_C1224M_Rep2_36
PA_C1224M_Rep2_54

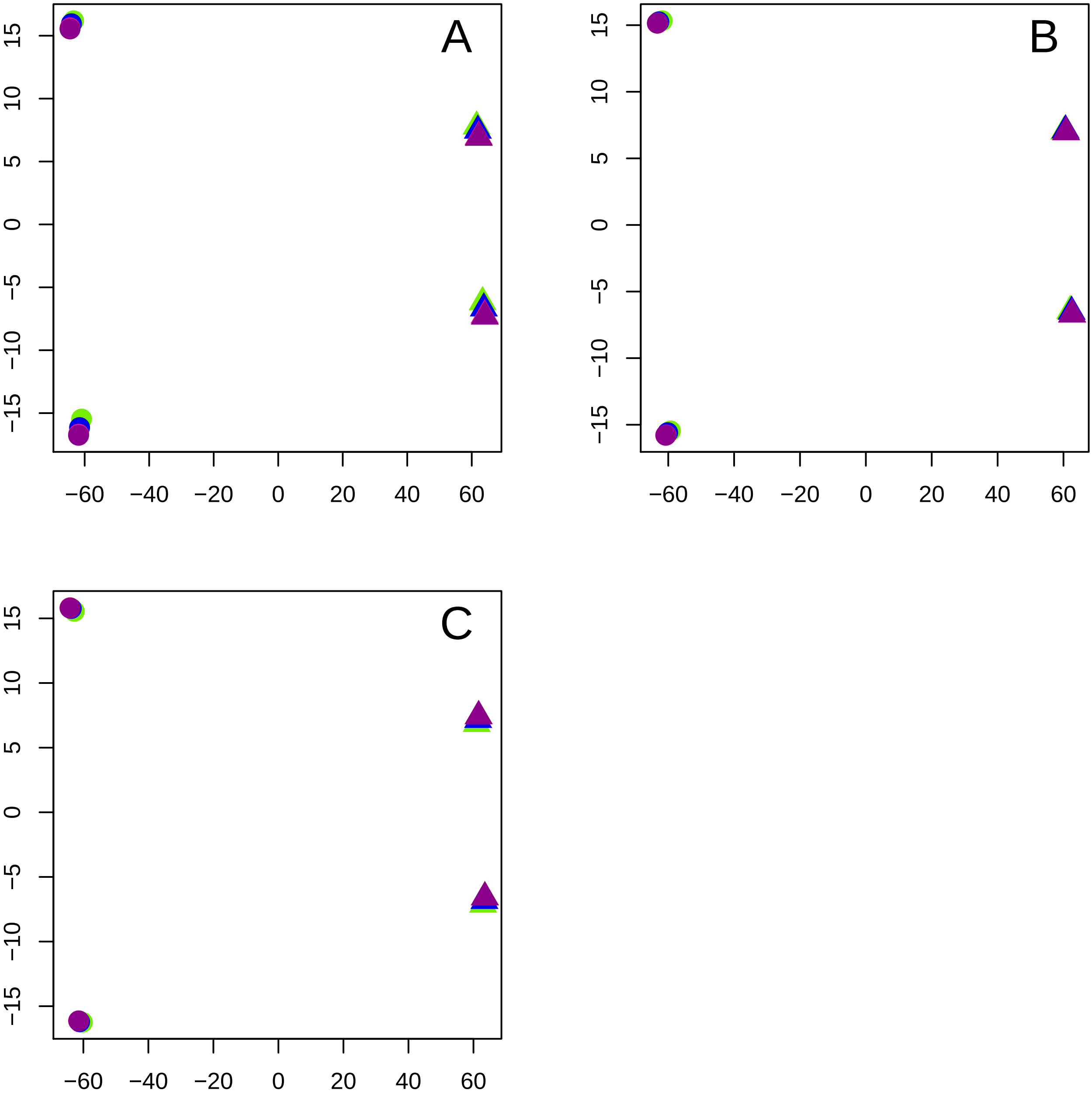
A
B
C
−60
−40
−20
0
20
40
60
−15
−10
−5
0
5
10
15

Additional File 14. Compendium of figures for data from two *P. aeruginosa* clinical isolates with results separated by read pairing status. A heatmap with hierarchical clustering with statistical support is shown on page 1 with the condition denoted from Supplementary Table 2, followed by the replicate designation and the read length. A PCA plot is shown on page 2 where the conditions are denoted by the shape and the read length by the color (green, 36 bp; blue, 54 bp; magenta, 72 bp; purple, 101 bp). On both pages, results are shown in the three panels for (A) paired end reads, (B) first-in-pair single end reads, and (C) second-in-pair single reads.

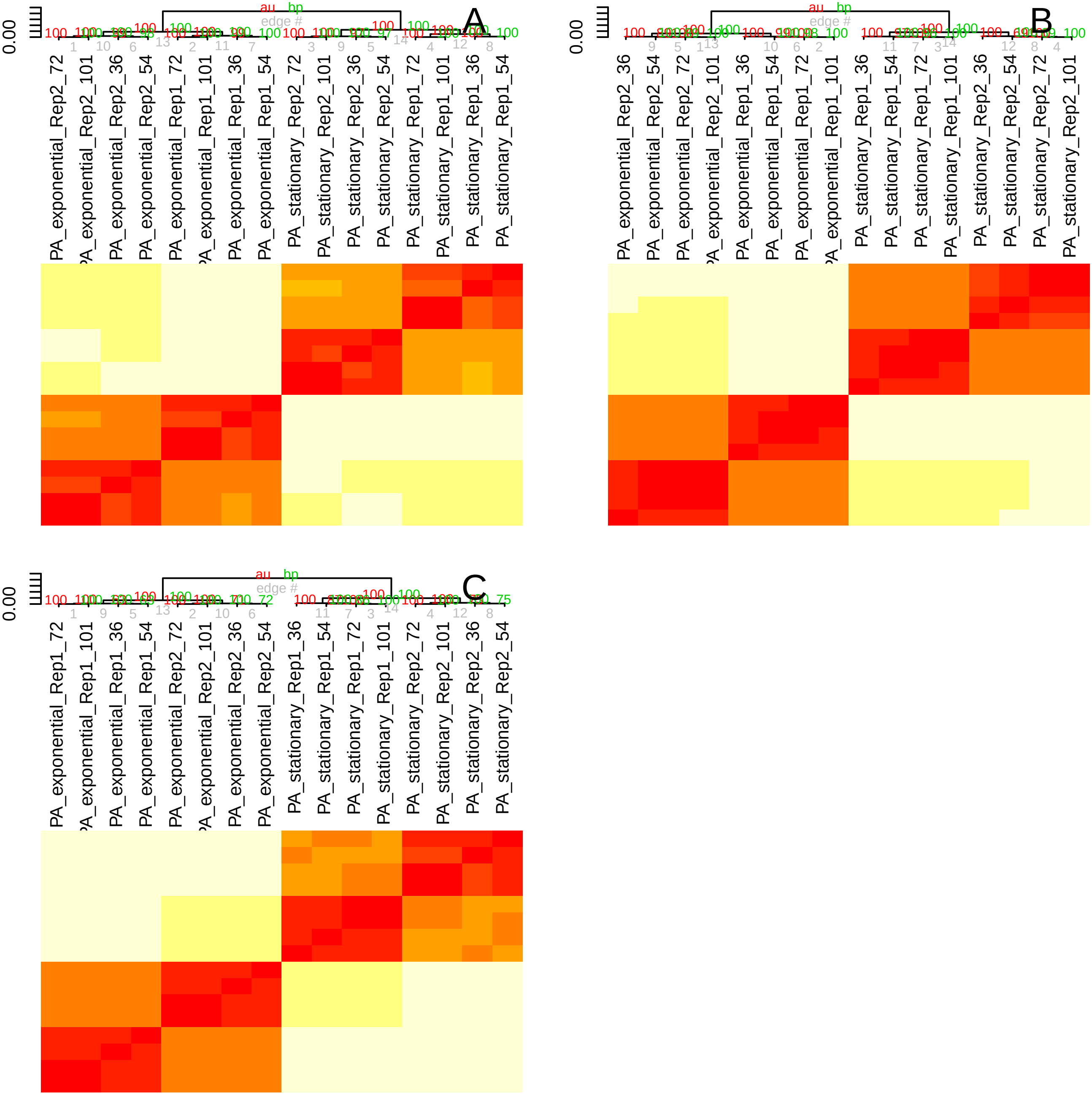
A
B
C
au
bp
edge #
0.00
PA_exponential_Rep2_72
PA_exponential_Rep2_101
PA_exponential_Rep2_36
PA_exponential_Rep2_54
PA_exponential_Rep1_72
PA_exponential_Rep1_101
PA_exponential_Rep1_36
PA_exponential_Rep1_54
PA_stationary_Rep2_72
PA_stationary_Rep2_101
PA_stationary_Rep2_36
PA_stationary_Rep2_54
PA_stationary_Rep1_72
PA_stationary_Rep1_101
PA_stationary_Rep1_36
PA_stationary_Rep1_54

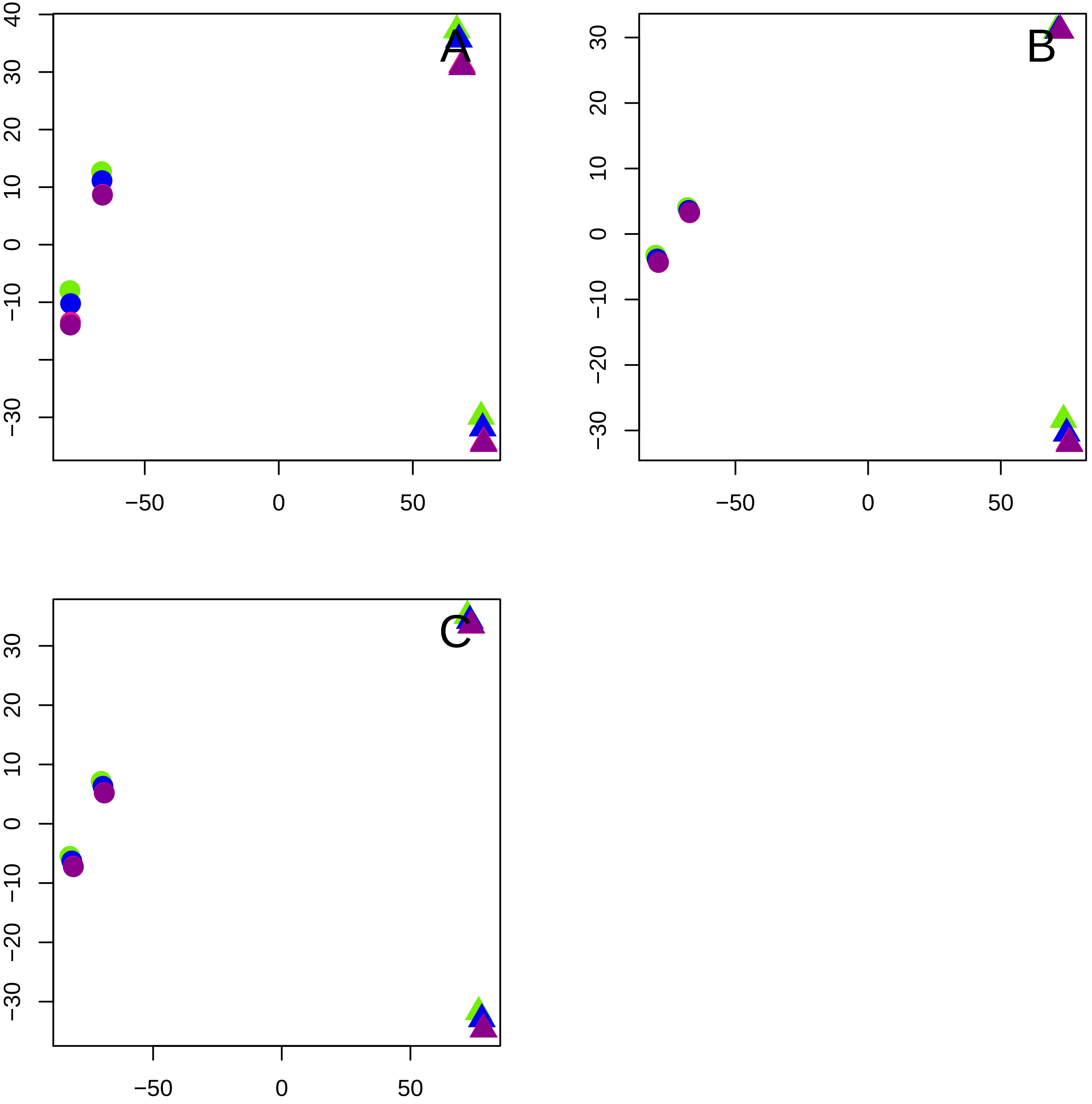
A
B
C
−50
0
50
40
30
20
10
0
−10
−20
−30

Additional File 15. Compendium of figures for Encode CSHL comparisons of IMR-90 v. NHD cells with results separated by read length. A heatmap with hierarchical clustering with statistical support is shown on page 1 with the condition denoted according to letter code from Supplementary Table 2, followed by the replicate designation and the pairing status such that (0) paired reads, (1) first-in-read single end read, and (2) second-in-read single end read. A PCA plot is shown on page 2 where the conditions are denoted by the shape (circle, IMR-90; triangle, NHD) and the pairing status by the color (green, paired end; blue, first-in-pair single end read; magenta, second-in-pair single end read). On both pages, results are shown in the four panels: (A) 36-bp reads, (B) 54-bp reads, (C) 72-bp reads, and (D) 101-bp reads.

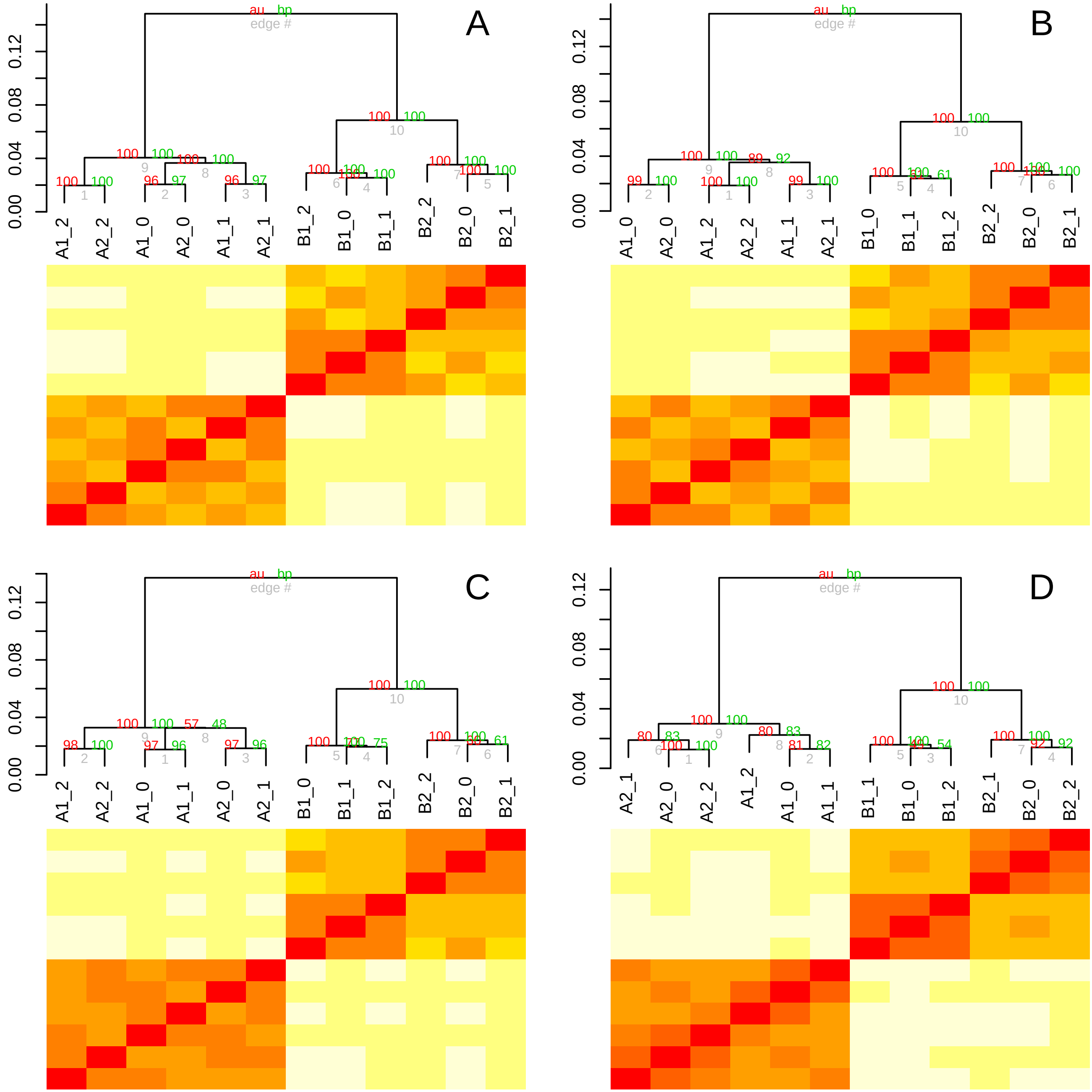
A
B
C
D
au
bp
edge #
0.00
0.04
0.08
0.12
A1_2 A2_2 A1_0 A2_0 A1_1 A2_1 B1_2 B1_0 B1_1 B2_2 B2_0 B2_1
A1_0 A2_0 A1_2 A2_2 A1_1 A2_1 B1_0 B1_1 B1_2 B2_2 B2_0 B2_1
A1_2 A2_2 A1_0 A1_1 A2_0 A2_1 B1_0 B1_1 B1_2 B2_2 B2_0 B2_1
A2_1 A2_0 A2_2 A1_2 A1_0 A1_1 B1_1 B1_0 B1_2 B2_1 B2_0 B2_2

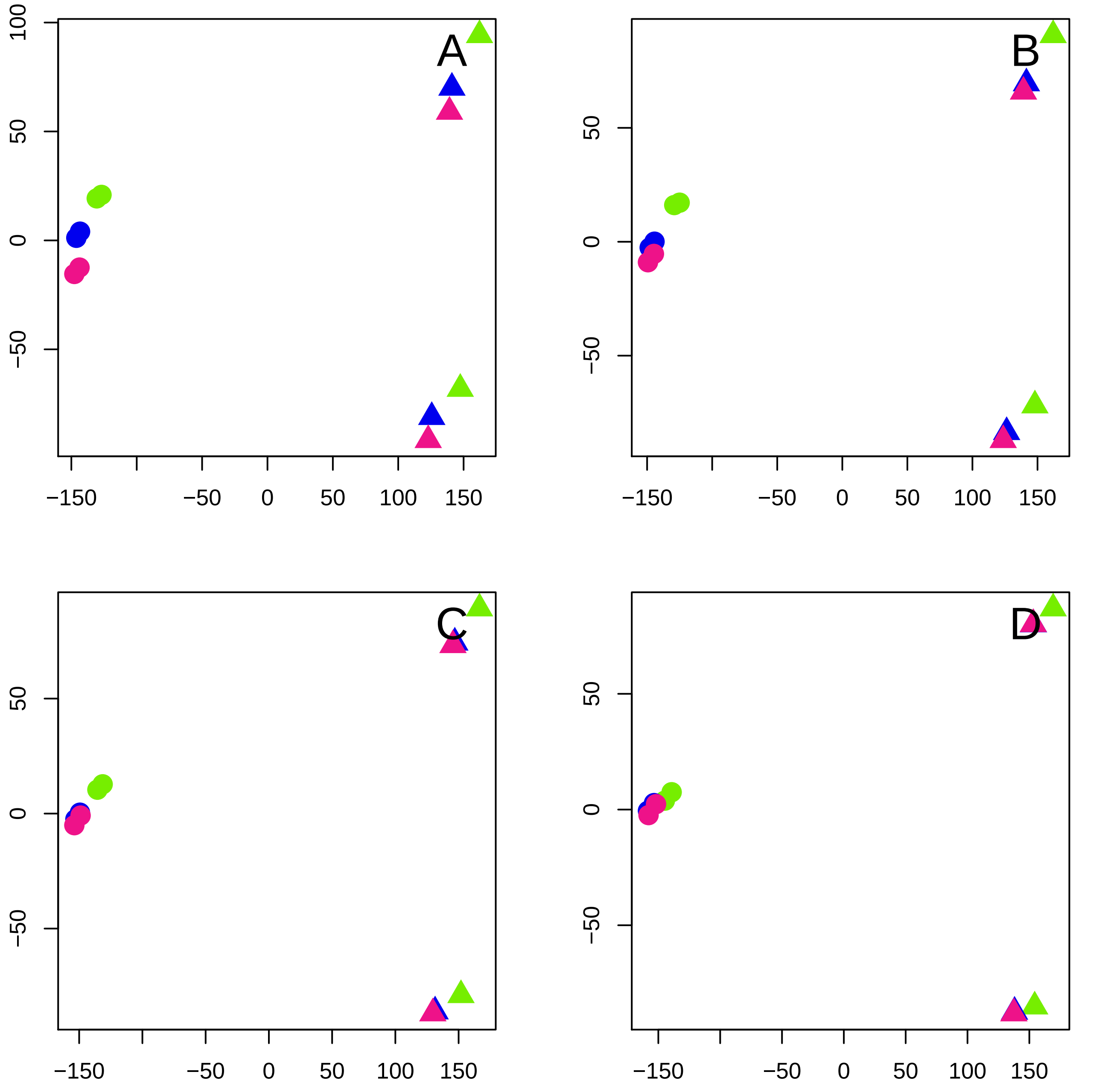
A
B
C
D
100
50
0
−50
−150
−50
0
50
100
150

Additional File 16. Compendium of figures for data from *Candida*-infected mouse vaginas with results separated by read length. A heatmap with hierarchical clustering with statistical support is shown on page 1 with the condition denoted according to letter code from Supplementary Table 2, followed by the replicate designation and the pairing status such that (0) paired reads, (1) first-in-read single end read, and (2) second-in-read single end read. A PCA plot is shown on page 2 where the conditions are denoted by the shape (circle, CA_d3; triangle, naïve_d3) and the pairing status by the color (green, paired end; blue, first-in-pair single end read; magenta, second-in-pair single end read). On both pages, results are shown in the four panels: (A) 36-bp reads, (B) 54-bp reads, (C) 72-bp reads, and (D) 101-bp reads.

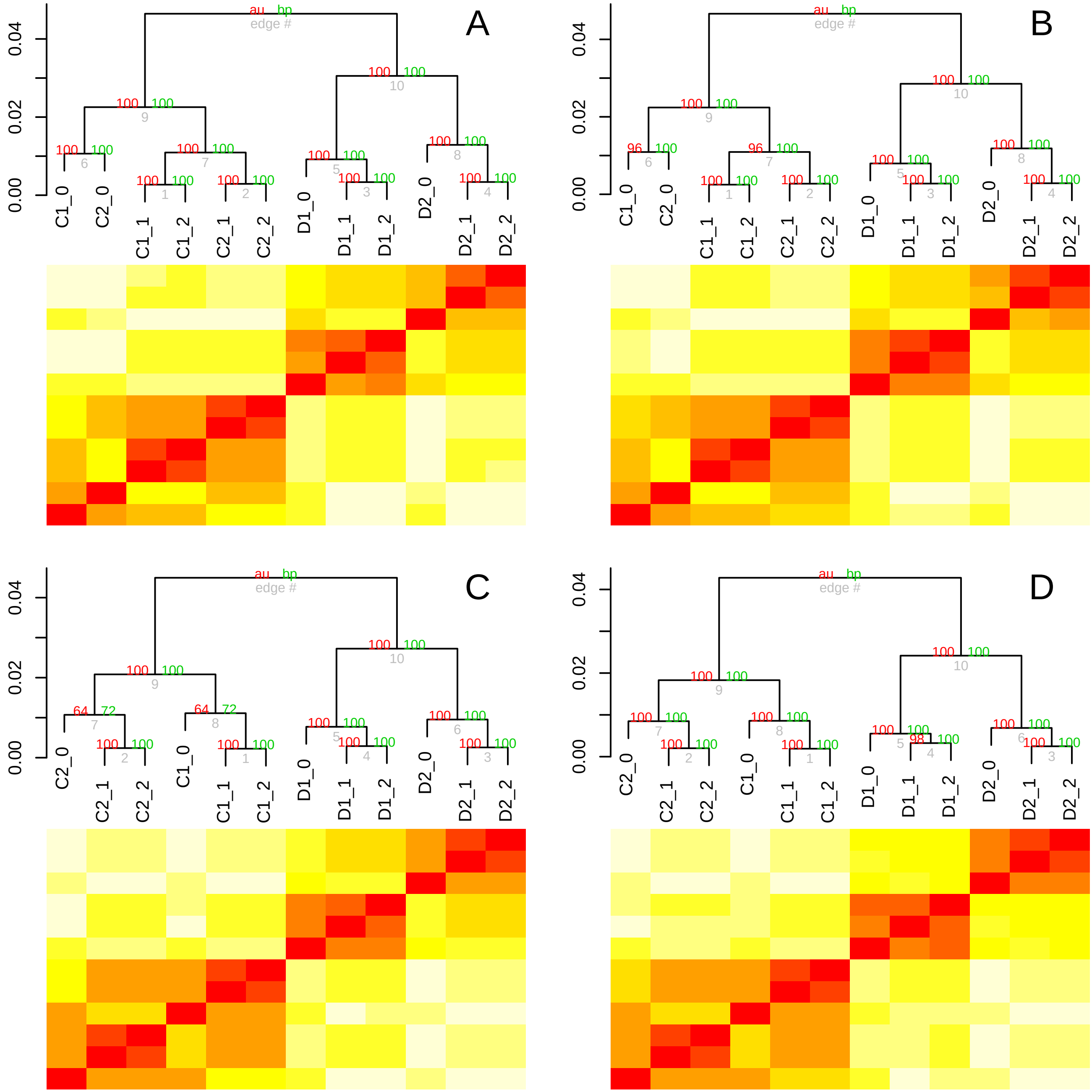

A
B
C
D
au
bp
edge #
0.00
0.02
0.04
C1_0
C2_0
C1_1
C1_2
C2_1
C2_2
D1_0
D1_1
D1_2
D2_0
D2_1
D2_2

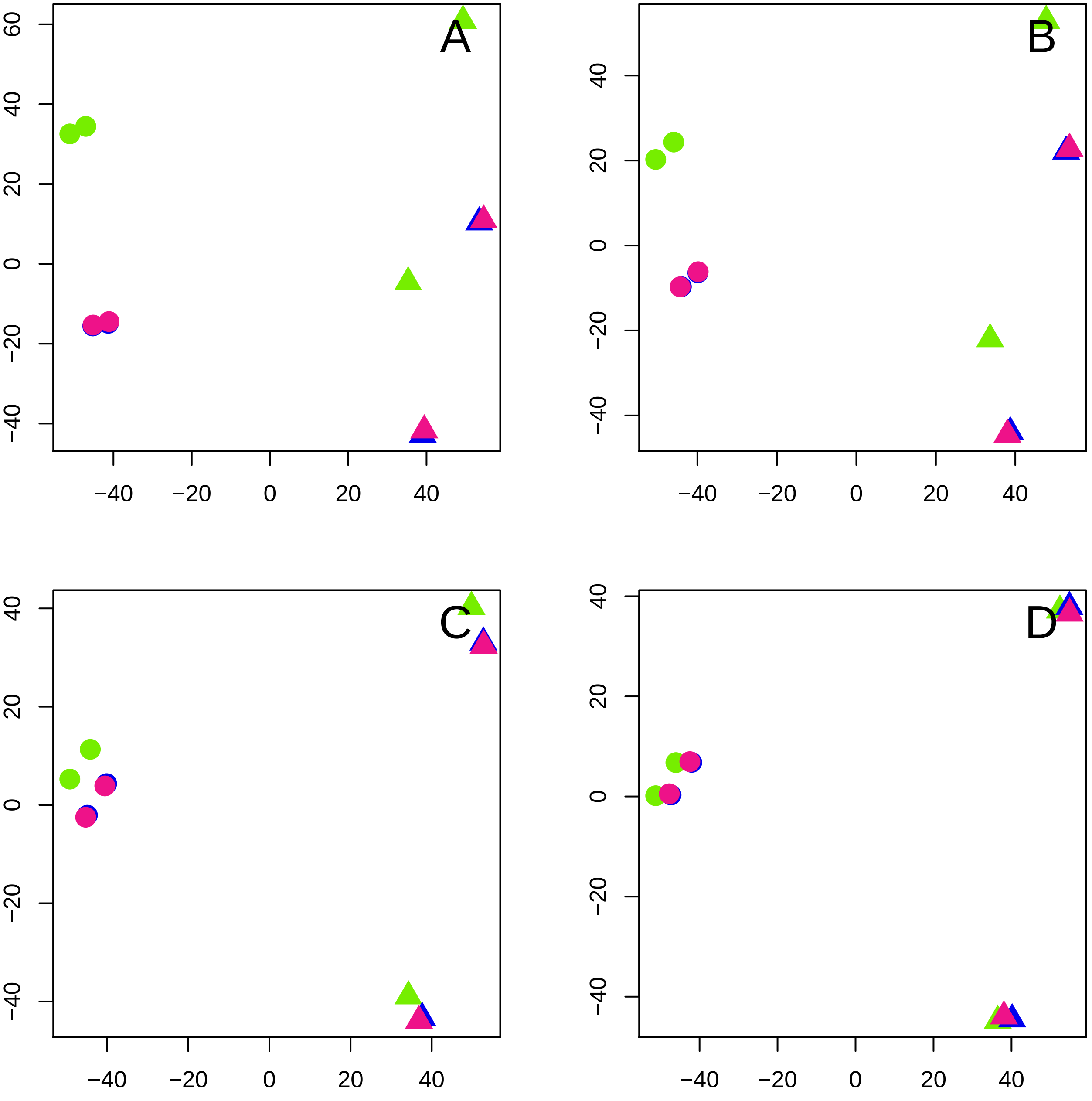
A
B
C
D
60
40
20
0
−20
−40
−40
−20
0
20
40

Additional File 17. Compendium of figures for *A. fumigatus* data with results separated by read length. A heatmap with hierarchical clustering with statistical support is shown on page 1 with the condition denoted according to letter code from Supplementary Table 2, followed by the replicate designation and the pairing status such that (0) paired reads, (1) first-in-read single end read, and (2) second-in-read single end read. A PCA plot is shown on page 2 where the conditions are denoted by the shape (circle, 1_6h_AF293; triangle, 4_6h_AF293) and the pairing status by the color (green, paired end; blue, first-in-pair single end read; magenta, second-in-pair single end read). On both pages, results are shown in the four panels: (A) 36-bp reads, (B) 54-bp reads, (C) 72-bp reads, and (D) 101-bp reads.

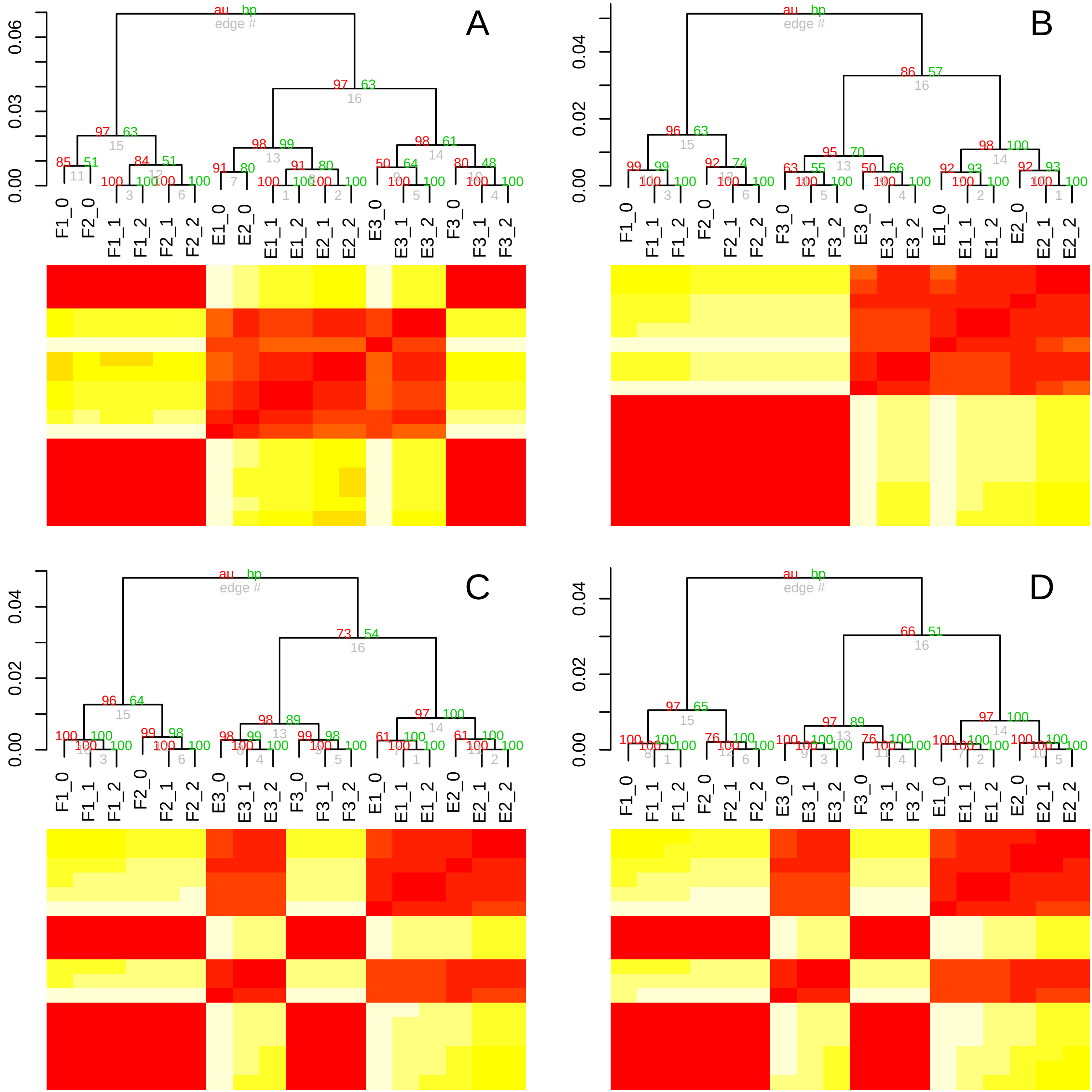
A
B
C
D
au
bp
edge #
F1_0
F2_0
F1_1
F1_2
F2_1
F2_2
E1_0
E2_0
E1_1
E1_2
E2_1
E2_2
E3_0
E3_1
E3_2
F3_0
F3_1
F3_2

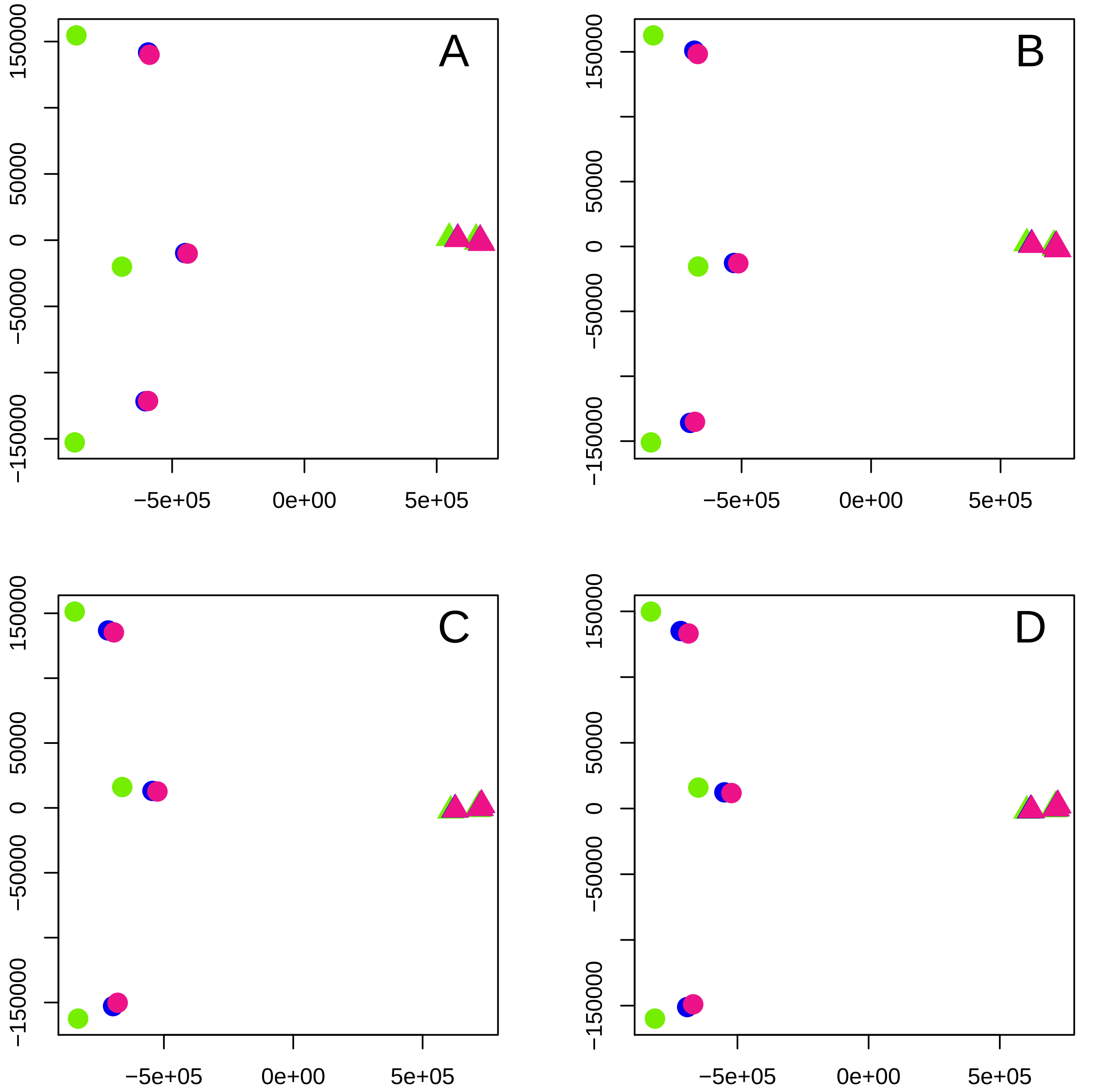
A
B
C
D
150000
50000
0
−50000
−150000
−5e+05
0e+00
5e+05

Additional File 18. Compendium of figures for *Candida*-human data with results separated by read length. A heatmap with hierarchical clustering with statistical support is shown on page 1 with the condition denoted according to letter code from Supplementary Table 2, followed by the replicate designation and the pairing status such that (0) paired reads, (1) first-in-read single end read, and (2) second-in-read single end read. A PCA plot is shown on page 2 where the conditions are denoted by the shape (circle, 5h_c; triangle, 5h_oc) and the pairing status by the color (green, paired end; blue, first-in-pair single end read; magenta, second-in-pair single end read). On both pages, results are shown in the four panels: (A) 36-bp reads, (B) 54-bp reads, (C) 72-bp reads, and (D) 101-bp reads.

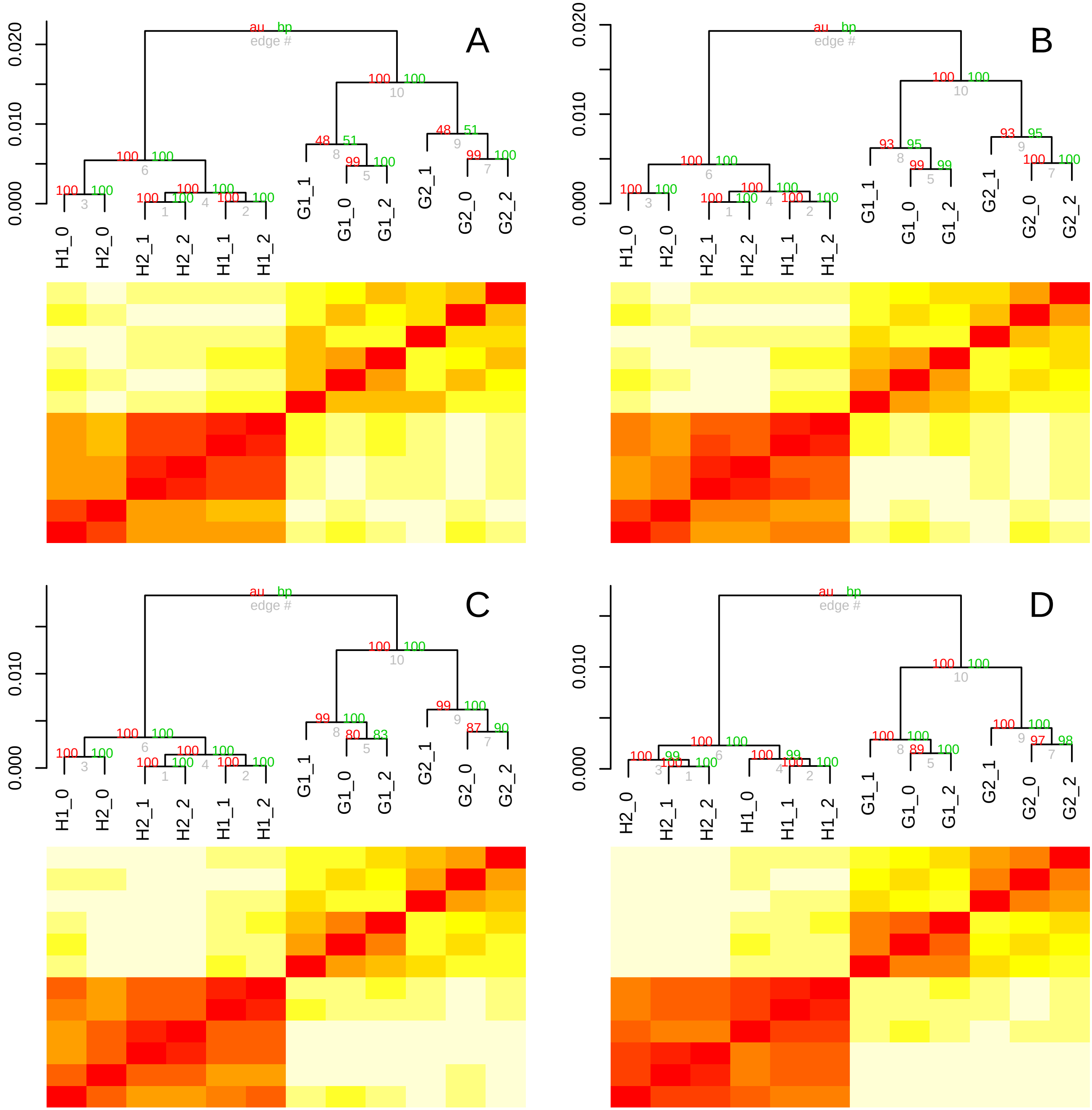

A
B
C
D
au bp
edge #
H1_0
H2_0
H2_1
H2_2
H1_1
H1_2
G1_1
G1_0
G1_2
G2_1
G2_0
G2_2

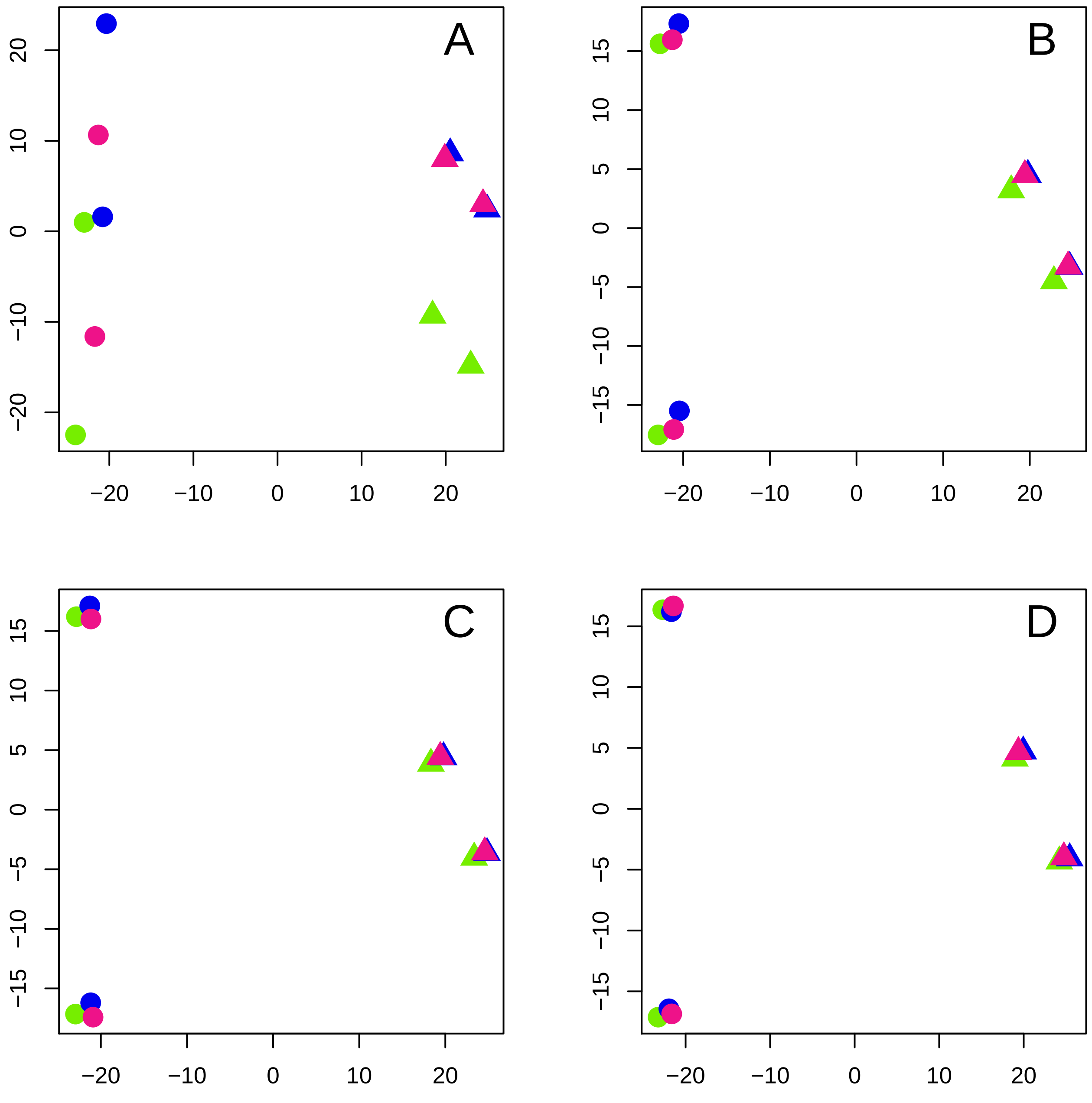
A
B
C
D
−20
−10
0
10
20
−15
−5
5
15

Additional File 19. Compendium of figures for *Candida*-only data with results separated by read length. A heatmap with hierarchical clustering with statistical support is shown on page 1 with the condition denoted according to letter code from Supplementary Table 2, followed by the replicate designation and the pairing status such that (0) paired reads, (1) first-in-read single end read, and (2) second-in-read single end read. A PCA plot is shown on page 2 where the conditions are denoted by the shape (circle, rh2_comp; triangle, rh2_del) and the pairing status by the color (green, paired end; blue, first-in-pair single end read; magenta, second-in-pair single end read). On both pages, results are shown in the four panels: (A) 36-bp reads, (B) 54-bp reads, (C) 72-bp reads, and (D) 101-bp reads.

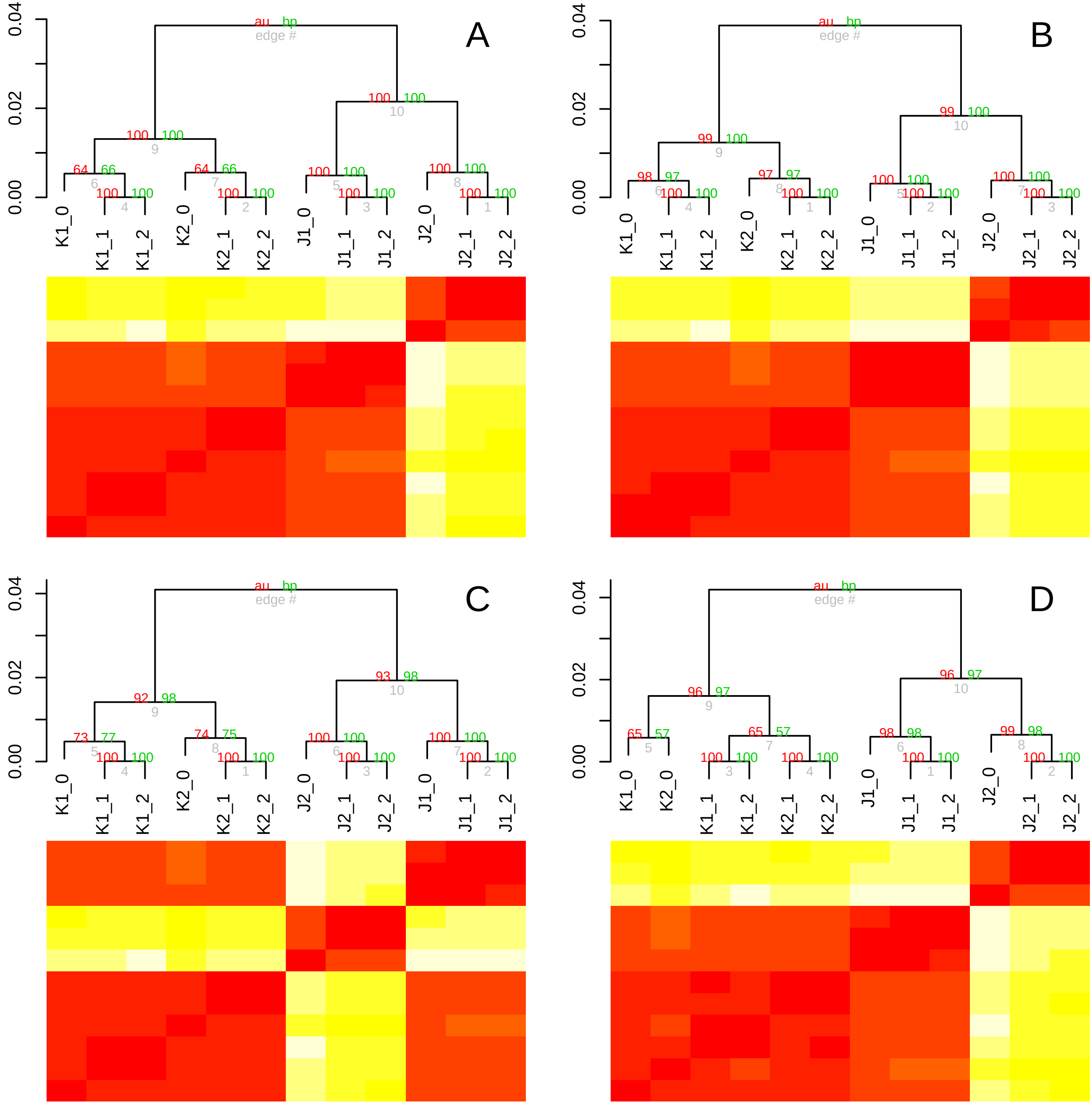
A
au bp
edge #
0.00
0.02
0.04
K1_0
K1_1
K1_2
K2_0
K2_1
K2_2
J1_0
J1_1
J1_2
J2_0
J2_1
J2_2
B
au bp
edge #
0.00
0.02
0.04
K1_0
K1_1
K1_2
K2_0
K2_1
K2_2
J1_0
J1_1
J1_2
J2_0
J2_1
J2_2
C
au bp
edge #
0.00
0.02
0.04
K1_0
K1_1
K1_2
K2_0
K2_1
K2_2
J2_0
J2_1
J2_2
J1_0
J1_1
J1_2
D
au bp
edge #
0.00
0.02
0.04
K1_0
K2_0
K1_1
K1_2
K2_1
K2_2
J1_0
J1_1
J1_2
J2_0
J2_1
J2_2

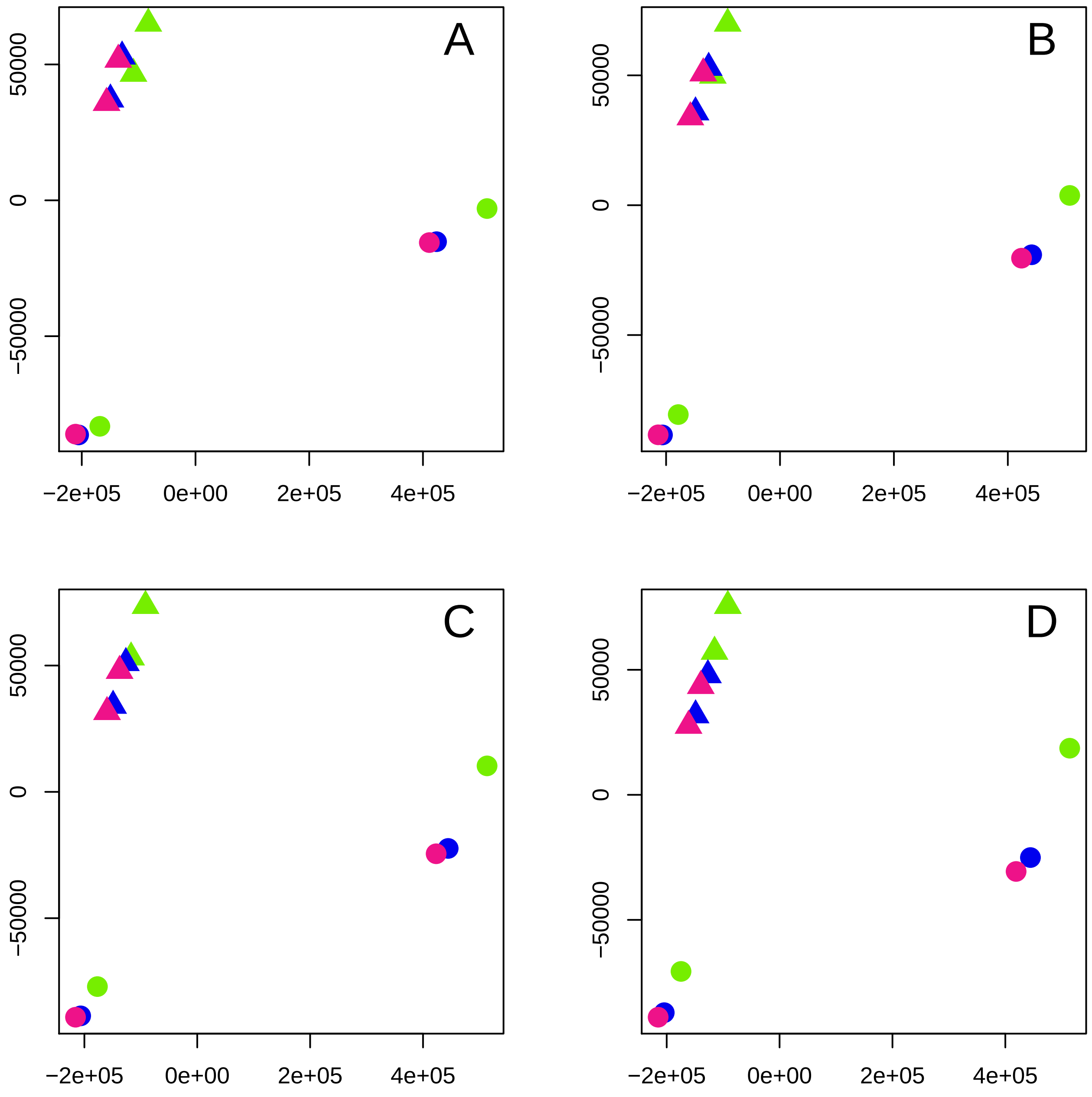
A
B
C
D
50000
0
−50000
−2e+05
0e+00
2e+05
4e+05

Additional File 20. Compendium of figures for *Es. coli* data with results separated by read length. A heatmap with hierarchical clustering with statistical support is shown on page 1 with the condition denoted according to letter code from Supplementary Table 2, followed by the replicate designation and the pairing status such that (0) paired reads, (1) first-in-read single end read, and (2) second-in-read single end read. A PCA plot is shown on page 2 where the conditions are denoted by the shape (circle, DMEM; triangle, LB) and the pairing status by the color (green, paired end; blue, first-in-pair single end read; magenta, second-in-pair single end read). On both pages, results are shown in the four panels: (A) 36-bp reads, (B) 54-bp reads, (C) 72-bp reads, and (D) 101-bp reads.

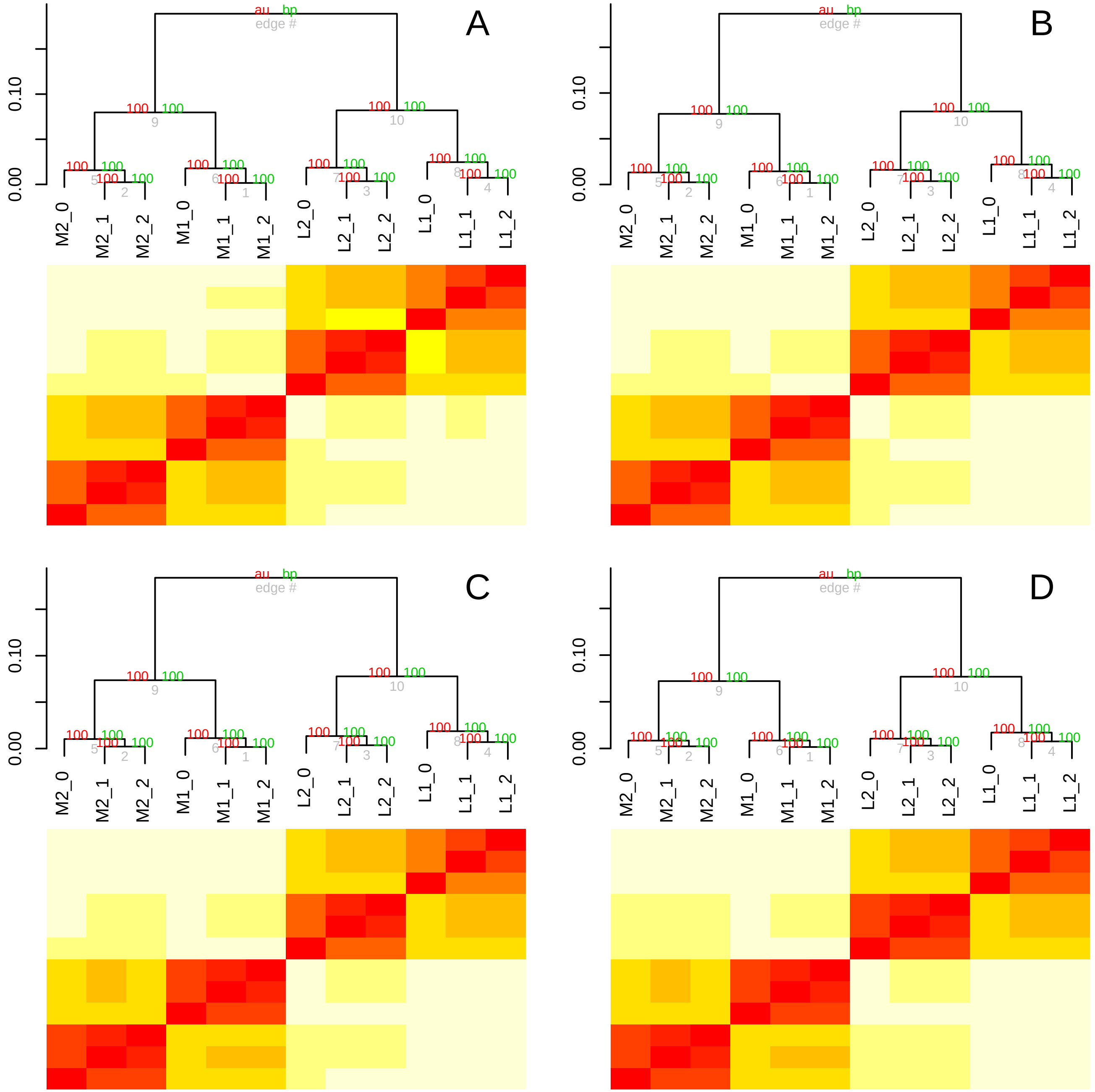
A
B
C
D
au
bp
edge #
0.00
0.10
M2_0
M2_1
M2_2
M1_0
M1_1
M1_2
L2_0
L2_1
L2_2
L1_0
L1_1
L1_2

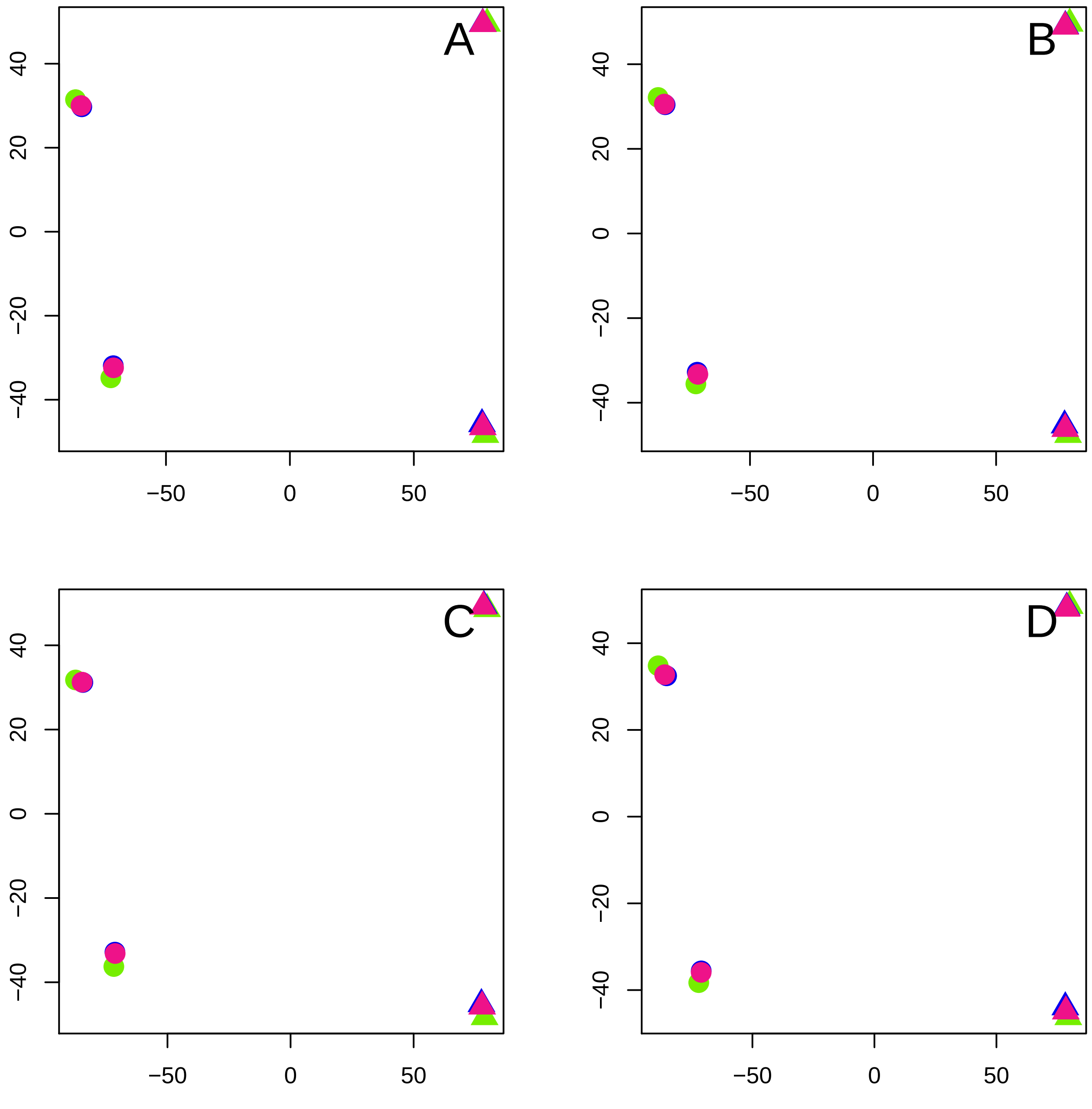
A
B
C
D
−50
0
50
40
20
0
−20
−40

Additional File 21. Compendium of figures for data from *Ixodes scapularis* cell lines with differential presence of *Eh. chaffeensis* strains with results separated by read length. A heatmap with hierarchical clustering with statistical support is shown on page 1 with the condition denoted according to letter code from Supplementary Table 2, followed by the replicate designation and the pairing status such that (0) paired reads, (1) first-in-read single end read, and (2) second-in-read single end read. A PCA plot is shown on page 2 where the conditions are denoted by the shape and the pairing status by the color (green, paired end; blue, first-in-pair single end read; magenta, second-in-pair single end read). On both pages, results are shown in the four panels: (A) 36-bp reads, (B) 54-bp reads, (C) 72-bp reads, and (D) 101-bp reads.

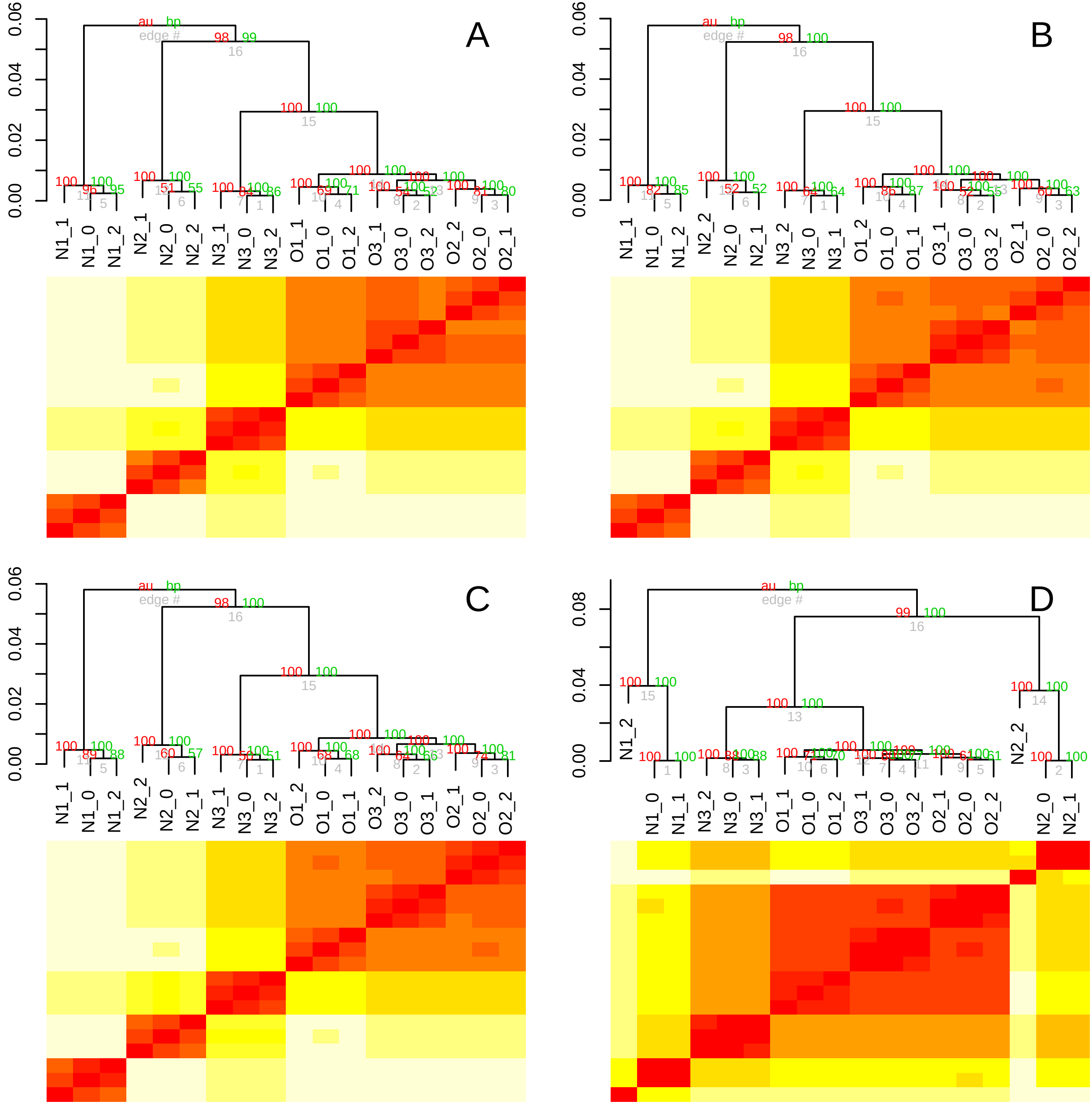
A
B
C
D
au
bp
edge #
0.00
0.02
0.04
0.06
0.08
N1_0
N1_1
N1_2
N2_0
N2_1
N2_2
N3_0
N3_1
N3_2
O1_0
O1_1
O1_2
O2_0
O2_1
O2_2
O3_0
O3_1
O3_2

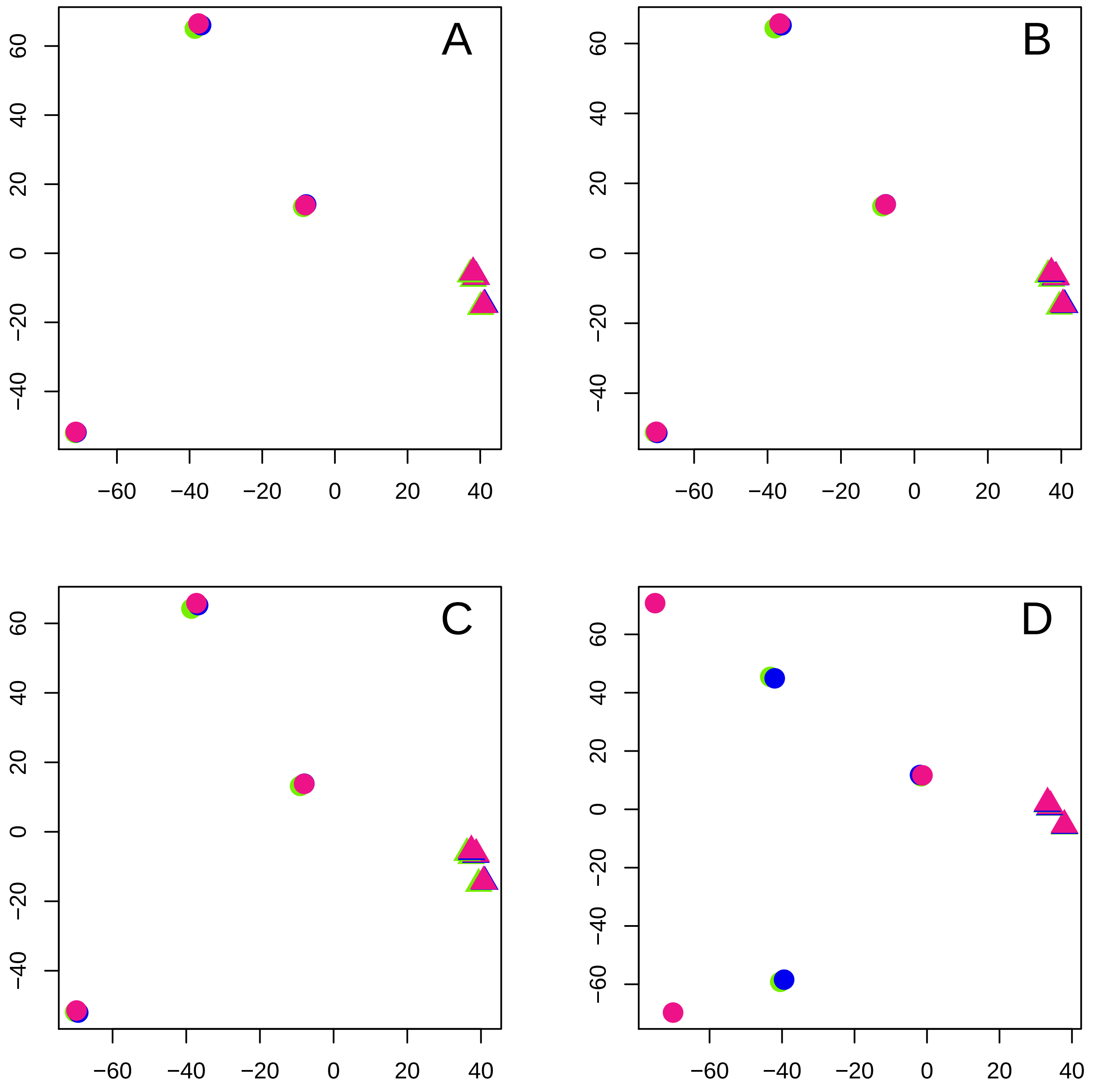
A
B
C
D
60
40
20
0
−20
−40
−60
−60
−40
−20
0
20
40

Additional File 22. Compendium of figures for data from canine cell lines with differential presence of *Eh. chaffeensis* strains with results separated by read length. A heatmap with hierarchical clustering with statistical support is shown on page 1 with the condition denoted according to letter code from Supplementary Table 2, followed by the replicate designation and the pairing status such that (0) paired reads, (1) first-in-read single end read, and (2) second-in-read single end read. A PCA plot is shown on page 2 where the conditions are denoted by the shape and the pairing status by the color (green, paired end; blue, first-in-pair single end read; magenta, second-in-pair single end read). On both pages, results are shown in the four panels: (A) 36-bp reads, (B) 54-bp reads, (C) 72-bp reads, and (D) 101-bp reads.

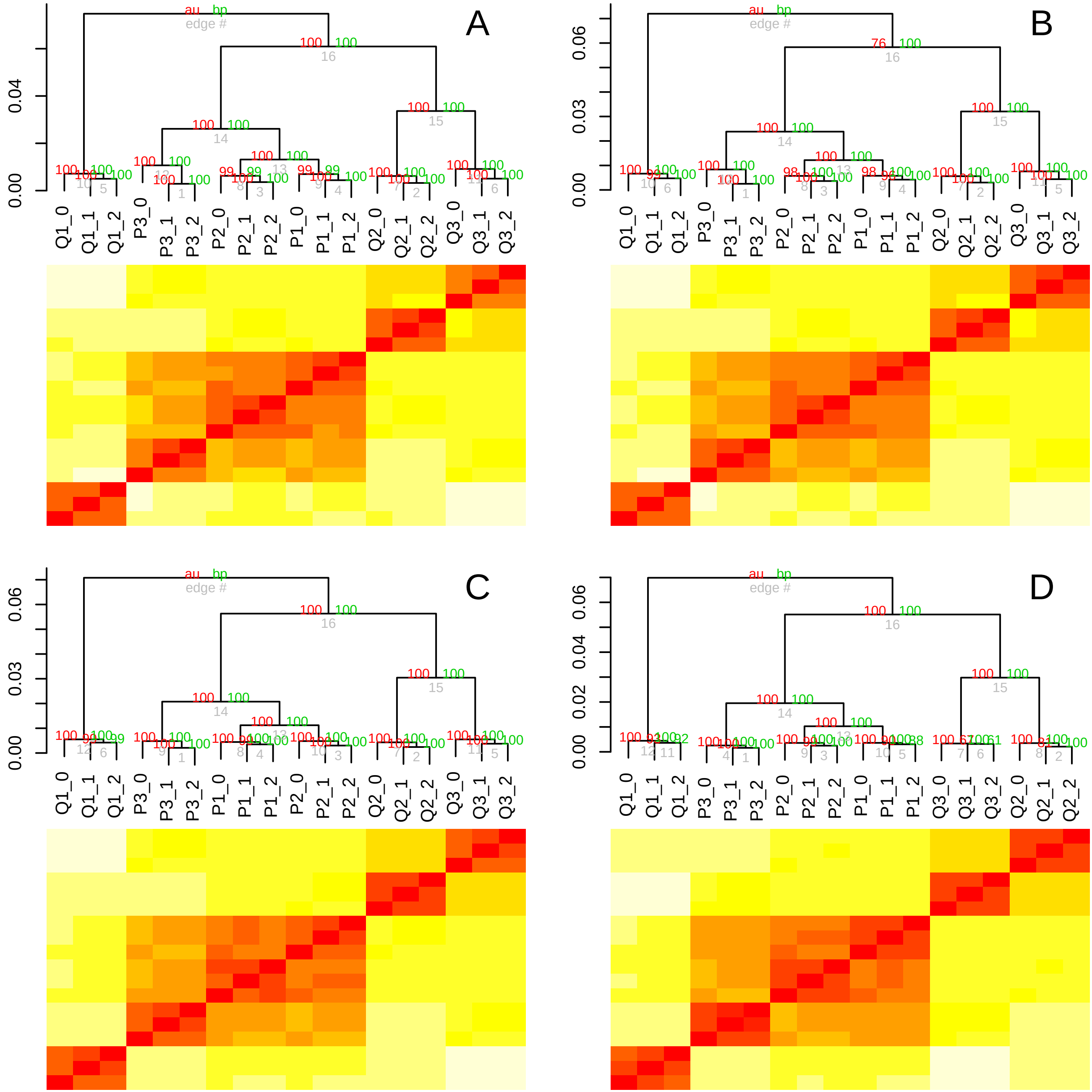
A
B
C
D
au bp
edge #
0.00
0.04
0.03
0.06
0.02
Q1_0 Q1_1 Q1_2 P3_0 P3_1 P3_2 P2_0 P2_1 P2_2 P1_0 P1_1 P1_2 Q2_0 Q2_1 Q2_2 Q3_0 Q3_1 Q3_2
Q1_0 Q1_1 Q1_2 P3_0 P3_1 P3_2 P1_0 P1_1 P1_2 P2_0 P2_1 P2_2 Q2_0 Q2_1 Q2_2 Q3_0 Q3_1 Q3_2
Q1_0 Q1_1 Q1_2 P3_0 P3_1 P3_2 P2_0 P2_1 P2_2 P1_0 P1_1 P1_2 Q3_0 Q3_1 Q3_2 Q2_0 Q2_1 Q2_2

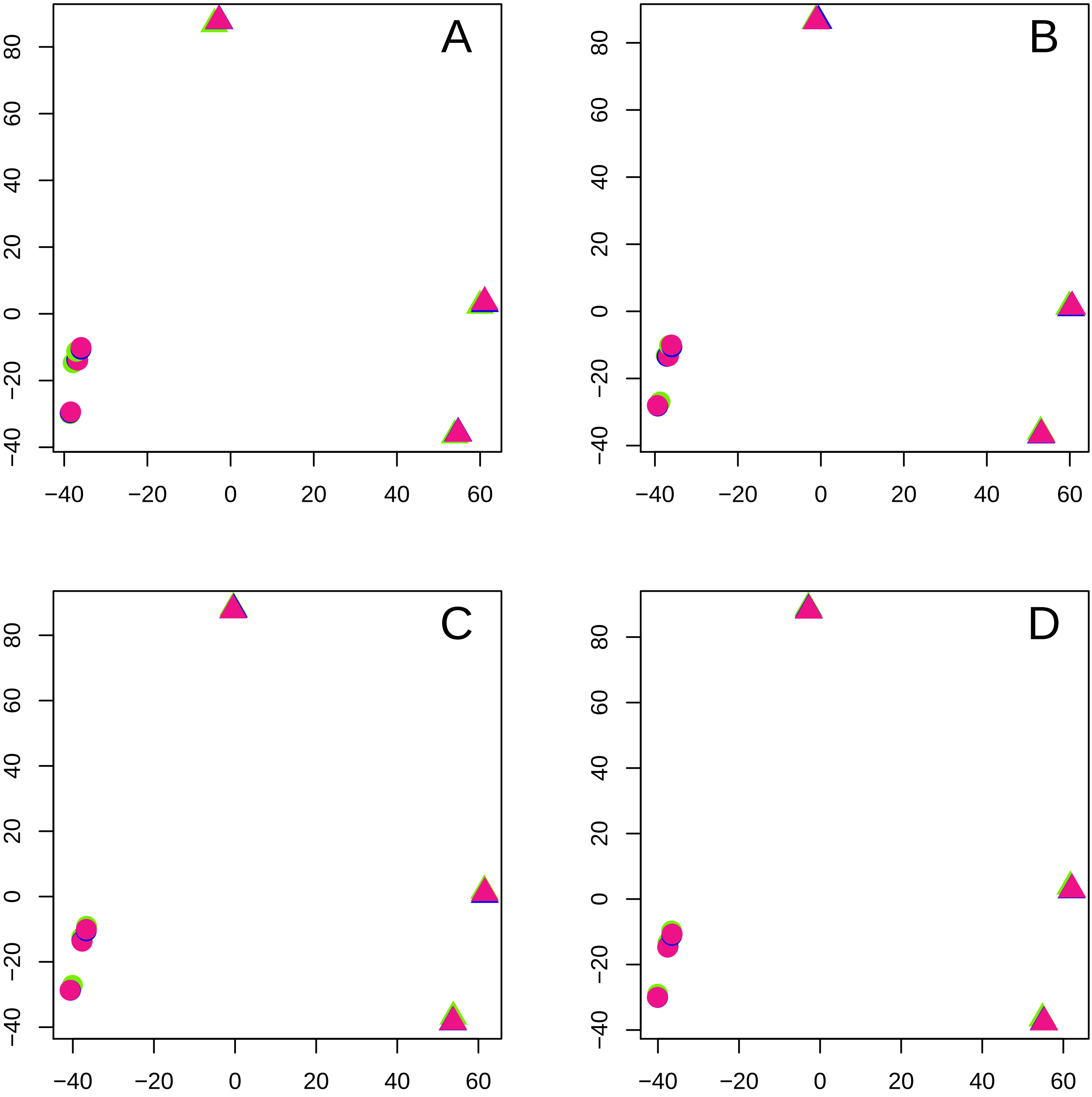
A
B
C
D

Additional File 23. Compendium of figures for data from the *Wolbachia* endosymbiont *w*Bm in adult male and adult female *B. malayi* hosts with results separated by read length. A heatmap with hierarchical clustering with statistical support is shown on page 1 with the condition denoted according to letter code from Supplementary Table 2, followed by the replicate designation and the pairing status such that (0) paired reads, (1) first-in-read single end read, and (2) second-in-read single end read. A PCA plot is shown on page 2 where the conditions are denoted by the shape and the pairing status by the color (green, paired end; blue, first-in-pair single end read; magenta, second-in-pair single end read). On both pages, results are shown in the four panels: (A) 36-bp reads, (B) 54-bp reads, (C) 72-bp reads, and (D) 101-bp reads.

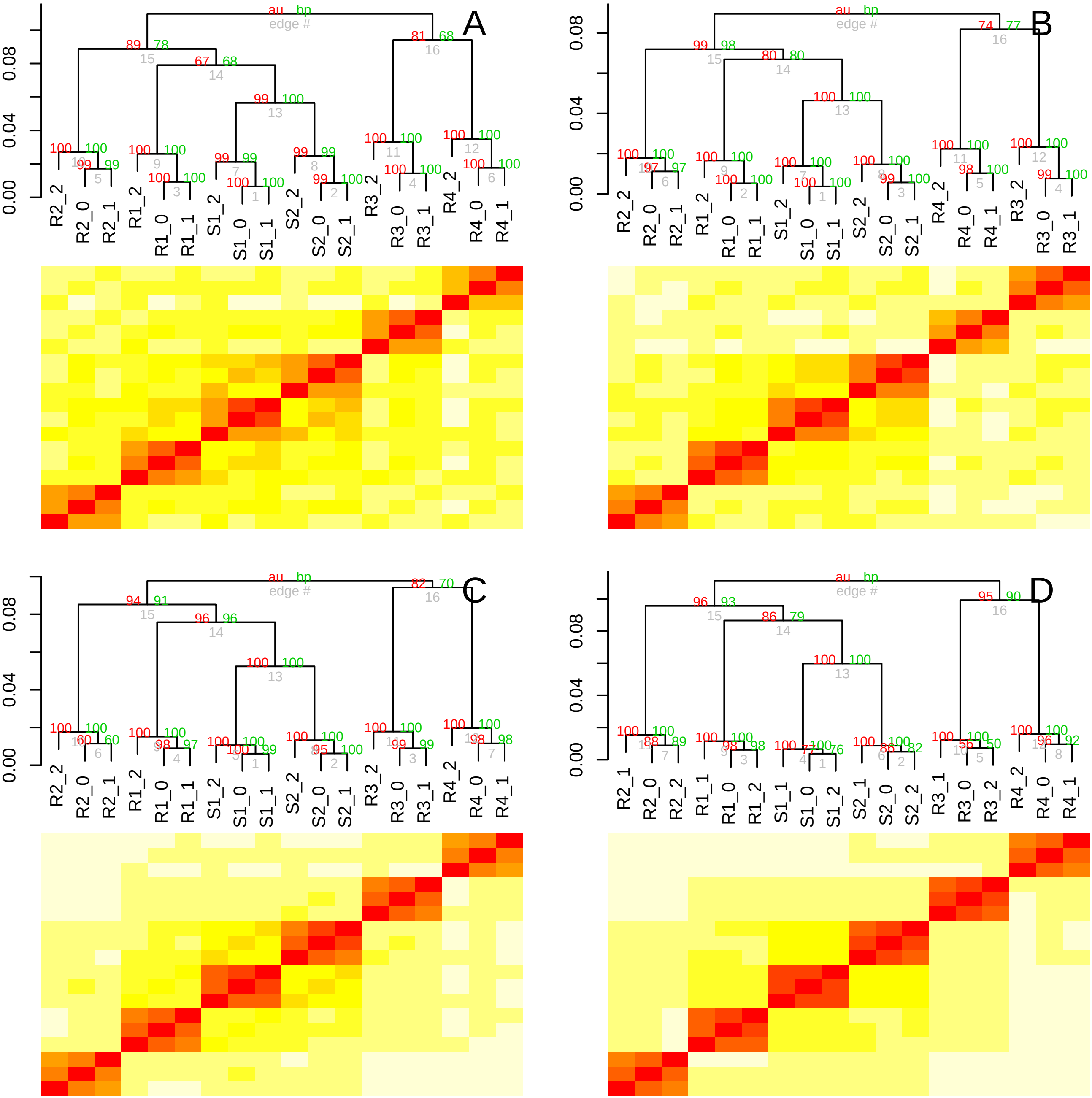
A
B
C
D
au
bp
edge #
0.00
0.04
0.08
R2_2
R2_0
R2_1
R1_2
R1_0
R1_1
S1_2
S1_0
S1_1
S2_2
S2_0
S2_1
R3_2
R3_0
R3_1
R4_2
R4_0
R4_1

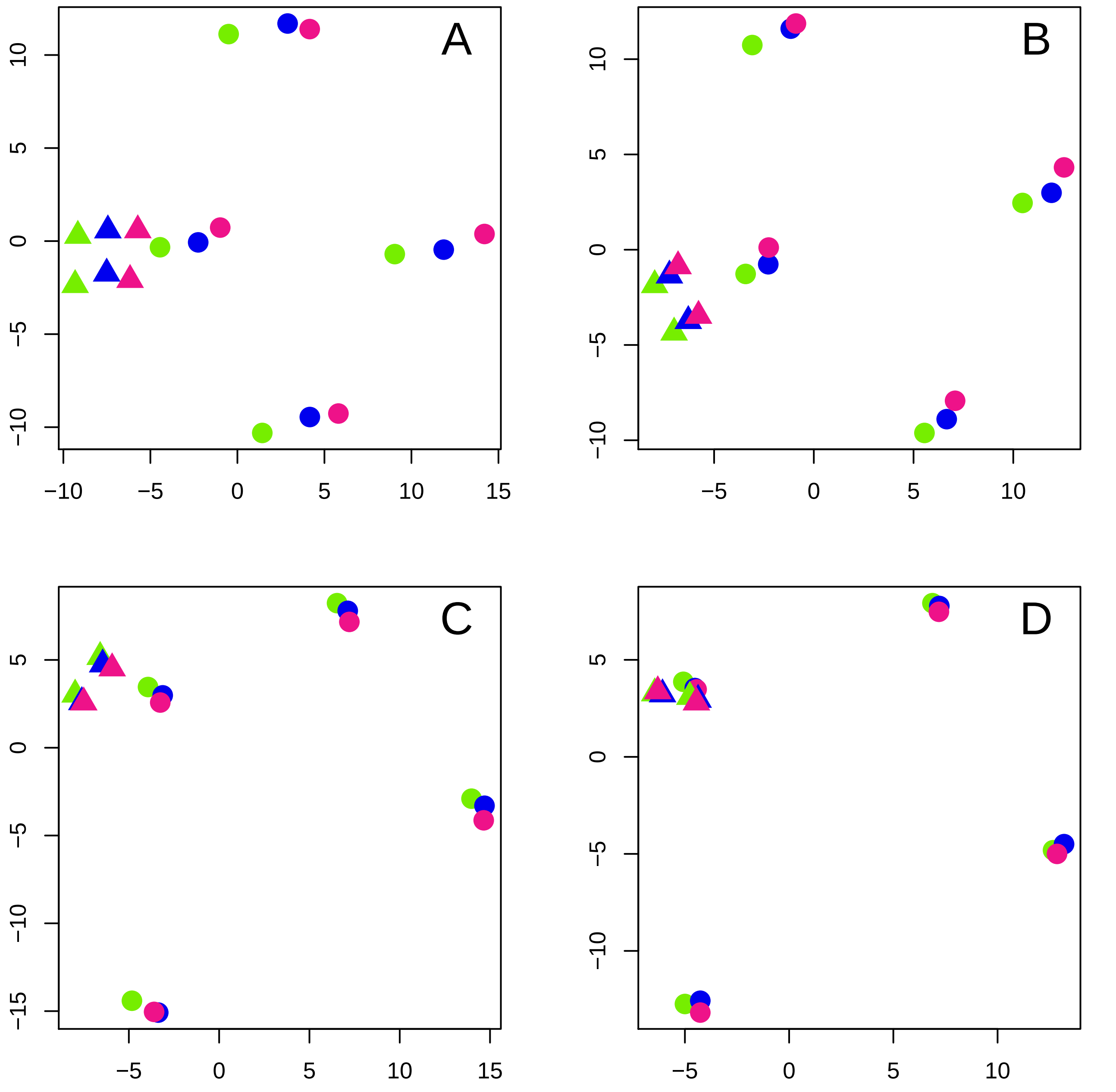
A
B
C
D

Additional File 24. Compendium of figures for data from adult male and adult female *B. malayi* with results separated by read length. A heatmap with hierarchical clustering with statistical support is shown on page 1 with the condition denoted according to letter code from Supplementary Table 2, followed by the replicate designation and the pairing status such that (0) paired reads, (1) first-in-read single end read, and (2) second-in-read single end read. A PCA plot is shown on page 2 where the conditions are denoted by the shape and the pairing status by the color (green, paired end; blue, first-in-pair single end read; magenta, second-in-pair single end read). On both pages, results are shown in the four panels: (A) 36-bp reads, (B) 54-bp reads, (C) 72-bp reads, and (D) 101-bp reads.

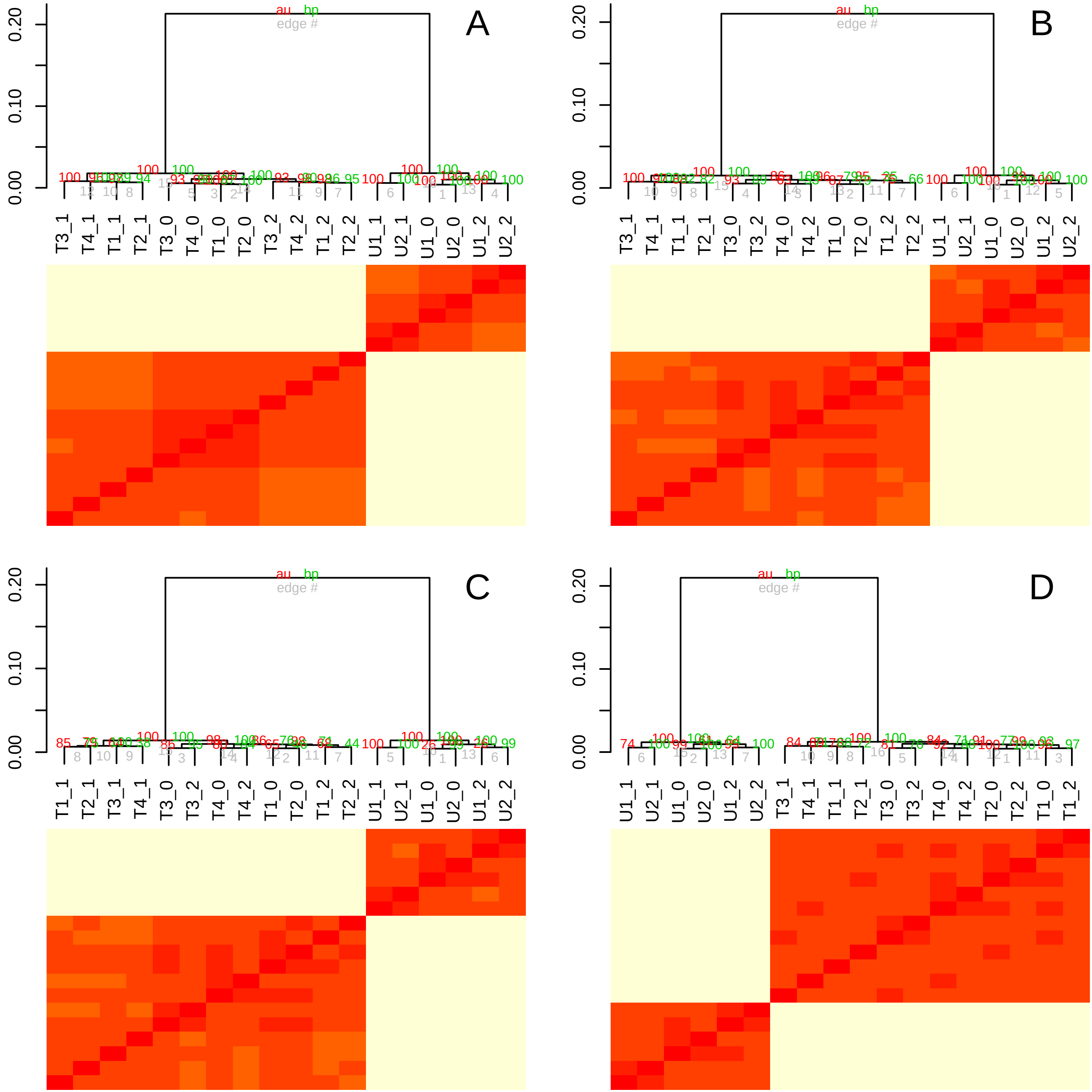
A
B
C
D
au
bp
edge #
0.00
0.10
0.20
T3_1 T4_1 T1_1 T2_1 T3_0 T4_0 T1_0 T2_0 T3_2 T4_2 T1_2 T2_2 U1_1 U2_1 U1_0 U2_0 U1_2 U2_2
T3_1 T4_1 T1_1 T2_1 T3_0 T3_2 T4_0 T4_2 T1_0 T2_0 T1_2 T2_2 U1_1 U2_1 U1_0 U2_0 U1_2 U2_2
T1_1 T2_1 T3_1 T4_1 T3_0 T3_2 T4_0 T4_2 T1_0 T2_0 T1_2 T2_2 U1_1 U2_1 U1_0 U2_0 U1_2 U2_2
U1_1 U2_1 U1_0 U2_0 U1_2 U2_2 T3_1 T4_1 T1_1 T2_1 T3_0 T3_2 T4_0 T4_2 T2_0 T2_2 T1_0 T1_2

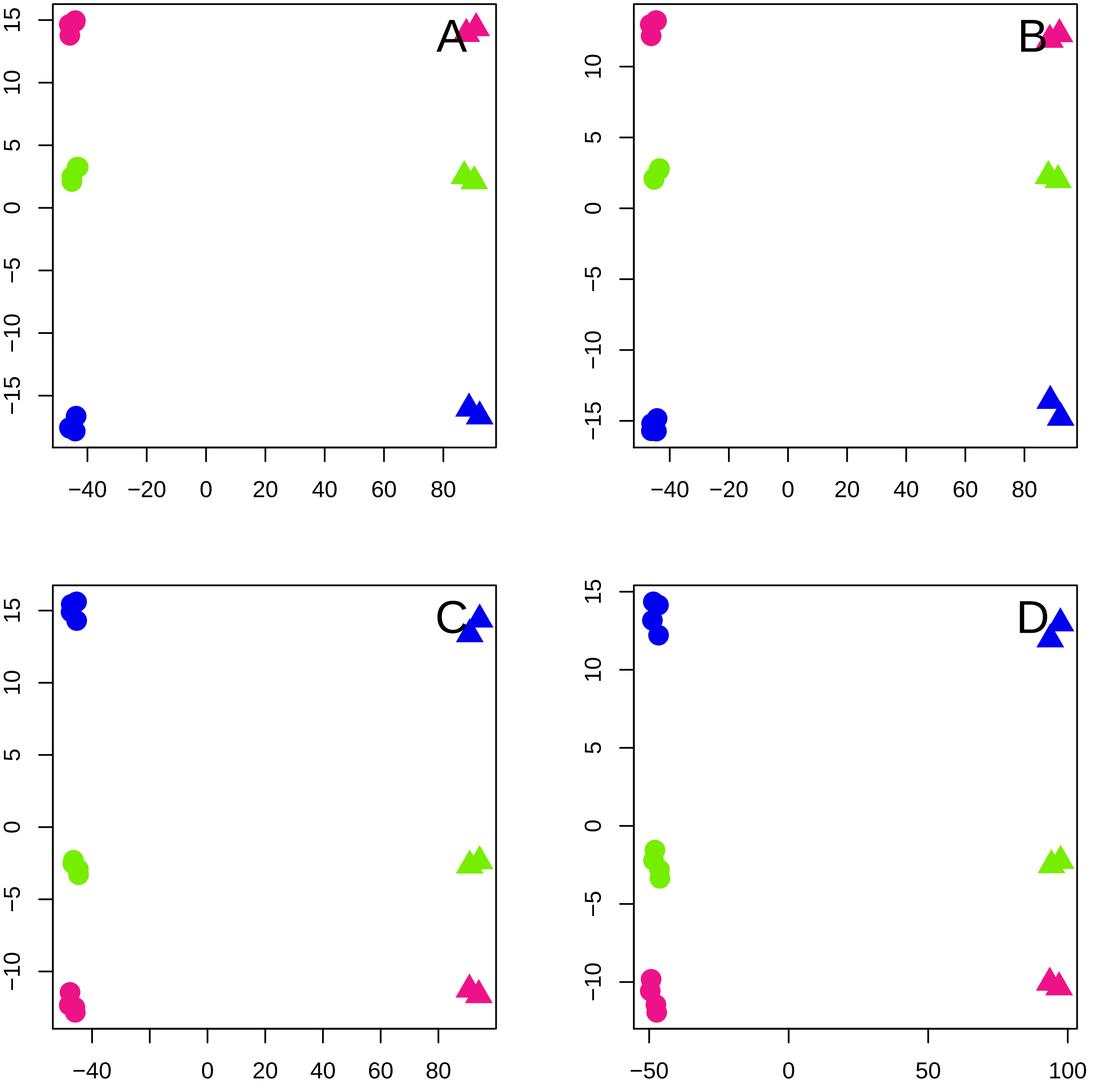
A
B
C
D
−40
−20
0
20
40
60
80
15
10
5
0
−5
−10
−15
−50
50
100

Additional File 25. Compendium of figures for data from *H. pylori* at two time points post infection of a human cell line with results separated by read length. A heatmap with hierarchical clustering with statistical support is shown on page 1 with the condition denoted according to letter code from Supplementary Table 2, followed by the replicate designation and the pairing status such that (0) paired reads, (1) first-in-read single end read, and (2) second-in-read single end read. A PCA plot is shown on page 2 where the conditions are denoted by the shape (circle, rh2_comp; triangle, rh2_del) and the pairing status by the color (green, paired end; blue, first-in-pair single end read; magenta, second-in-pair single end read). On both pages, results are shown in the four panels: (A) 36-bp reads, (B) 54-bp reads, (C) 72-bp reads, and (D) 101-bp reads.

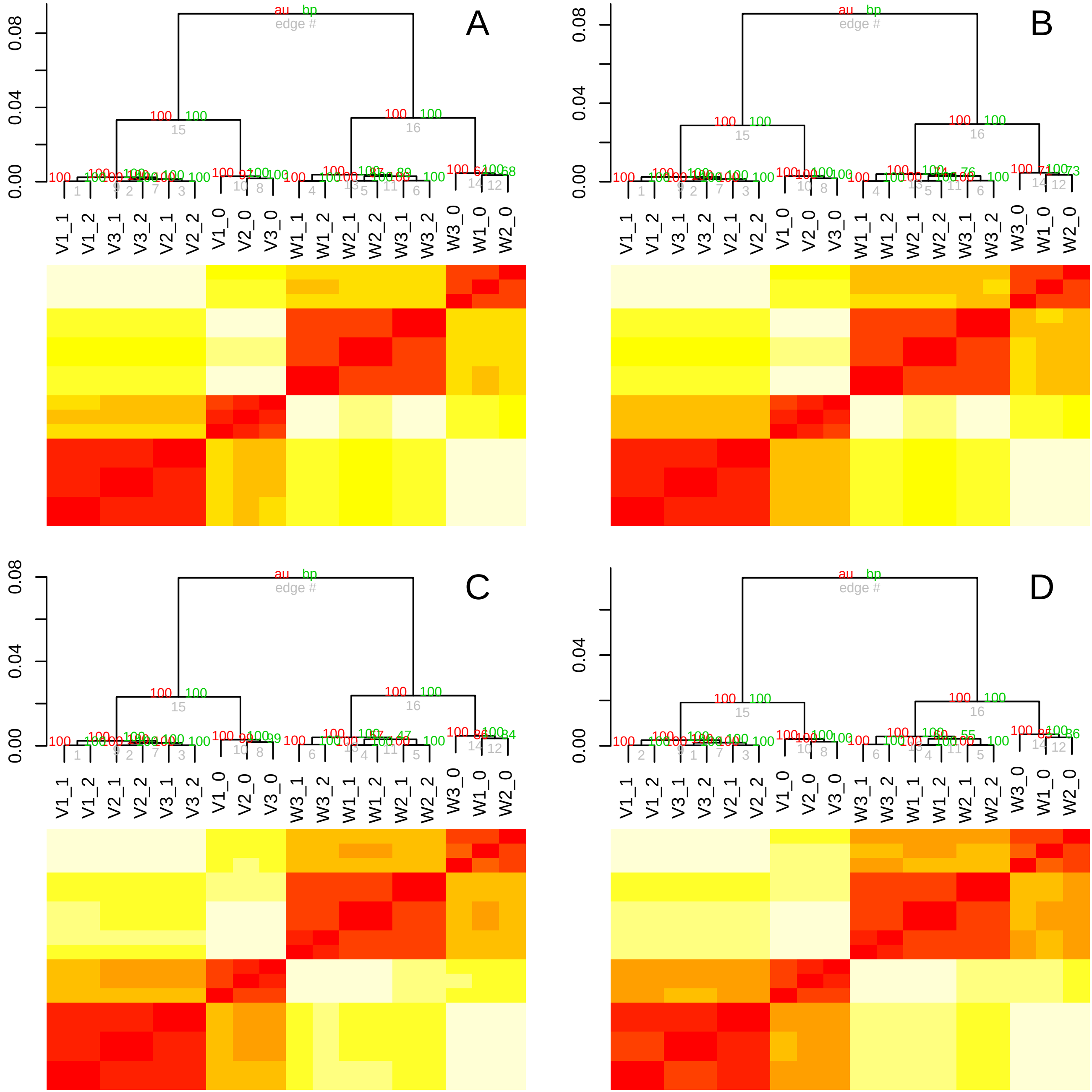
A
B
C
D
au
bp
edge #
0.00
0.04
0.08
V1_1 V1_2 V3_1 V3_2 V2_1 V2_2 V1_0 V2_0 V3_0 W1_1 W1_2 W2_1 W2_2 W3_1 W3_2 W3_0 W1_0 W2_0
V1_1 V1_2 V2_1 V2_2 V3_1 V3_2 V1_0 V2_0 V3_0 W3_1 W3_2 W1_1 W1_2 W2_1 W2_2 W3_0 W1_0 W2_0
V1_1 V1_2 V3_1 V3_2 V2_1 V2_2 V1_0 V2_0 V3_0 W3_1 W3_2 W1_1 W1_2 W2_1 W2_2 W3_0 W1_0 W2_0

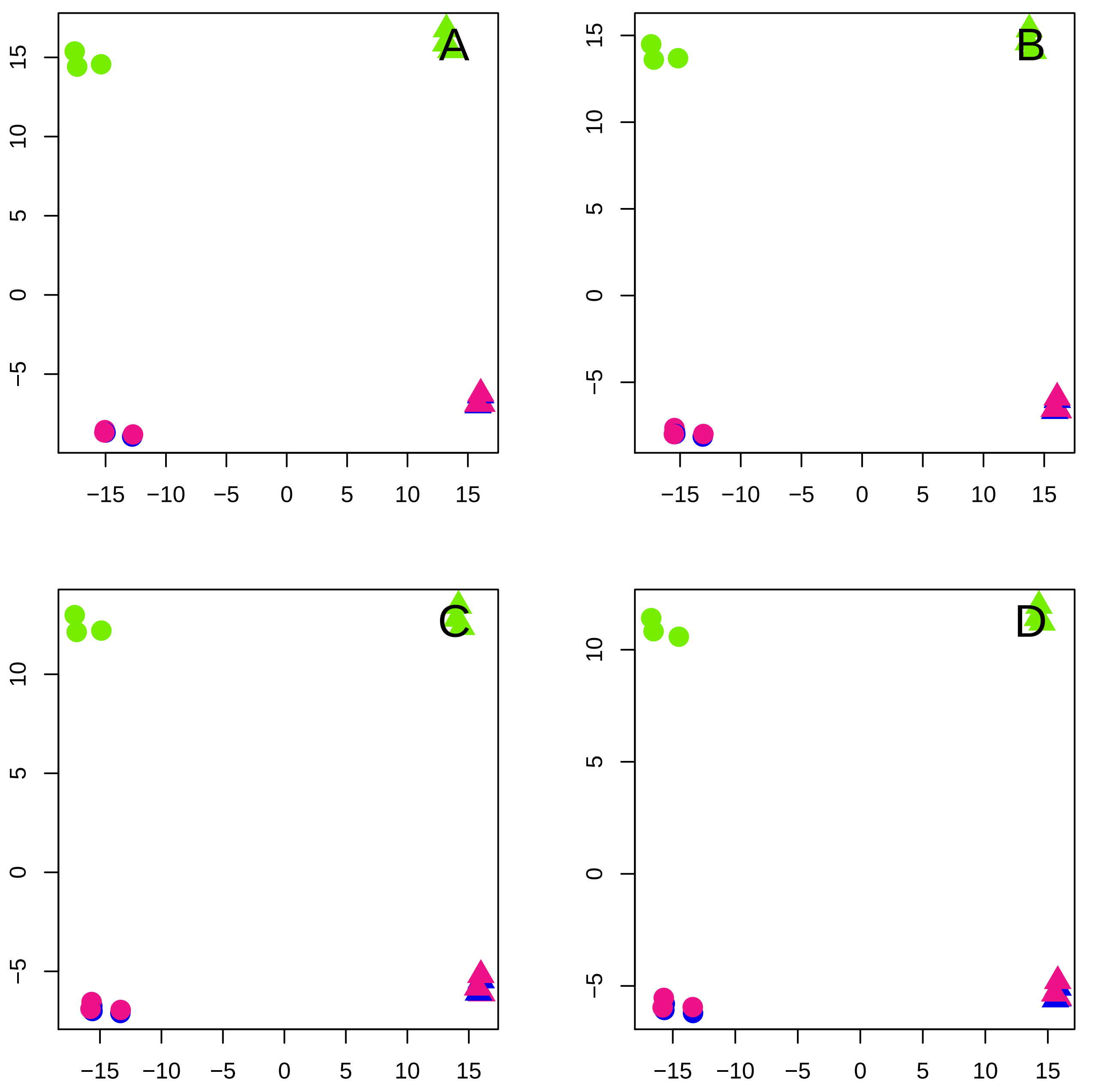
A
B
C
D
−15
−10
−5
0
5
10
15

Additional File 26. Compendium of figures for data from human cells infected with *H. pylori* at two time points with results separated by read length. A heatmap with hierarchical clustering with statistical support is shown on page 1 with the condition denoted according to Supplementary Table 2, followed by the replicate designation and the pairing status such that (0) paired reads, (1) first-in-read single end read, and (2) second-in-read single end read. A PCA plot is shown on page 2 where the conditions are denoted by the shape (circle, DMEM; triangle, LB) and the pairing status by the color (green, paired end; blue, first-in-pair single end read; magenta, second-in-pair single end read). On both pages, results are shown in the four panels: (A) 36-bp reads, (B) 54-bp reads, (C) 72-bp reads, and (D) 101-bp reads.

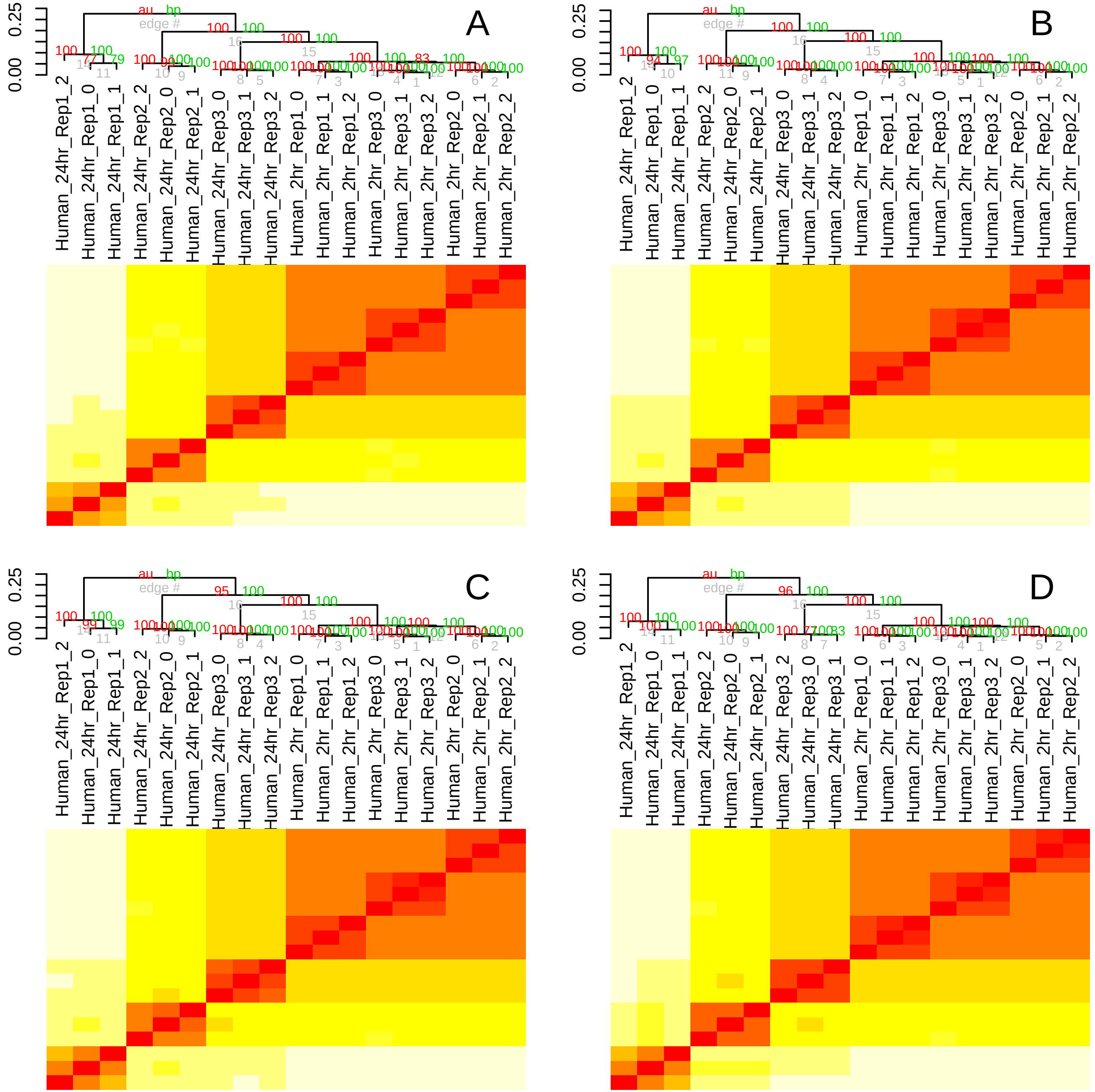
A
B
C
D
au
bp
edge #
0.00
0.25
Human_24hr_Rep1_2
Human_24hr_Rep1_0
Human_24hr_Rep1_1
Human_24hr_Rep2_2
Human_24hr_Rep2_0
Human_24hr_Rep2_1
Human_24hr_Rep3_0
Human_24hr_Rep3_1
Human_24hr_Rep3_2
Human_2hr_Rep1_0
Human_2hr_Rep1_1
Human_2hr_Rep1_2
Human_2hr_Rep3_0
Human_2hr_Rep3_1
Human_2hr_Rep3_2
Human_2hr_Rep2_0
Human_2hr_Rep2_1
Human_2hr_Rep2_2

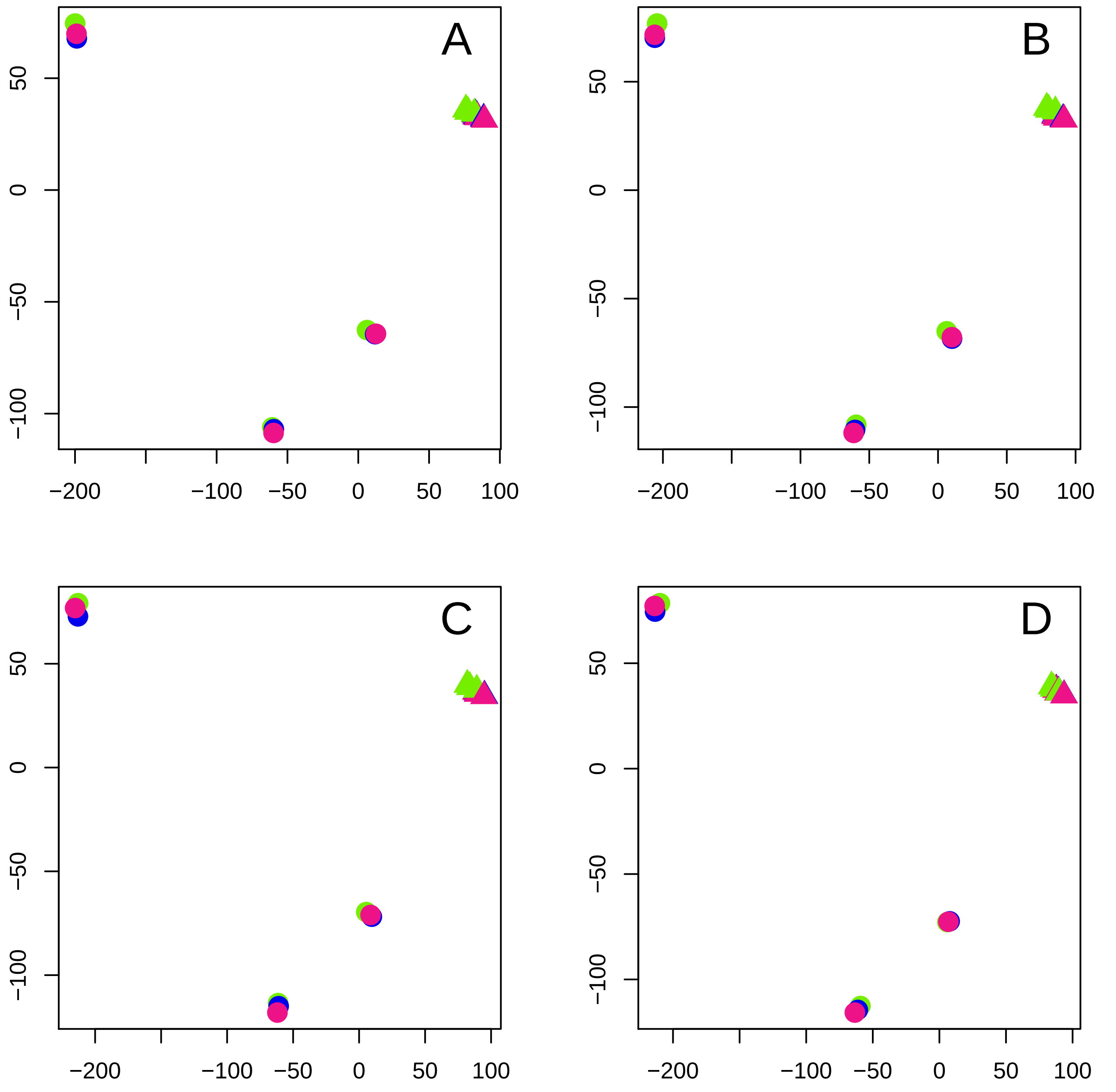

A
B
C
D
50
0
−50
−100
−200
−100
−50
0
50
100

Additional File 27. Compendium of figures for data from *P. aeruginosa* in stationary and exponential phase with results separated by read length. A heatmap with hierarchical clustering with statistical support is shown on page 1 with the condition denoted according to Supplementary Table 2, followed by the replicate designation and the pairing status such that (0) paired reads, (1) first-in-read single end read, and (2) second-in-read single end read. A PCA plot is shown on page 2 where the conditions are denoted by the shape (circle, DMEM; triangle, LB) and the pairing status by the color (green, paired end; blue, first-in-pair single end read; magenta, second-in-pair single end read). On both pages, results are shown in the four panels: (A) 36-bp reads, (B) 54-bp reads, (C) 72-bp reads, and (D) 101-bp reads.

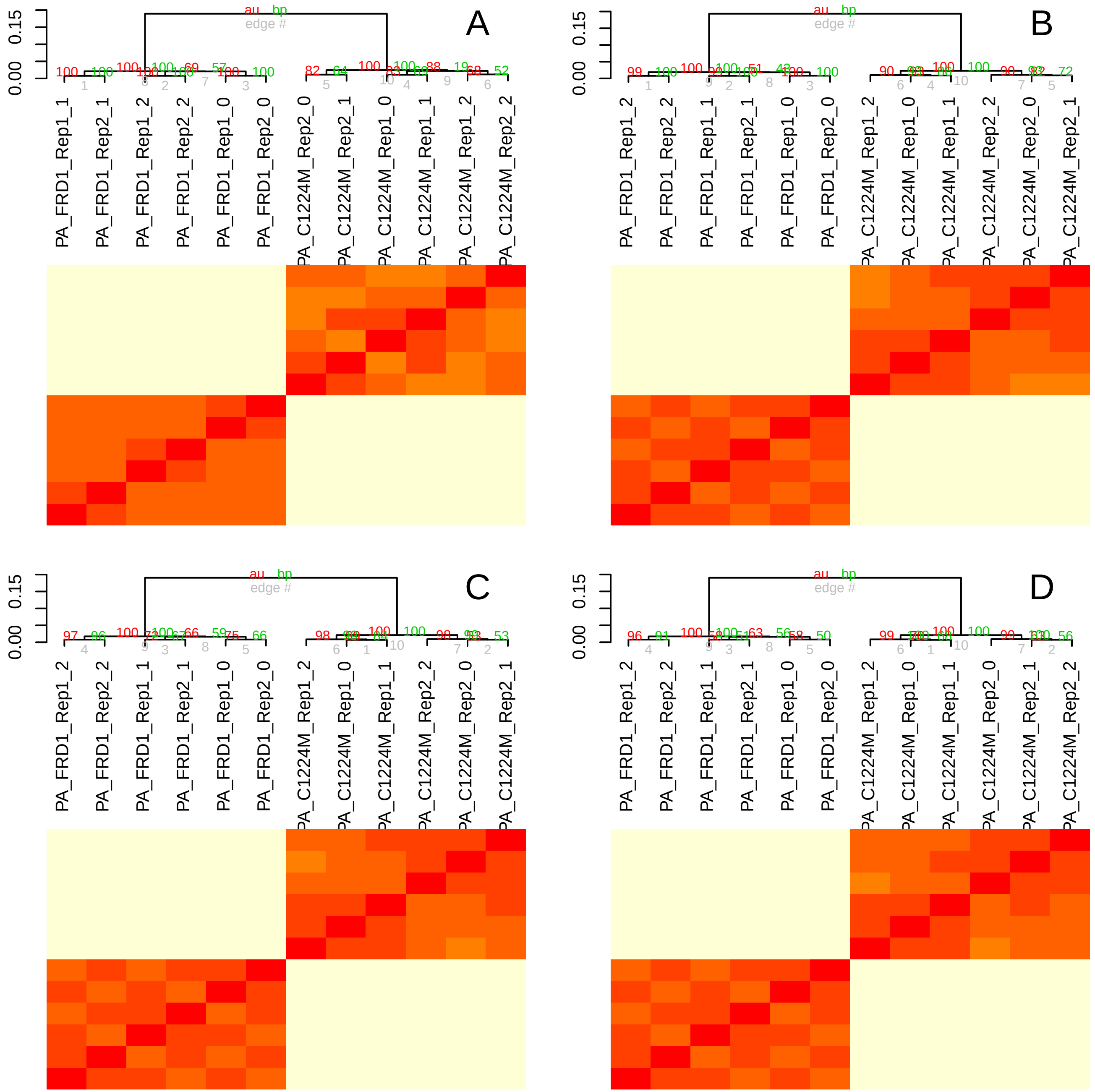
A
B
C
D
au
bp
edge #
0.00
0.15
PA_FRD1_Rep1_1
PA_FRD1_Rep2_1
PA_FRD1_Rep1_2
PA_FRD1_Rep2_2
PA_FRD1_Rep1_0
PA_FRD1_Rep2_0
PA_C1224M_Rep2_0
PA_C1224M_Rep2_1
PA_C1224M_Rep1_0
PA_C1224M_Rep1_1
PA_C1224M_Rep1_2
PA_C1224M_Rep2_2

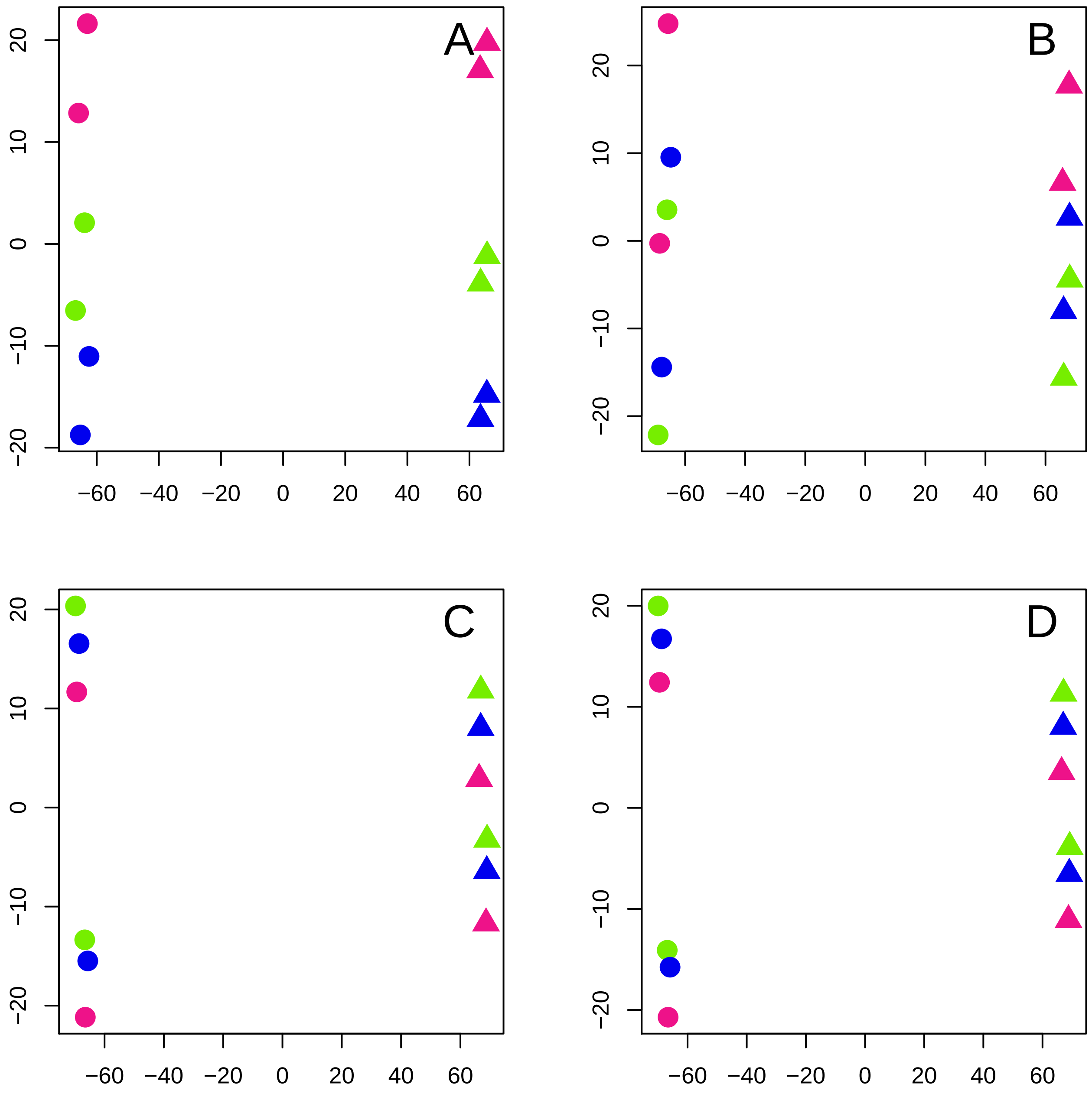
A
B
C
D
−60 −40 −20 0 20 40 60
−20 −10 0 10 20

Additional File 28. Compendium of figures for data from two *P. aeruginosa* clinical isolates with results separated by read length. A heatmap with hierarchical clustering with statistical support is shown on page 1 with the condition denoted according to Supplementary Table 2, followed by the replicate designation and the pairing status such that (0) paired reads, (1) first-in-read single end read, and (2) second-in-read single end read. A PCA plot is shown on page 2 where the conditions are denoted by the shape (circle, DMEM; triangle, LB) and the pairing status by the color (green, paired end; blue, first-in-pair single end read; magenta, second-in-pair single end read). On both pages, results are shown in the four panels: (A) 36-bp reads, (B) 54-bp reads, (C) 72-bp reads, and (D) 101-bp reads.

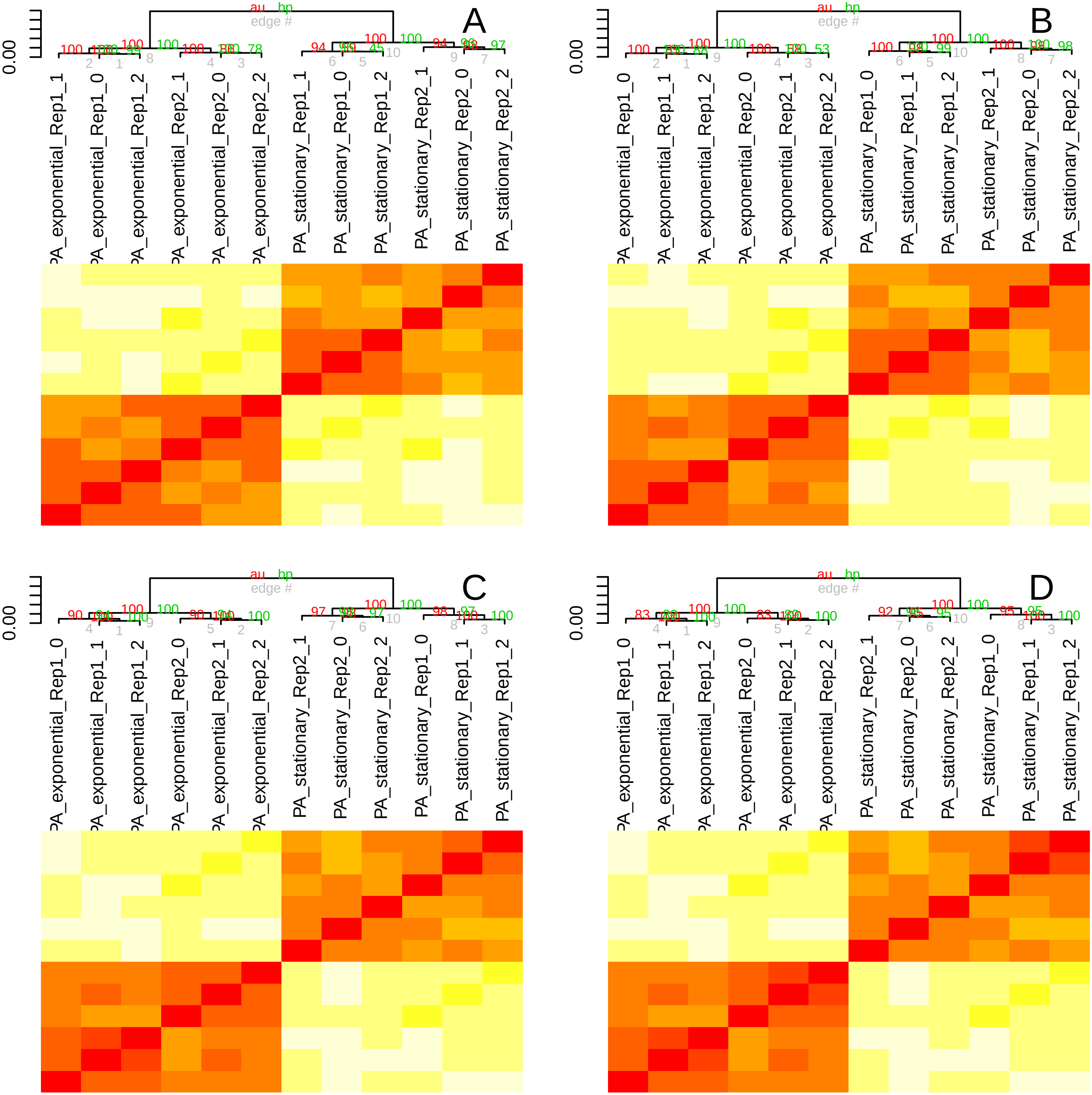

A
B
C
D
au
bp
edge #
0.00
PA_exponential_Rep1_1
PA_exponential_Rep1_0
PA_exponential_Rep1_2
PA_exponential_Rep2_1
PA_exponential_Rep2_0
PA_exponential_Rep2_2
PA_stationary_Rep1_1
PA_stationary_Rep1_0
PA_stationary_Rep1_2
PA_stationary_Rep2_1
PA_stationary_Rep2_0
PA_stationary_Rep2_2

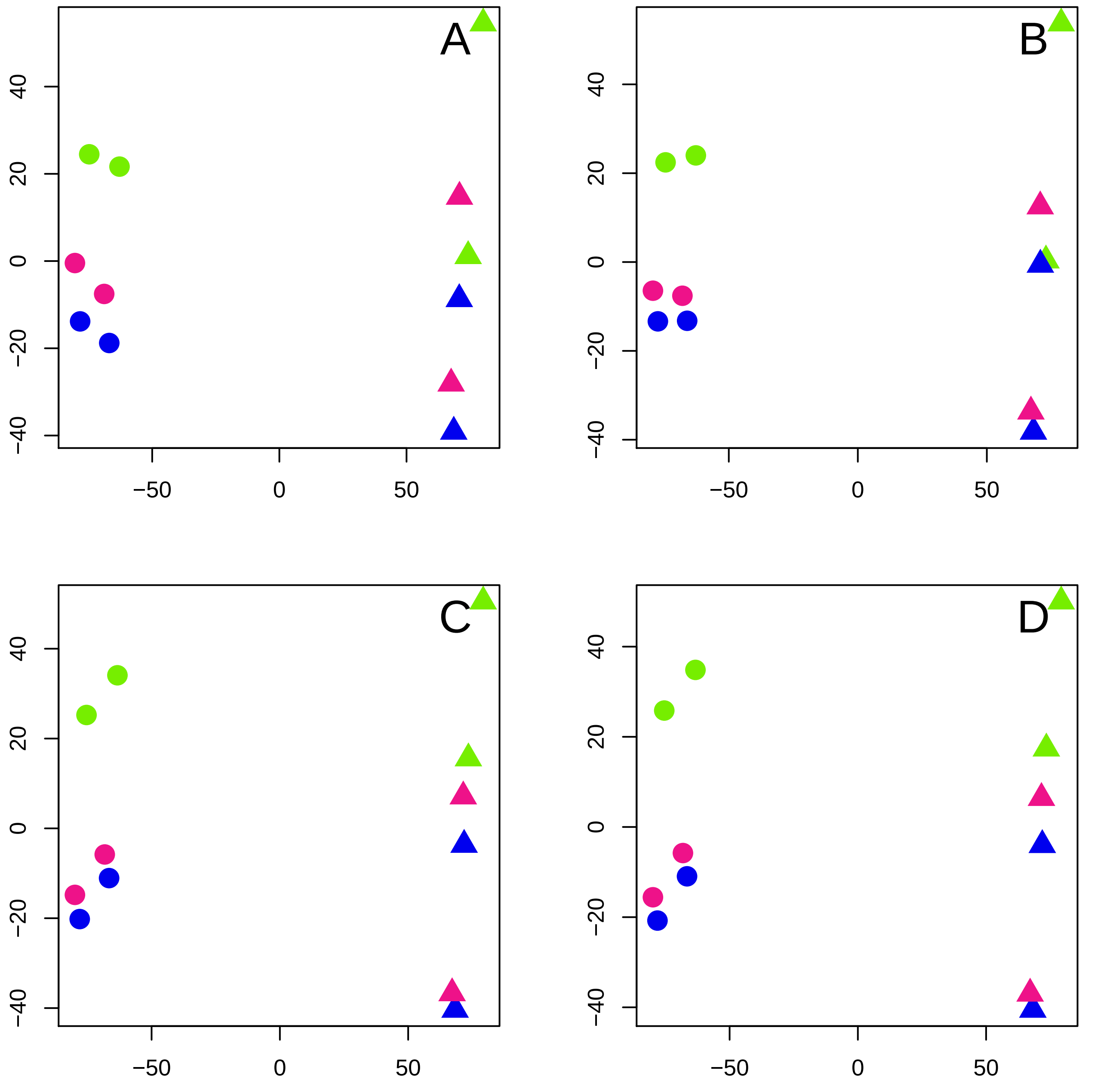

A
B
C
D
40
20
0
−20
−40
−50
0
50

Additional File 29. Compendium of scatterplots for all data sets with results aggregated by read length. The differentially expressed genes identified using an adjusted p-value (FDR) cutoff ≤0.05 at varying read lengths within a dataset were compared using Pearson's correlation implemented in the R statistical tool and illustrated as a matrix of scatterplots. The diagonal represents the histogram of log-transformed fold-changes within the comparison. The lower plots represent the correlation between comparisons with singleton DEGs identified for comparisons on the x-axis (pink) and y-axis (green). Genes with FDR > 0.05 in both comparisons are not shown. The upper portion of the plot lists the corresponding Pearson's correlation coefficient and the number of singleton DEGs identified in each comparison. Each scatterplot is labeled by the comparison according to the letter code from Supplementary Table 2. A separate plot is shown for paired reads (labelled "0"), first read in pair (labelled "1"), and second read in pair (labelled "2").

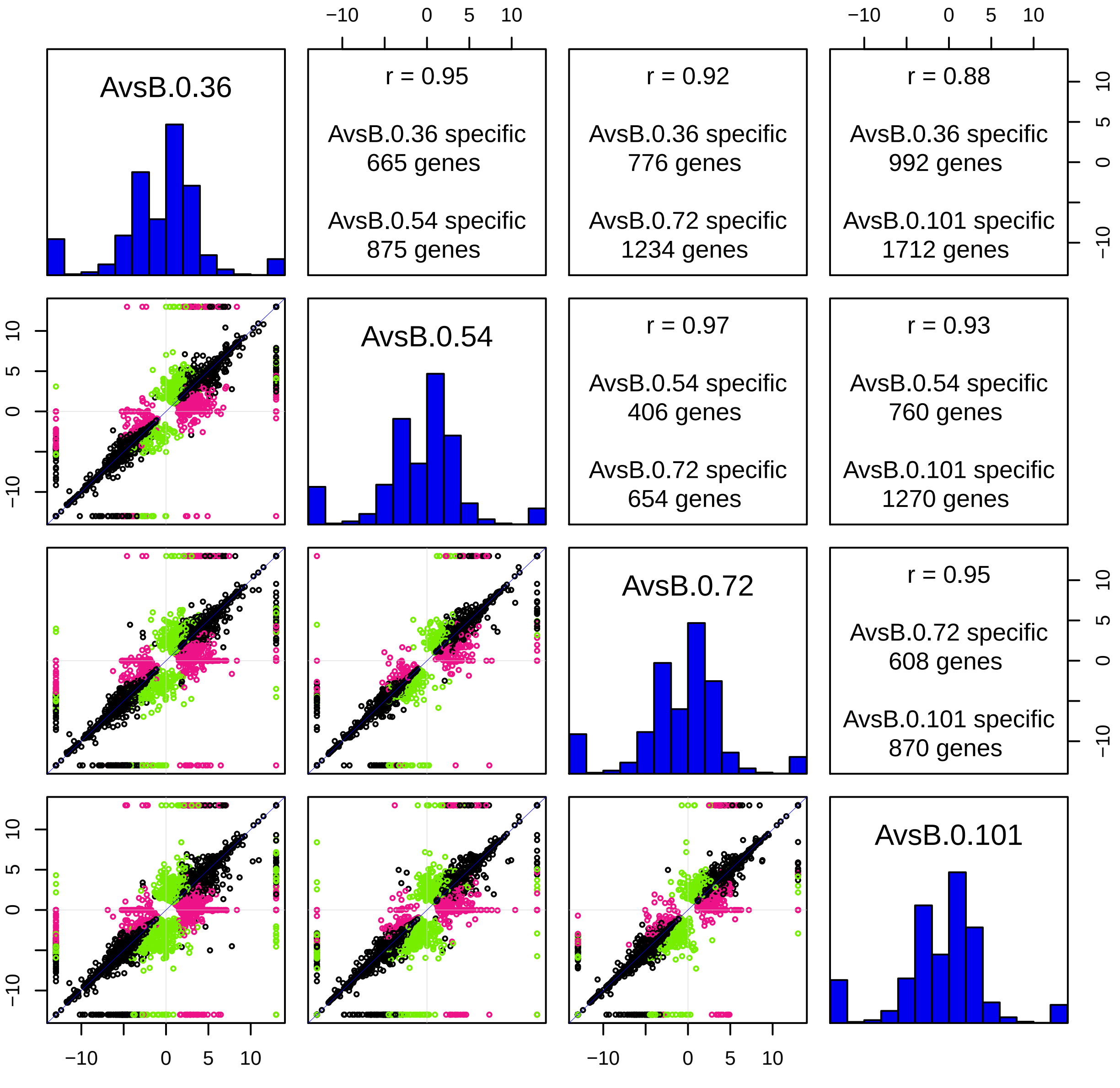
AvsB.0.36
r = 0.95
AvsB.0.36 specific
665 genes
AvsB.0.54 specific
875 genes
r = 0.92
AvsB.0.36 specific
776 genes
AvsB.0.72 specific
1234 genes
r = 0.88
AvsB.0.36 specific
992 genes
AvsB.0.101 specific
1712 genes
AvsB.0.54
r = 0.97
AvsB.0.54 specific
406 genes
AvsB.0.72 specific
654 genes
r = 0.93
AvsB.0.54 specific
760 genes
AvsB.0.101 specific
1270 genes
AvsB.0.72
r = 0.95
AvsB.0.72 specific
608 genes
AvsB.0.101 specific
870 genes
AvsB.0.101
−10
0
5
10

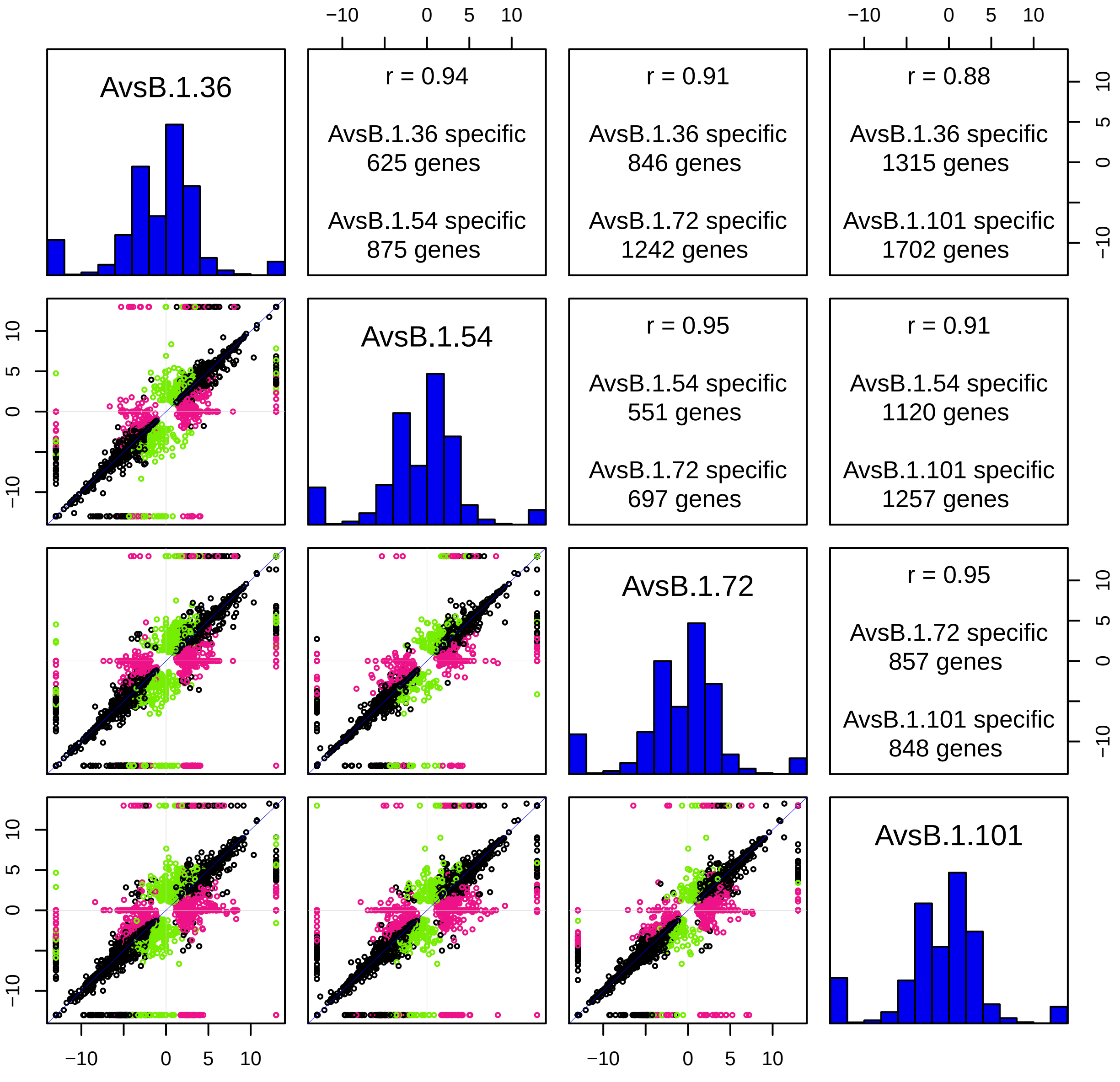
AvsB.1.36
r = 0.94
AvsB.1.36 specific
625 genes
AvsB.1.54 specific
875 genes
r = 0.91
AvsB.1.36 specific
846 genes
AvsB.1.72 specific
1242 genes
r = 0.88
AvsB.1.36 specific
1315 genes
AvsB.1.101 specific
1702 genes
AvsB.1.54
r = 0.95
AvsB.1.54 specific
551 genes
AvsB.1.72 specific
697 genes
r = 0.91
AvsB.1.54 specific
1120 genes
AvsB.1.101 specific
1257 genes
AvsB.1.72
r = 0.95
AvsB.1.72 specific
857 genes
AvsB.1.101 specific
848 genes
AvsB.1.101
−10
0
5
10

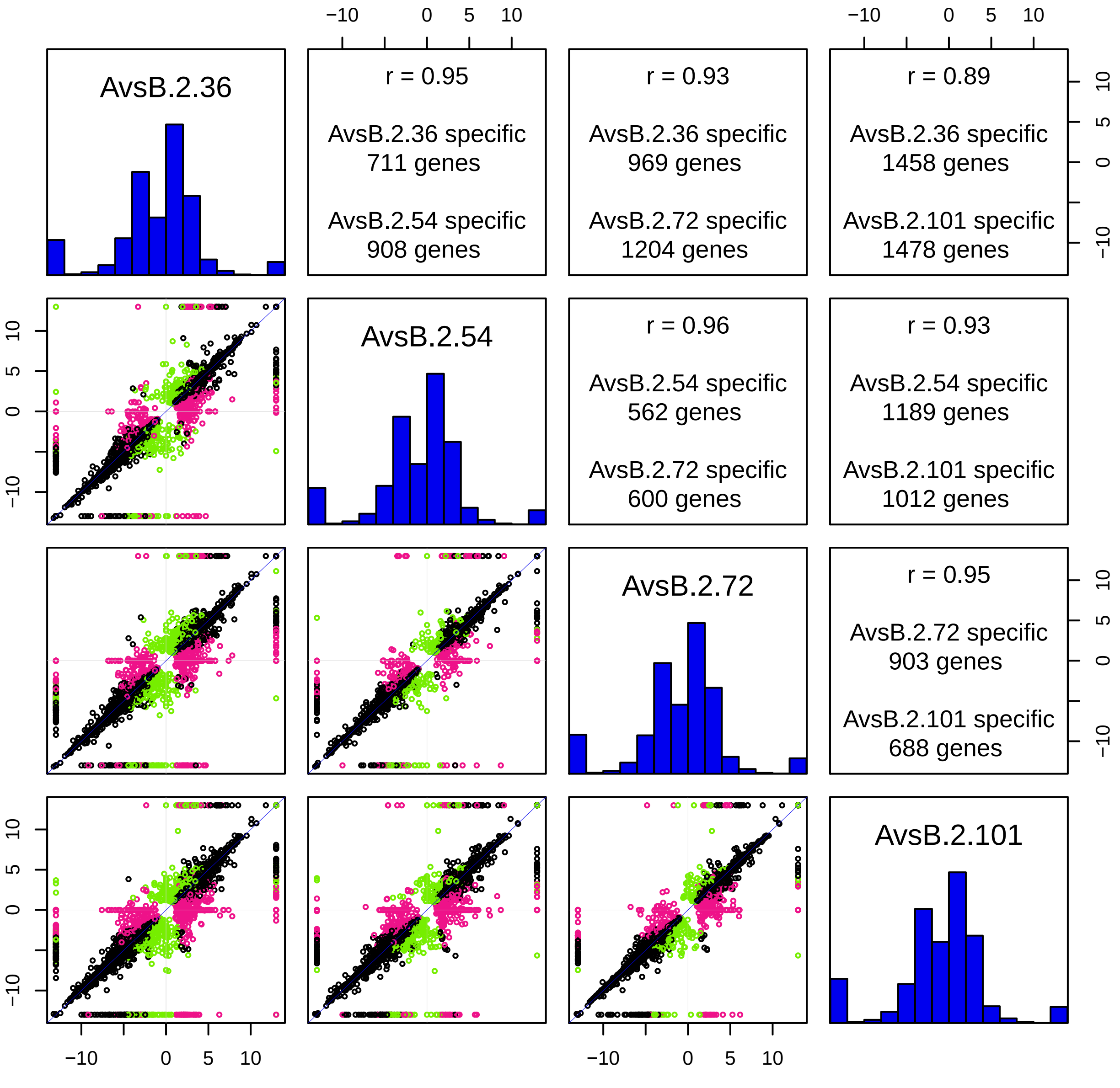
AvsB.2.36
r = 0.95
AvsB.2.36 specific
711 genes
AvsB.2.54 specific
908 genes
r = 0.93
AvsB.2.36 specific
969 genes
AvsB.2.72 specific
1204 genes
r = 0.89
AvsB.2.36 specific
1458 genes
AvsB.2.101 specific
1478 genes
AvsB.2.54
r = 0.96
AvsB.2.54 specific
562 genes
AvsB.2.72 specific
600 genes
r = 0.93
AvsB.2.54 specific
1189 genes
AvsB.2.101 specific
1012 genes
AvsB.2.72
r = 0.95
AvsB.2.72 specific
903 genes
AvsB.2.101 specific
688 genes
AvsB.2.101
−10
0
5
10

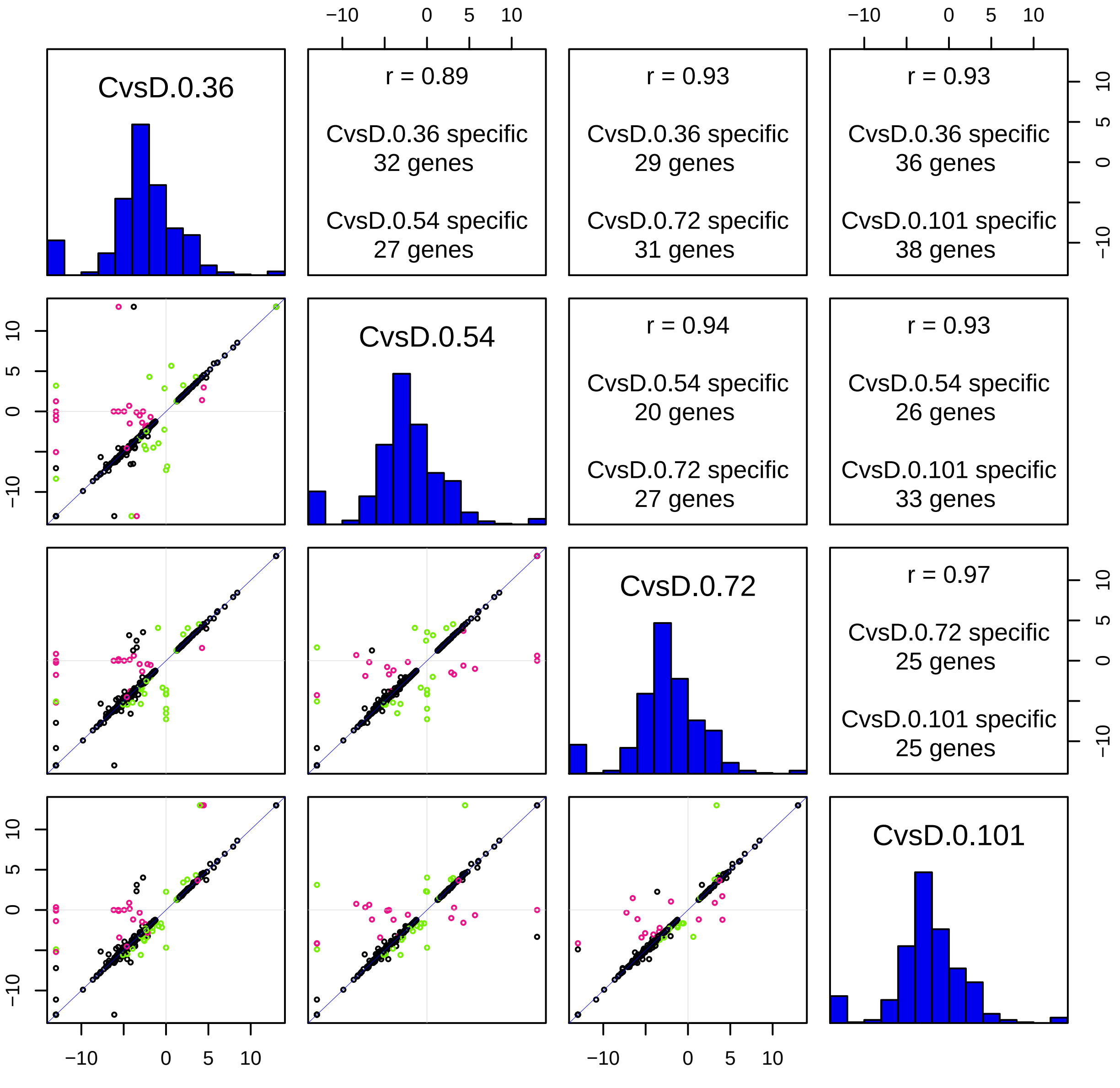

CvsD.0.36
r = 0.89
CvsD.0.36 specific
32 genes
CvsD.0.54 specific
27 genes
r = 0.93
CvsD.0.36 specific
29 genes
CvsD.0.72 specific
31 genes
r = 0.93
CvsD.0.36 specific
36 genes
CvsD.0.101 specific
38 genes
CvsD.0.54
r = 0.94
CvsD.0.54 specific
20 genes
CvsD.0.72 specific
27 genes
r = 0.93
CvsD.0.54 specific
26 genes
CvsD.0.101 specific
33 genes
CvsD.0.72
r = 0.97
CvsD.0.72 specific
25 genes
CvsD.0.101 specific
25 genes
CvsD.0.101
−10
0
5
10

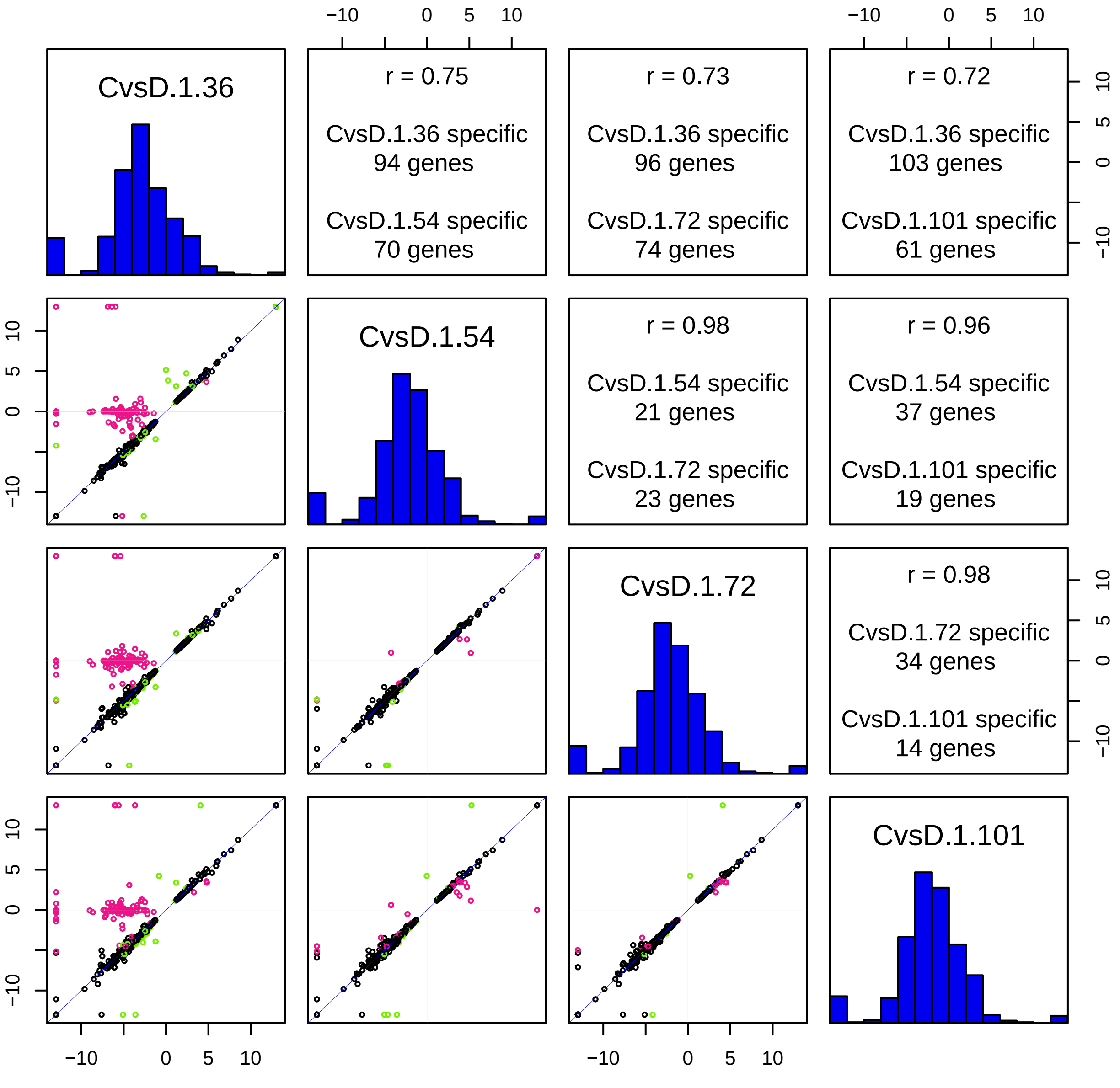

CvsD.1.36
r = 0.75
CvsD.1.36 specific
94 genes
CvsD.1.54 specific
70 genes
r = 0.73
CvsD.1.36 specific
96 genes
CvsD.1.72 specific
74 genes
r = 0.72
CvsD.1.36 specific
103 genes
CvsD.1.101 specific
61 genes
CvsD.1.54
r = 0.98
CvsD.1.54 specific
21 genes
CvsD.1.72 specific
23 genes
r = 0.96
CvsD.1.54 specific
37 genes
CvsD.1.101 specific
19 genes
CvsD.1.72
r = 0.98
CvsD.1.72 specific
34 genes
CvsD.1.101 specific
14 genes
CvsD.1.101
−10
0
5
10

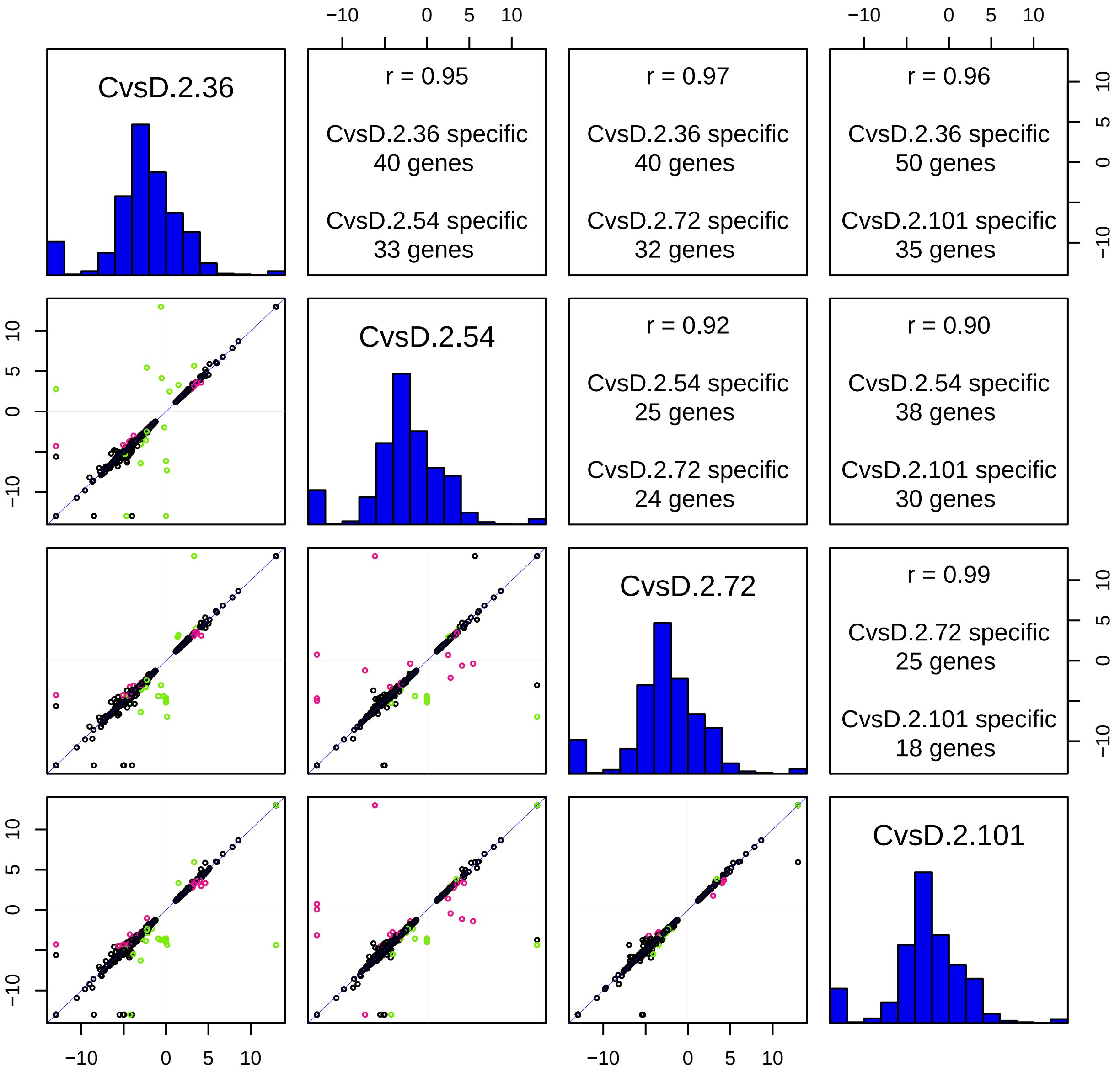

CvsD.2.36
r = 0.95
CvsD.2.36 specific
40 genes
CvsD.2.54 specific
33 genes
r = 0.97
CvsD.2.36 specific
40 genes
CvsD.2.72 specific
32 genes
r = 0.96
CvsD.2.36 specific
50 genes
CvsD.2.101 specific
35 genes
CvsD.2.54
r = 0.92
CvsD.2.54 specific
25 genes
CvsD.2.72 specific
24 genes
r = 0.90
CvsD.2.54 specific
38 genes
CvsD.2.101 specific
30 genes
CvsD.2.72
r = 0.99
CvsD.2.72 specific
25 genes
CvsD.2.101 specific
18 genes
CvsD.2.101
−10
0
5
10

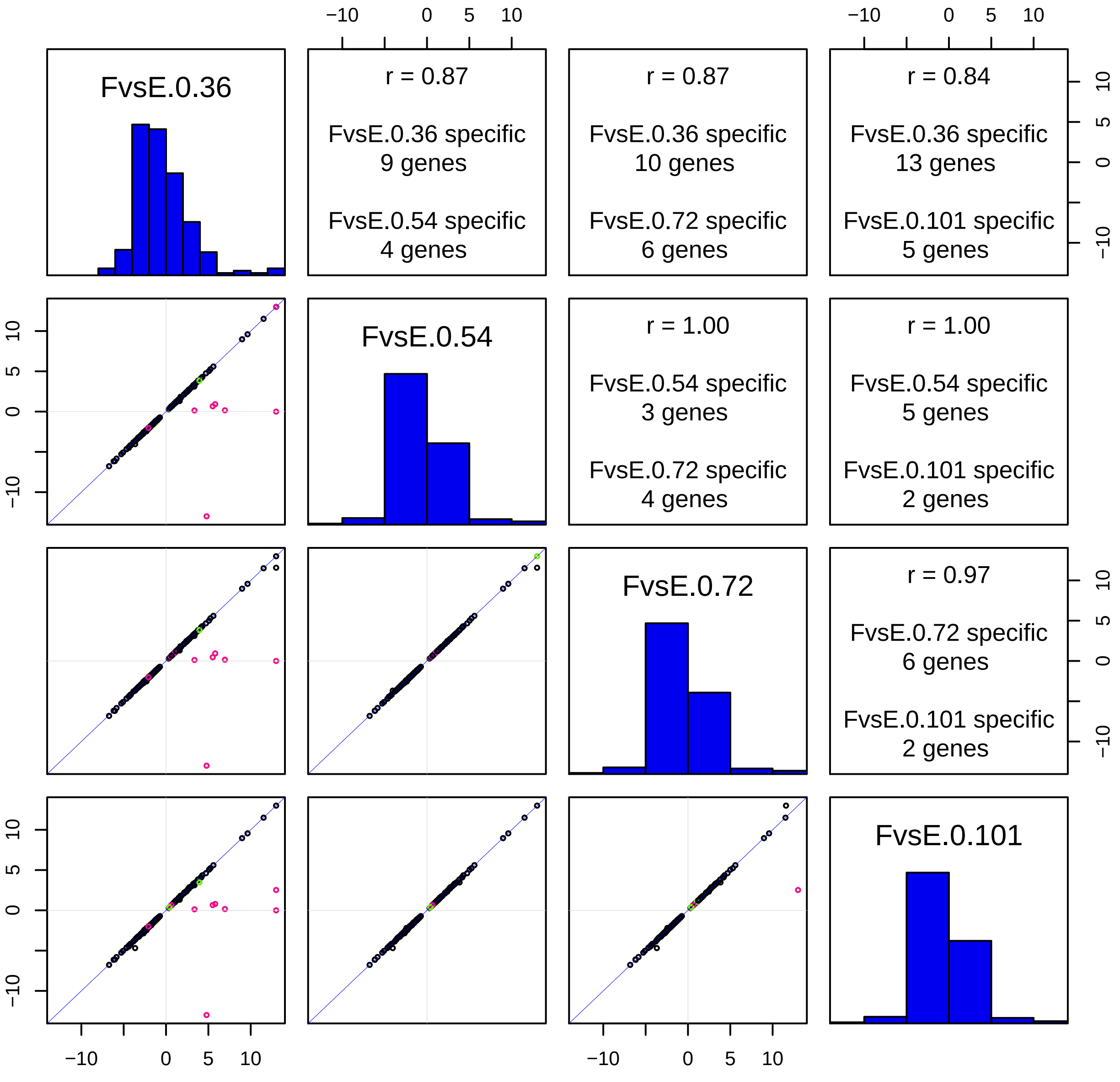

FvsE.0.36
r = 0.87
FvsE.0.36 specific
9 genes
FvsE.0.54 specific
4 genes
r = 0.87
FvsE.0.36 specific
10 genes
FvsE.0.72 specific
6 genes
r = 0.84
FvsE.0.36 specific
13 genes
FvsE.0.101 specific
5 genes
FvsE.0.54
r = 1.00
FvsE.0.54 specific
3 genes
FvsE.0.72 specific
4 genes
r = 1.00
FvsE.0.54 specific
5 genes
FvsE.0.101 specific
2 genes
FvsE.0.72
r = 0.97
FvsE.0.72 specific
6 genes
FvsE.0.101 specific
2 genes
FvsE.0.101
−10
0
5
10

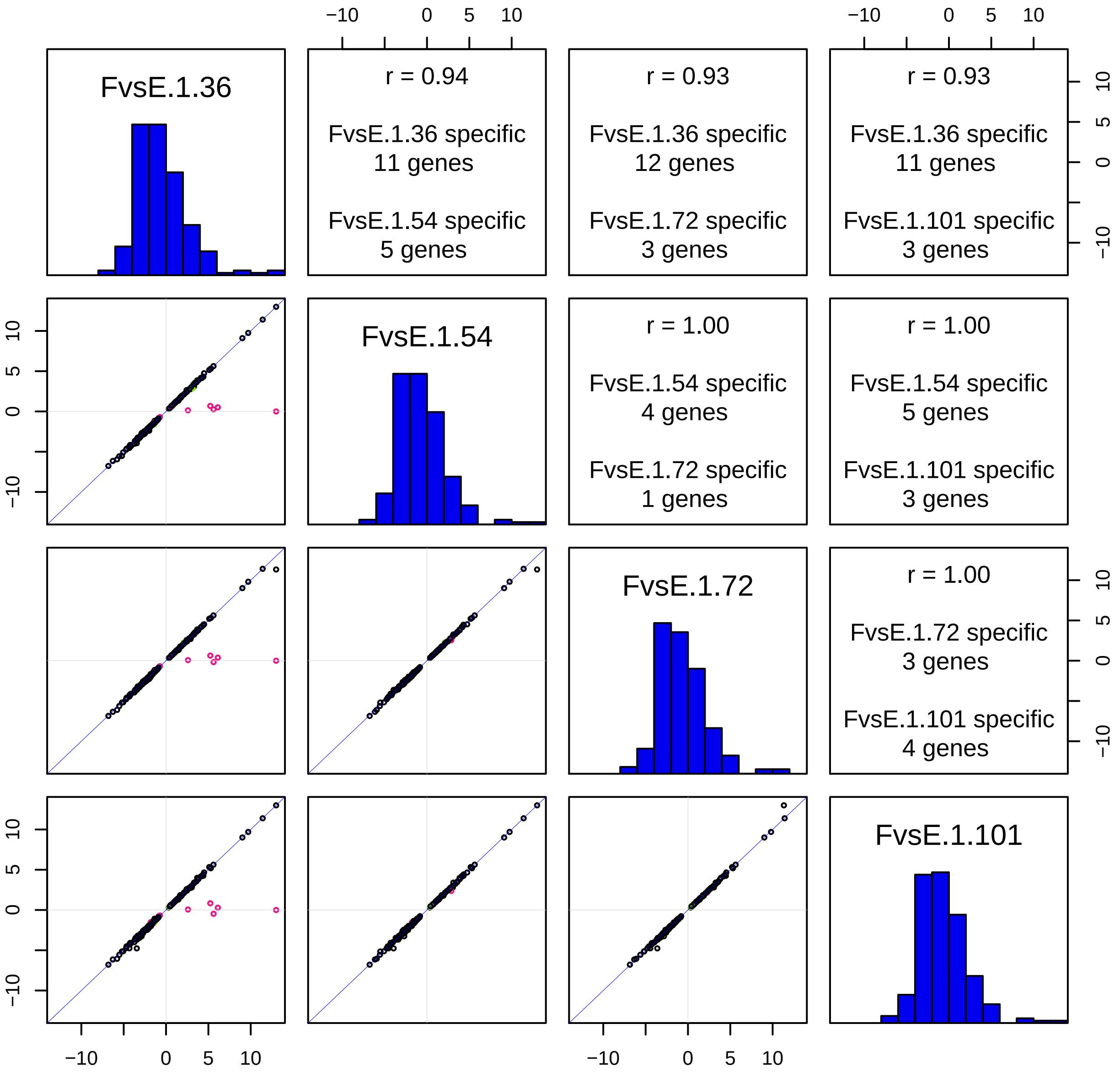

FvsE.1.36
r = 0.94
FvsE.1.36 specific
11 genes
FvsE.1.54 specific
5 genes
r = 0.93
FvsE.1.36 specific
12 genes
FvsE.1.72 specific
3 genes
r = 0.93
FvsE.1.36 specific
11 genes
FvsE.1.101 specific
3 genes
FvsE.1.54
r = 1.00
FvsE.1.54 specific
4 genes
FvsE.1.72 specific
1 genes
r = 1.00
FvsE.1.54 specific
5 genes
FvsE.1.101 specific
3 genes
FvsE.1.72
r = 1.00
FvsE.1.72 specific
3 genes
FvsE.1.101 specific
4 genes
FvsE.1.101
−10
0
5
10

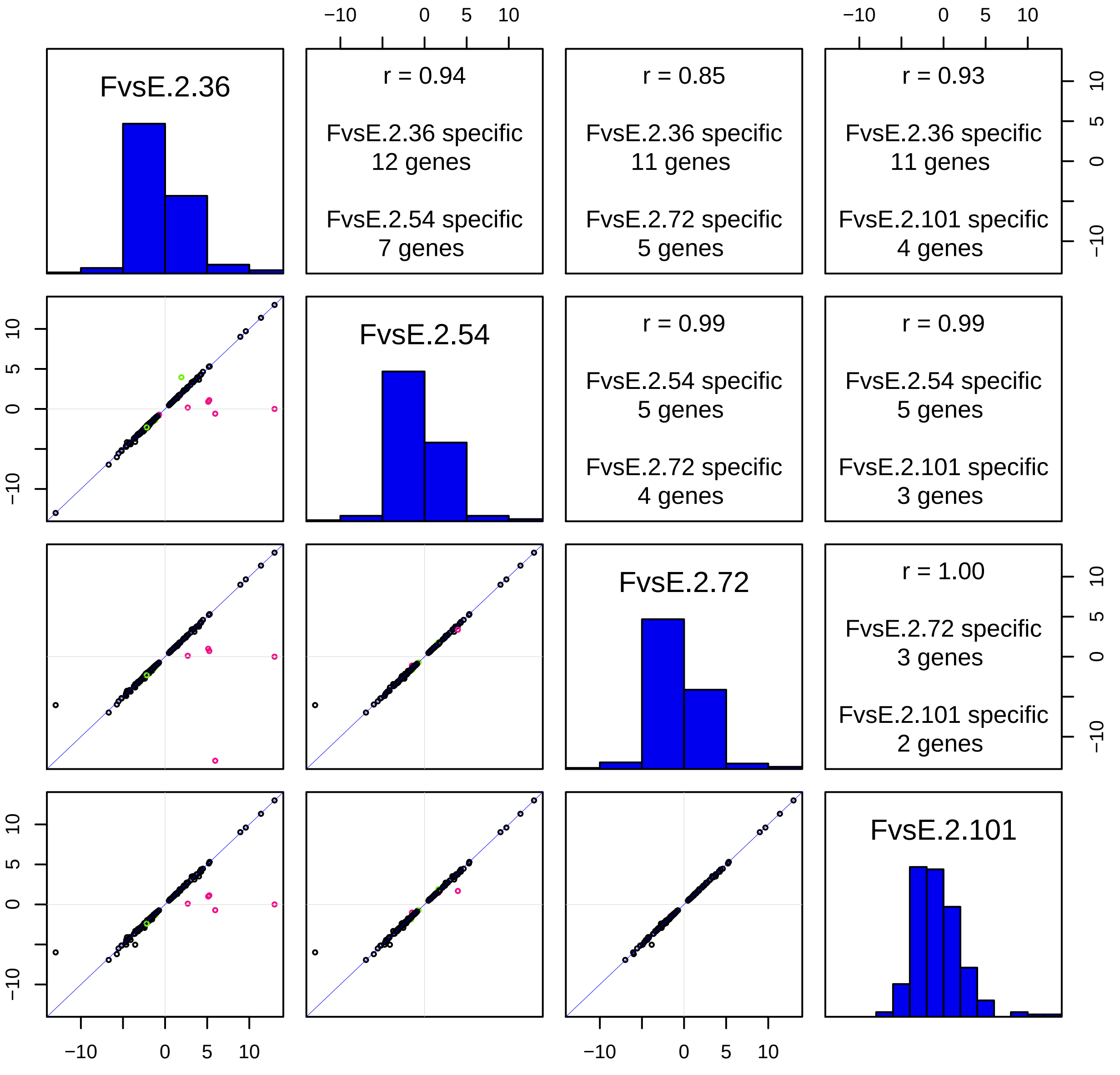
FvsE.2.36
r = 0.94
FvsE.2.36 specific
12 genes
FvsE.2.54 specific
7 genes
r = 0.85
FvsE.2.36 specific
11 genes
FvsE.2.72 specific
5 genes
r = 0.93
FvsE.2.36 specific
11 genes
FvsE.2.101 specific
4 genes
FvsE.2.54
r = 0.99
FvsE.2.54 specific
5 genes
FvsE.2.72 specific
4 genes
r = 0.99
FvsE.2.54 specific
5 genes
FvsE.2.101 specific
3 genes
FvsE.2.72
r = 1.00
FvsE.2.72 specific
3 genes
FvsE.2.101 specific
2 genes
FvsE.2.101
−10
0
5
10

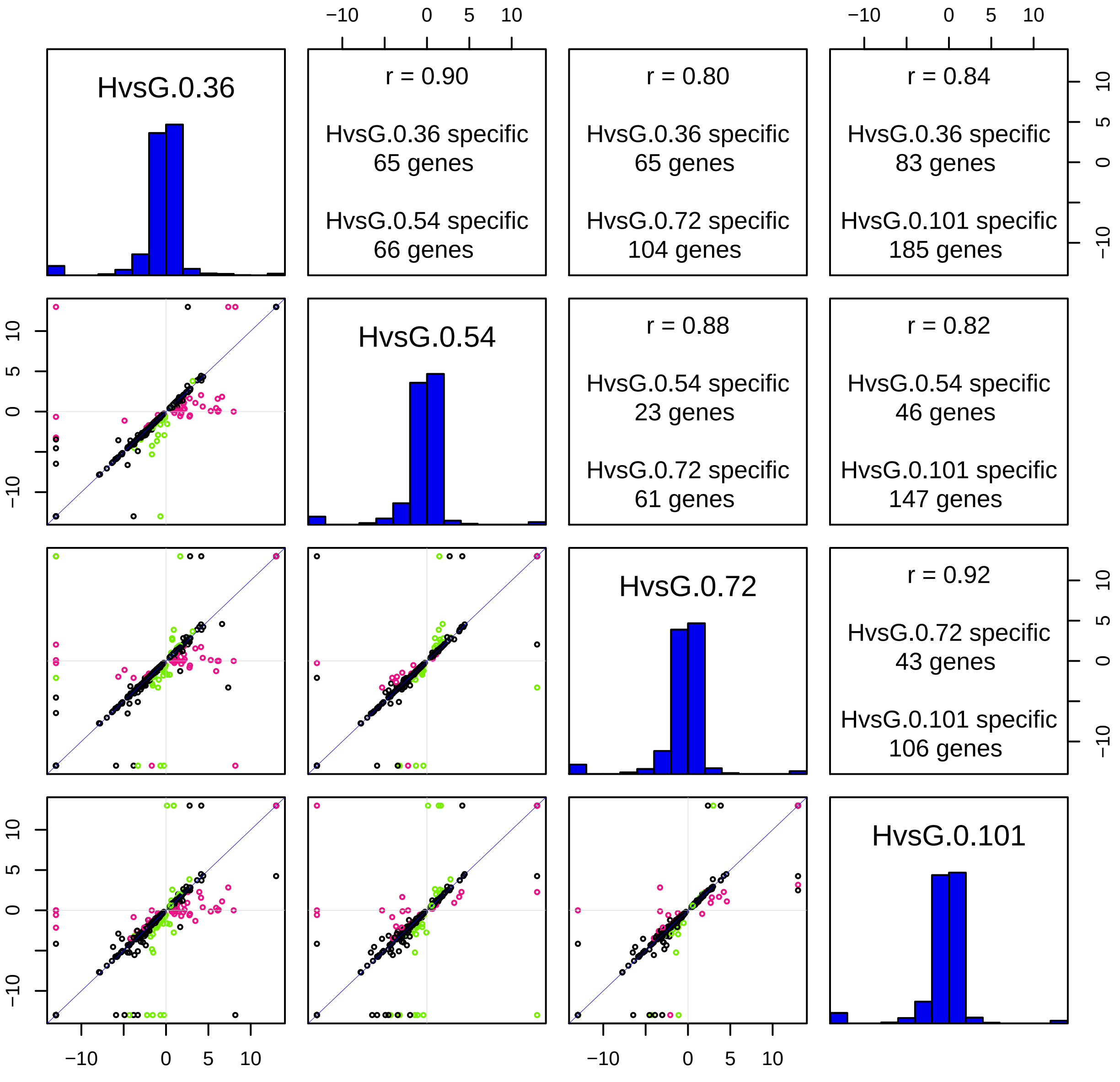
HvsG.0.36
r = 0.90
HvsG.0.36 specific
65 genes
HvsG.0.54 specific
66 genes
r = 0.80
HvsG.0.36 specific
65 genes
HvsG.0.72 specific
104 genes
r = 0.84
HvsG.0.36 specific
83 genes
HvsG.0.101 specific
185 genes
HvsG.0.54
r = 0.88
HvsG.0.54 specific
23 genes
HvsG.0.72 specific
61 genes
r = 0.82
HvsG.0.54 specific
46 genes
HvsG.0.101 specific
147 genes
HvsG.0.72
r = 0.92
HvsG.0.72 specific
43 genes
HvsG.0.101 specific
106 genes
HvsG.0.101
−10
0
5
10

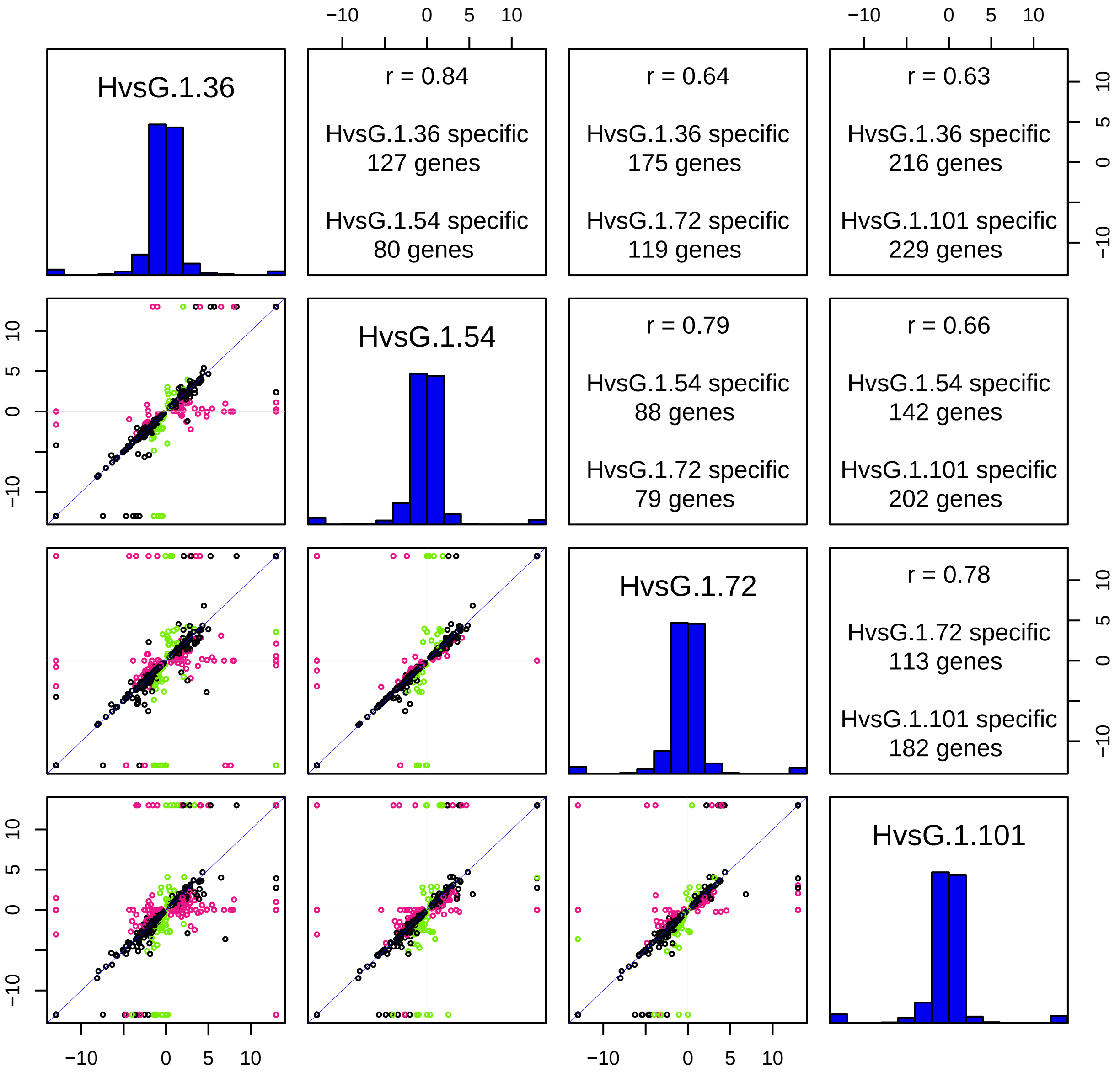
HvsG.1.36
r = 0.84
HvsG.1.36 specific
127 genes
HvsG.1.54 specific
80 genes
r = 0.64
HvsG.1.36 specific
175 genes
HvsG.1.72 specific
119 genes
r = 0.63
HvsG.1.36 specific
216 genes
HvsG.1.101 specific
229 genes
HvsG.1.54
r = 0.79
HvsG.1.54 specific
88 genes
HvsG.1.72 specific
79 genes
r = 0.66
HvsG.1.54 specific
142 genes
HvsG.1.101 specific
202 genes
HvsG.1.72
r = 0.78
HvsG.1.72 specific
113 genes
HvsG.1.101 specific
182 genes
HvsG.1.101
−10
0
5
10

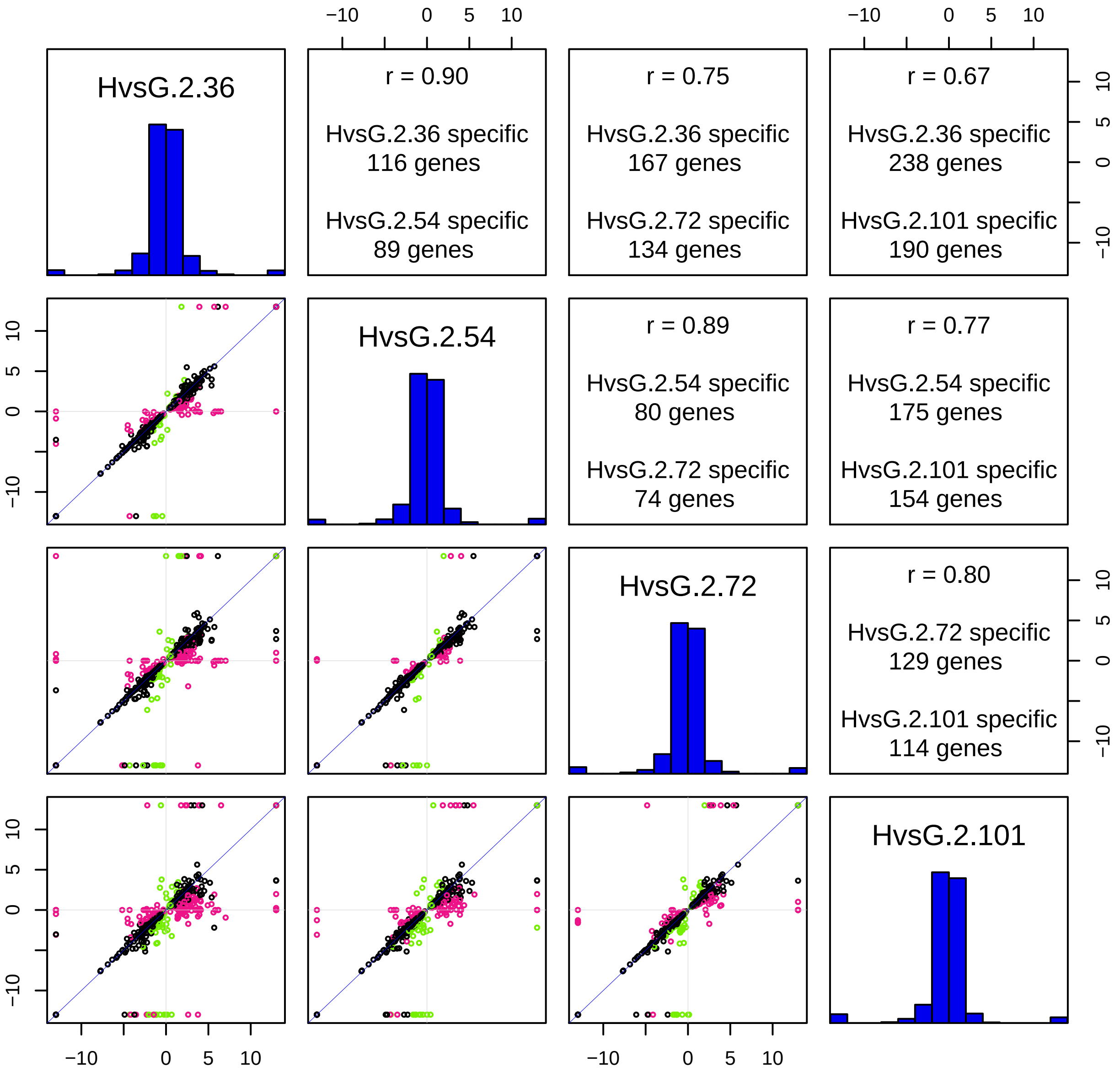

HvsG.2.36
r = 0.90
HvsG.2.36 specific
116 genes
HvsG.2.54 specific
89 genes
r = 0.75
HvsG.2.36 specific
167 genes
HvsG.2.72 specific
134 genes
r = 0.67
HvsG.2.36 specific
238 genes
HvsG.2.101 specific
190 genes
HvsG.2.54
r = 0.89
HvsG.2.54 specific
80 genes
HvsG.2.72 specific
74 genes
r = 0.77
HvsG.2.54 specific
175 genes
HvsG.2.101 specific
154 genes
HvsG.2.72
r = 0.80
HvsG.2.72 specific
129 genes
HvsG.2.101 specific
114 genes
HvsG.2.101
−10
0
5
10

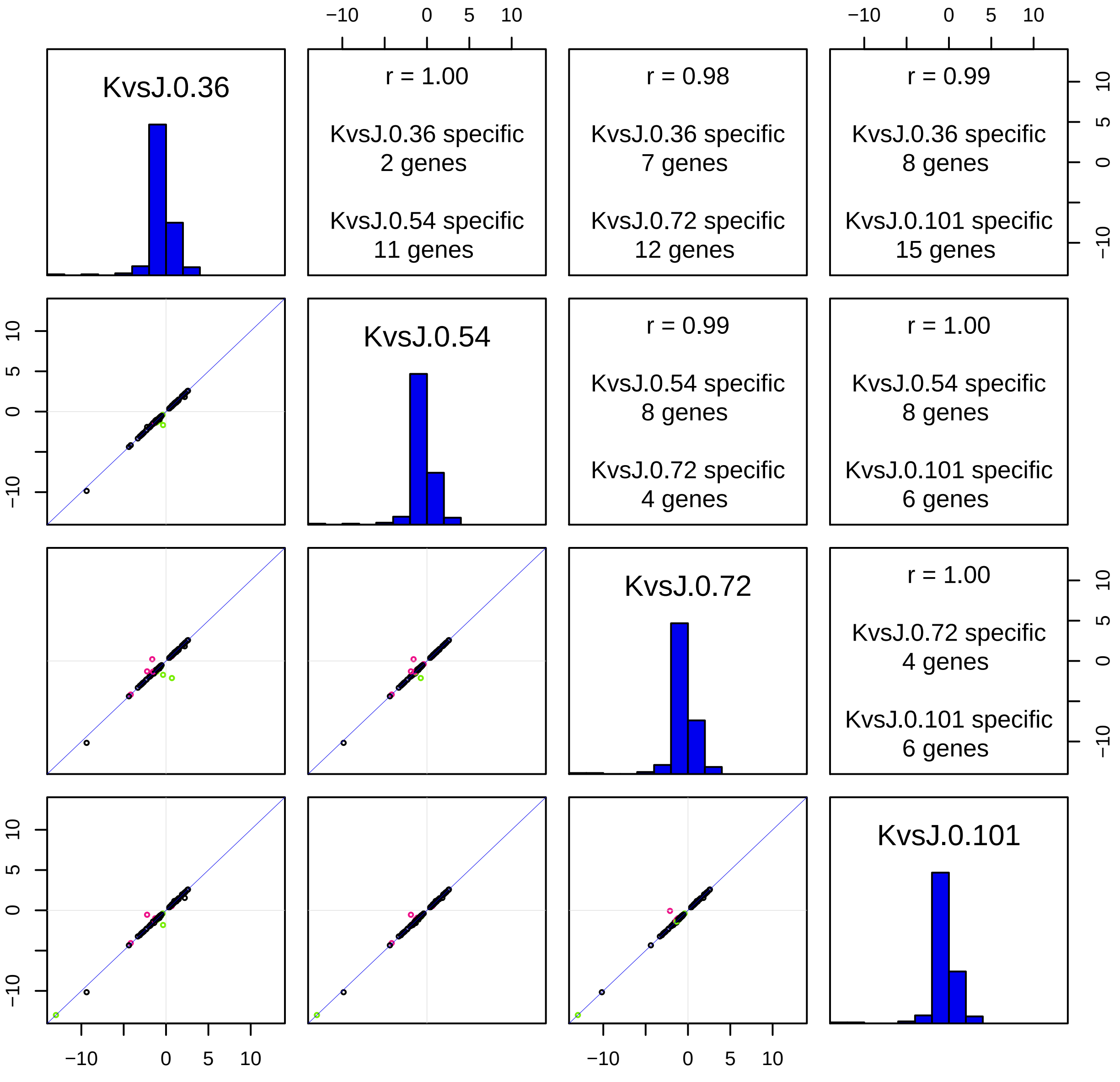
KvsJ.0.36
KvsJ.0.54
KvsJ.0.72
KvsJ.0.101
r = 1.00
KvsJ.0.36 specific
2 genes
KvsJ.0.54 specific
11 genes
r = 0.98
KvsJ.0.36 specific
7 genes
KvsJ.0.72 specific
12 genes
r = 0.99
KvsJ.0.36 specific
8 genes
KvsJ.0.101 specific
15 genes
r = 0.99
KvsJ.0.54 specific
8 genes
KvsJ.0.72 specific
4 genes
r = 1.00
KvsJ.0.54 specific
8 genes
KvsJ.0.101 specific
6 genes
r = 1.00
KvsJ.0.72 specific
4 genes
KvsJ.0.101 specific
6 genes
−10
0
5
10

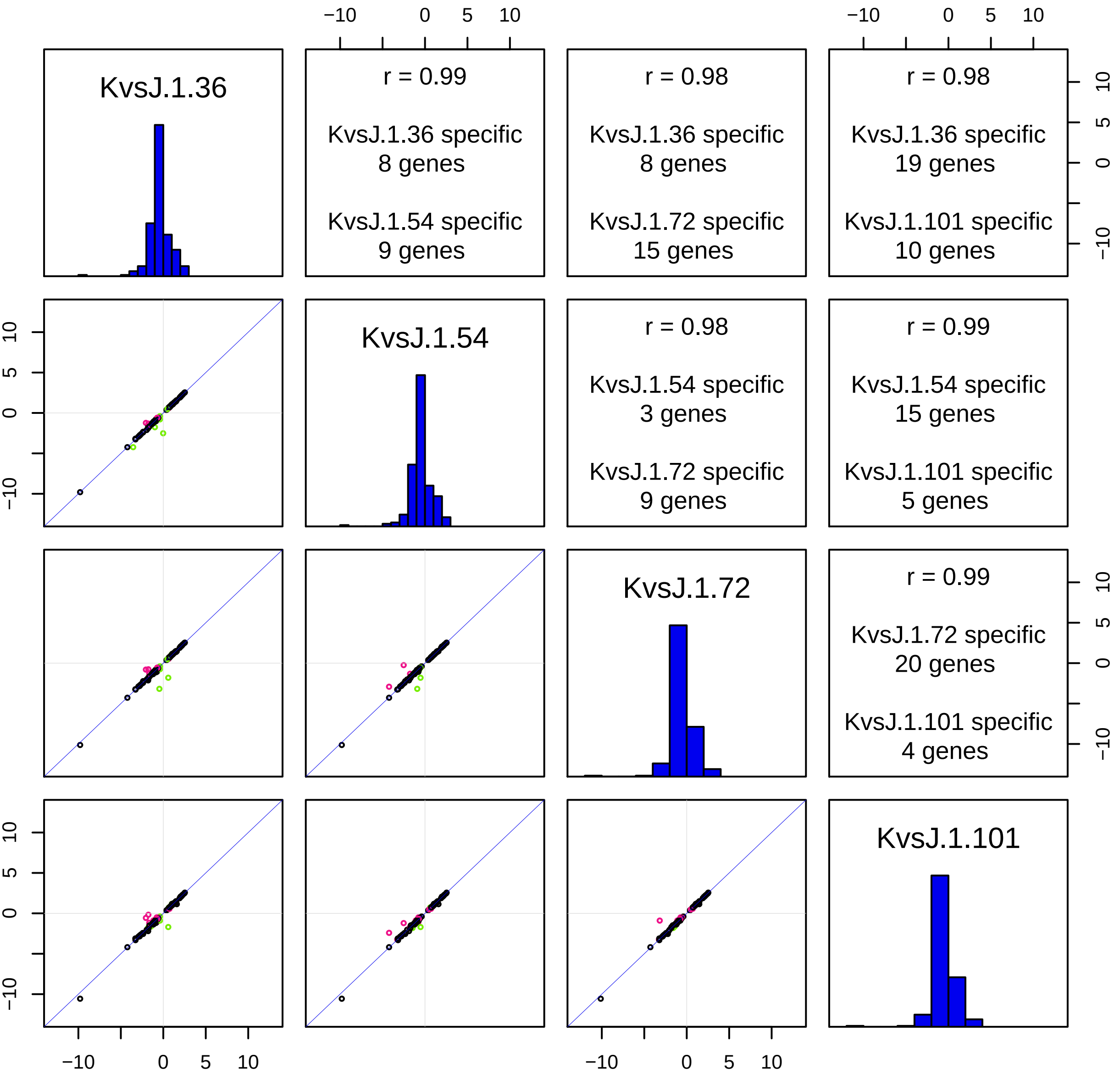
KvsJ.1.36
r = 0.99
KvsJ.1.36 specific
8 genes
KvsJ.1.54 specific
9 genes
r = 0.98
KvsJ.1.36 specific
8 genes
KvsJ.1.72 specific
15 genes
r = 0.98
KvsJ.1.36 specific
19 genes
KvsJ.1.101 specific
10 genes
KvsJ.1.54
r = 0.98
KvsJ.1.54 specific
3 genes
KvsJ.1.72 specific
9 genes
r = 0.99
KvsJ.1.54 specific
15 genes
KvsJ.1.101 specific
5 genes
KvsJ.1.72
r = 0.99
KvsJ.1.72 specific
20 genes
KvsJ.1.101 specific
4 genes
KvsJ.1.101
−10
0
5
10

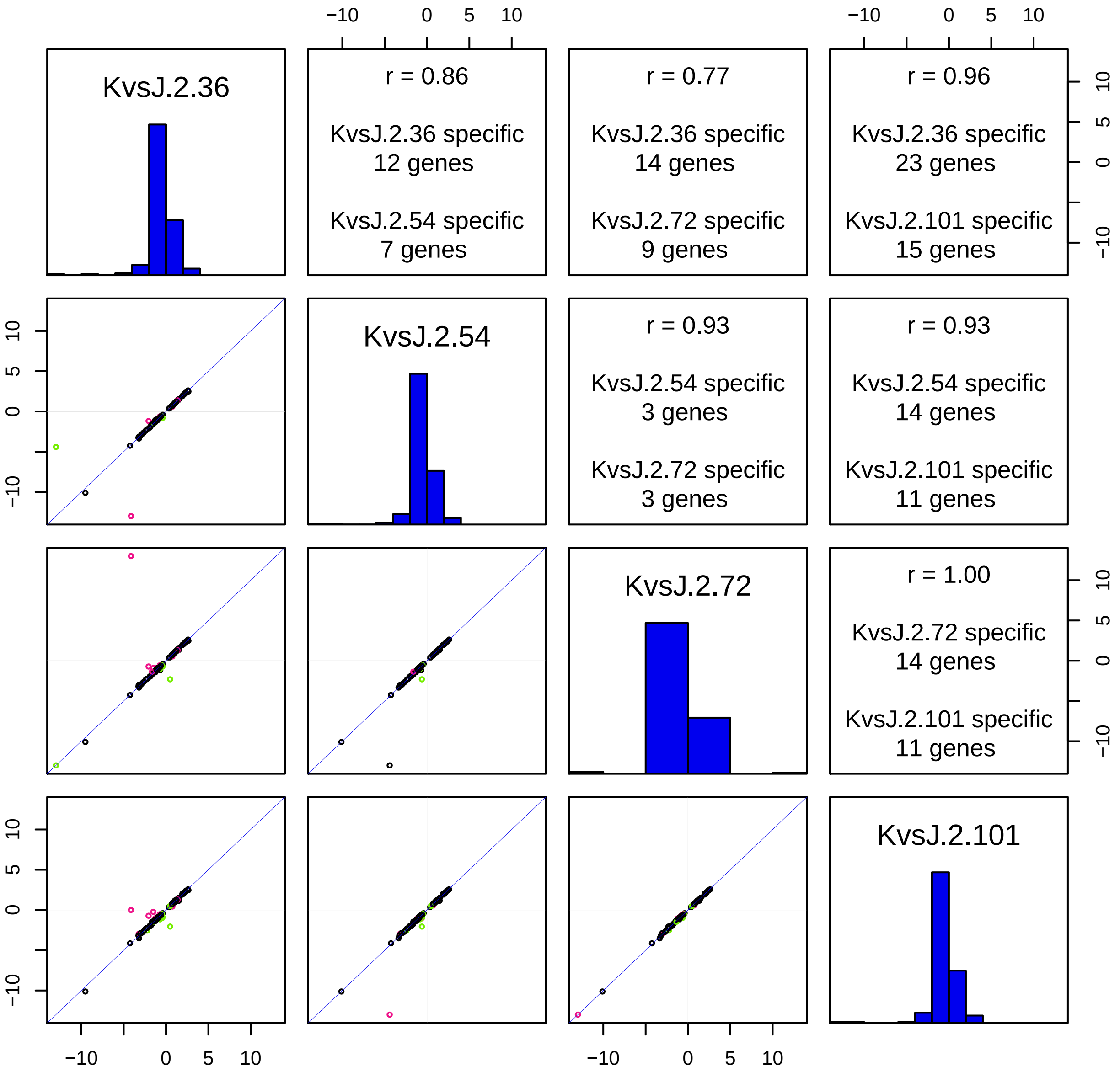

KvsJ.2.36
r = 0.86
KvsJ.2.36 specific
12 genes
KvsJ.2.54 specific
7 genes
r = 0.77
KvsJ.2.36 specific
14 genes
KvsJ.2.72 specific
9 genes
r = 0.96
KvsJ.2.36 specific
23 genes
KvsJ.2.101 specific
15 genes
KvsJ.2.54
r = 0.93
KvsJ.2.54 specific
3 genes
KvsJ.2.72 specific
3 genes
r = 0.93
KvsJ.2.54 specific
14 genes
KvsJ.2.101 specific
11 genes
KvsJ.2.72
r = 1.00
KvsJ.2.72 specific
14 genes
KvsJ.2.101 specific
11 genes
KvsJ.2.101
−10
0
5
10

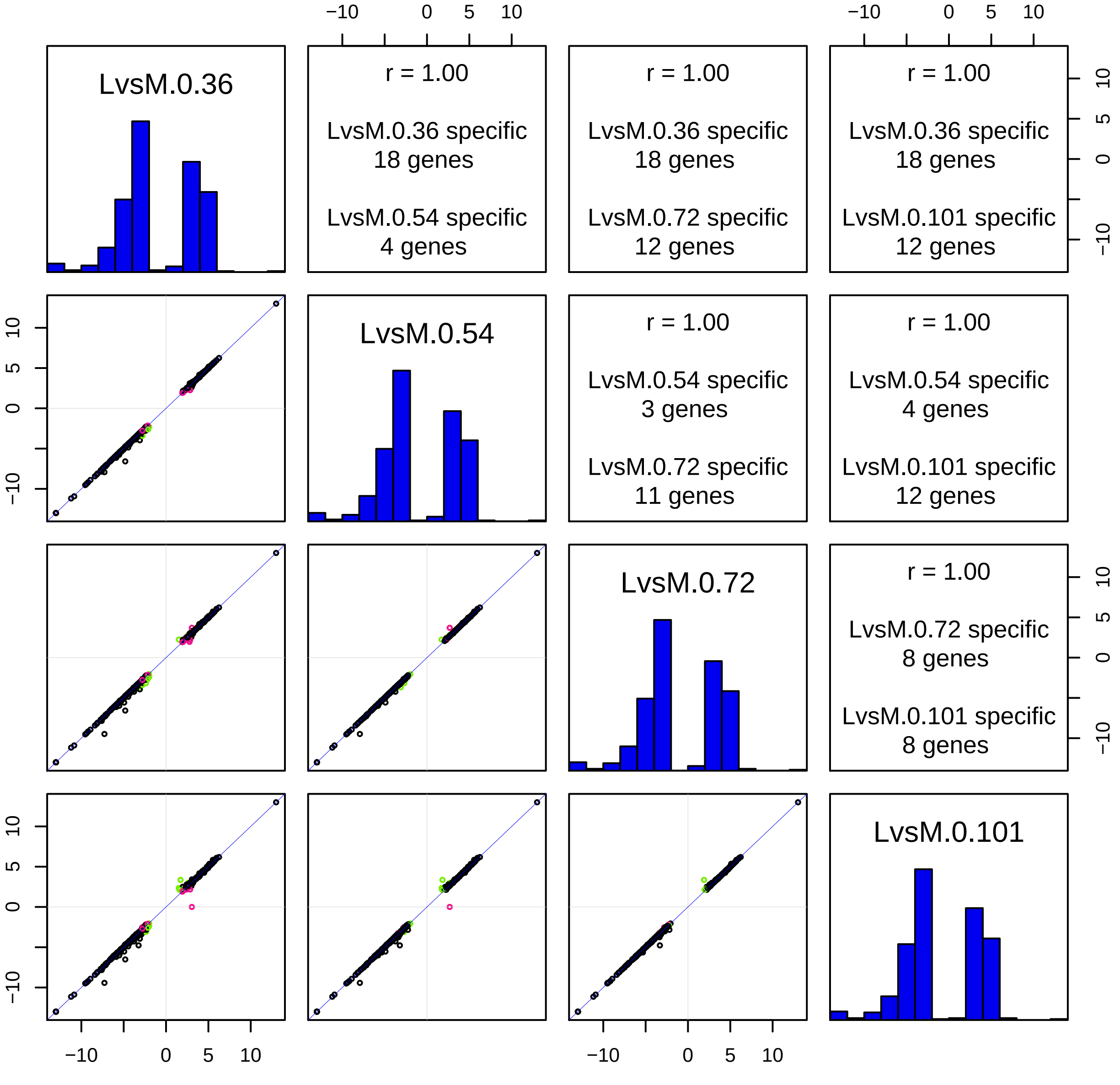
LvsM.0.36
r = 1.00
LvsM.0.36 specific
18 genes
LvsM.0.54 specific
4 genes
r = 1.00
LvsM.0.36 specific
18 genes
LvsM.0.72 specific
12 genes
r = 1.00
LvsM.0.36 specific
18 genes
LvsM.0.101 specific
12 genes
LvsM.0.54
r = 1.00
LvsM.0.54 specific
3 genes
LvsM.0.72 specific
11 genes
r = 1.00
LvsM.0.54 specific
4 genes
LvsM.0.101 specific
12 genes
LvsM.0.72
r = 1.00
LvsM.0.72 specific
8 genes
LvsM.0.101 specific
8 genes
LvsM.0.101
−10
0
5
10

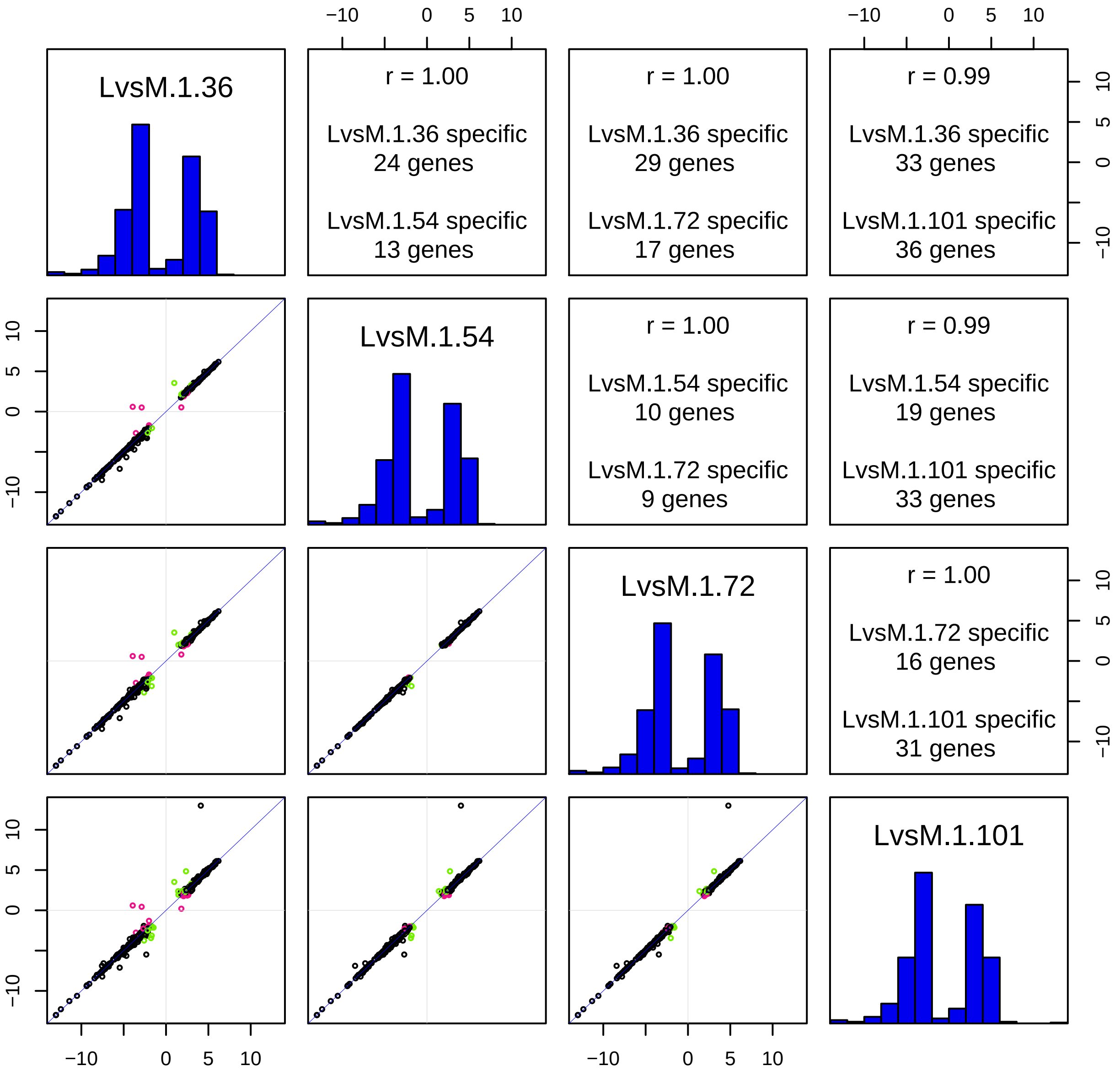
LvsM.1.36
r = 1.00
LvsM.1.36 specific
24 genes
LvsM.1.54 specific
13 genes
r = 1.00
LvsM.1.36 specific
29 genes
LvsM.1.72 specific
17 genes
r = 0.99
LvsM.1.36 specific
33 genes
LvsM.1.101 specific
36 genes
LvsM.1.54
r = 1.00
LvsM.1.54 specific
10 genes
LvsM.1.72 specific
9 genes
r = 0.99
LvsM.1.54 specific
19 genes
LvsM.1.101 specific
33 genes
LvsM.1.72
r = 1.00
LvsM.1.72 specific
16 genes
LvsM.1.101 specific
31 genes
LvsM.1.101
−10
0
5
10

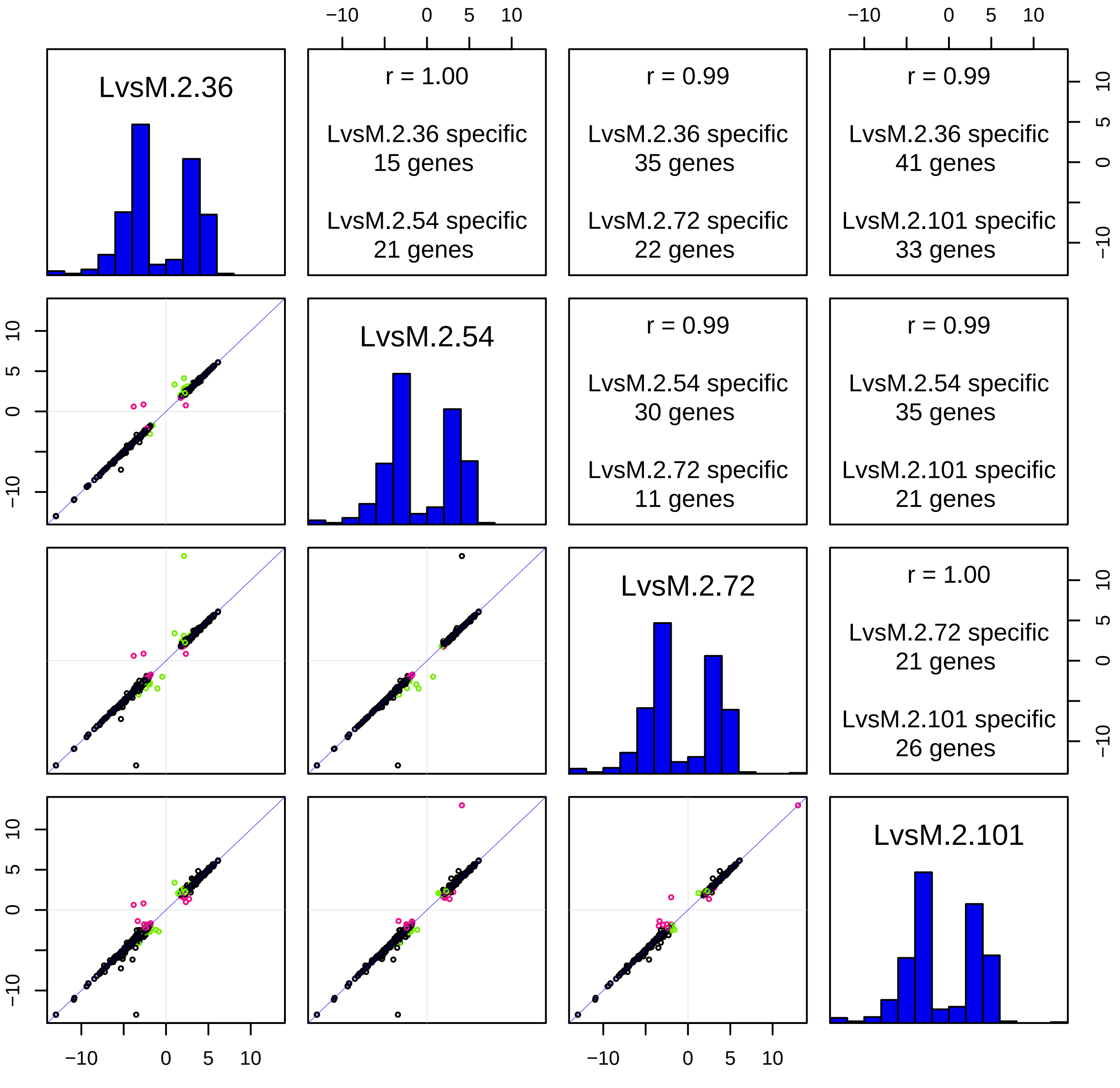
LvsM.2.36
LvsM.2.54
LvsM.2.72
LvsM.2.101
r = 1.00
LvsM.2.36 specific
15 genes
LvsM.2.54 specific
21 genes
r = 0.99
LvsM.2.36 specific
35 genes
LvsM.2.72 specific
22 genes
r = 0.99
LvsM.2.36 specific
41 genes
LvsM.2.101 specific
33 genes
r = 0.99
LvsM.2.54 specific
30 genes
LvsM.2.72 specific
11 genes
r = 0.99
LvsM.2.54 specific
35 genes
LvsM.2.101 specific
21 genes
r = 1.00
LvsM.2.72 specific
21 genes
LvsM.2.101 specific
26 genes
−10
0
5
10

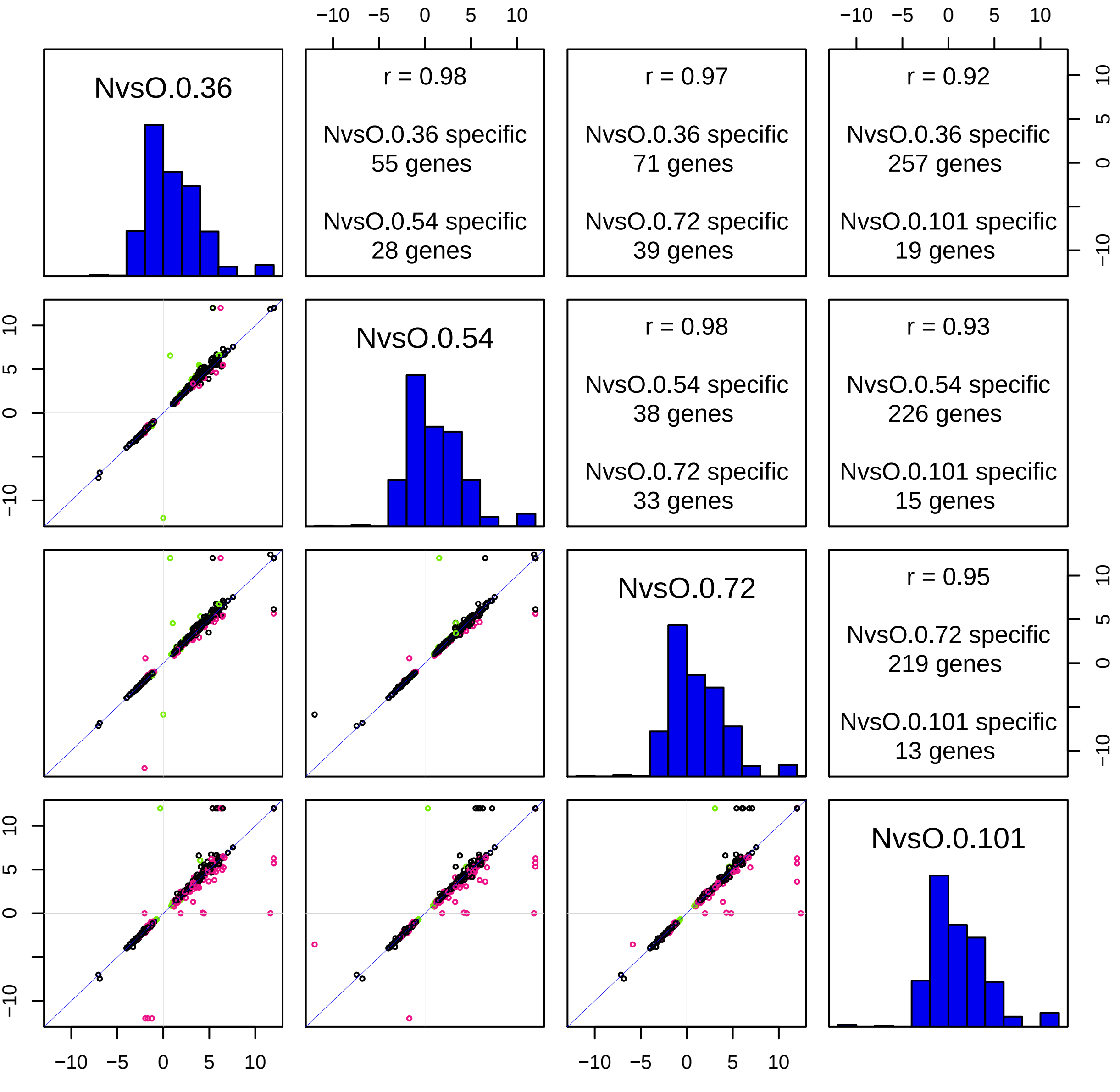

NvsO.0.36
r = 0.98
NvsO.0.36 specific
55 genes
NvsO.0.54 specific
28 genes
r = 0.97
NvsO.0.36 specific
71 genes
NvsO.0.72 specific
39 genes
r = 0.92
NvsO.0.36 specific
257 genes
NvsO.0.101 specific
19 genes
NvsO.0.54
r = 0.98
NvsO.0.54 specific
38 genes
NvsO.0.72 specific
33 genes
r = 0.93
NvsO.0.54 specific
226 genes
NvsO.0.101 specific
15 genes
NvsO.0.72
r = 0.95
NvsO.0.72 specific
219 genes
NvsO.0.101 specific
13 genes
NvsO.0.101
−10
−5
0
5
10

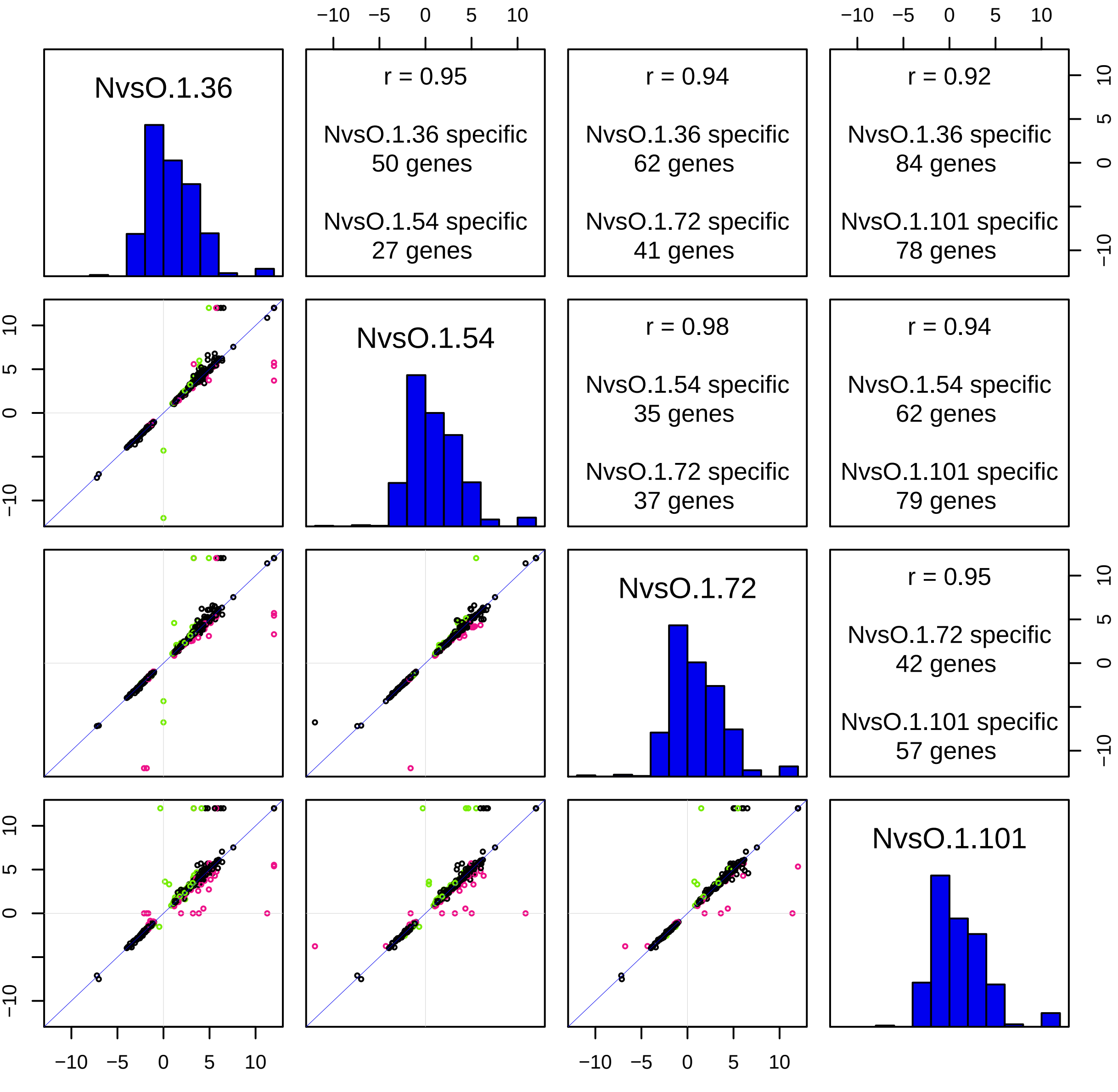

NvsO.1.36
NvsO.1.54
NvsO.1.72
NvsO.1.101
r = 0.95
NvsO.1.36 specific
50 genes
NvsO.1.54 specific
27 genes
r = 0.94
NvsO.1.36 specific
62 genes
NvsO.1.72 specific
41 genes
r = 0.92
NvsO.1.36 specific
84 genes
NvsO.1.101 specific
78 genes
r = 0.98
NvsO.1.54 specific
35 genes
NvsO.1.72 specific
37 genes
r = 0.94
NvsO.1.54 specific
62 genes
NvsO.1.101 specific
79 genes
r = 0.95
NvsO.1.72 specific
42 genes
NvsO.1.101 specific
57 genes
−10 −5 0 5 10

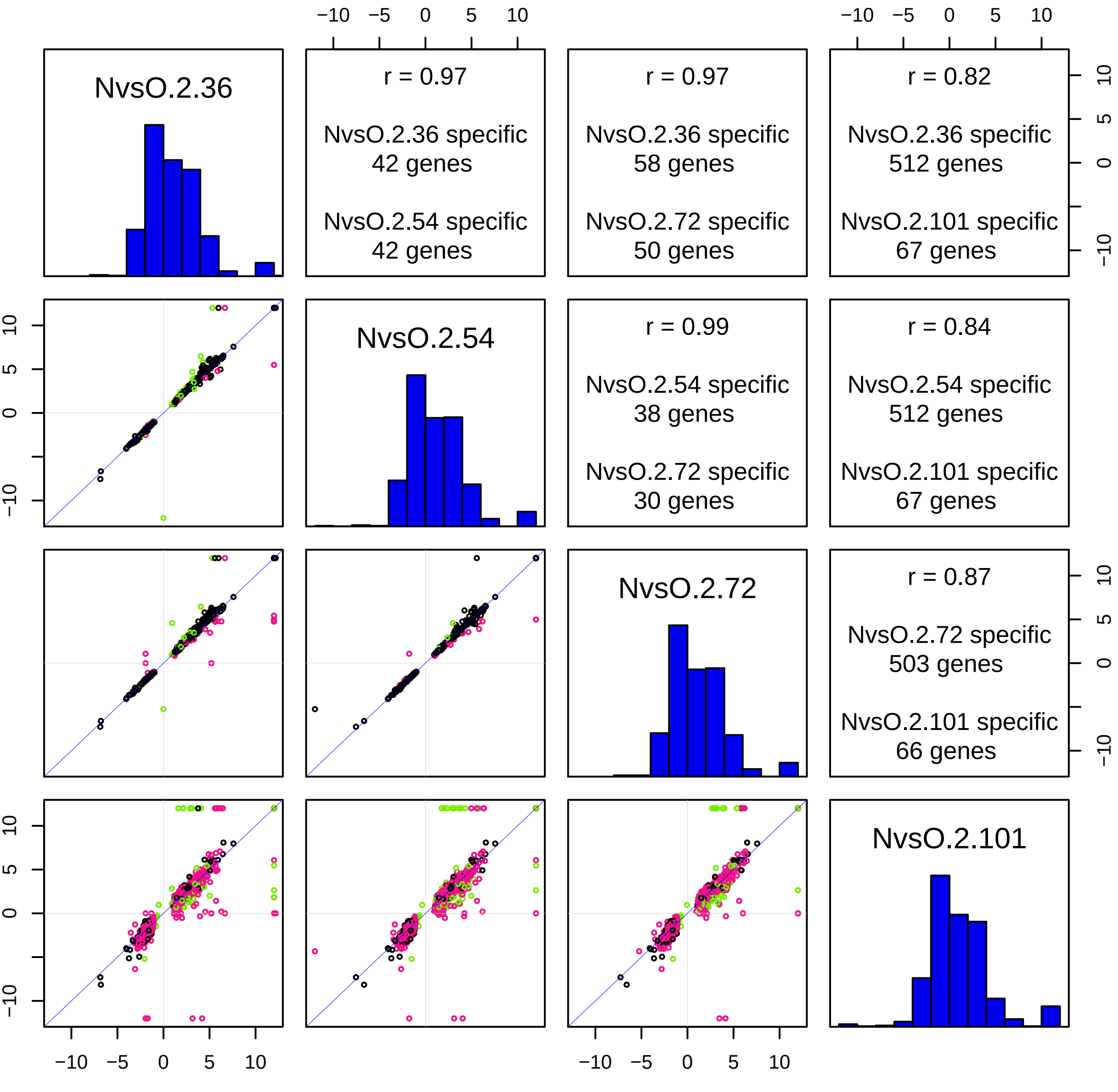
NvsO.2.36
r = 0.97
NvsO.2.36 specific
42 genes
NvsO.2.54 specific
42 genes
r = 0.97
NvsO.2.36 specific
58 genes
NvsO.2.72 specific
50 genes
r = 0.82
NvsO.2.36 specific
512 genes
NvsO.2.101 specific
67 genes
NvsO.2.54
r = 0.99
NvsO.2.54 specific
38 genes
NvsO.2.72 specific
30 genes
r = 0.84
NvsO.2.54 specific
512 genes
NvsO.2.101 specific
67 genes
NvsO.2.72
r = 0.87
NvsO.2.72 specific
503 genes
NvsO.2.101 specific
66 genes
NvsO.2.101
−10
−5
0
5
10

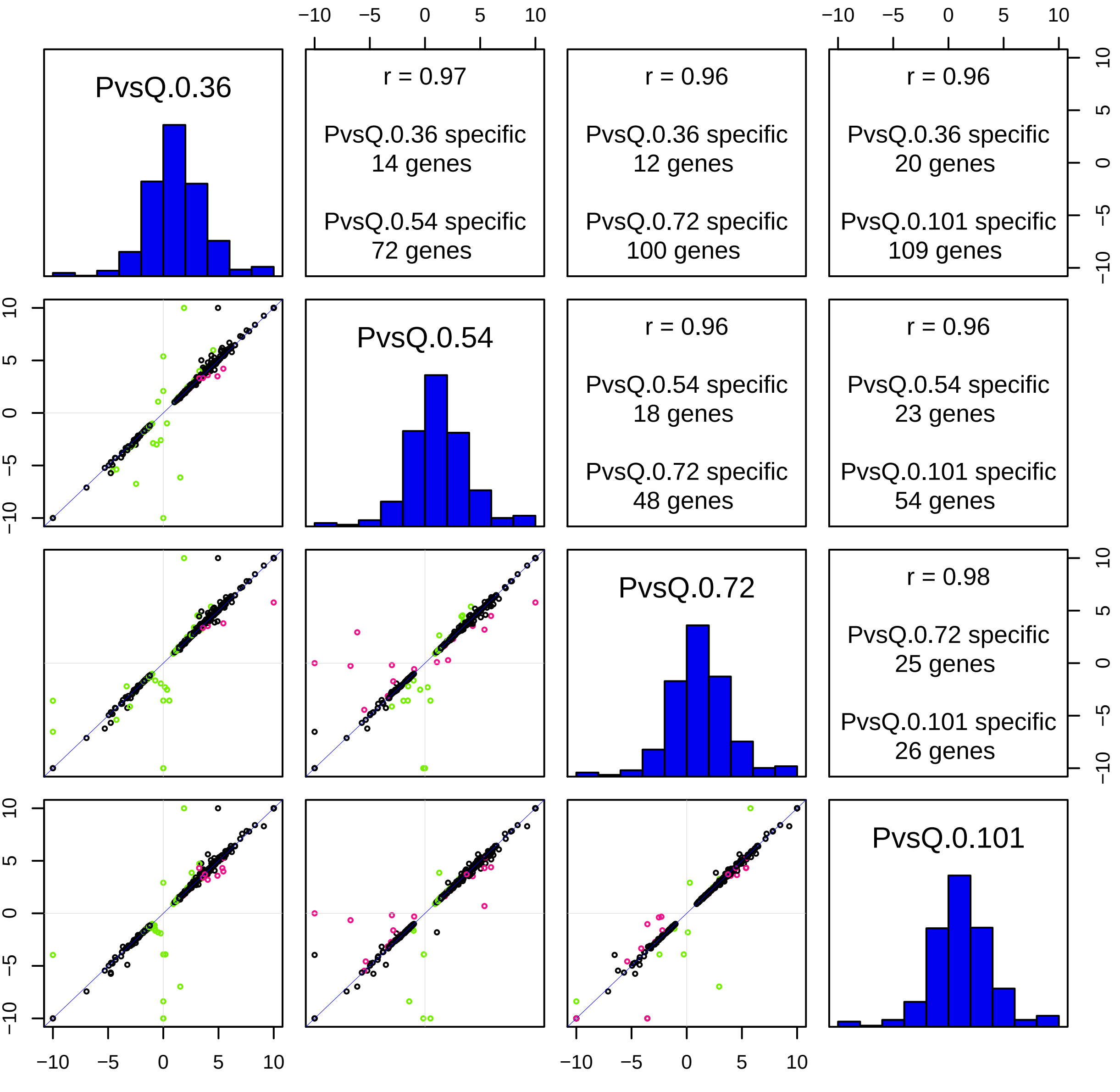

PvsQ.0.36
r = 0.97
PvsQ.0.36 specific
14 genes
PvsQ.0.54 specific
72 genes
r = 0.96
PvsQ.0.36 specific
12 genes
PvsQ.0.72 specific
100 genes
r = 0.96
PvsQ.0.36 specific
20 genes
PvsQ.0.101 specific
109 genes
PvsQ.0.54
r = 0.96
PvsQ.0.54 specific
18 genes
PvsQ.0.72 specific
48 genes
r = 0.96
PvsQ.0.54 specific
23 genes
PvsQ.0.101 specific
54 genes
PvsQ.0.72
r = 0.98
PvsQ.0.72 specific
25 genes
PvsQ.0.101 specific
26 genes
PvsQ.0.101
−10
−5
0
5
10

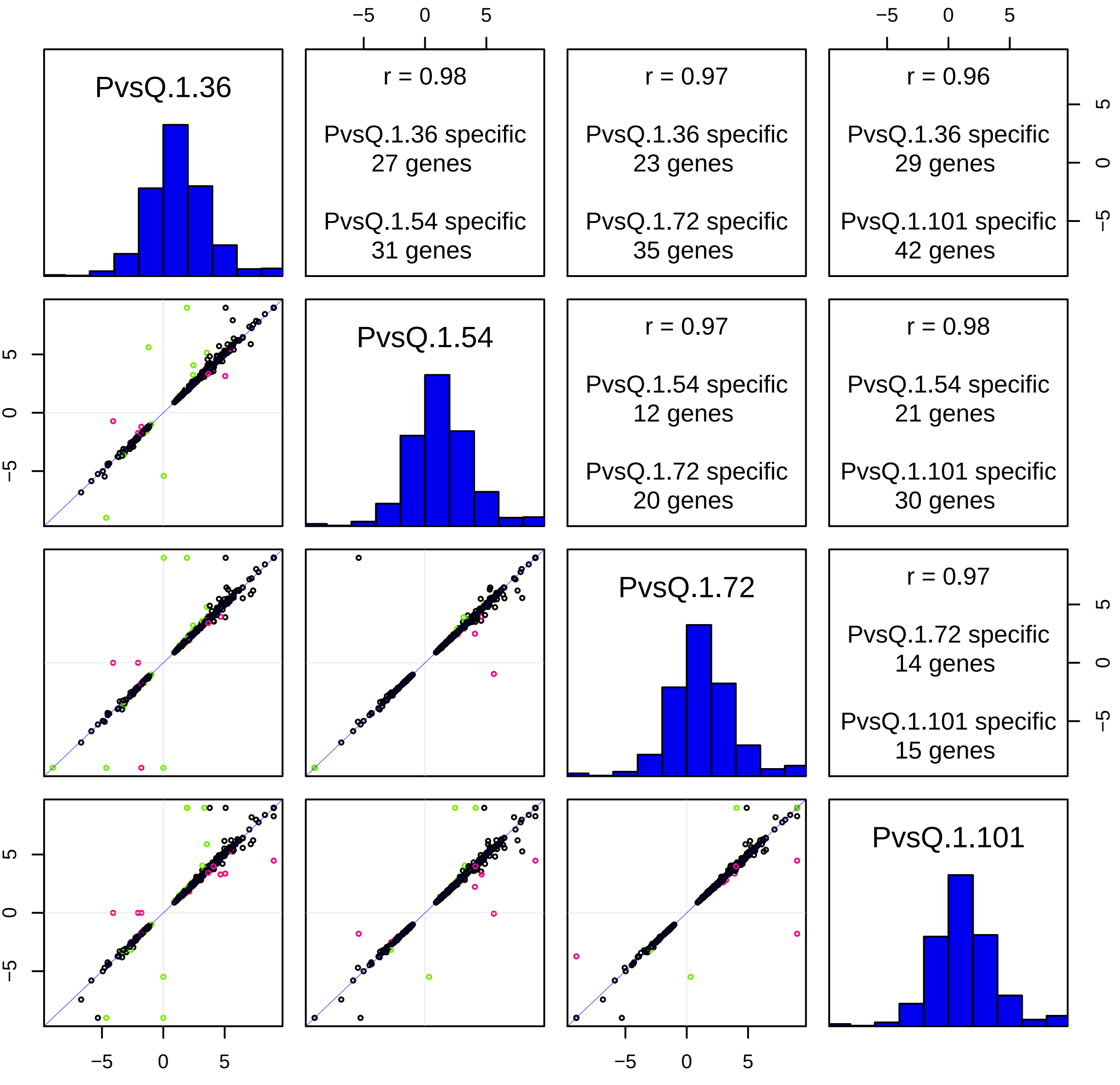
PvsQ.1.36
r = 0.98
PvsQ.1.36 specific
27 genes
PvsQ.1.54 specific
31 genes
r = 0.97
PvsQ.1.36 specific
23 genes
PvsQ.1.72 specific
35 genes
r = 0.96
PvsQ.1.36 specific
29 genes
PvsQ.1.101 specific
42 genes
PvsQ.1.54
r = 0.97
PvsQ.1.54 specific
12 genes
PvsQ.1.72 specific
20 genes
r = 0.98
PvsQ.1.54 specific
21 genes
PvsQ.1.101 specific
30 genes
PvsQ.1.72
r = 0.97
PvsQ.1.72 specific
14 genes
PvsQ.1.101 specific
15 genes
PvsQ.1.101
−5
0
5

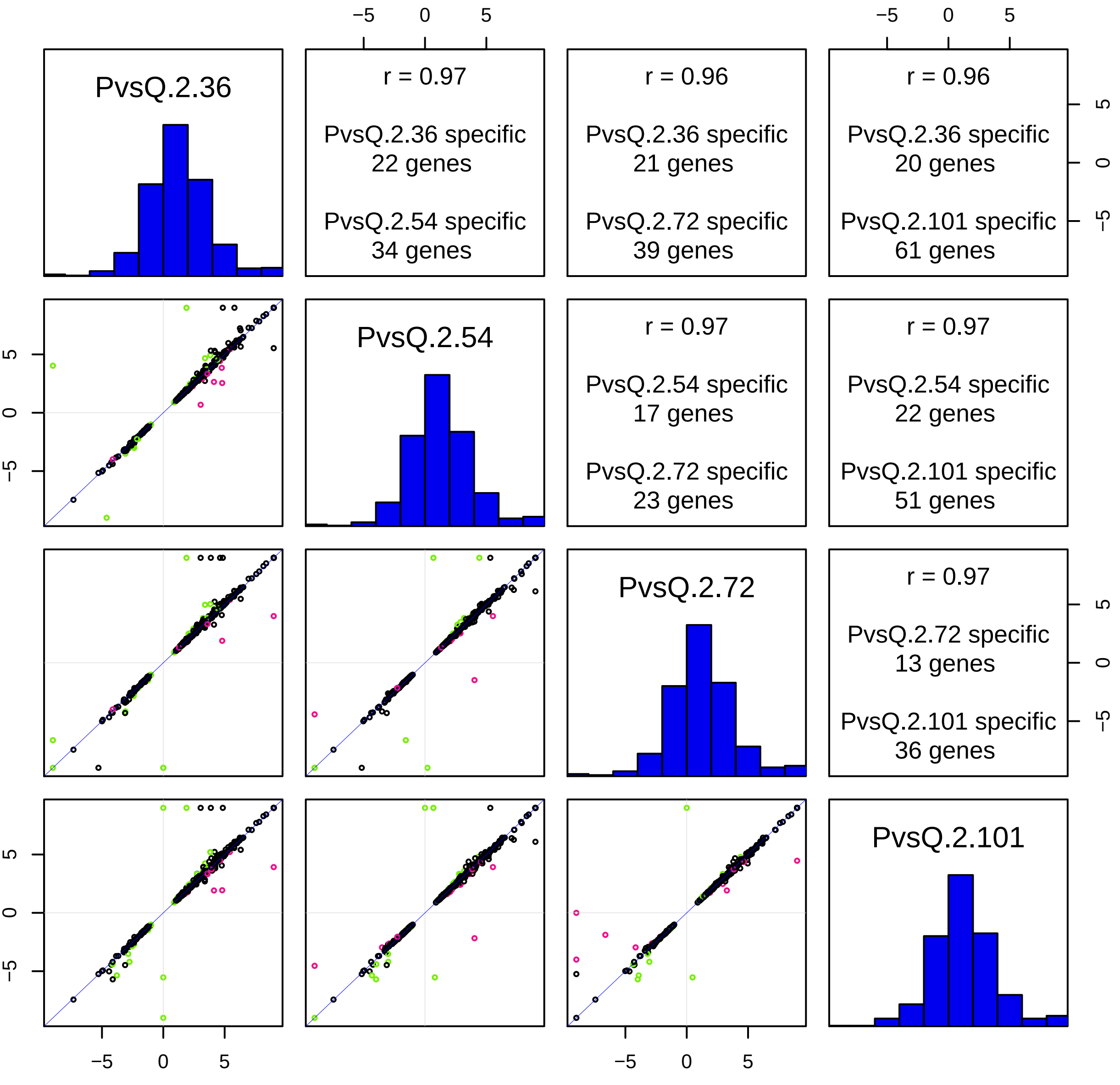
PvsQ.2.36
r = 0.97
PvsQ.2.36 specific
22 genes
PvsQ.2.54 specific
34 genes
r = 0.96
PvsQ.2.36 specific
21 genes
PvsQ.2.72 specific
39 genes
r = 0.96
PvsQ.2.36 specific
20 genes
PvsQ.2.101 specific
61 genes
PvsQ.2.54
r = 0.97
PvsQ.2.54 specific
17 genes
PvsQ.2.72 specific
23 genes
r = 0.97
PvsQ.2.54 specific
22 genes
PvsQ.2.101 specific
51 genes
PvsQ.2.72
r = 0.97
PvsQ.2.72 specific
13 genes
PvsQ.2.101 specific
36 genes
PvsQ.2.101
−5
0
5

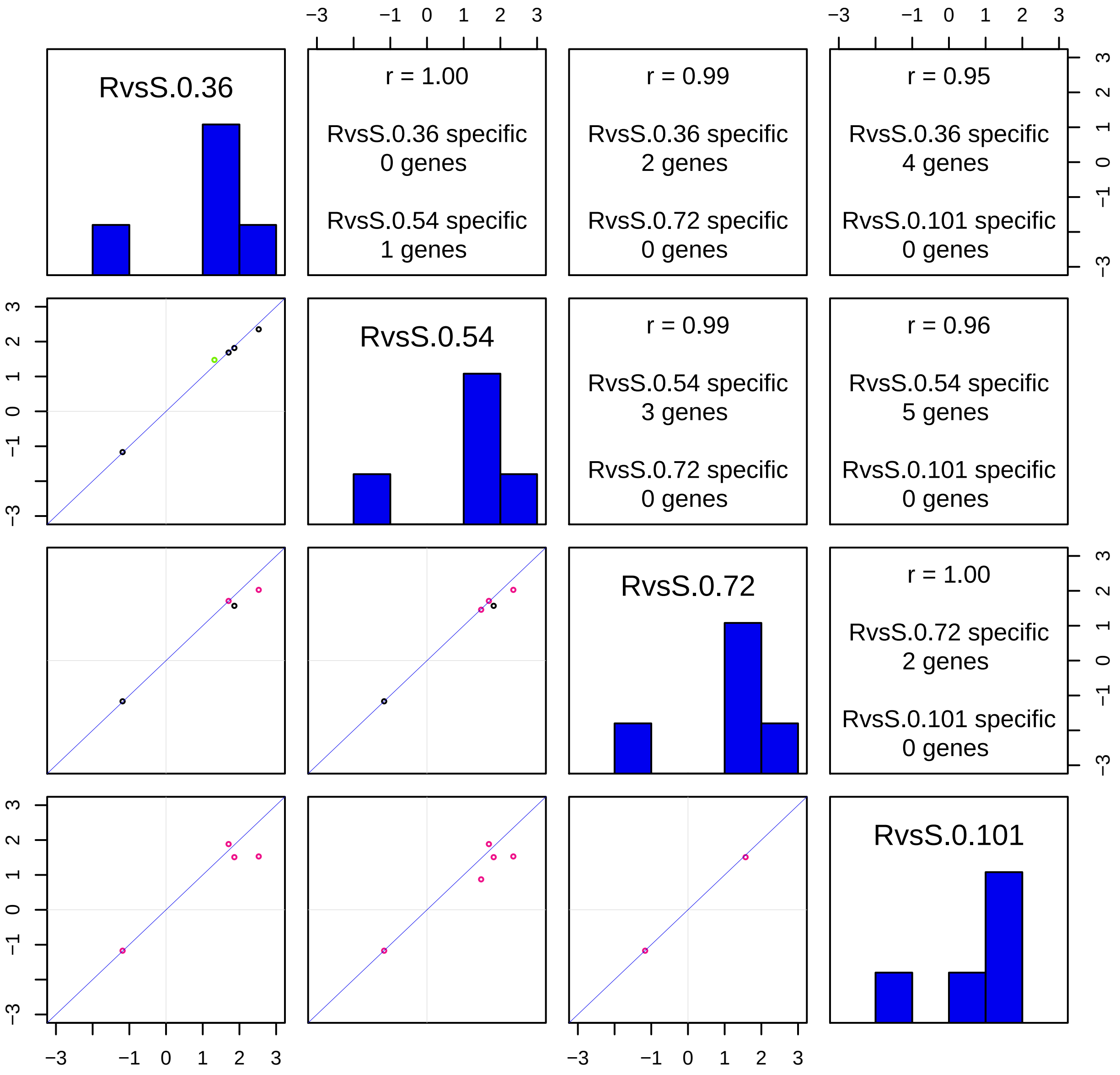
RvsS.0.36
r = 1.00
RvsS.0.36 specific
0 genes
RvsS.0.54 specific
1 genes
r = 0.99
RvsS.0.36 specific
2 genes
RvsS.0.72 specific
0 genes
r = 0.95
RvsS.0.36 specific
4 genes
RvsS.0.101 specific
0 genes
RvsS.0.54
r = 0.99
RvsS.0.54 specific
3 genes
RvsS.0.72 specific
0 genes
r = 0.96
RvsS.0.54 specific
5 genes
RvsS.0.101 specific
0 genes
RvsS.0.72
r = 1.00
RvsS.0.72 specific
2 genes
RvsS.0.101 specific
0 genes
RvsS.0.101
−3 −1 0 1 2 3

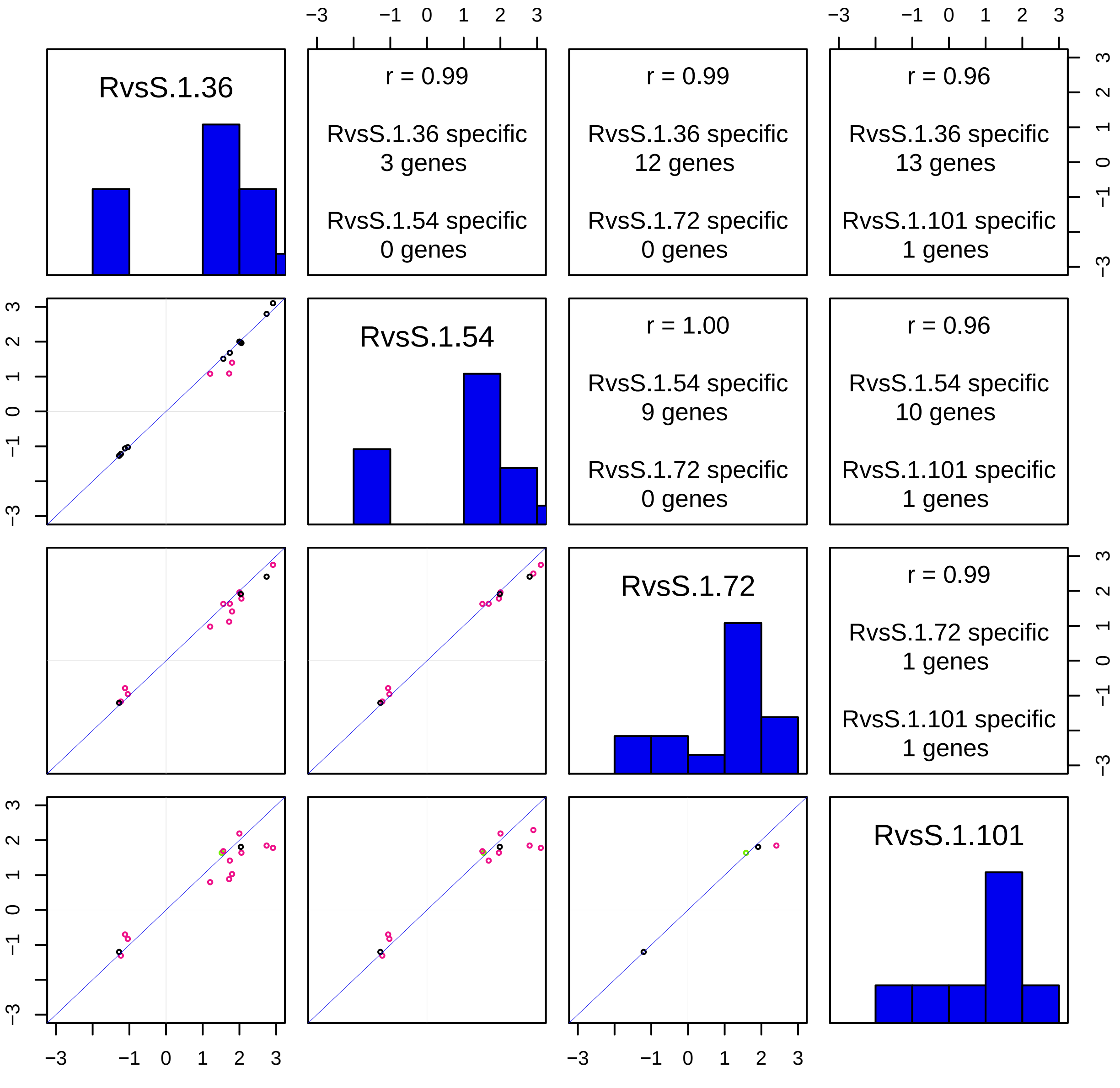
RvsS.1.36
r = 0.99
RvsS.1.36 specific
3 genes
RvsS.1.54 specific
0 genes
r = 0.99
RvsS.1.36 specific
12 genes
RvsS.1.72 specific
0 genes
r = 0.96
RvsS.1.36 specific
13 genes
RvsS.1.101 specific
1 genes
RvsS.1.54
r = 1.00
RvsS.1.54 specific
9 genes
RvsS.1.72 specific
0 genes
r = 0.96
RvsS.1.54 specific
10 genes
RvsS.1.101 specific
1 genes
RvsS.1.72
r = 0.99
RvsS.1.72 specific
1 genes
RvsS.1.101 specific
1 genes
RvsS.1.101
−3 −1 0 1 2 3

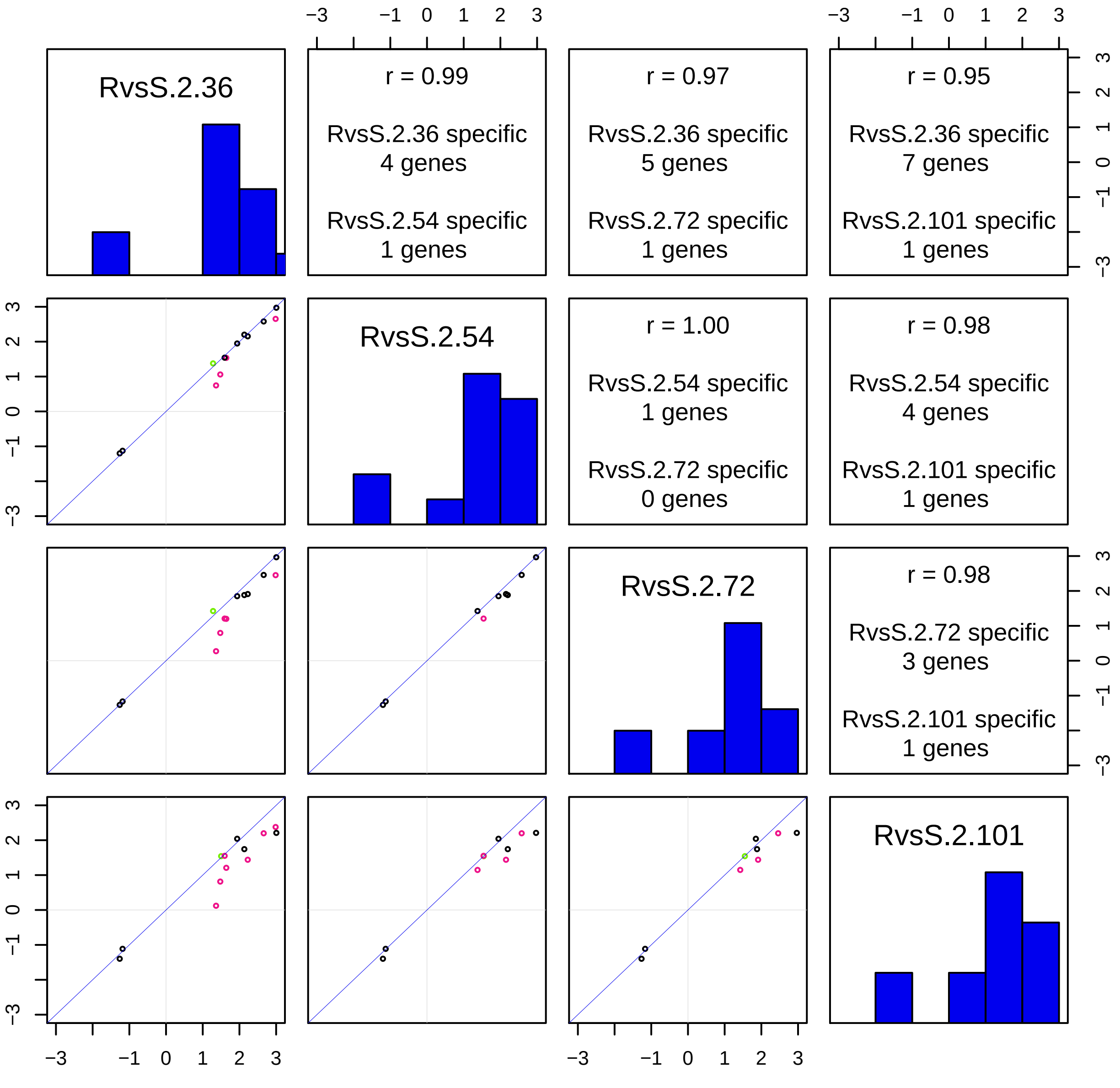

RvsS.2.36
r = 0.99
RvsS.2.36 specific
4 genes
RvsS.2.54 specific
1 genes
r = 0.97
RvsS.2.36 specific
5 genes
RvsS.2.72 specific
1 genes
r = 0.95
RvsS.2.36 specific
7 genes
RvsS.2.101 specific
1 genes
RvsS.2.54
r = 1.00
RvsS.2.54 specific
1 genes
RvsS.2.72 specific
0 genes
r = 0.98
RvsS.2.54 specific
4 genes
RvsS.2.101 specific
1 genes
RvsS.2.72
r = 0.98
RvsS.2.72 specific
3 genes
RvsS.2.101 specific
1 genes
RvsS.2.101
−3 −1 0 1 2 3

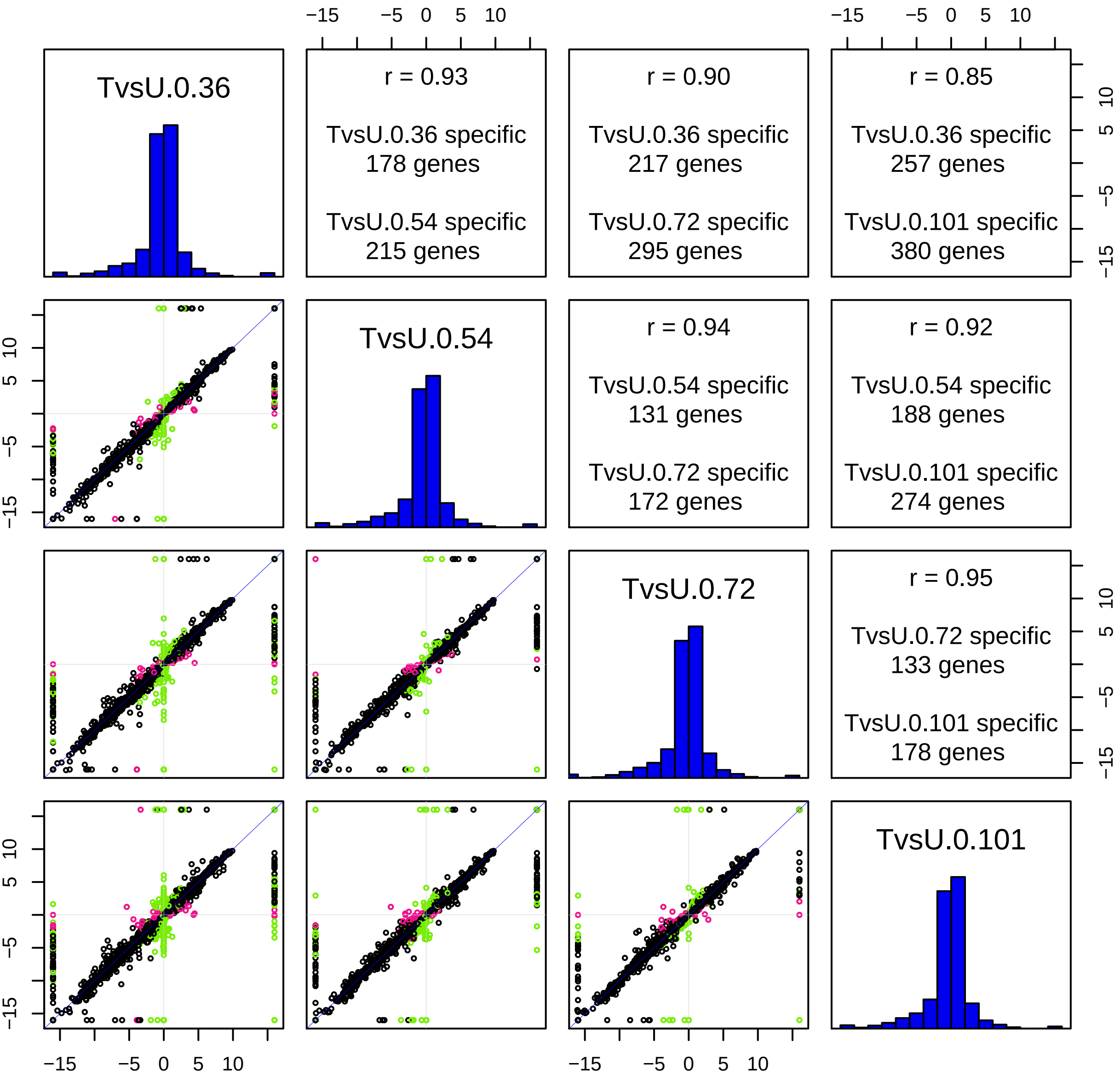
TvsU.0.36
r = 0.93
TvsU.0.36 specific
178 genes
TvsU.0.54 specific
215 genes
r = 0.90
TvsU.0.36 specific
217 genes
TvsU.0.72 specific
295 genes
r = 0.85
TvsU.0.36 specific
257 genes
TvsU.0.101 specific
380 genes
TvsU.0.54
r = 0.94
TvsU.0.54 specific
131 genes
TvsU.0.72 specific
172 genes
r = 0.92
TvsU.0.54 specific
188 genes
TvsU.0.101 specific
274 genes
TvsU.0.72
r = 0.95
TvsU.0.72 specific
133 genes
TvsU.0.101 specific
178 genes
TvsU.0.101
−15
−5
0
5
10

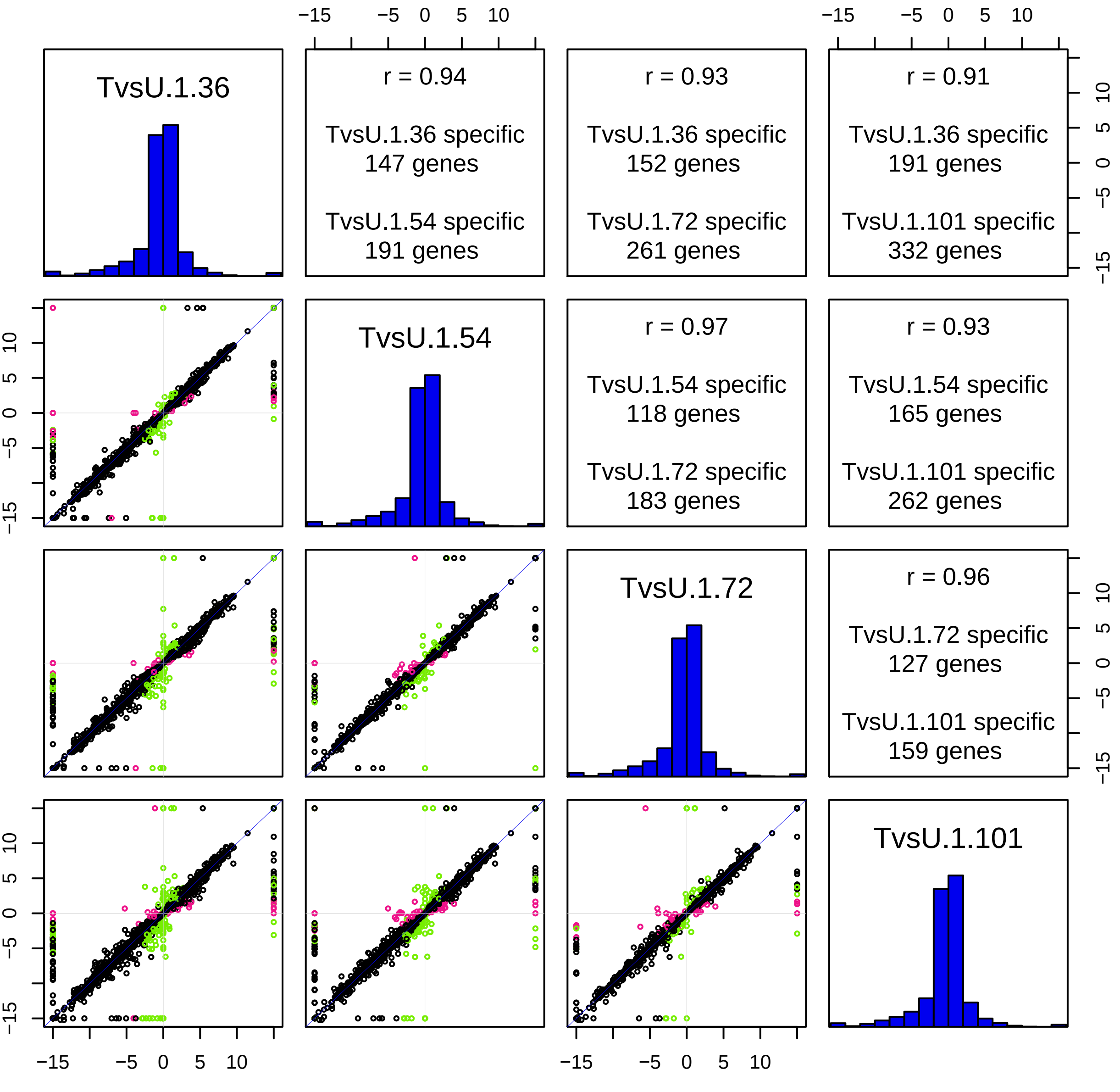

TvsU.1.36
r = 0.94
TvsU.1.36 specific
147 genes
TvsU.1.54 specific
191 genes
r = 0.93
TvsU.1.36 specific
152 genes
TvsU.1.72 specific
261 genes
r = 0.91
TvsU.1.36 specific
191 genes
TvsU.1.101 specific
332 genes
TvsU.1.54
r = 0.97
TvsU.1.54 specific
118 genes
TvsU.1.72 specific
183 genes
r = 0.93
TvsU.1.54 specific
165 genes
TvsU.1.101 specific
262 genes
TvsU.1.72
r = 0.96
TvsU.1.72 specific
127 genes
TvsU.1.101 specific
159 genes
TvsU.1.101
−15
−5
0
5
10

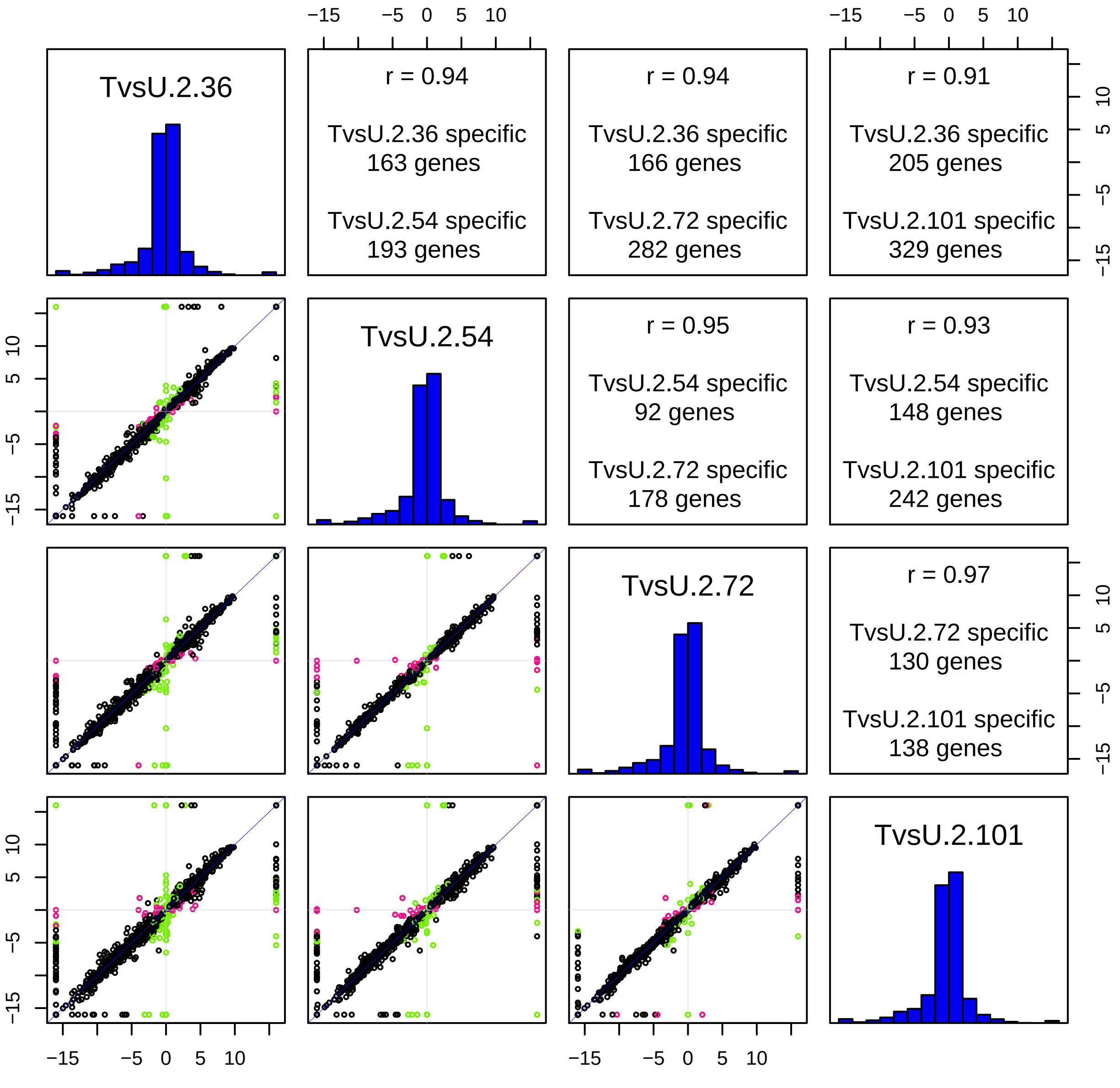

TvsU.2.36
r = 0.94
TvsU.2.36 specific
163 genes
TvsU.2.54 specific
193 genes
r = 0.94
TvsU.2.36 specific
166 genes
TvsU.2.72 specific
282 genes
r = 0.91
TvsU.2.36 specific
205 genes
TvsU.2.101 specific
329 genes
TvsU.2.54
r = 0.95
TvsU.2.54 specific
92 genes
TvsU.2.72 specific
178 genes
r = 0.93
TvsU.2.54 specific
148 genes
TvsU.2.101 specific
242 genes
TvsU.2.72
r = 0.97
TvsU.2.72 specific
130 genes
TvsU.2.101 specific
138 genes
TvsU.2.101
−15
−5
0
5
10

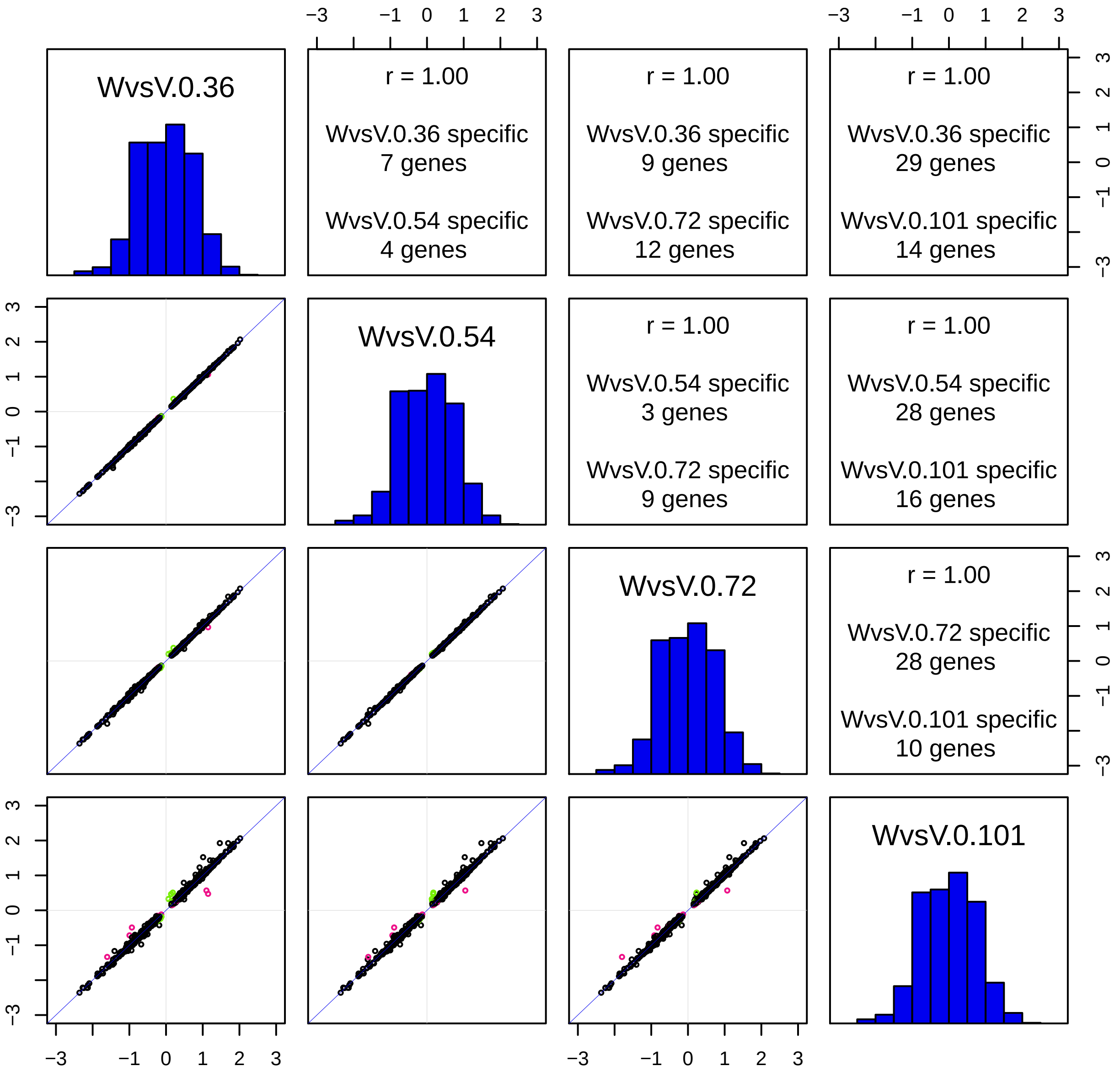

WvsV.0.36
r = 1.00
WvsV.0.36 specific
7 genes
WvsV.0.54 specific
4 genes
r = 1.00
WvsV.0.36 specific
9 genes
WvsV.0.72 specific
12 genes
r = 1.00
WvsV.0.36 specific
29 genes
WvsV.0.101 specific
14 genes
WvsV.0.54
r = 1.00
WvsV.0.54 specific
3 genes
WvsV.0.72 specific
9 genes
r = 1.00
WvsV.0.54 specific
28 genes
WvsV.0.101 specific
16 genes
WvsV.0.72
r = 1.00
WvsV.0.72 specific
28 genes
WvsV.0.101 specific
10 genes
WvsV.0.101
−3 −1 0 1 2 3

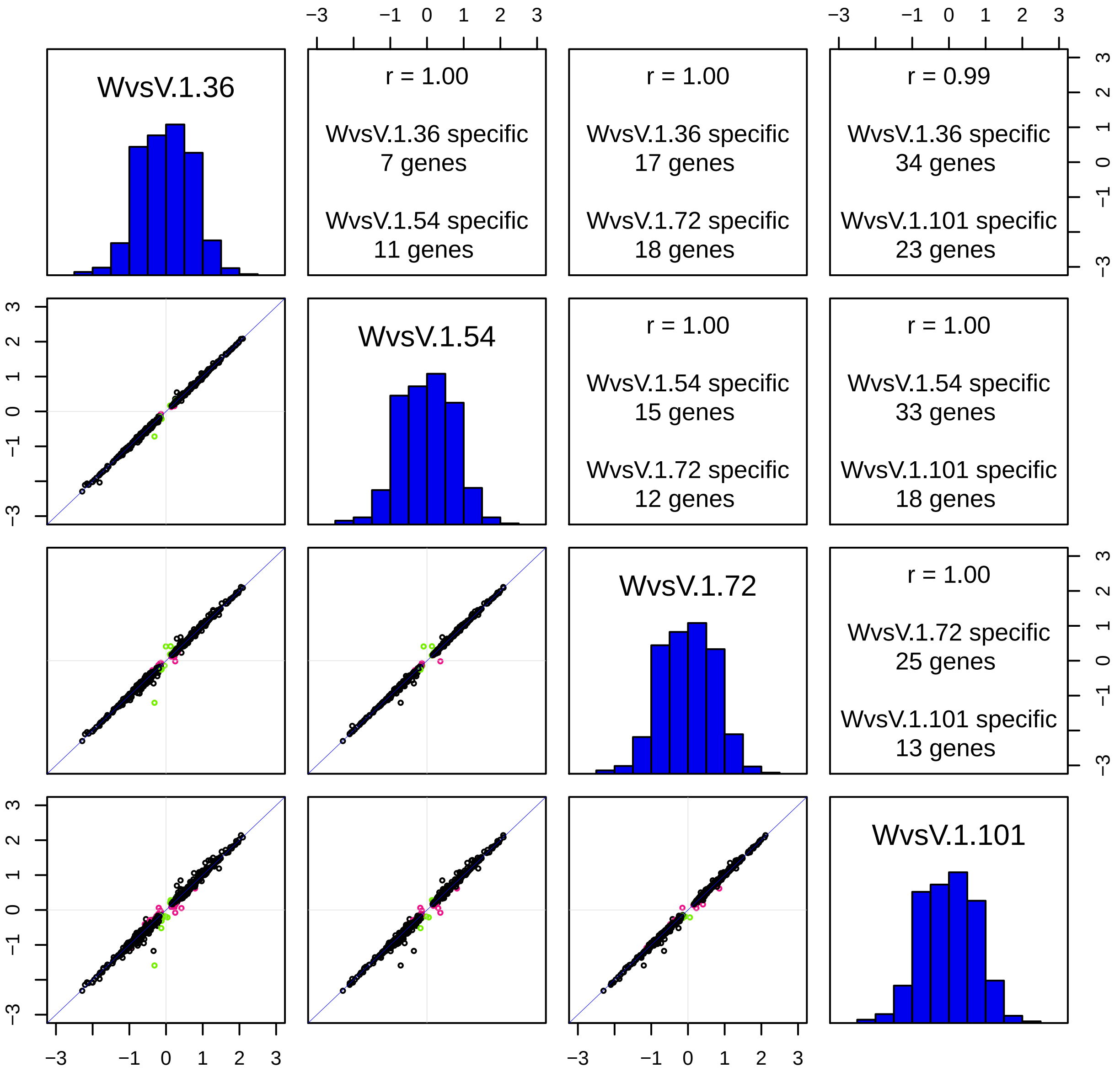
WvsV.1.36
r = 1.00
WvsV.1.36 specific
7 genes
WvsV.1.54 specific
11 genes
r = 1.00
WvsV.1.36 specific
17 genes
WvsV.1.72 specific
18 genes
r = 0.99
WvsV.1.36 specific
34 genes
WvsV.1.101 specific
23 genes
WvsV.1.54
r = 1.00
WvsV.1.54 specific
15 genes
WvsV.1.72 specific
12 genes
r = 1.00
WvsV.1.54 specific
33 genes
WvsV.1.101 specific
18 genes
WvsV.1.72
r = 1.00
WvsV.1.72 specific
25 genes
WvsV.1.101 specific
13 genes
WvsV.1.101
−3 −1 0 1 2 3

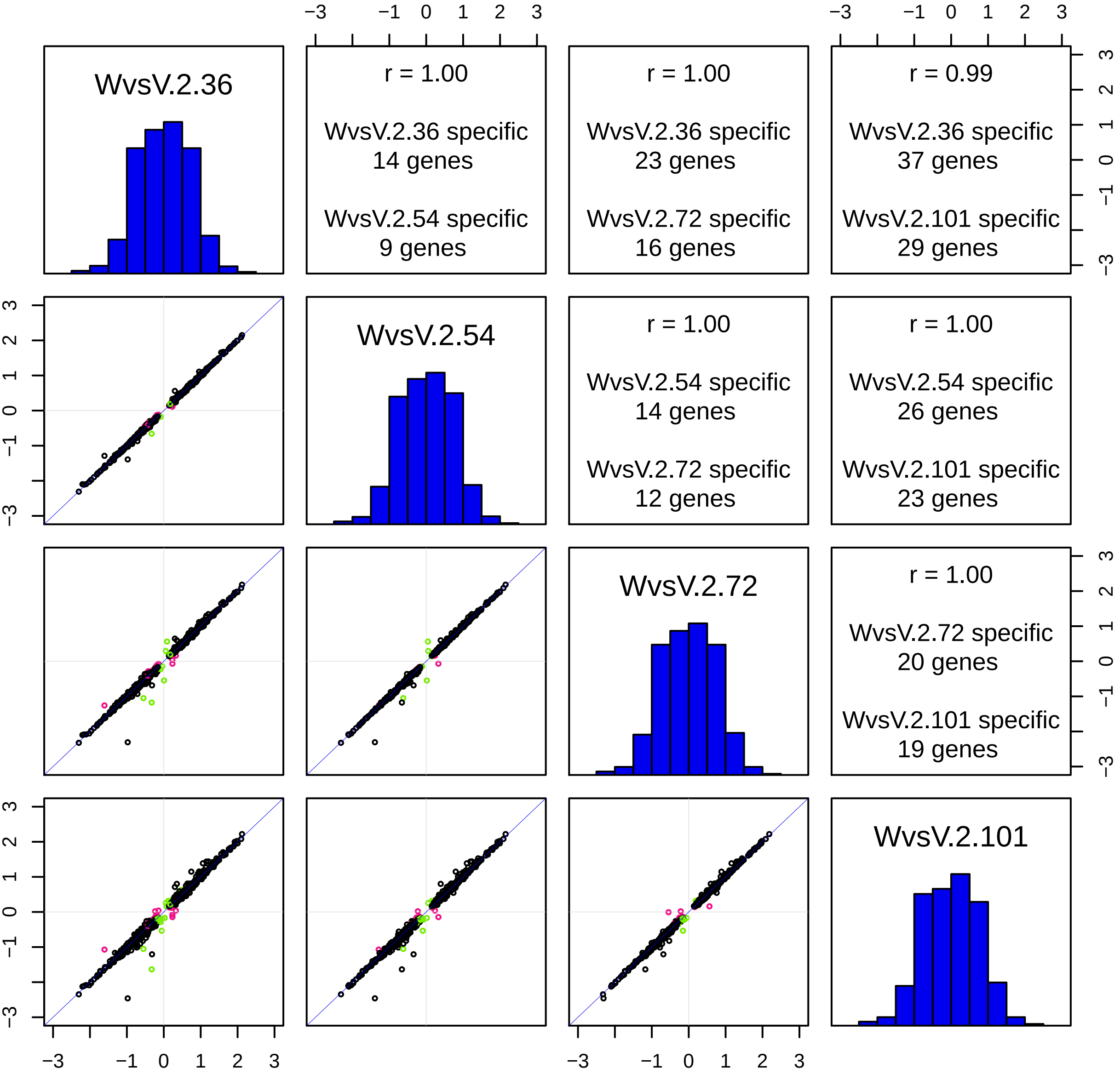

WvsV.2.36
r = 1.00
WvsV.2.36 specific
14 genes
WvsV.2.54 specific
9 genes
r = 1.00
WvsV.2.36 specific
23 genes
WvsV.2.72 specific
16 genes
r = 0.99
WvsV.2.36 specific
37 genes
WvsV.2.101 specific
29 genes
WvsV.2.54
r = 1.00
WvsV.2.54 specific
14 genes
WvsV.2.72 specific
12 genes
r = 1.00
WvsV.2.54 specific
26 genes
WvsV.2.101 specific
23 genes
WvsV.2.72
r = 1.00
WvsV.2.72 specific
20 genes
WvsV.2.101 specific
19 genes
WvsV.2.101
−3 −1 0 1 2 3

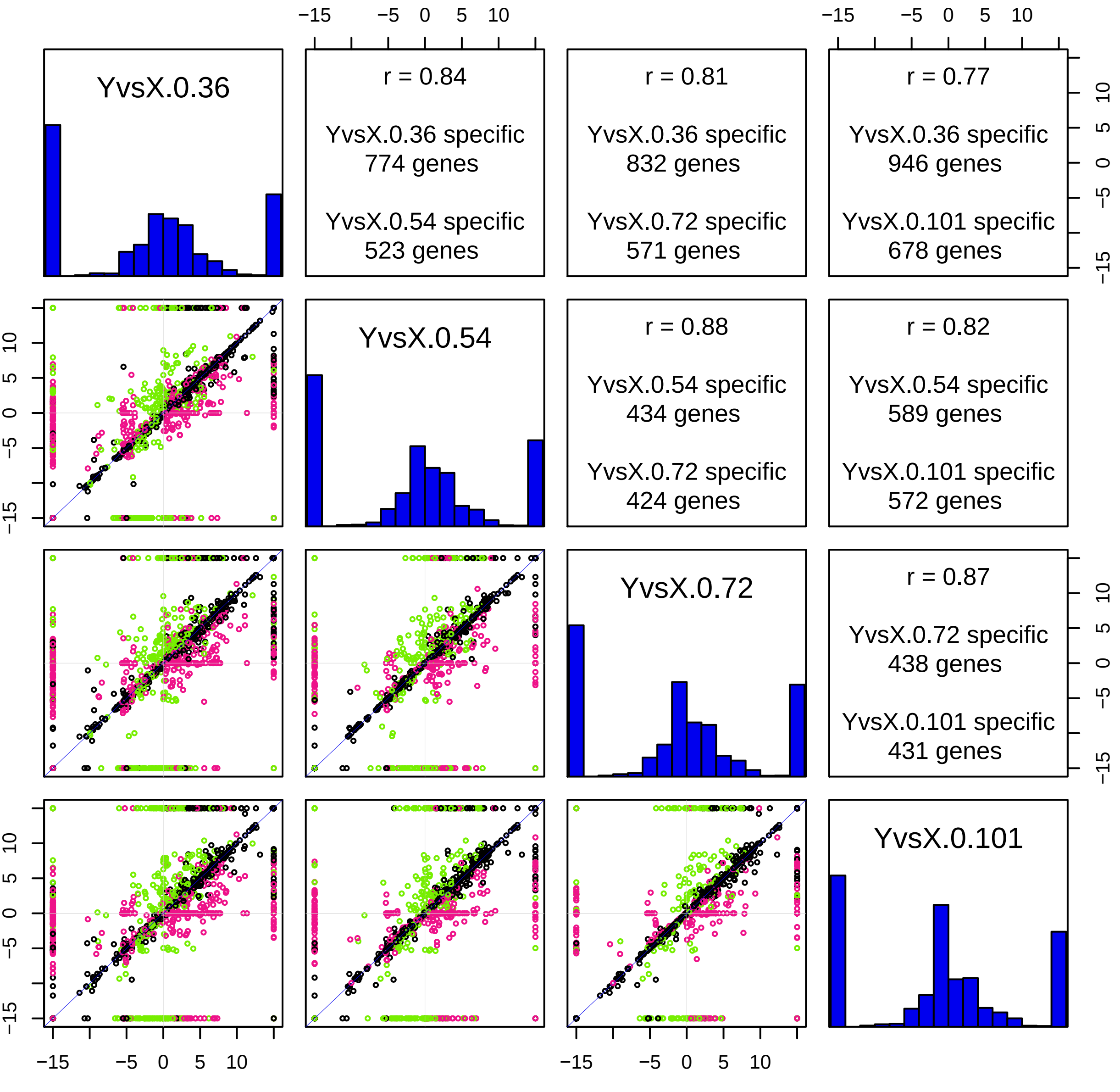

YvsX.0.36
r = 0.84
YvsX.0.36 specific
774 genes
YvsX.0.54 specific
523 genes
r = 0.81
YvsX.0.36 specific
832 genes
YvsX.0.72 specific
571 genes
r = 0.77
YvsX.0.36 specific
946 genes
YvsX.0.101 specific
678 genes
YvsX.0.54
r = 0.88
YvsX.0.54 specific
434 genes
YvsX.0.72 specific
424 genes
r = 0.82
YvsX.0.54 specific
589 genes
YvsX.0.101 specific
572 genes
YvsX.0.72
r = 0.87
YvsX.0.72 specific
438 genes
YvsX.0.101 specific
431 genes
YvsX.0.101
−15 −5 0 5 10

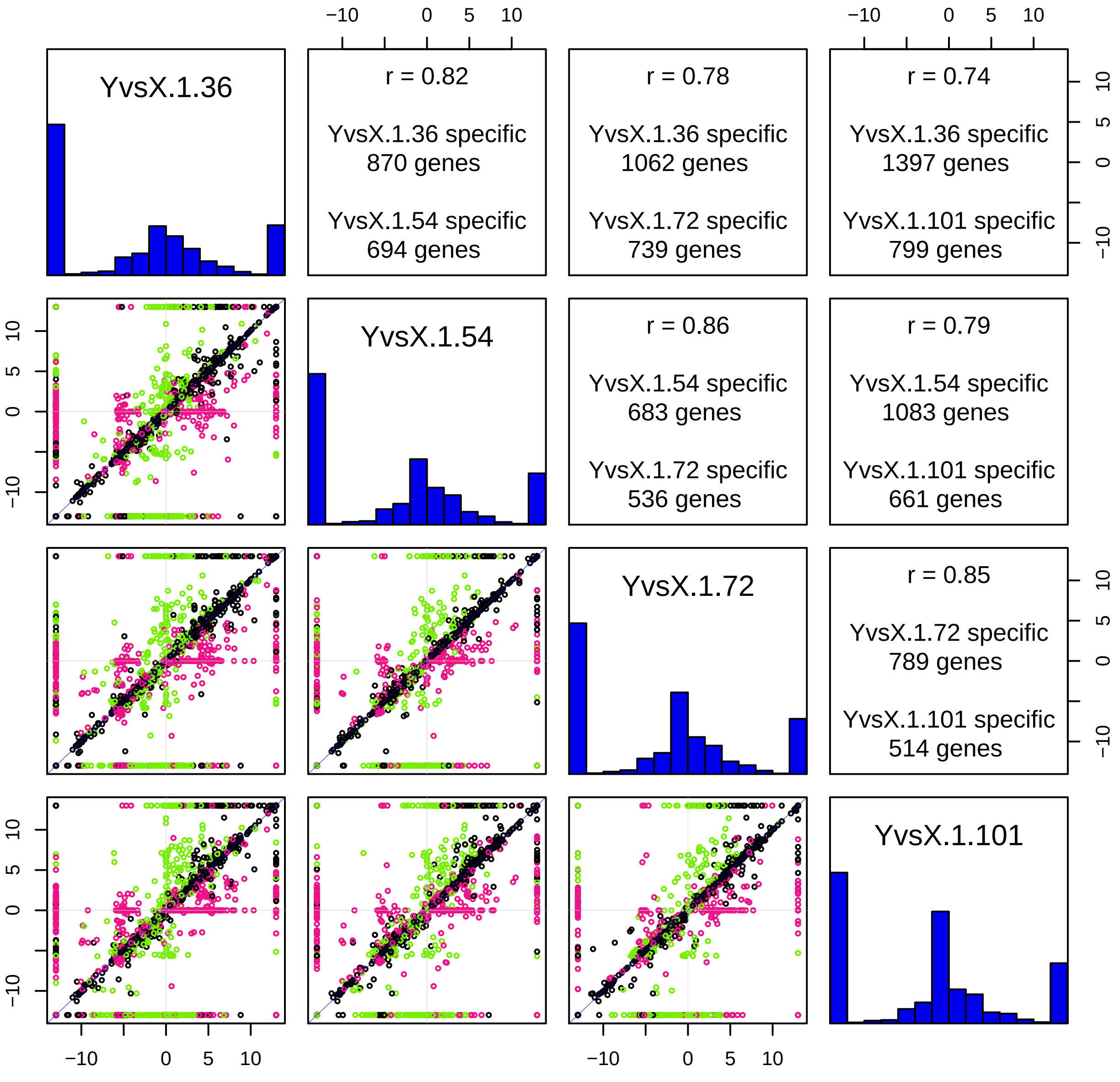
YvsX.1.36
YvsX.1.54
YvsX.1.72
YvsX.1.101
r = 0.82
YvsX.1.36 specific
870 genes
YvsX.1.54 specific
694 genes
r = 0.78
YvsX.1.36 specific
1062 genes
YvsX.1.72 specific
739 genes
r = 0.74
YvsX.1.36 specific
1397 genes
YvsX.1.101 specific
799 genes
r = 0.86
YvsX.1.54 specific
683 genes
YvsX.1.72 specific
536 genes
r = 0.79
YvsX.1.54 specific
1083 genes
YvsX.1.101 specific
661 genes
r = 0.85
YvsX.1.72 specific
789 genes
YvsX.1.101 specific
514 genes
−10
0
5
10

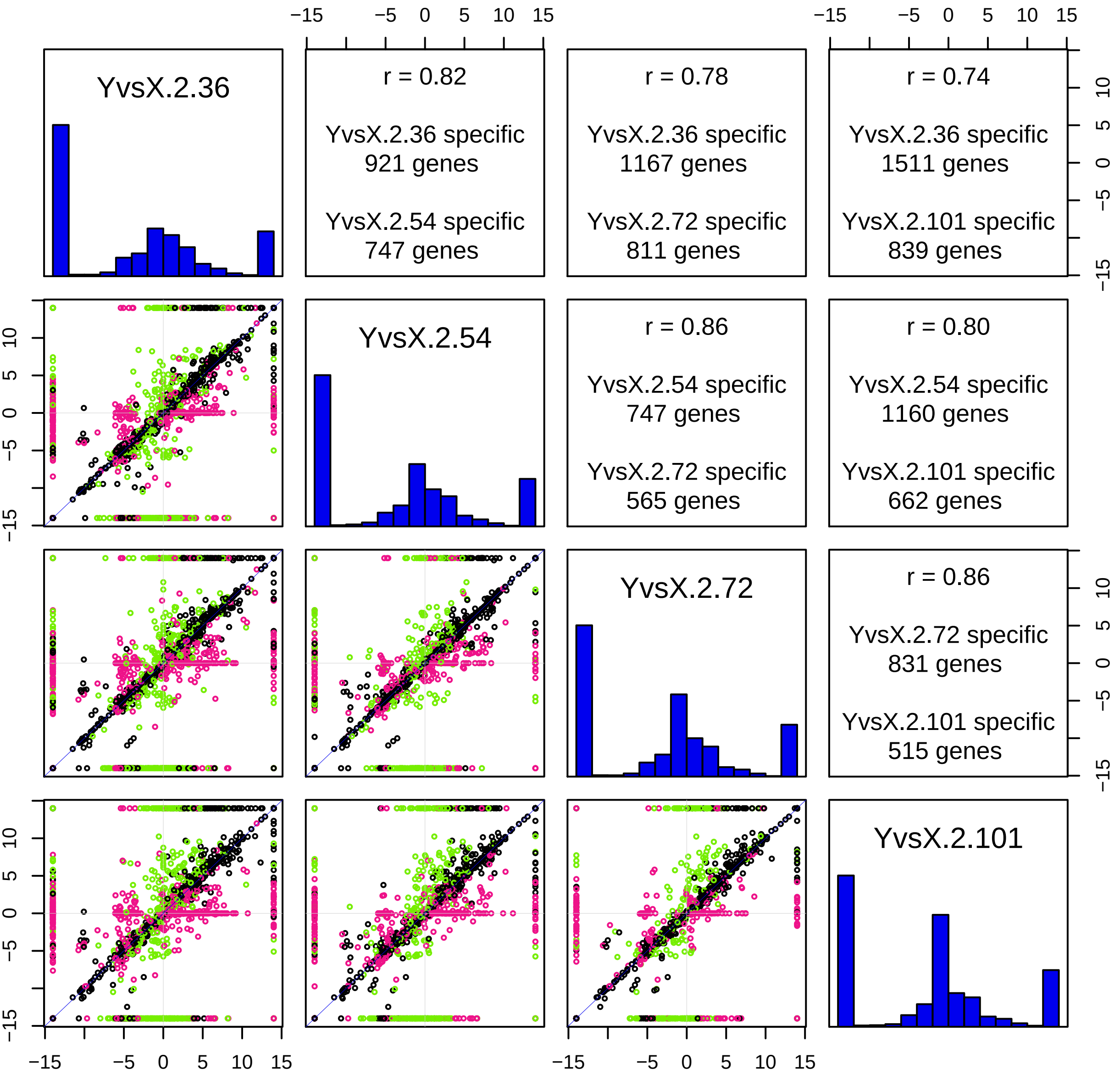
YvsX.2.36
r = 0.82
YvsX.2.36 specific
921 genes
YvsX.2.54 specific
747 genes
r = 0.78
YvsX.2.36 specific
1167 genes
YvsX.2.72 specific
811 genes
r = 0.74
YvsX.2.36 specific
1511 genes
YvsX.2.101 specific
839 genes
YvsX.2.54
r = 0.86
YvsX.2.54 specific
747 genes
YvsX.2.72 specific
565 genes
r = 0.80
YvsX.2.54 specific
1160 genes
YvsX.2.101 specific
662 genes
YvsX.2.72
r = 0.86
YvsX.2.72 specific
831 genes
YvsX.2.101 specific
515 genes
YvsX.2.101
−15
−5
0
5
10
15

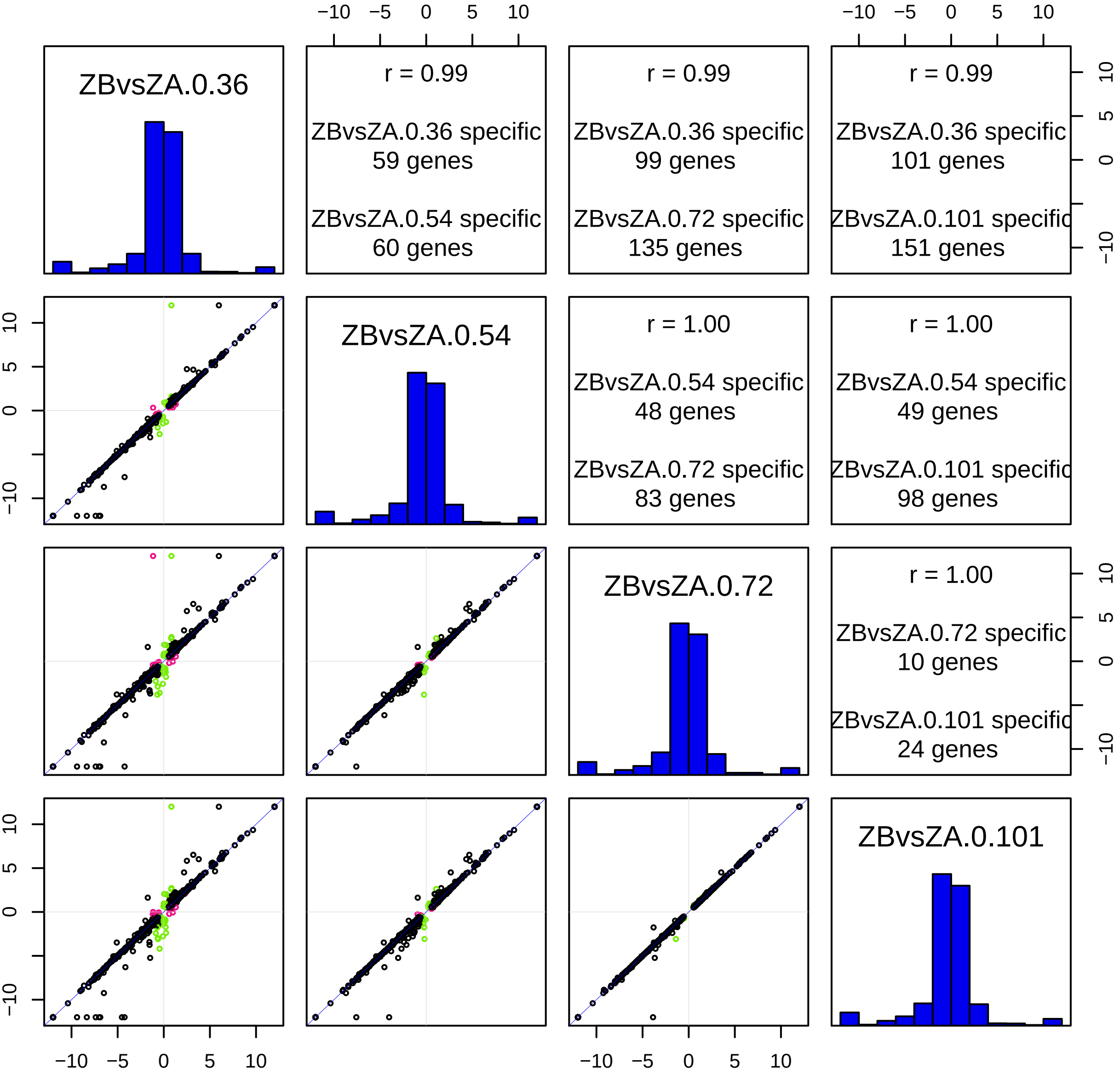

ZBvsZA.0.36
r = 0.99
ZBvsZA.0.36 specific
59 genes
ZBvsZA.0.54 specific
60 genes
r = 0.99
ZBvsZA.0.36 specific
99 genes
ZBvsZA.0.72 specific
135 genes
r = 0.99
ZBvsZA.0.36 specific
101 genes
ZBvsZA.0.101 specific
151 genes
ZBvsZA.0.54
r = 1.00
ZBvsZA.0.54 specific
48 genes
ZBvsZA.0.72 specific
83 genes
r = 1.00
ZBvsZA.0.54 specific
49 genes
ZBvsZA.0.101 specific
98 genes
ZBvsZA.0.72
r = 1.00
ZBvsZA.0.72 specific
10 genes
ZBvsZA.0.101 specific
24 genes
ZBvsZA.0.101
−10 −5 0 5 10

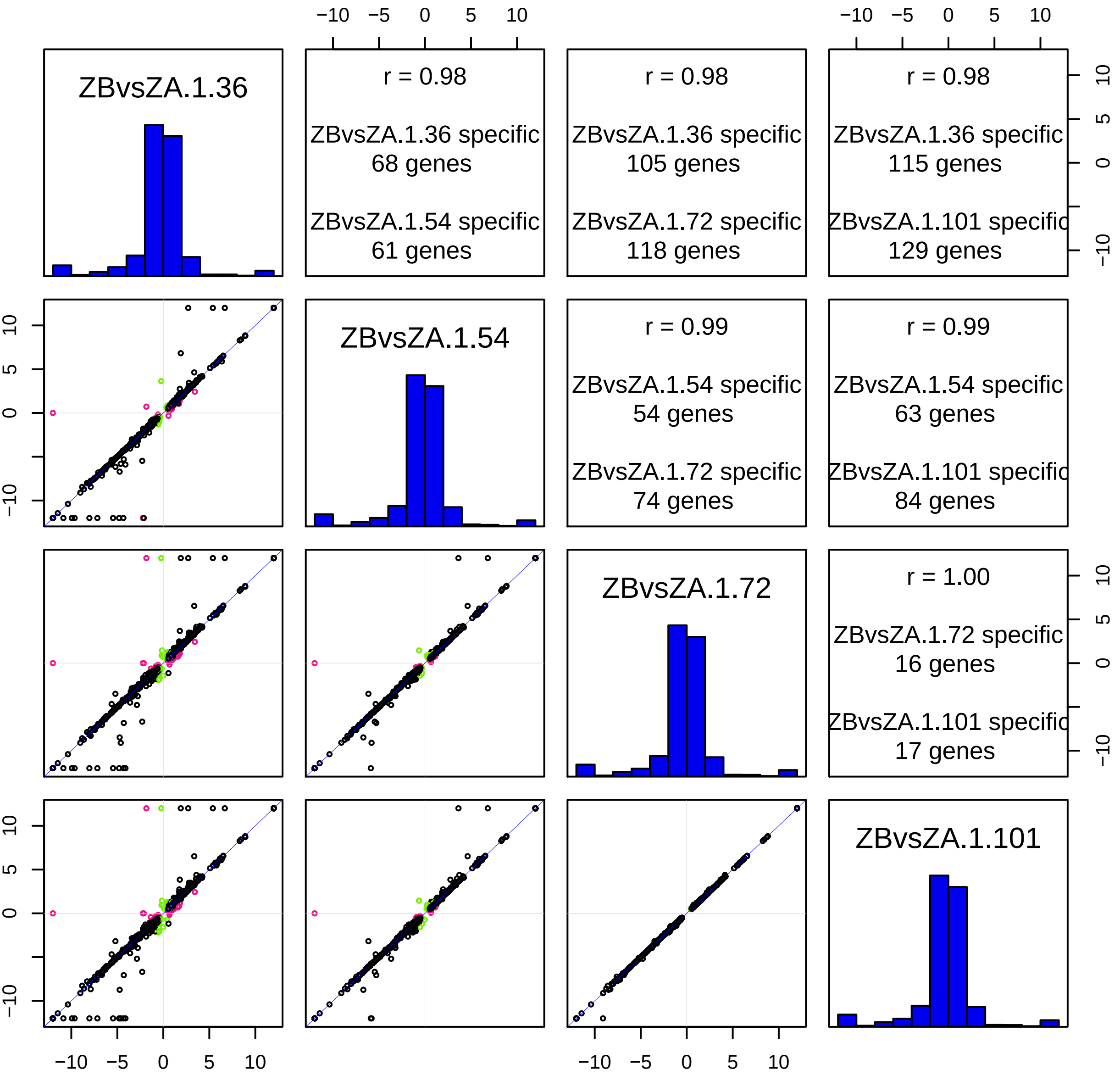
ZBvsZA.1.36
ZBvsZA.1.54
ZBvsZA.1.72
ZBvsZA.1.101
r = 0.98
ZBvsZA.1.36 specific
68 genes
ZBvsZA.1.54 specific
61 genes
r = 0.98
ZBvsZA.1.36 specific
105 genes
ZBvsZA.1.72 specific
118 genes
r = 0.98
ZBvsZA.1.36 specific
115 genes
ZBvsZA.1.101 specific
129 genes
r = 0.99
ZBvsZA.1.54 specific
54 genes
ZBvsZA.1.72 specific
74 genes
r = 0.99
ZBvsZA.1.54 specific
63 genes
ZBvsZA.1.101 specific
84 genes
r = 1.00
ZBvsZA.1.72 specific
16 genes
ZBvsZA.1.101 specific
17 genes
−10 −5 0 5 10

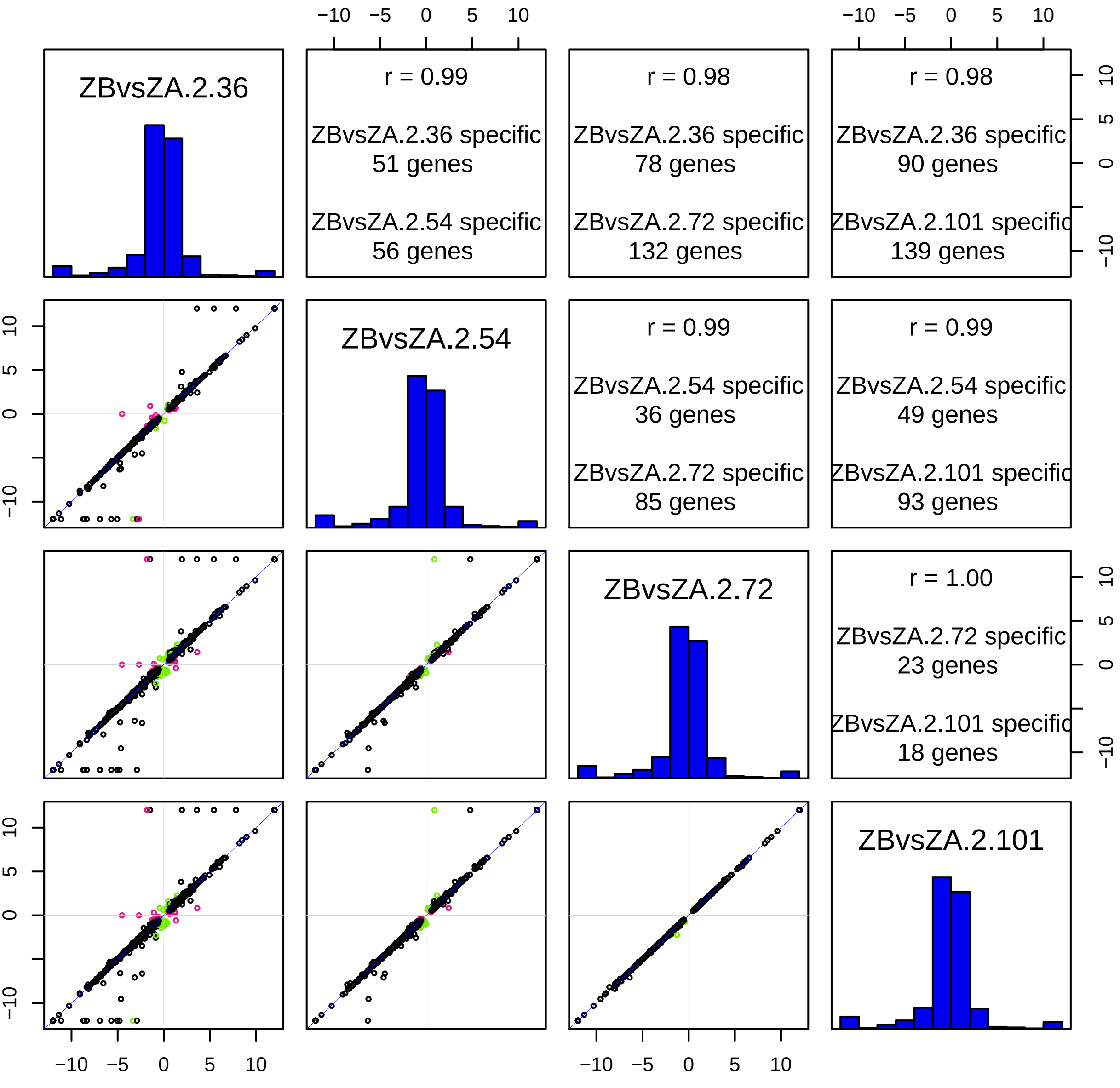
ZBvsZA.2.36
r = 0.99
ZBvsZA.2.36 specific
51 genes
ZBvsZA.2.54 specific
56 genes
r = 0.98
ZBvsZA.2.36 specific
78 genes
ZBvsZA.2.72 specific
132 genes
r = 0.98
ZBvsZA.2.36 specific
90 genes
ZBvsZA.2.101 specific
139 genes
ZBvsZA.2.54
r = 0.99
ZBvsZA.2.54 specific
36 genes
ZBvsZA.2.72 specific
85 genes
r = 0.99
ZBvsZA.2.54 specific
49 genes
ZBvsZA.2.101 specific
93 genes
ZBvsZA.2.72
r = 1.00
ZBvsZA.2.72 specific
23 genes
ZBvsZA.2.101 specific
18 genes
ZBvsZA.2.101
−10
−5
0
5
10

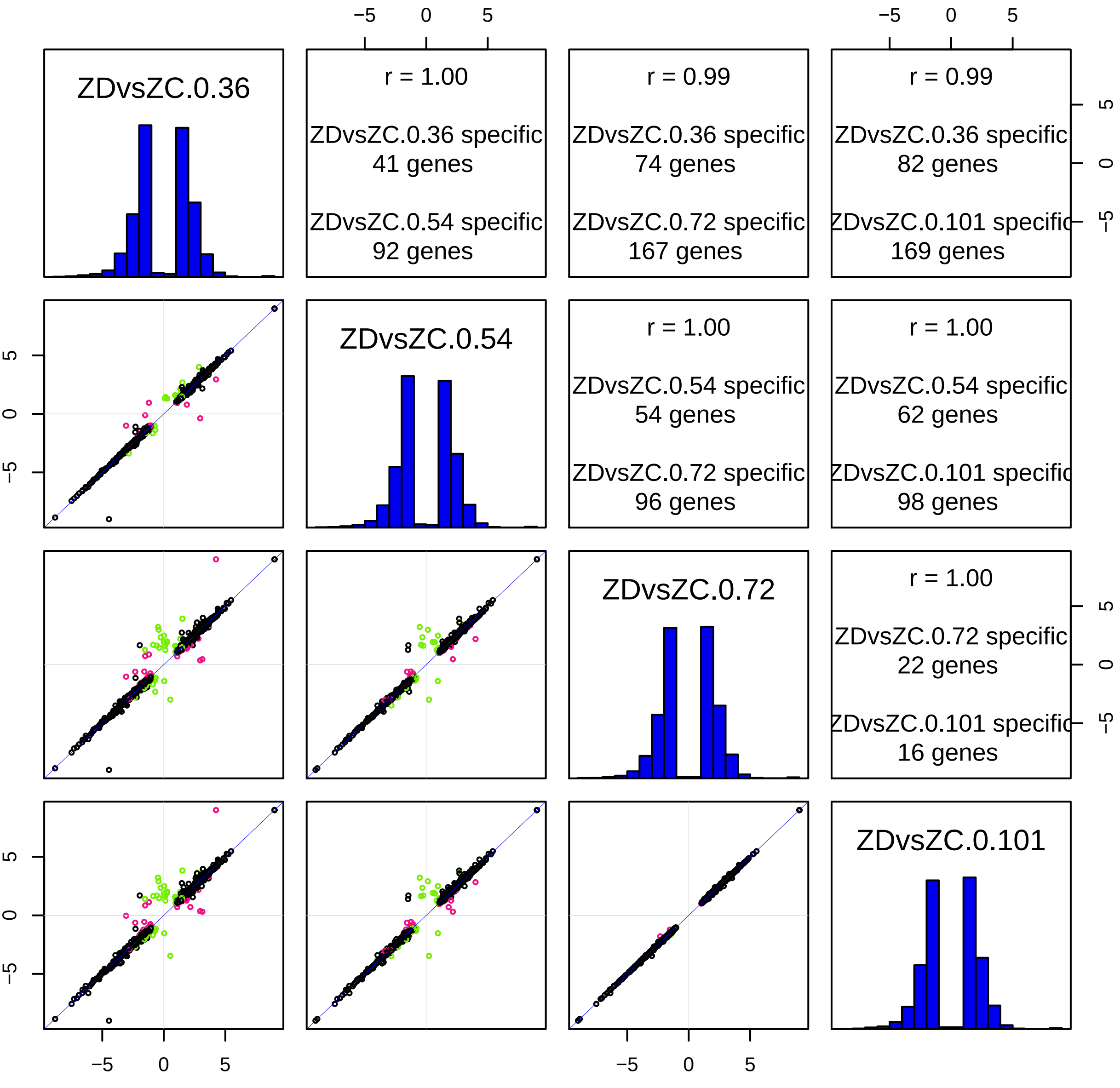

ZDvsZC.0.36
r = 1.00
ZDvsZC.0.36 specific
41 genes
ZDvsZC.0.54 specific
92 genes
r = 0.99
ZDvsZC.0.36 specific
74 genes
ZDvsZC.0.72 specific
167 genes
r = 0.99
ZDvsZC.0.36 specific
82 genes
ZDvsZC.0.101 specific
169 genes
ZDvsZC.0.54
r = 1.00
ZDvsZC.0.54 specific
54 genes
ZDvsZC.0.72 specific
96 genes
r = 1.00
ZDvsZC.0.54 specific
62 genes
ZDvsZC.0.101 specific
98 genes
ZDvsZC.0.72
r = 1.00
ZDvsZC.0.72 specific
22 genes
ZDvsZC.0.101 specific
16 genes
ZDvsZC.0.101
−5
0
5

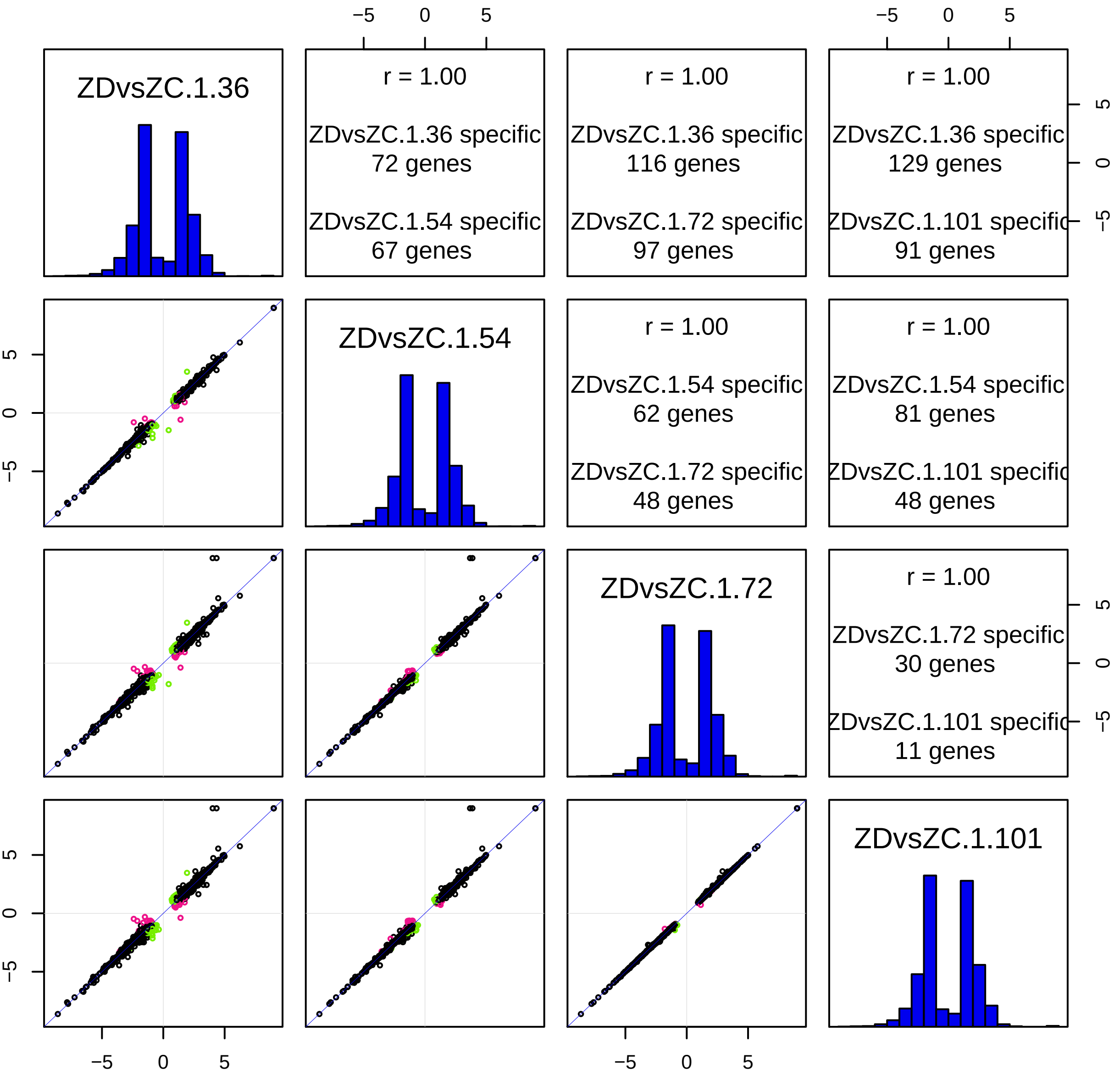

ZDvsZC.1.36
r = 1.00
ZDvsZC.1.36 specific
72 genes
ZDvsZC.1.54 specific
67 genes
r = 1.00
ZDvsZC.1.36 specific
116 genes
ZDvsZC.1.72 specific
97 genes
r = 1.00
ZDvsZC.1.36 specific
129 genes
ZDvsZC.1.101 specific
91 genes
ZDvsZC.1.54
r = 1.00
ZDvsZC.1.54 specific
62 genes
ZDvsZC.1.72 specific
48 genes
r = 1.00
ZDvsZC.1.54 specific
81 genes
ZDvsZC.1.101 specific
48 genes
ZDvsZC.1.72
r = 1.00
ZDvsZC.1.72 specific
30 genes
ZDvsZC.1.101 specific
11 genes
ZDvsZC.1.101
−5
0
5

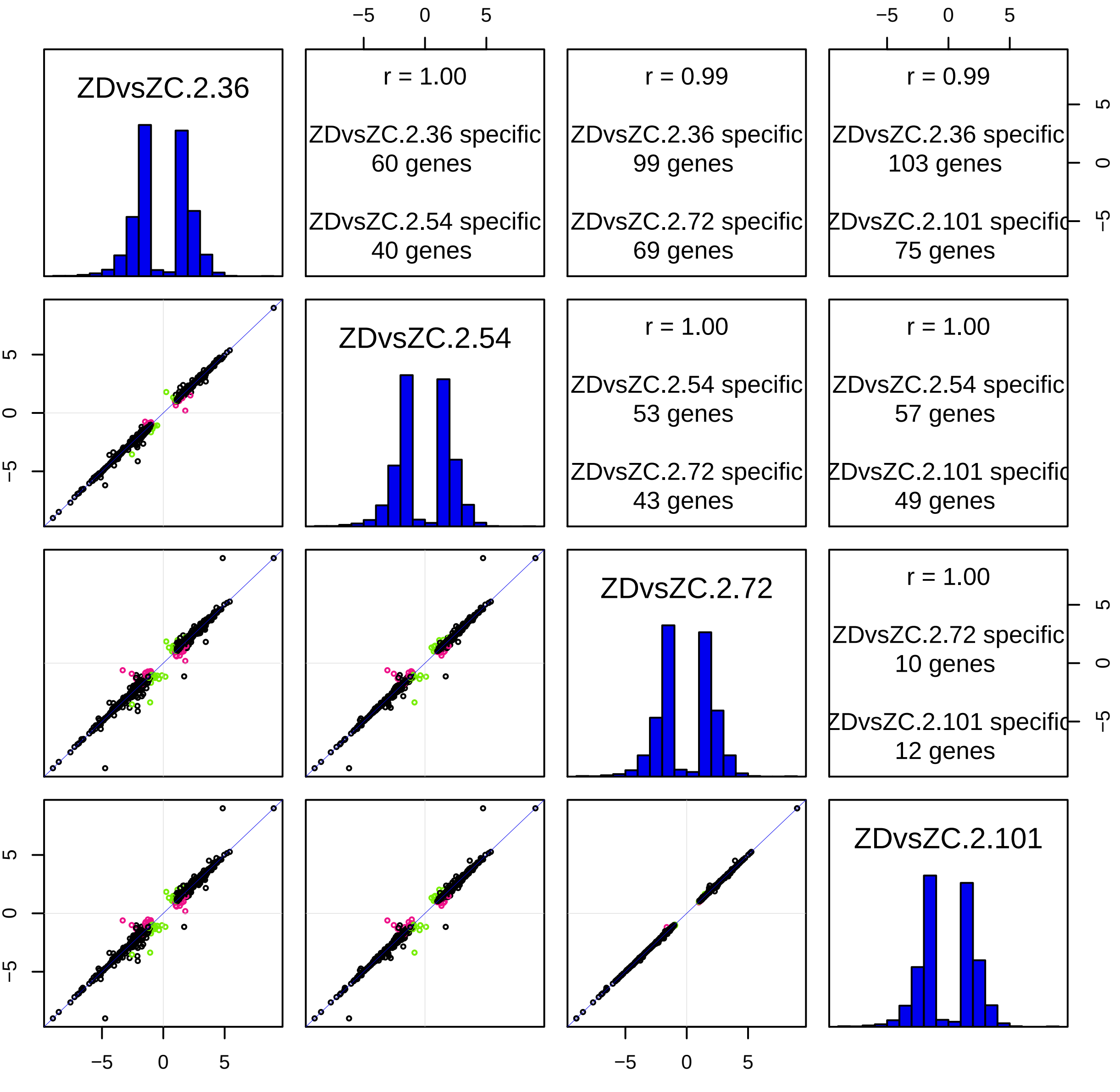
ZDvsZC.2.36
r = 1.00
ZDvsZC.2.36 specific
60 genes
ZDvsZC.2.54 specific
40 genes
r = 0.99
ZDvsZC.2.36 specific
99 genes
ZDvsZC.2.72 specific
69 genes
r = 0.99
ZDvsZC.2.36 specific
103 genes
ZDvsZC.2.101 specific
75 genes
ZDvsZC.2.54
r = 1.00
ZDvsZC.2.54 specific
53 genes
ZDvsZC.2.72 specific
43 genes
r = 1.00
ZDvsZC.2.54 specific
57 genes
ZDvsZC.2.101 specific
49 genes
ZDvsZC.2.72
r = 1.00
ZDvsZC.2.72 specific
10 genes
ZDvsZC.2.101 specific
12 genes
ZDvsZC.2.101
−5
0
5

Additional File 30. Compendium of scatterplots for all data sets with results aggregated by read pairing. The differentially expressed genes identified using an adjusted p-value (FDR) cutoff ≤0.05 at varying read lengths within a dataset were compared using Pearson's correlation implemented in the R statistical tool and illustrated as a matrix of scatterplots. The diagonal represents the histogram of log-transformed fold-changes within the comparison. The lower plots represent the correlation between comparisons with singleton DEGs identified for comparisons on the x-axis (pink) and y-axis (green). Genes with FDR > 0.05 in both comparisons are not shown. The upper portion of the plot lists the corresponding Pearson's correlation coefficient and the number of singleton DEGs identified in each comparison. Each scatterplot is labeled by the comparison according to the letter code from Supplementary Table 2. A separate plot is shown for the various read lengths.

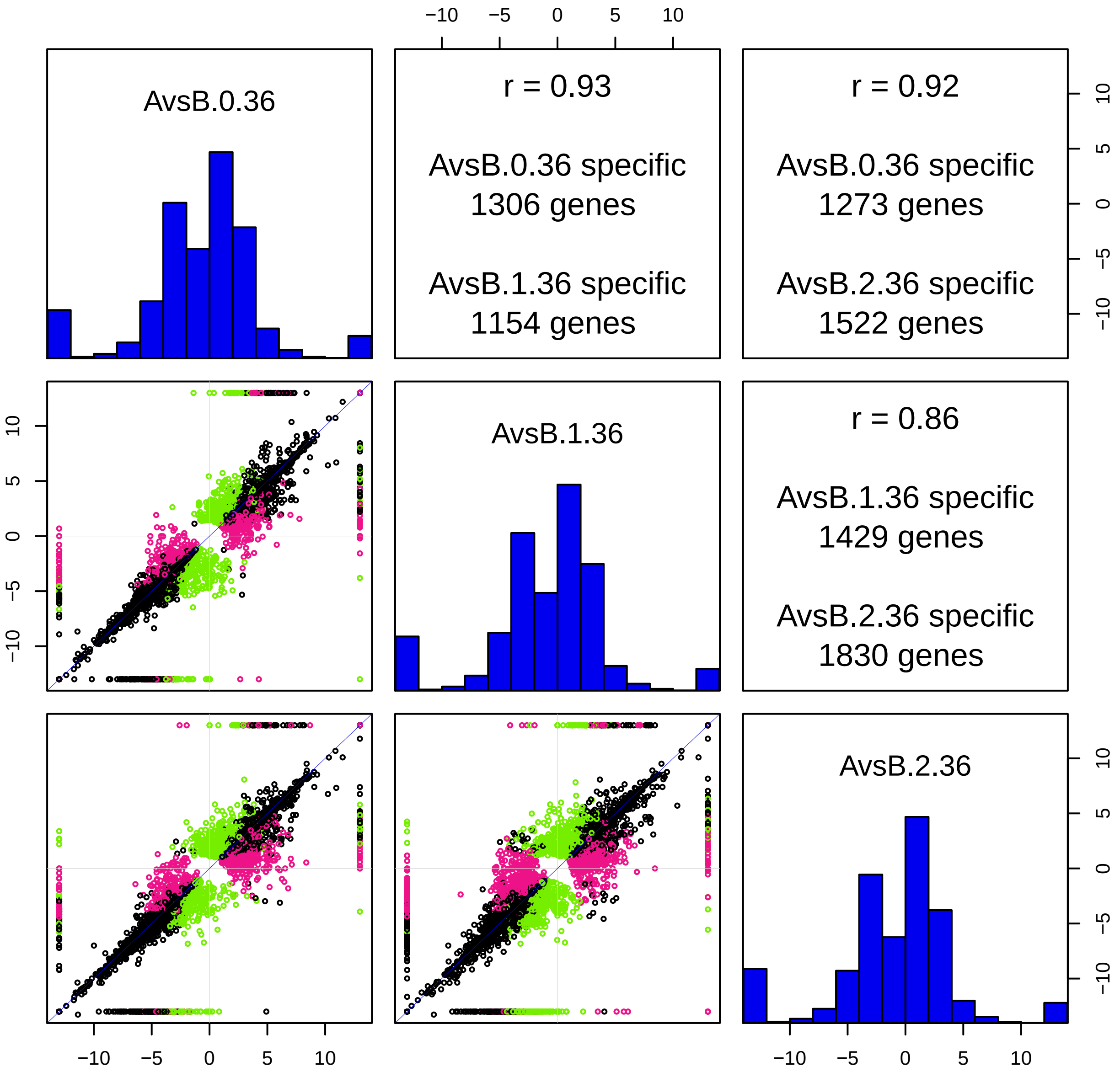
AvsB.0.36
r = 0.93
AvsB.0.36 specific
1306 genes
AvsB.1.36 specific
1154 genes
r = 0.92
AvsB.0.36 specific
1273 genes
AvsB.2.36 specific
1522 genes
AvsB.1.36
r = 0.86
AvsB.1.36 specific
1429 genes
AvsB.2.36 specific
1830 genes
AvsB.2.36
−10
−5
0
5
10

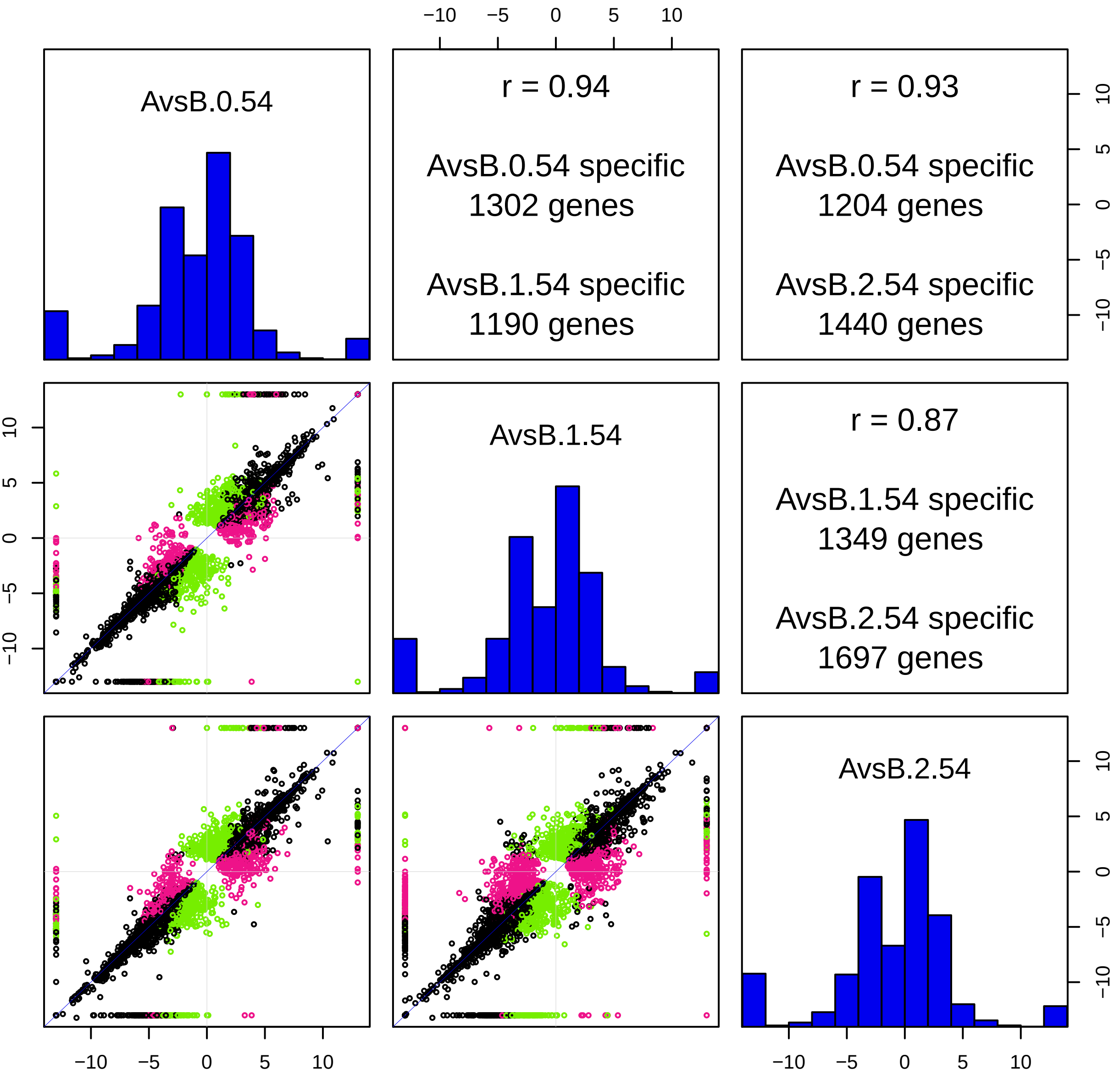
AvsB.0.54
r = 0.94
AvsB.0.54 specific
1302 genes
AvsB.1.54 specific
1190 genes
r = 0.93
AvsB.0.54 specific
1204 genes
AvsB.2.54 specific
1440 genes
AvsB.1.54
r = 0.87
AvsB.1.54 specific
1349 genes
AvsB.2.54 specific
1697 genes
AvsB.2.54
−10
−5
0
5
10

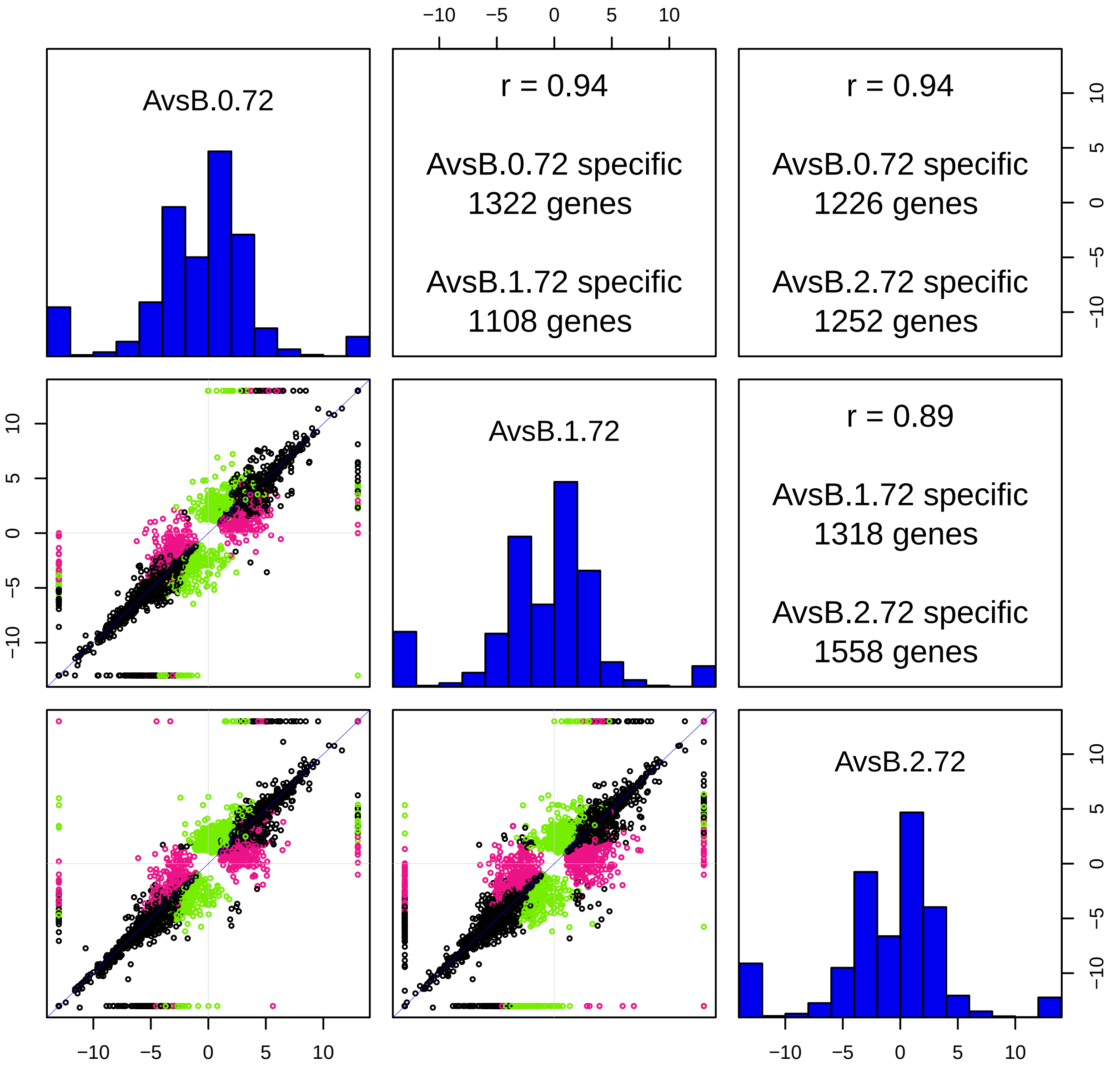

AvsB.0.72
r = 0.94
AvsB.0.72 specific
1322 genes
AvsB.1.72 specific
1108 genes
r = 0.94
AvsB.0.72 specific
1226 genes
AvsB.2.72 specific
1252 genes
AvsB.1.72
r = 0.89
AvsB.1.72 specific
1318 genes
AvsB.2.72 specific
1558 genes
AvsB.2.72
−10
−5
0
5
10

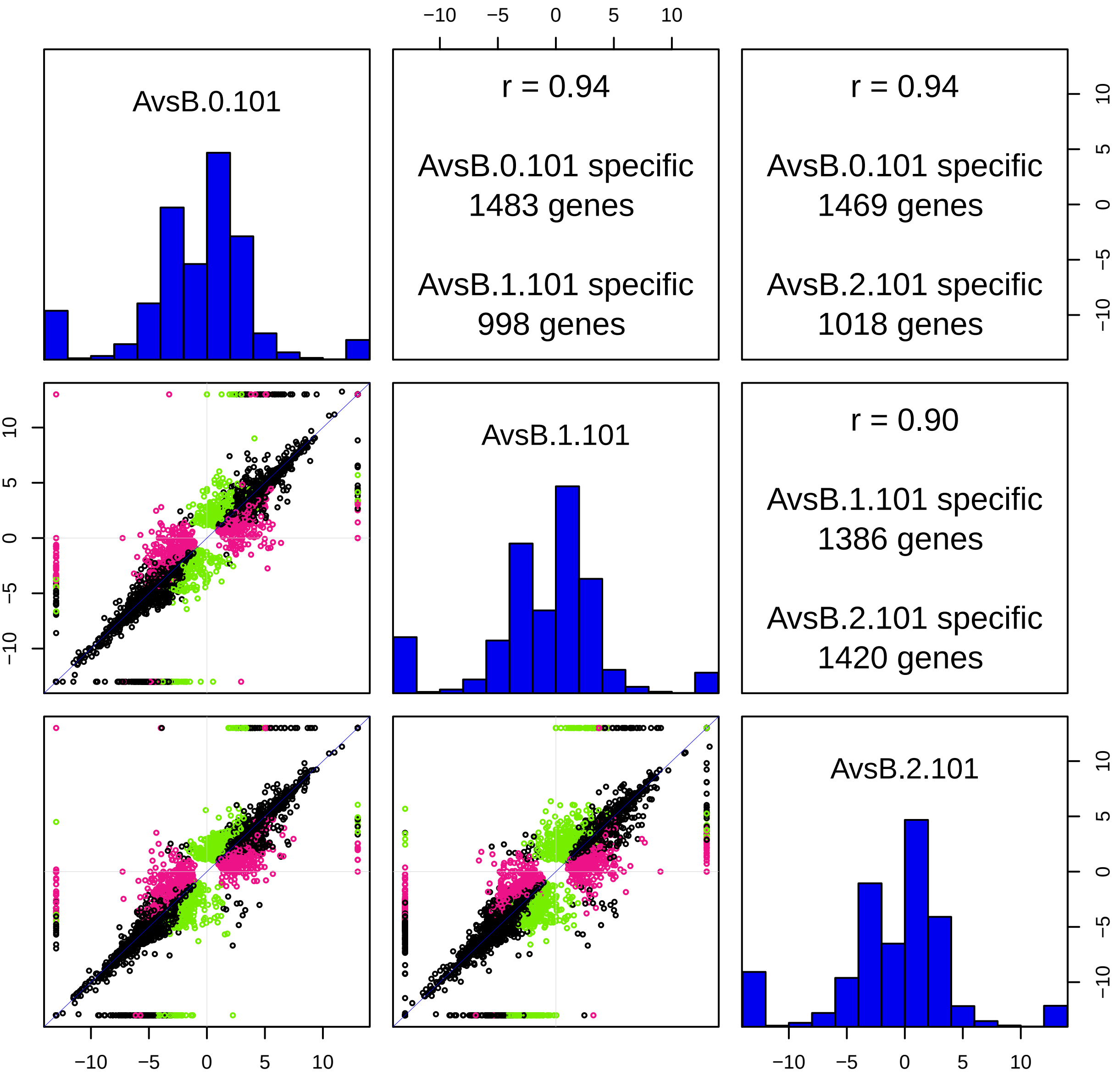
AvsB.0.101
r = 0.94
AvsB.0.101 specific
1483 genes
AvsB.1.101 specific
998 genes
r = 0.94
AvsB.0.101 specific
1469 genes
AvsB.2.101 specific
1018 genes
AvsB.1.101
r = 0.90
AvsB.1.101 specific
1386 genes
AvsB.2.101 specific
1420 genes
AvsB.2.101
−10
−5
0
5
10

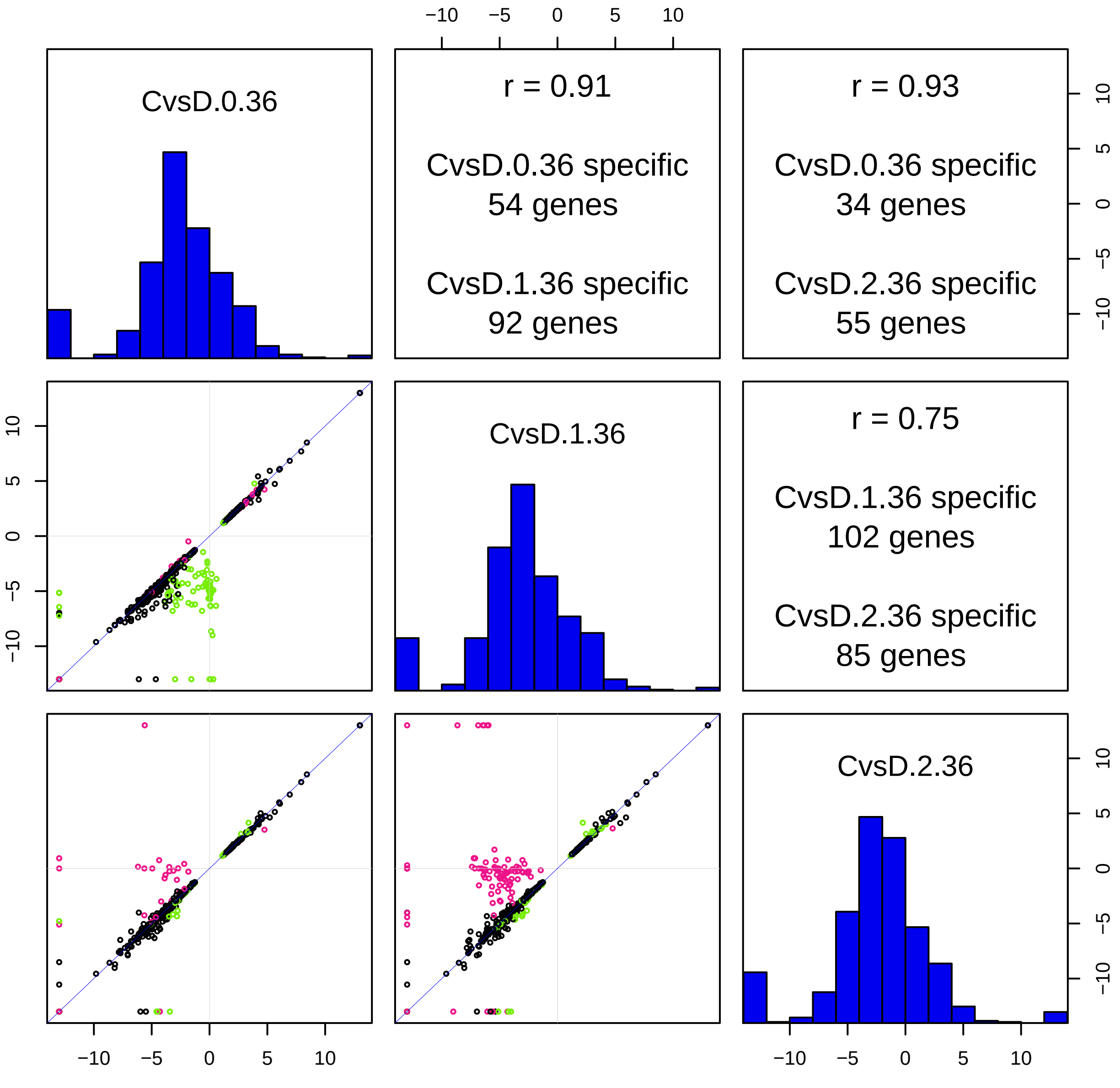
CvsD.0.36
r = 0.91
CvsD.0.36 specific
54 genes
CvsD.1.36 specific
92 genes
r = 0.93
CvsD.0.36 specific
34 genes
CvsD.2.36 specific
55 genes
CvsD.1.36
r = 0.75
CvsD.1.36 specific
102 genes
CvsD.2.36 specific
85 genes
CvsD.2.36
−10
−5
0
5
10

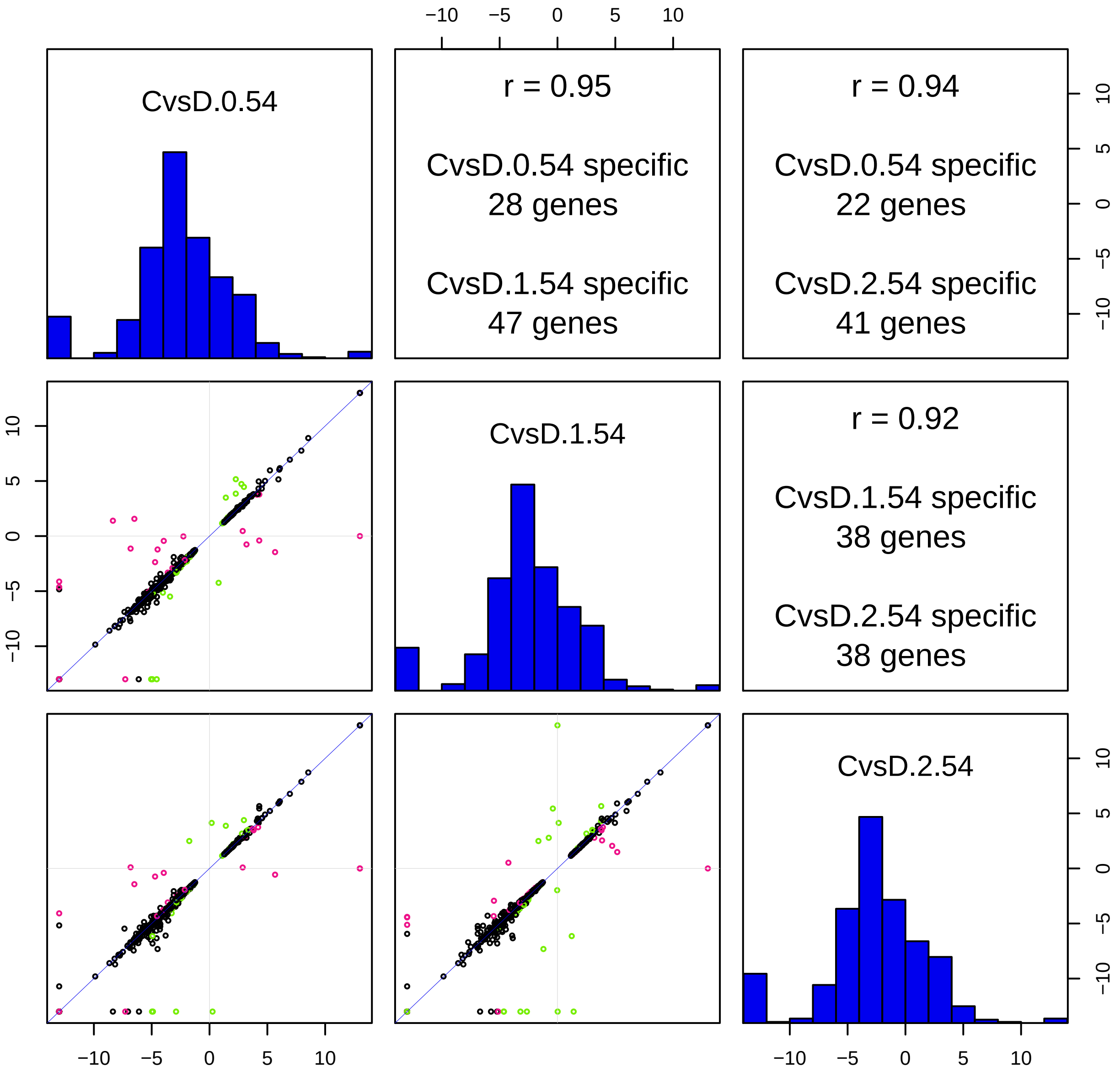

CvsD.0.54
r = 0.95
CvsD.0.54 specific
28 genes
CvsD.1.54 specific
47 genes
r = 0.94
CvsD.0.54 specific
22 genes
CvsD.2.54 specific
41 genes
CvsD.1.54
r = 0.92
CvsD.1.54 specific
38 genes
CvsD.2.54 specific
38 genes
CvsD.2.54
−10
−5
0
5
10

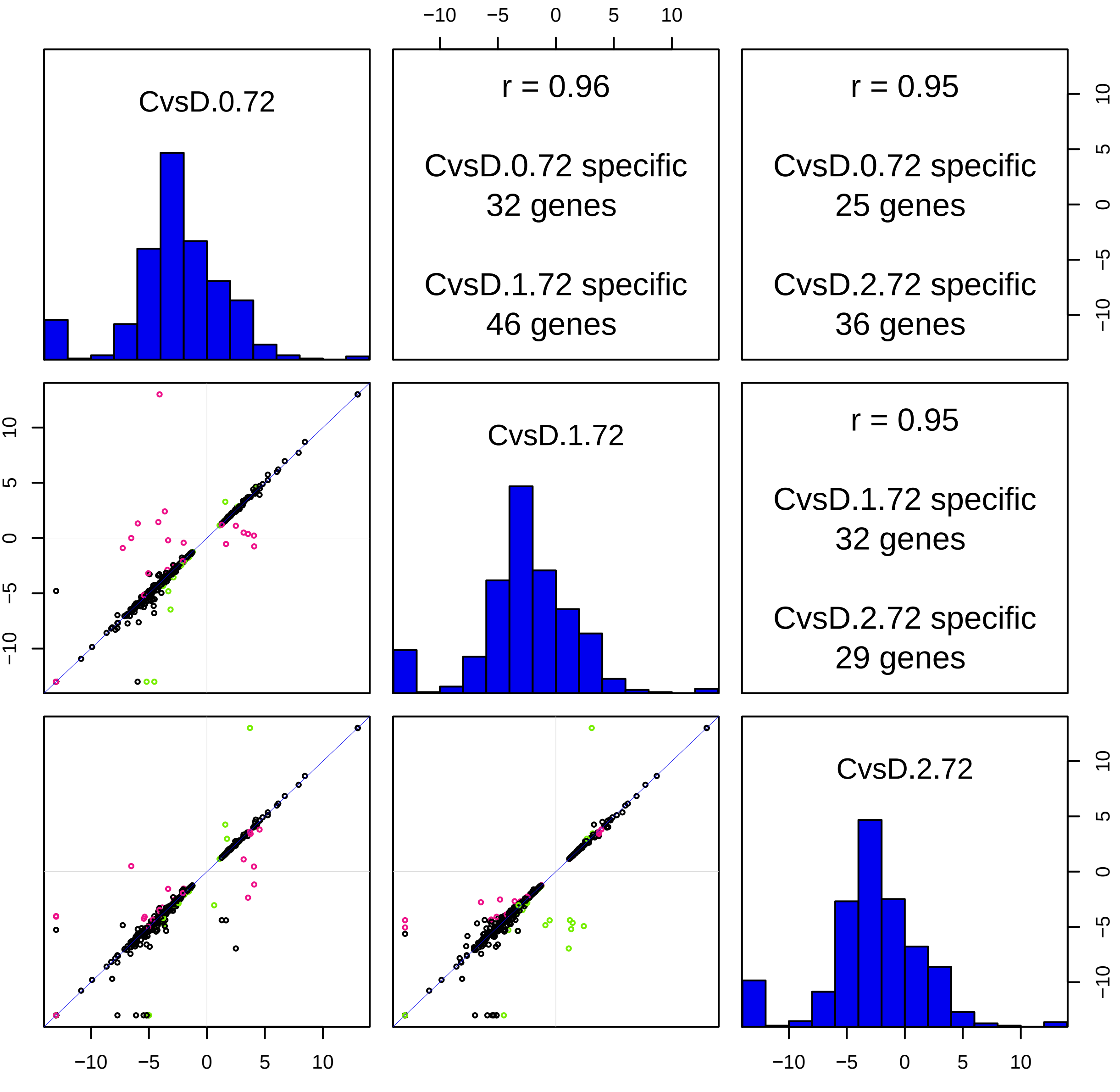
CvsD.0.72
r = 0.96
CvsD.0.72 specific
32 genes
CvsD.1.72 specific
46 genes
r = 0.95
CvsD.0.72 specific
25 genes
CvsD.2.72 specific
36 genes
CvsD.1.72
r = 0.95
CvsD.1.72 specific
32 genes
CvsD.2.72 specific
29 genes
CvsD.2.72
−10
−5
0
5
10

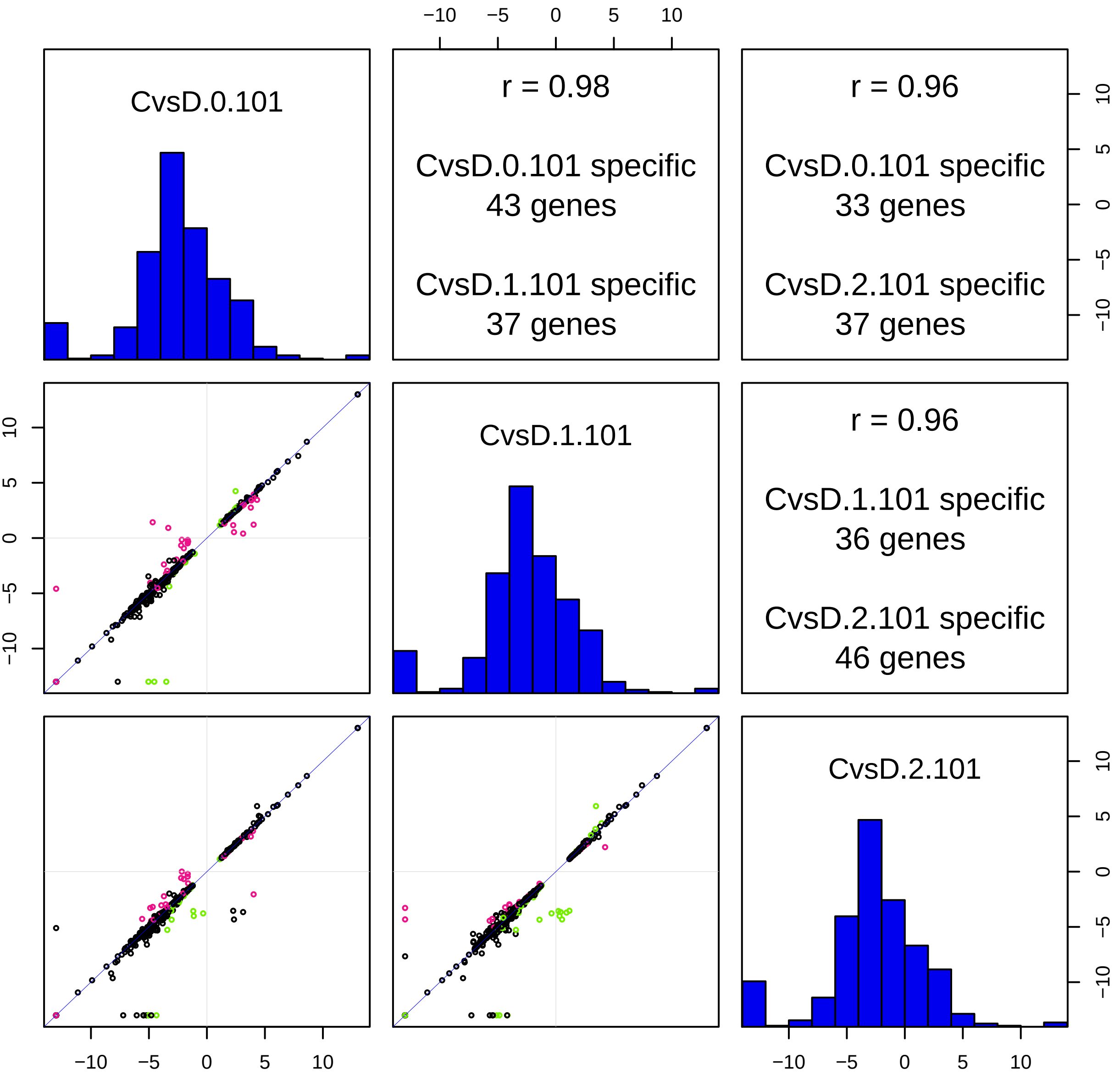

CvsD.0.101
r = 0.98
CvsD.0.101 specific
43 genes
CvsD.1.101 specific
37 genes
r = 0.96
CvsD.0.101 specific
33 genes
CvsD.2.101 specific
37 genes
CvsD.1.101
r = 0.96
CvsD.1.101 specific
36 genes
CvsD.2.101 specific
46 genes
CvsD.2.101
−10
−5
0
5
10

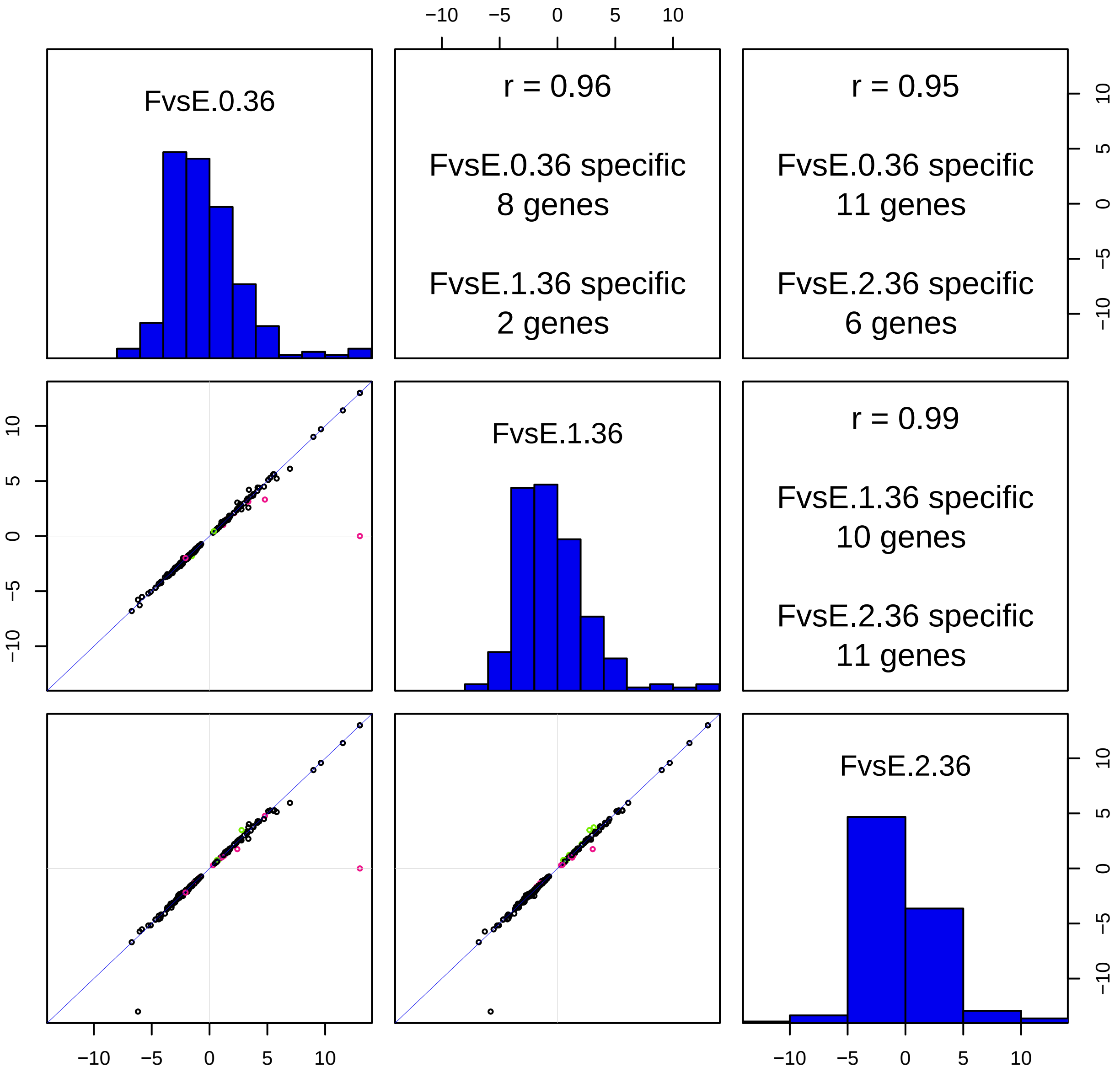

FvsE.0.36
r = 0.96
FvsE.0.36 specific
8 genes
FvsE.1.36 specific
2 genes
r = 0.95
FvsE.0.36 specific
11 genes
FvsE.2.36 specific
6 genes
FvsE.1.36
r = 0.99
FvsE.1.36 specific
10 genes
FvsE.2.36 specific
11 genes
FvsE.2.36
−10
−5
0
5
10

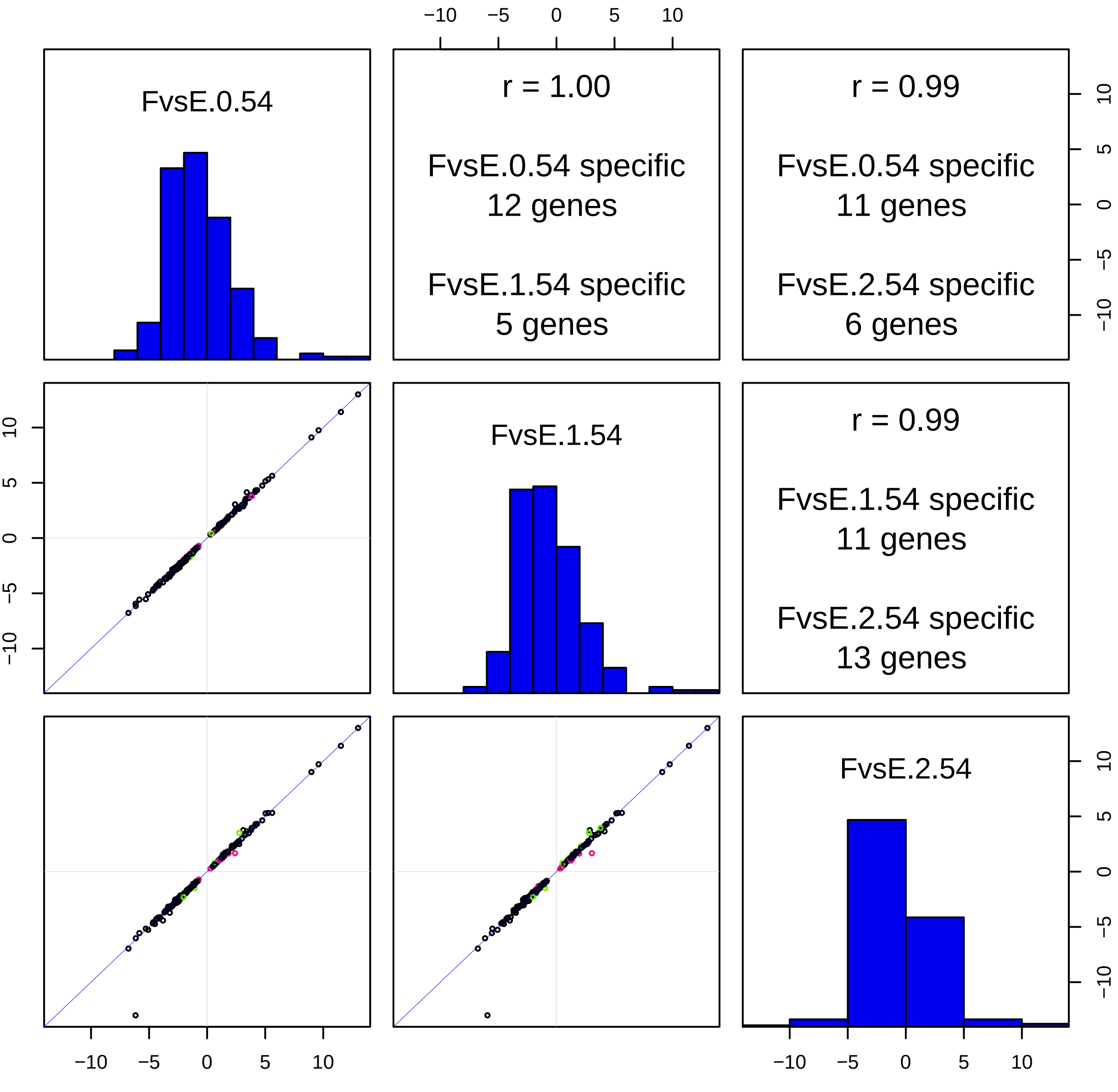
FvsE.0.54
r = 1.00
FvsE.0.54 specific
12 genes
FvsE.1.54 specific
5 genes
r = 0.99
FvsE.0.54 specific
11 genes
FvsE.2.54 specific
6 genes
FvsE.1.54
r = 0.99
FvsE.1.54 specific
11 genes
FvsE.2.54 specific
13 genes
FvsE.2.54
−10
−5
0
5
10

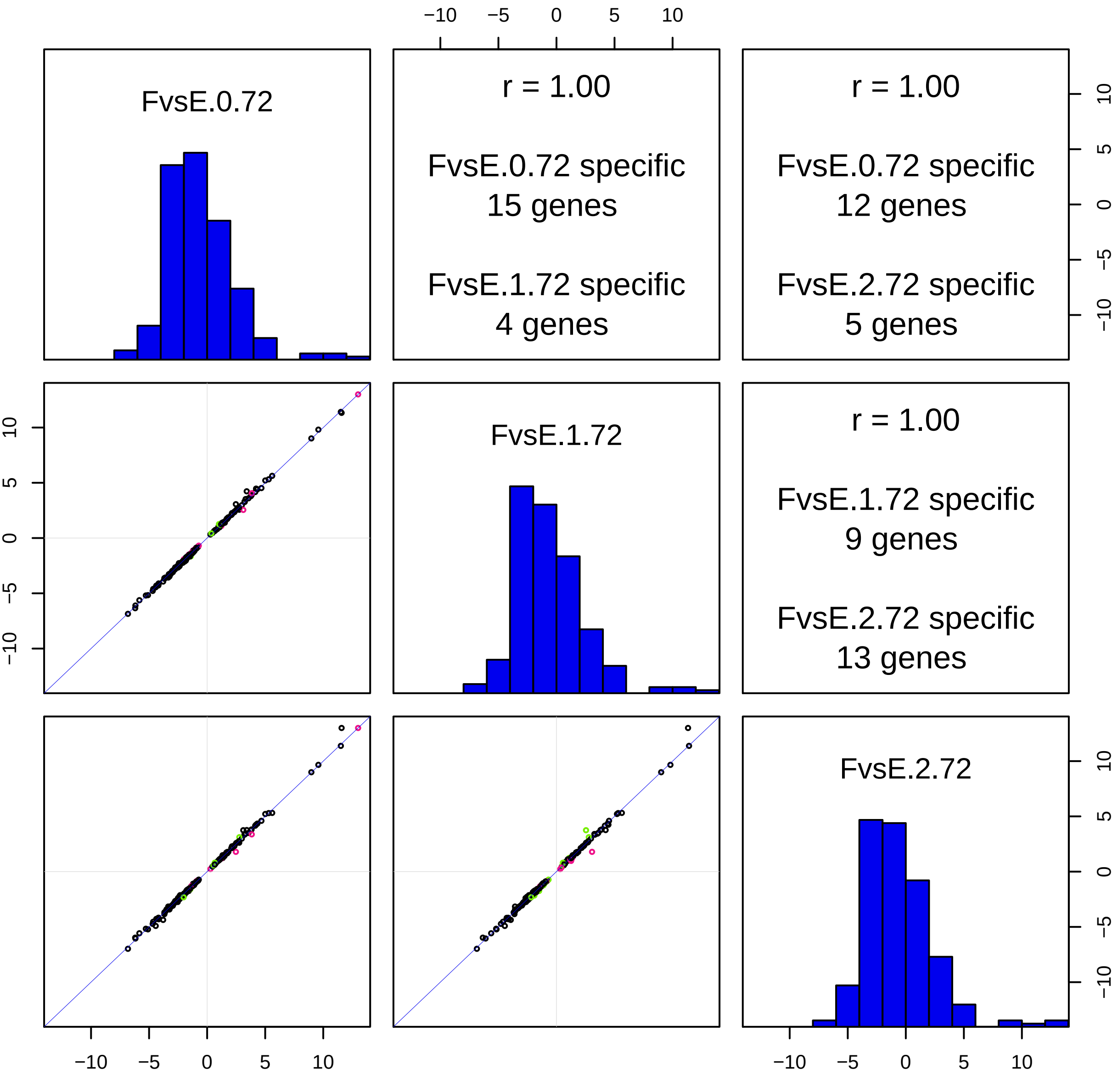
FvsE.0.72
r = 1.00
FvsE.0.72 specific
15 genes
FvsE.1.72 specific
4 genes
r = 1.00
FvsE.0.72 specific
12 genes
FvsE.2.72 specific
5 genes
FvsE.1.72
r = 1.00
FvsE.1.72 specific
9 genes
FvsE.2.72 specific
13 genes
FvsE.2.72
−10
−5
0
5
10

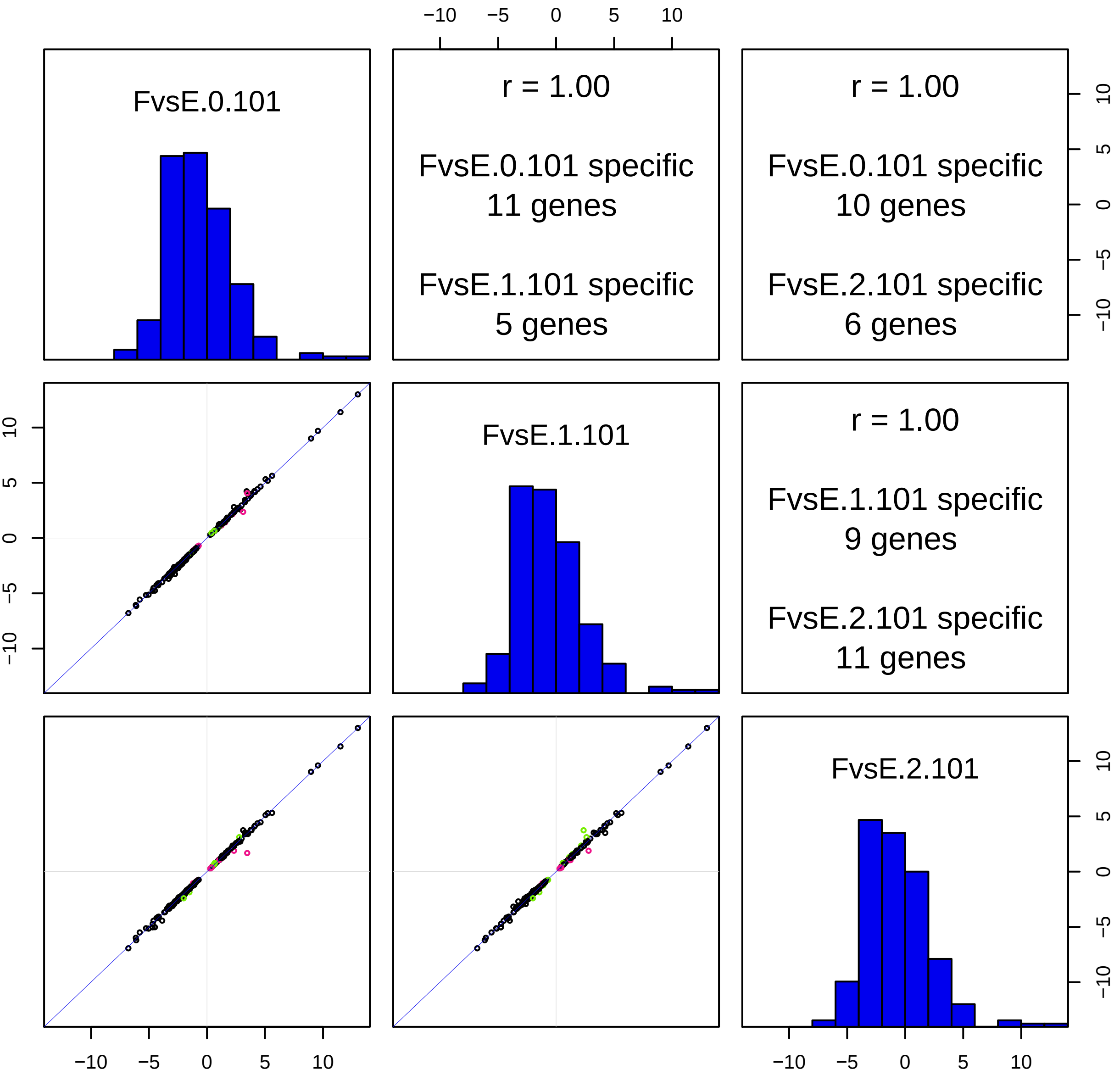
FvsE.0.101
r = 1.00
FvsE.0.101 specific
11 genes
FvsE.1.101 specific
5 genes
r = 1.00
FvsE.0.101 specific
10 genes
FvsE.2.101 specific
6 genes
FvsE.1.101
r = 1.00
FvsE.1.101 specific
9 genes
FvsE.2.101 specific
11 genes
FvsE.2.101
−10
−5
0
5
10

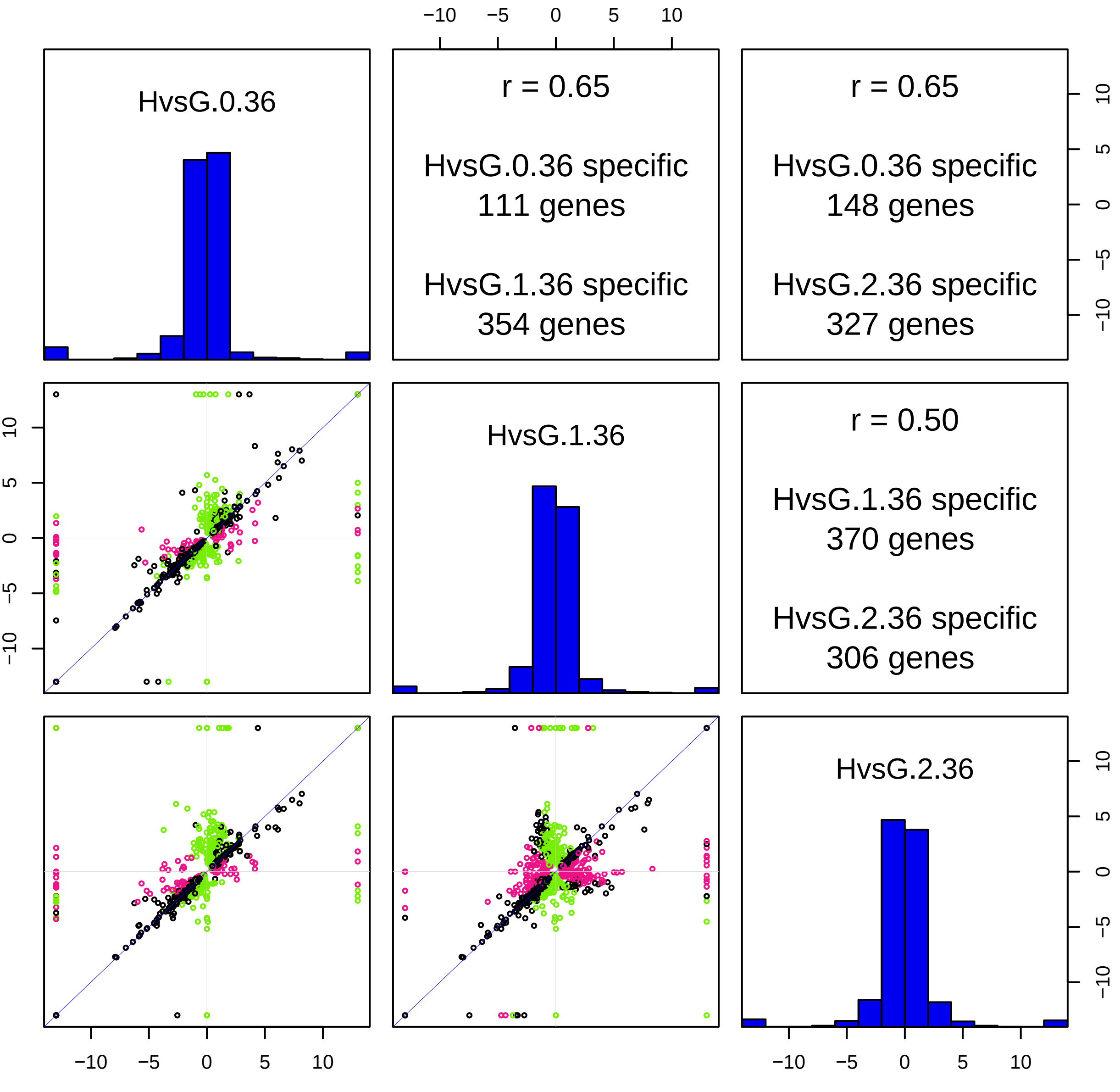
HvsG.0.36
r = 0.65
HvsG.0.36 specific
111 genes
HvsG.1.36 specific
354 genes
r = 0.65
HvsG.0.36 specific
148 genes
HvsG.2.36 specific
327 genes
HvsG.1.36
r = 0.50
HvsG.1.36 specific
370 genes
HvsG.2.36 specific
306 genes
HvsG.2.36
−10
−5
0
5
10

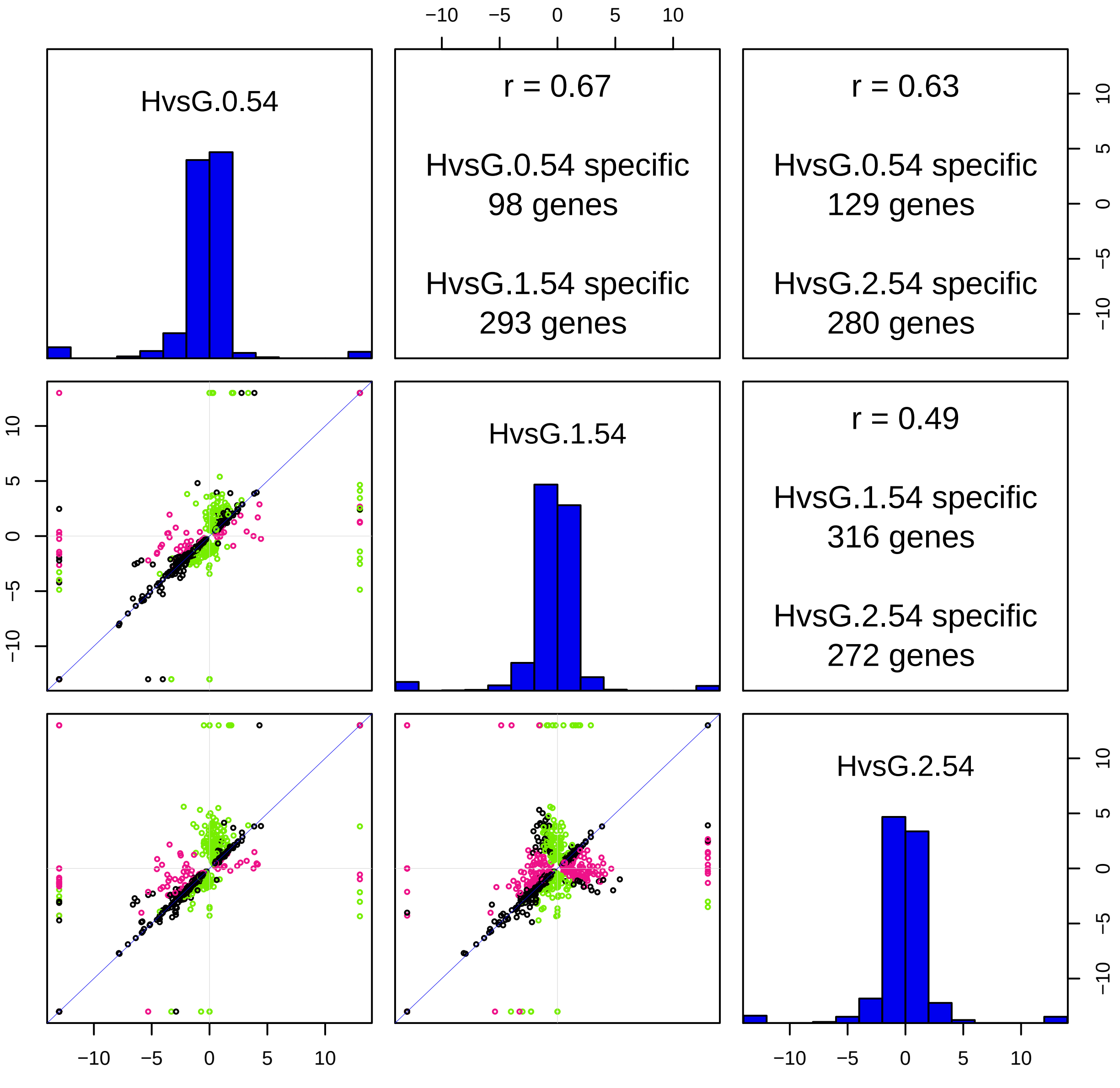
HvsG.0.54
r = 0.67
HvsG.0.54 specific
98 genes
HvsG.1.54 specific
293 genes
r = 0.63
HvsG.0.54 specific
129 genes
HvsG.2.54 specific
280 genes
HvsG.1.54
r = 0.49
HvsG.1.54 specific
316 genes
HvsG.2.54 specific
272 genes
HvsG.2.54

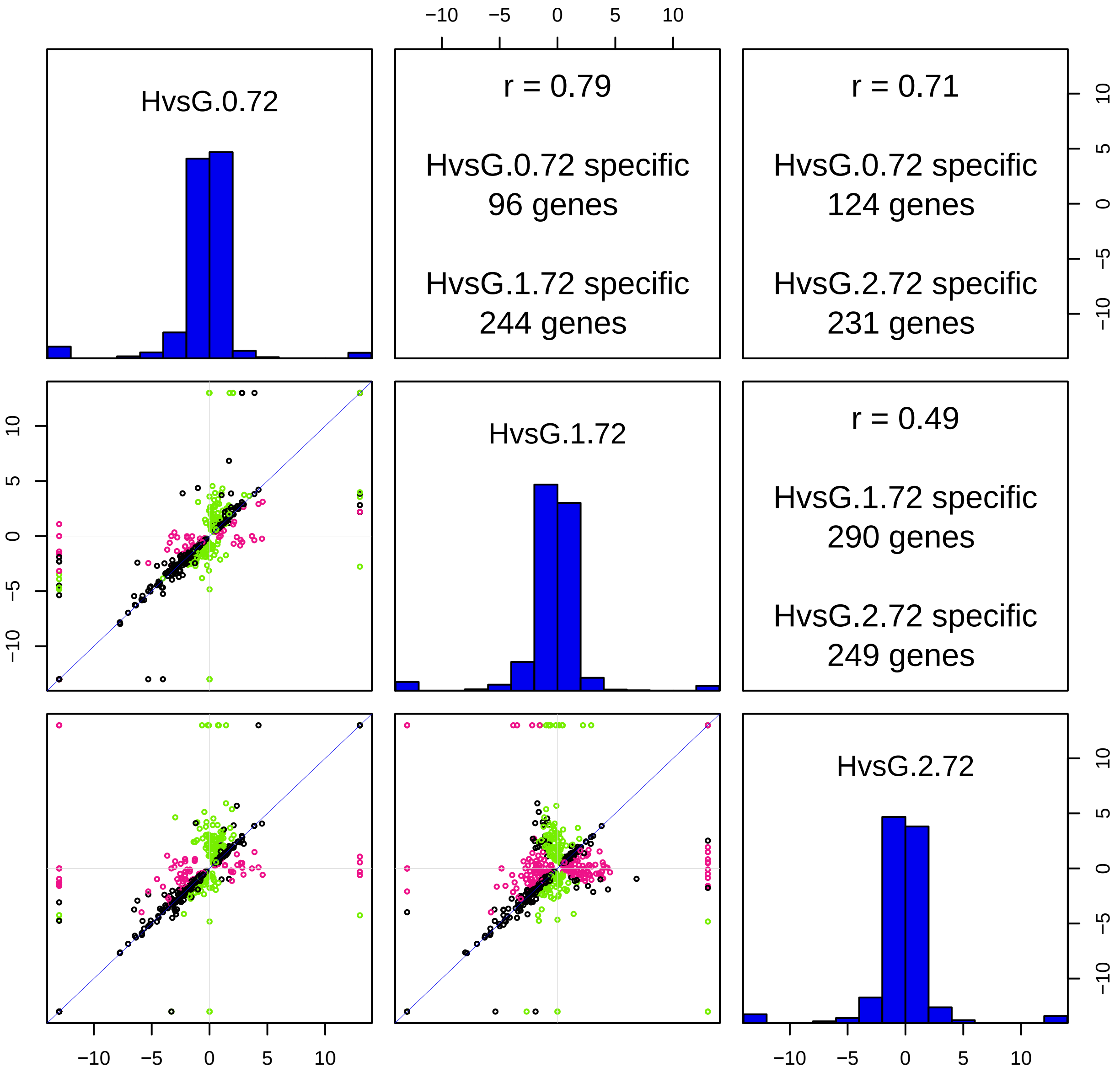
HvsG.0.72
r = 0.79
HvsG.0.72 specific
96 genes
HvsG.1.72 specific
244 genes
r = 0.71
HvsG.0.72 specific
124 genes
HvsG.2.72 specific
231 genes
HvsG.1.72
r = 0.49
HvsG.1.72 specific
290 genes
HvsG.2.72 specific
249 genes
HvsG.2.72
−10
−5
0
5
10

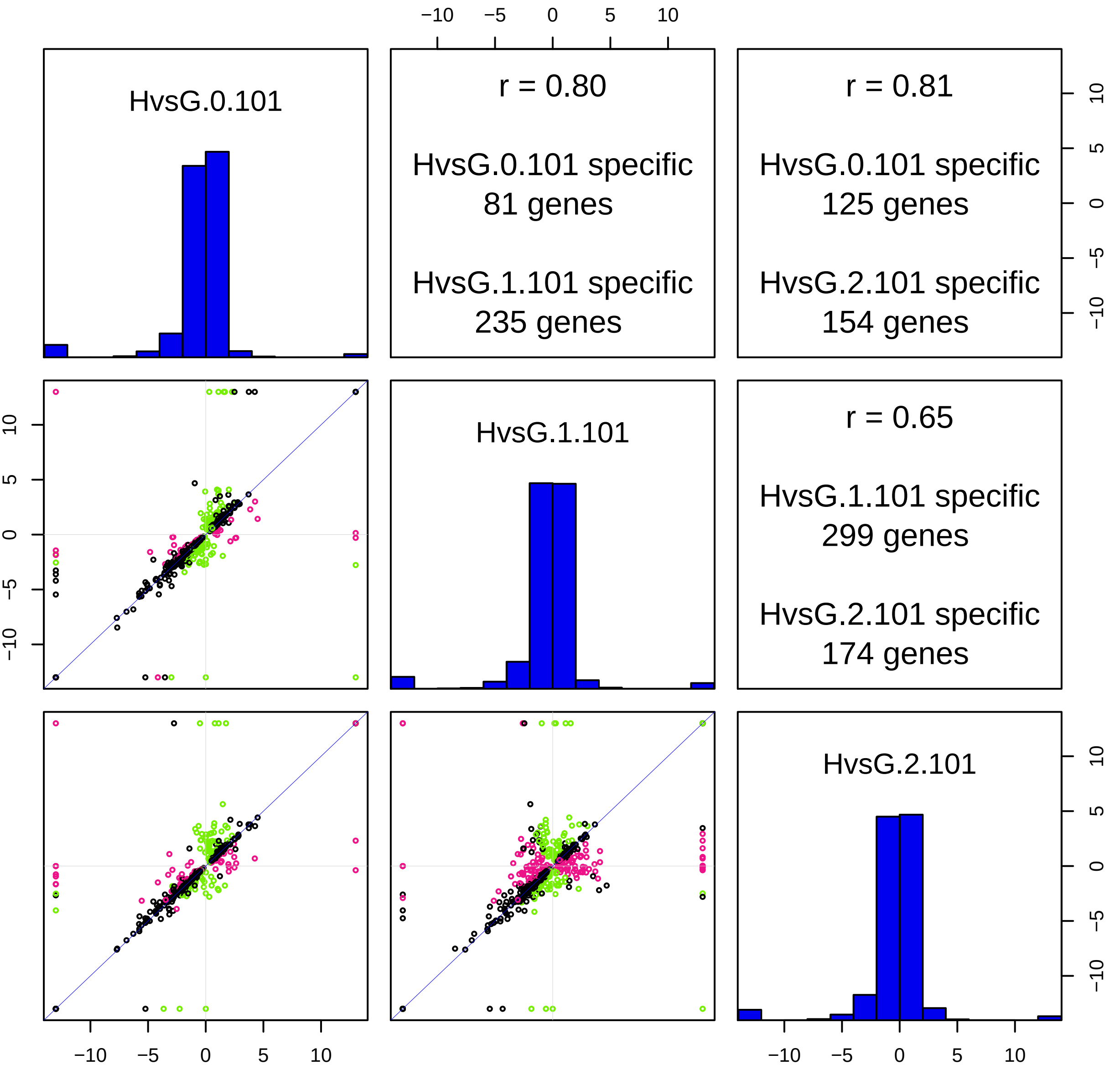
HvsG.0.101
r = 0.80
HvsG.0.101 specific
81 genes
HvsG.1.101 specific
235 genes
r = 0.81
HvsG.0.101 specific
125 genes
HvsG.2.101 specific
154 genes
HvsG.1.101
r = 0.65
HvsG.1.101 specific
299 genes
HvsG.2.101 specific
174 genes
HvsG.2.101
−10
−5
0
5
10

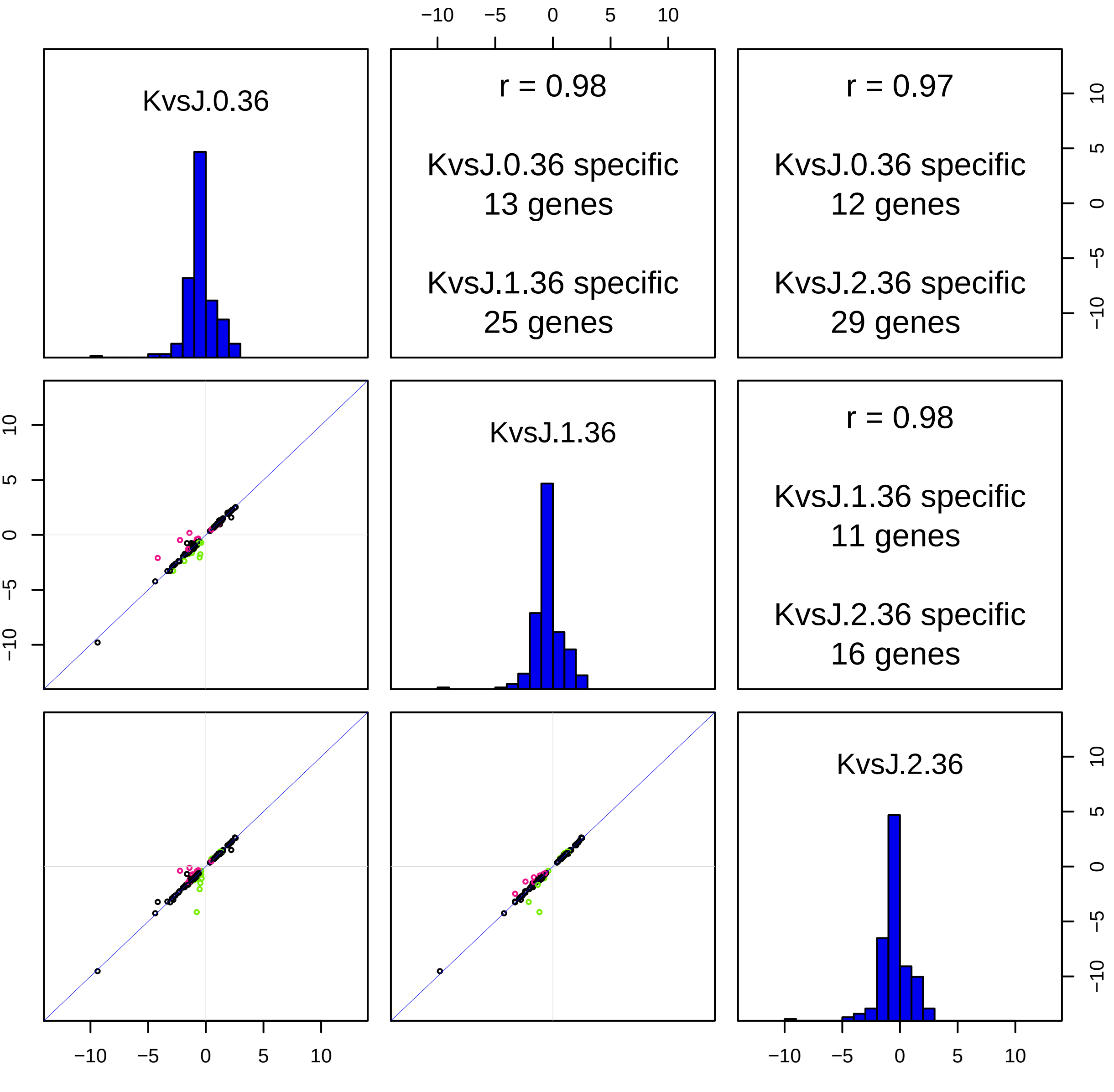
KvsJ.0.36
r = 0.98
KvsJ.0.36 specific
13 genes
KvsJ.1.36 specific
25 genes
r = 0.97
KvsJ.0.36 specific
12 genes
KvsJ.2.36 specific
29 genes
KvsJ.1.36
r = 0.98
KvsJ.1.36 specific
11 genes
KvsJ.2.36 specific
16 genes
KvsJ.2.36
−10
−5
0
5
10

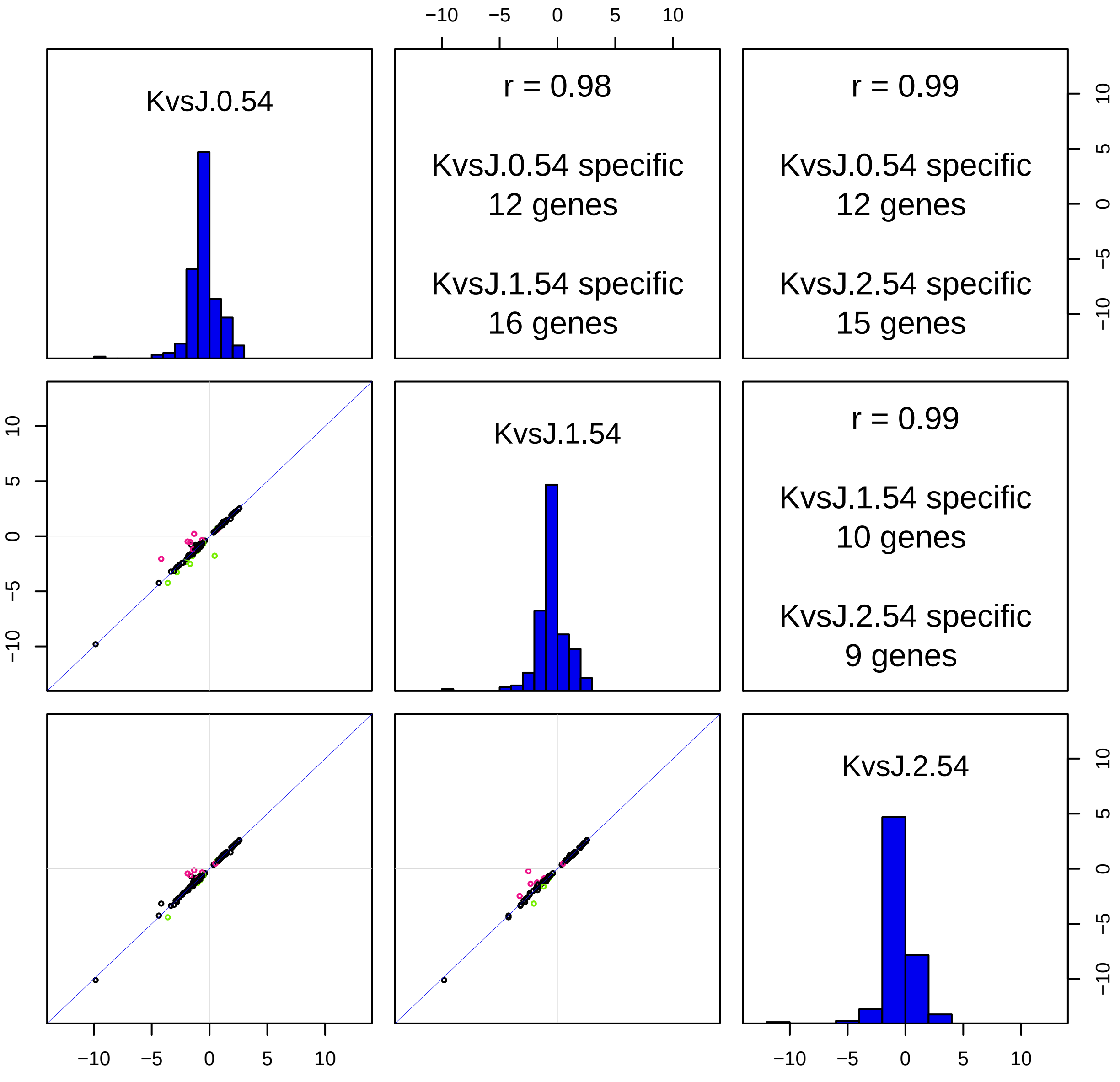

KvsJ.0.54
r = 0.98
KvsJ.0.54 specific
12 genes
KvsJ.1.54 specific
16 genes
r = 0.99
KvsJ.0.54 specific
12 genes
KvsJ.2.54 specific
15 genes
KvsJ.1.54
r = 0.99
KvsJ.1.54 specific
10 genes
KvsJ.2.54 specific
9 genes
KvsJ.2.54
−10
−5
0
5
10

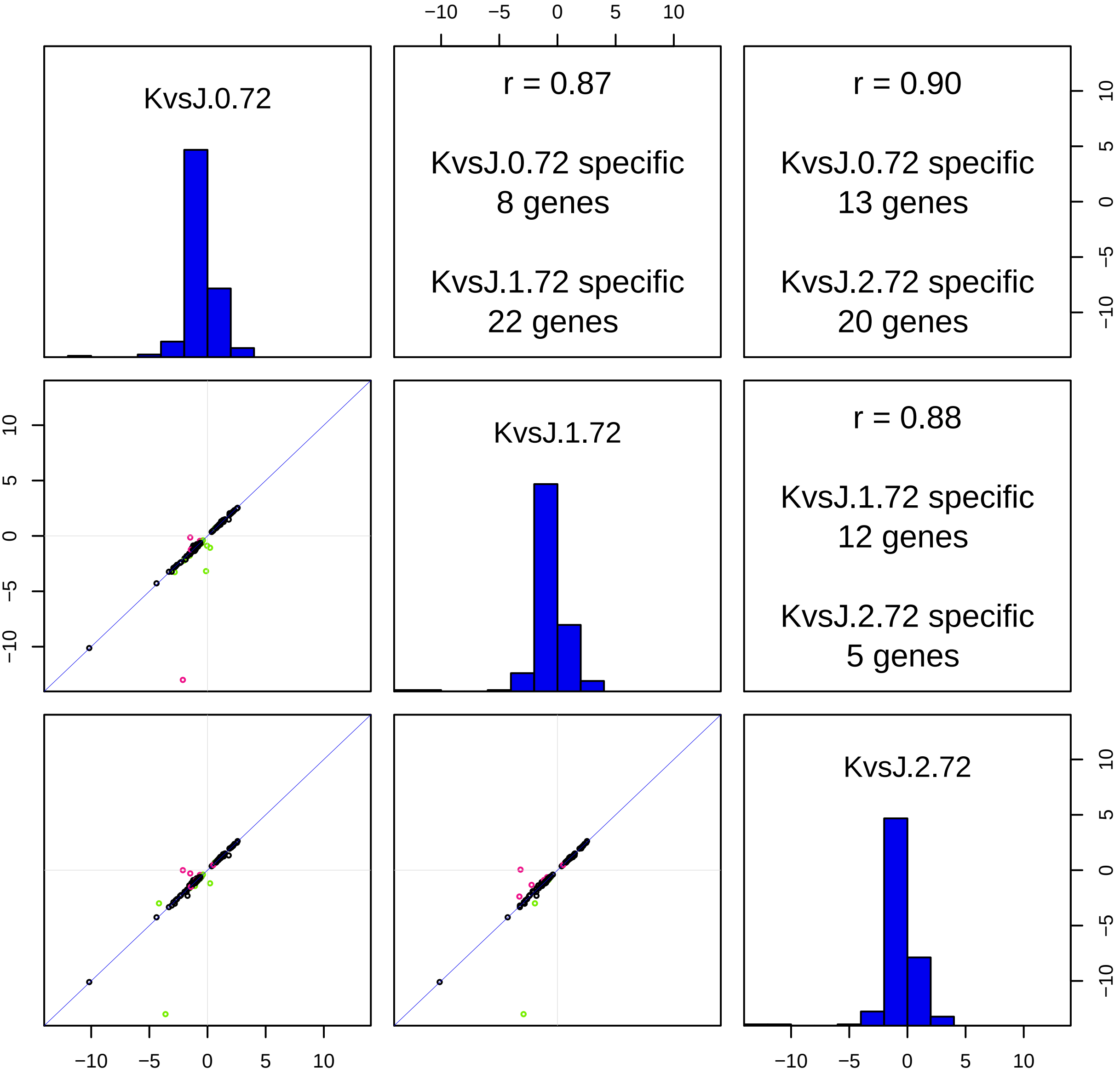
KvsJ.0.72
r = 0.87
KvsJ.0.72 specific
8 genes
KvsJ.1.72 specific
22 genes
r = 0.90
KvsJ.0.72 specific
13 genes
KvsJ.2.72 specific
20 genes
KvsJ.1.72
r = 0.88
KvsJ.1.72 specific
12 genes
KvsJ.2.72 specific
5 genes
KvsJ.2.72
−10
−5
0
5
10

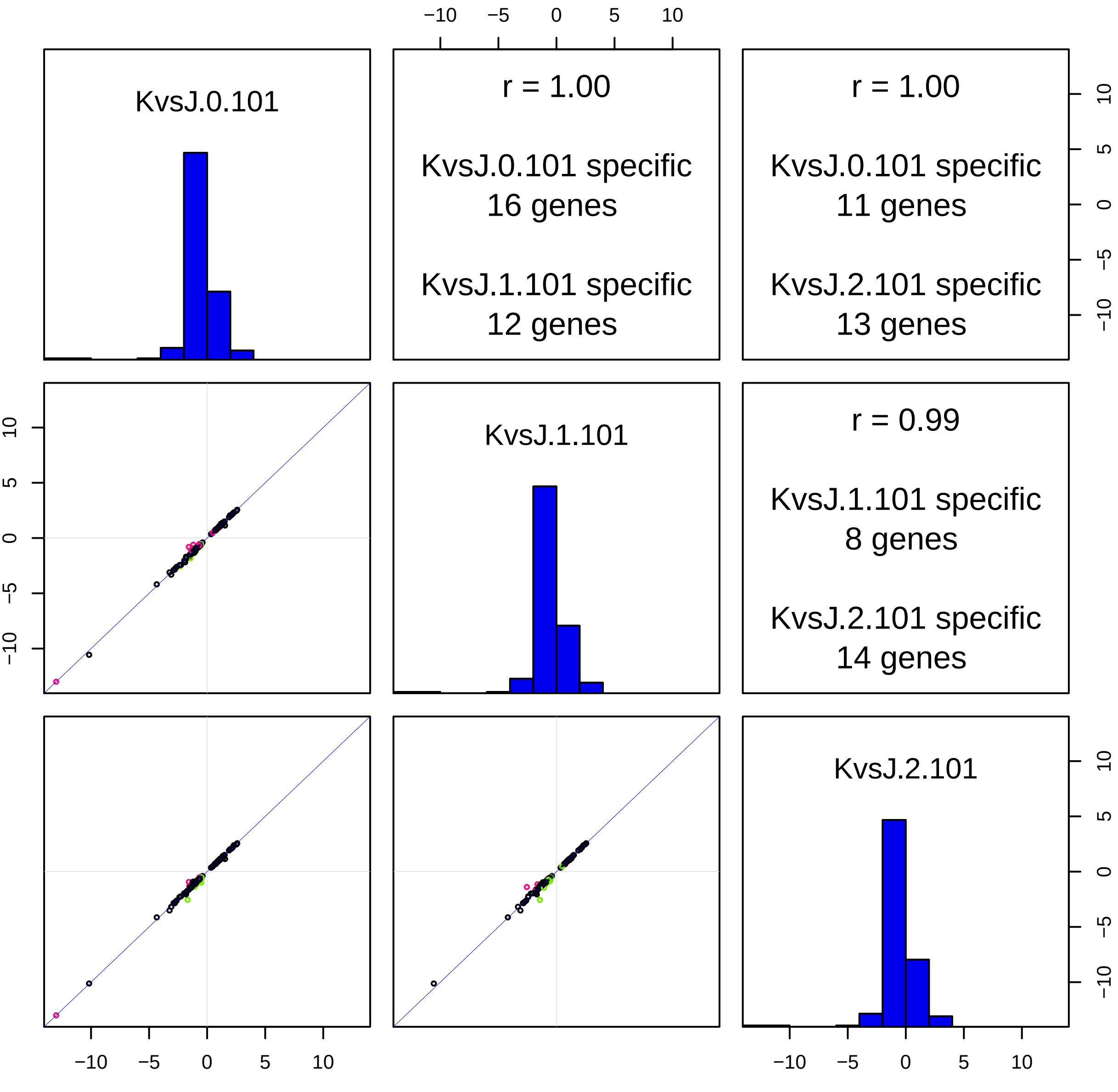
KvsJ.0.101
r = 1.00
KvsJ.0.101 specific
16 genes
KvsJ.1.101 specific
12 genes
r = 1.00
KvsJ.0.101 specific
11 genes
KvsJ.2.101 specific
13 genes
KvsJ.1.101
r = 0.99
KvsJ.1.101 specific
8 genes
KvsJ.2.101 specific
14 genes
KvsJ.2.101
−10
−5
0
5
10

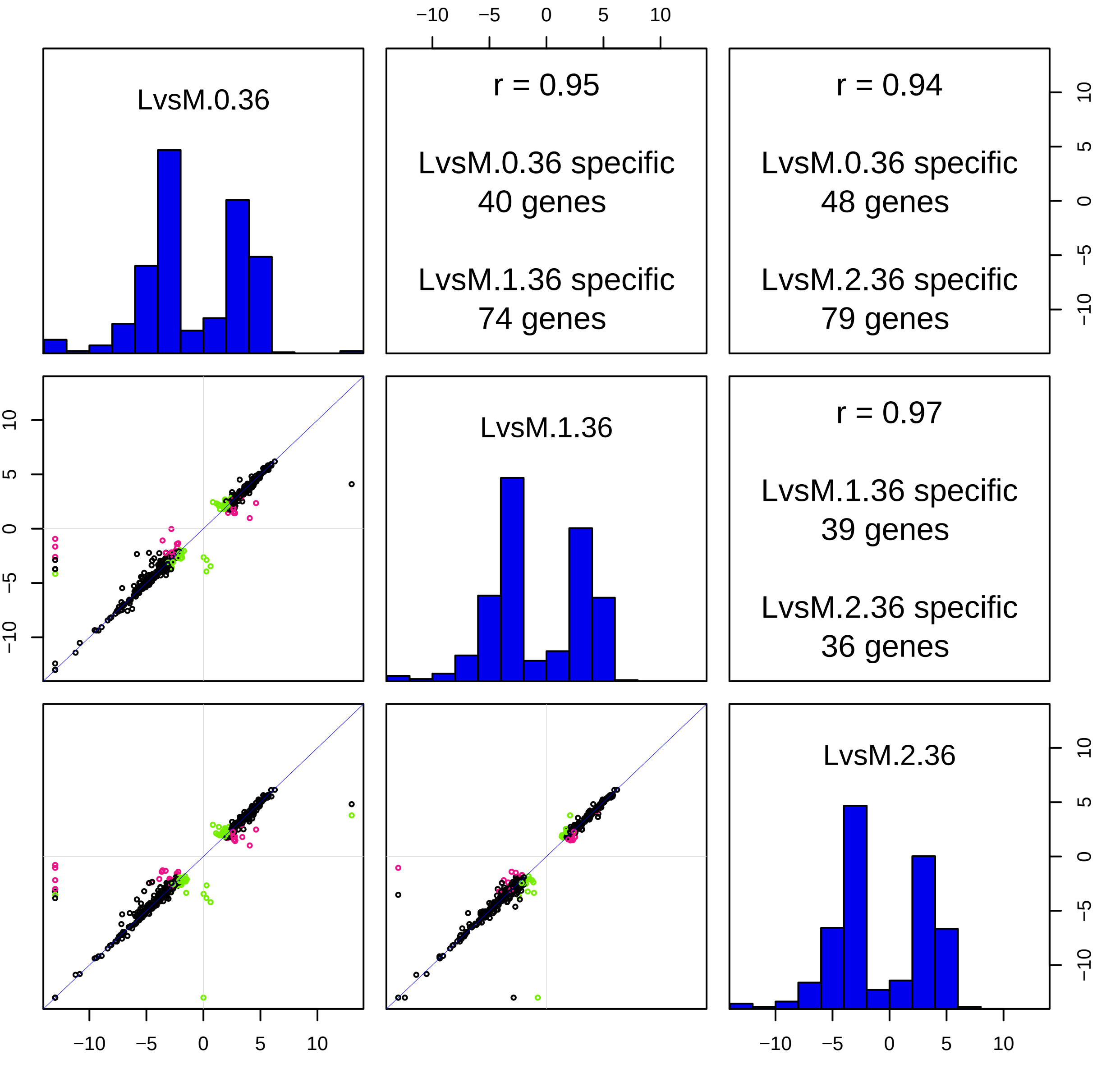
LvsM.0.36
r = 0.95
LvsM.0.36 specific
40 genes
LvsM.1.36 specific
74 genes
r = 0.94
LvsM.0.36 specific
48 genes
LvsM.2.36 specific
79 genes
LvsM.1.36
r = 0.97
LvsM.1.36 specific
39 genes
LvsM.2.36 specific
36 genes
LvsM.2.36
−10 −5 0 5 10

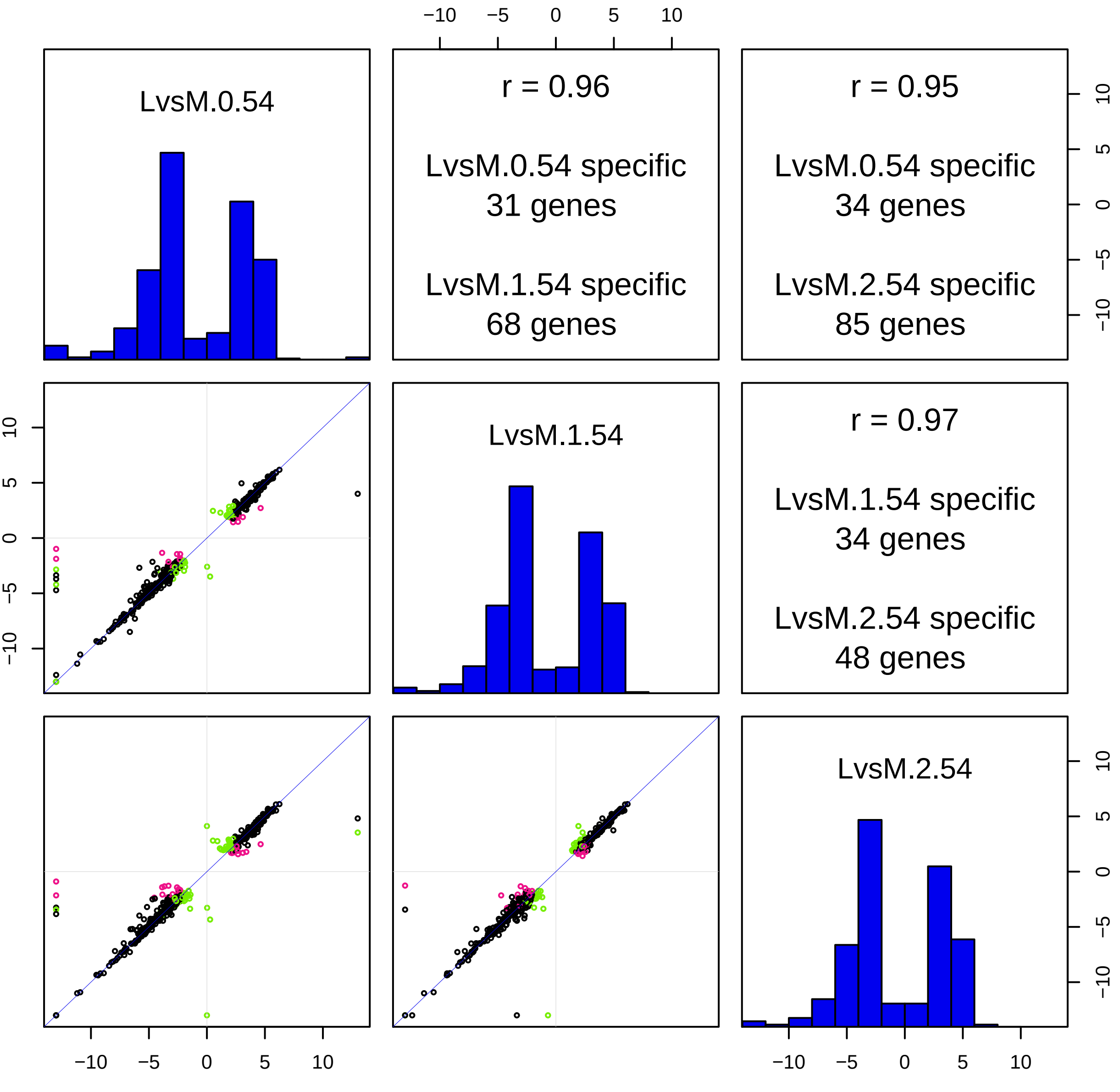

LvsM.0.54
r = 0.96
LvsM.0.54 specific
31 genes
LvsM.1.54 specific
68 genes
r = 0.95
LvsM.0.54 specific
34 genes
LvsM.2.54 specific
85 genes
LvsM.1.54
r = 0.97
LvsM.1.54 specific
34 genes
LvsM.2.54 specific
48 genes
LvsM.2.54
−10
−5
0
5
10

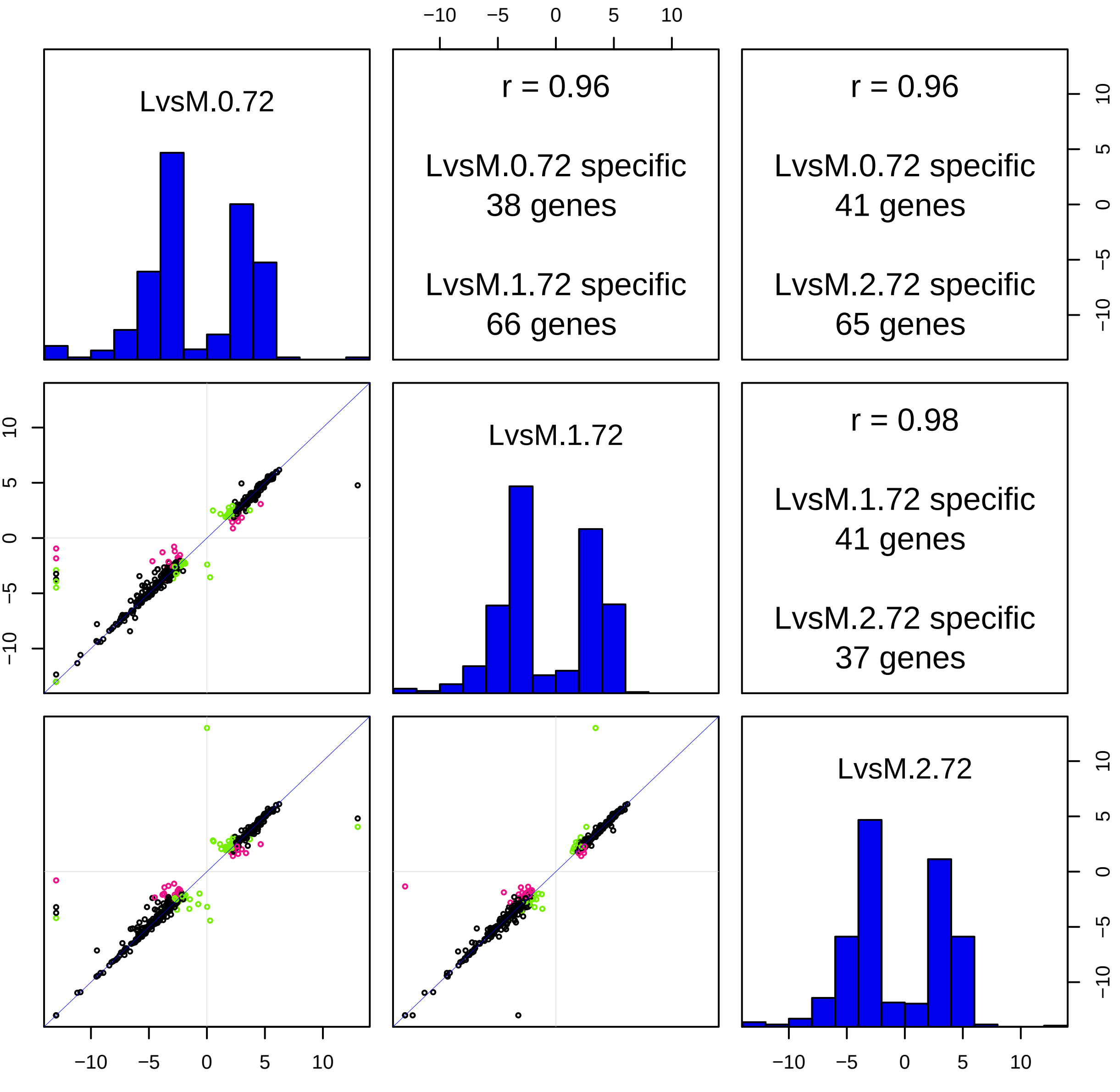

LvsM.0.72
r = 0.96
LvsM.0.72 specific
38 genes
LvsM.1.72 specific
66 genes
r = 0.96
LvsM.0.72 specific
41 genes
LvsM.2.72 specific
65 genes
LvsM.1.72
r = 0.98
LvsM.1.72 specific
41 genes
LvsM.2.72 specific
37 genes
LvsM.2.72
−10
−5
0
5
10

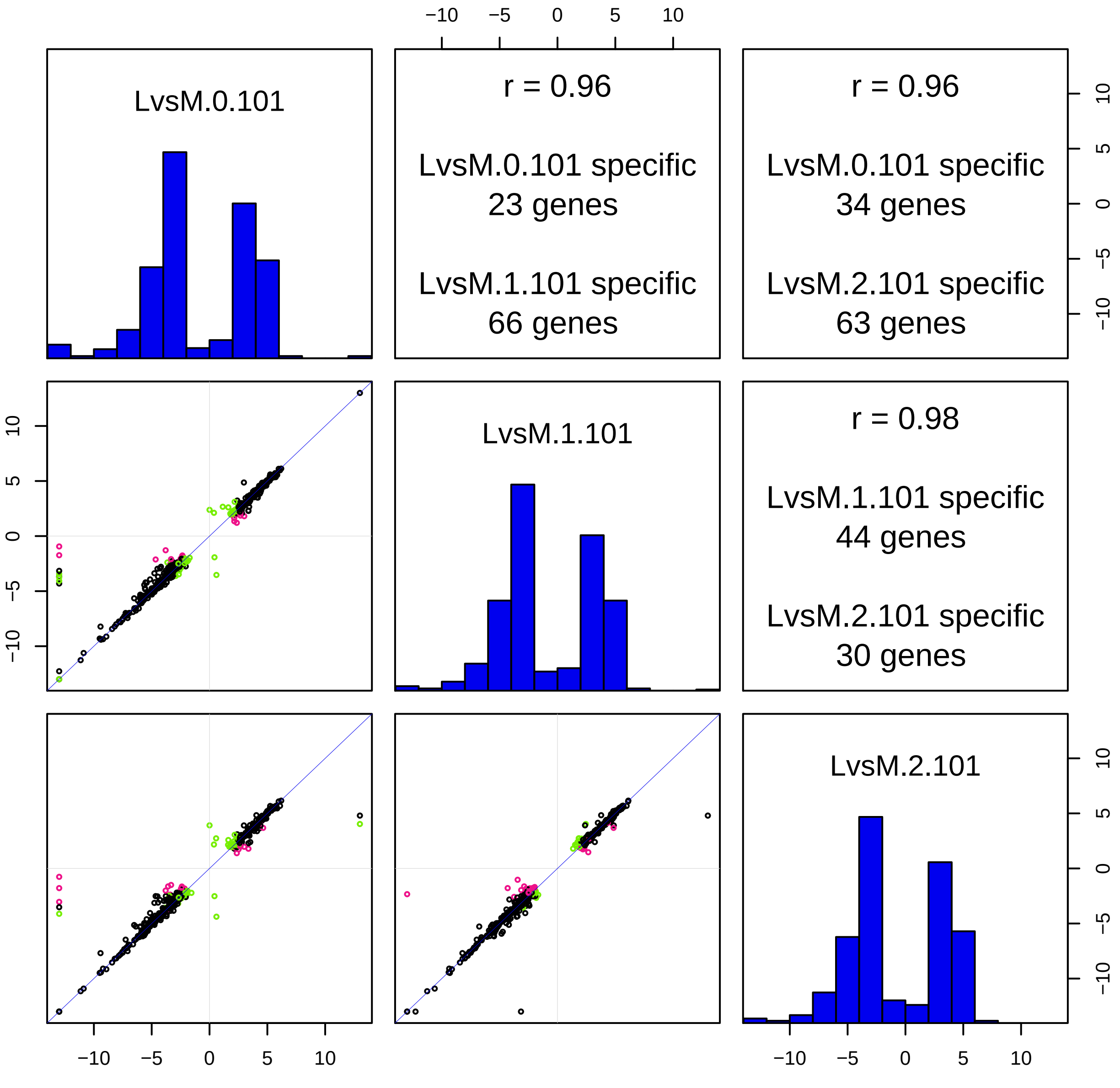
LvsM.0.101
r = 0.96
LvsM.0.101 specific
23 genes
LvsM.1.101 specific
66 genes
r = 0.96
LvsM.0.101 specific
34 genes
LvsM.2.101 specific
63 genes
LvsM.1.101
r = 0.98
LvsM.1.101 specific
44 genes
LvsM.2.101 specific
30 genes
LvsM.2.101
−10
−5
0
5
10

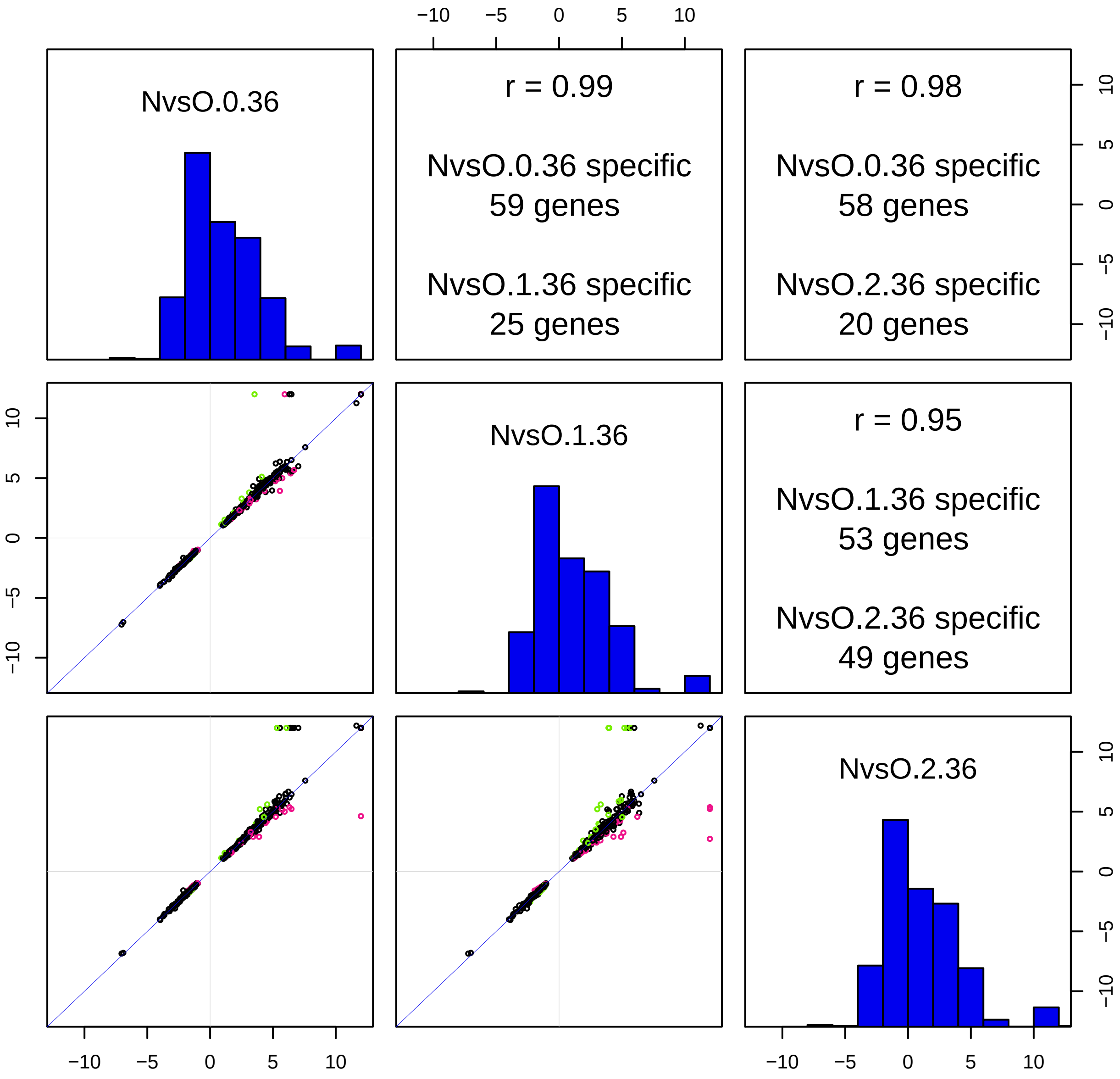

NvsO.0.36
r = 0.99
NvsO.0.36 specific
59 genes
NvsO.1.36 specific
25 genes
r = 0.98
NvsO.0.36 specific
58 genes
NvsO.2.36 specific
20 genes
NvsO.1.36
r = 0.95
NvsO.1.36 specific
53 genes
NvsO.2.36 specific
49 genes
NvsO.2.36
−10
−5
0
5
10

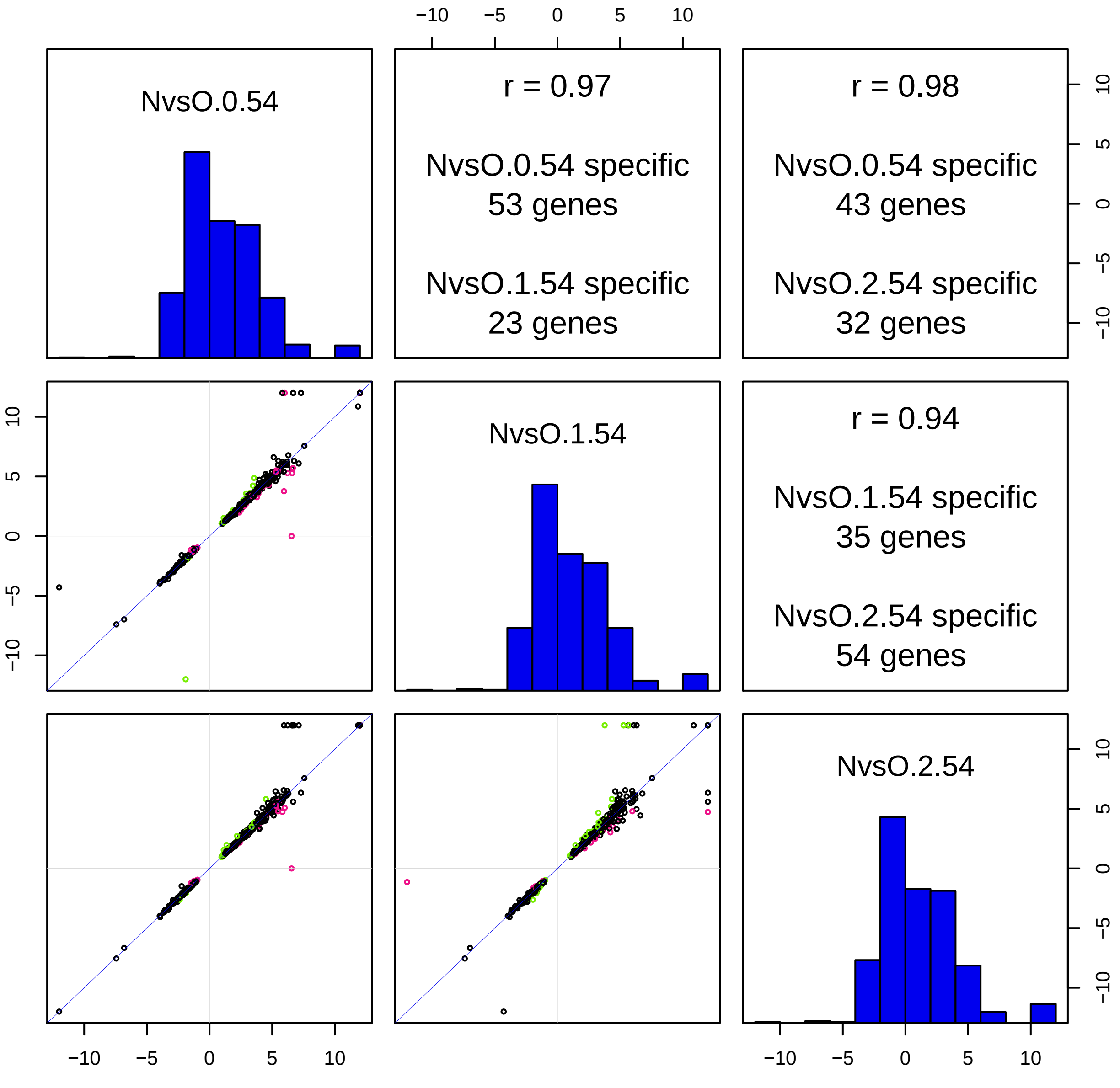

NvsO.0.54
r = 0.97
NvsO.0.54 specific
53 genes
NvsO.1.54 specific
23 genes
r = 0.98
NvsO.0.54 specific
43 genes
NvsO.2.54 specific
32 genes
NvsO.1.54
r = 0.94
NvsO.1.54 specific
35 genes
NvsO.2.54 specific
54 genes
NvsO.2.54
−10
−5
0
5
10

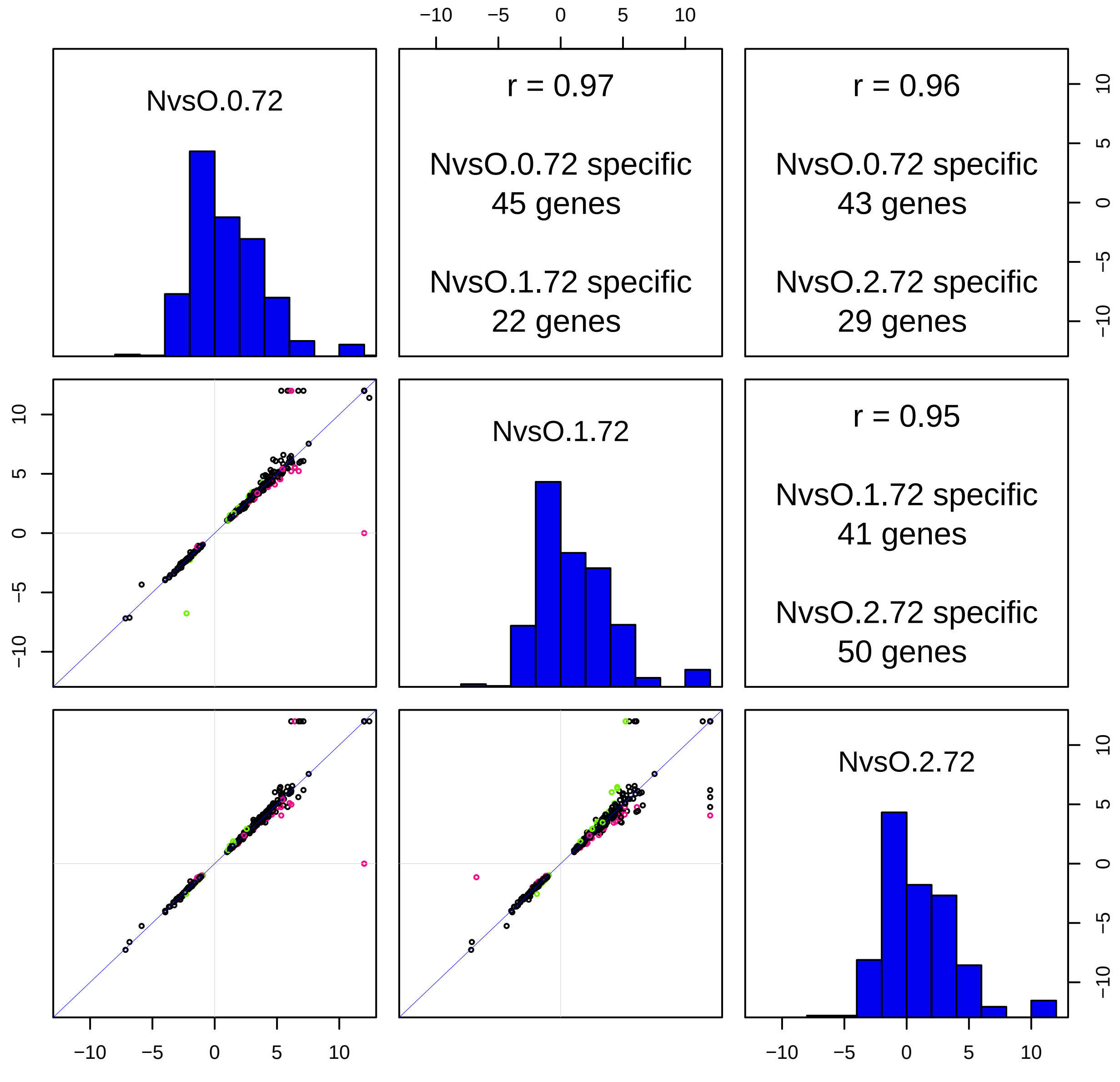
NvsO.0.72
r = 0.97
NvsO.0.72 specific
45 genes
NvsO.1.72 specific
22 genes
r = 0.96
NvsO.0.72 specific
43 genes
NvsO.2.72 specific
29 genes
NvsO.1.72
r = 0.95
NvsO.1.72 specific
41 genes
NvsO.2.72 specific
50 genes
NvsO.2.72
−10
−5
0
5
10

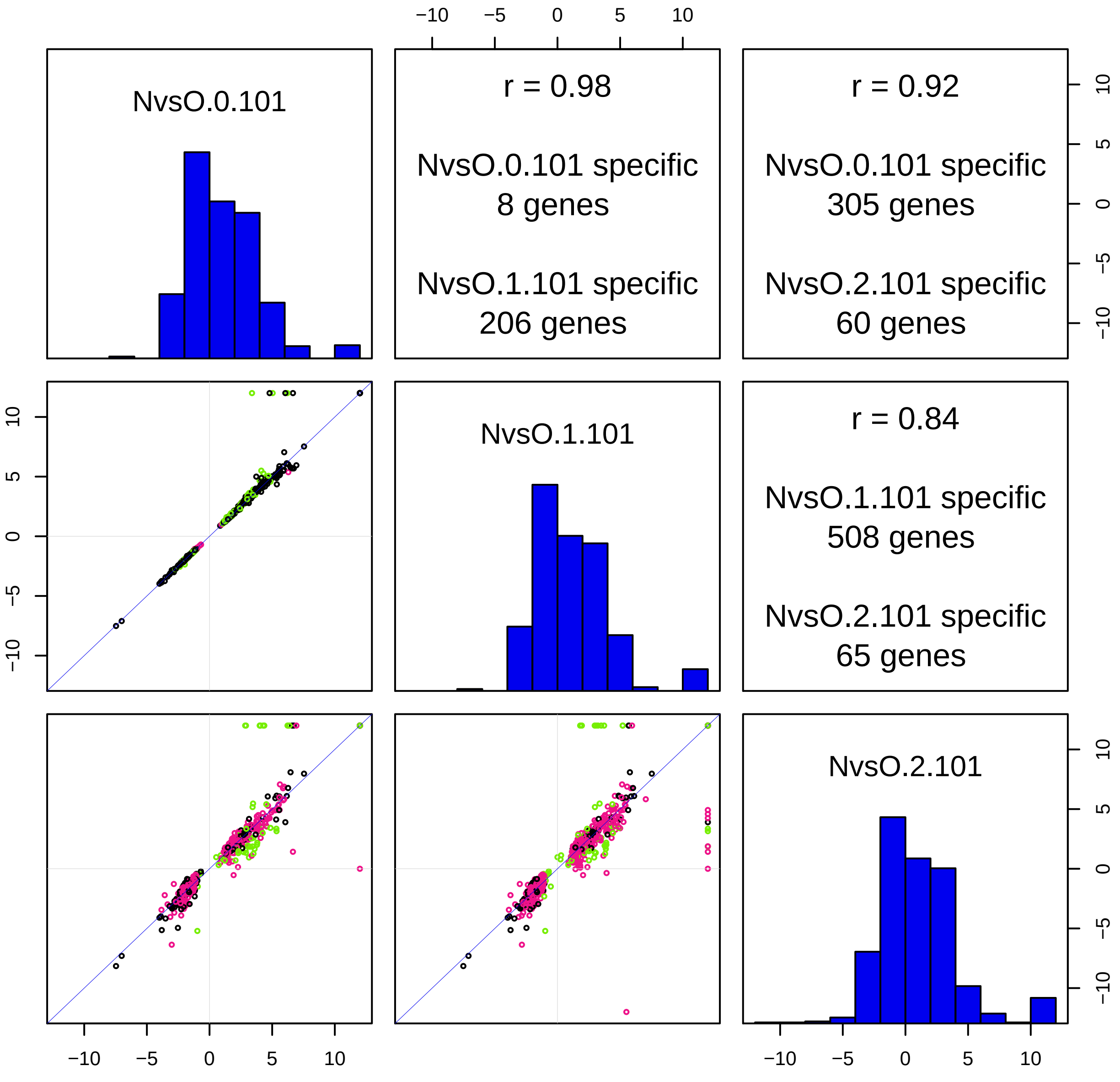

NvsO.0.101
r = 0.98
NvsO.0.101 specific
8 genes
NvsO.1.101 specific
206 genes
r = 0.92
NvsO.0.101 specific
305 genes
NvsO.2.101 specific
60 genes
NvsO.1.101
r = 0.84
NvsO.1.101 specific
508 genes
NvsO.2.101 specific
65 genes
NvsO.2.101
−10
−5
0
5
10

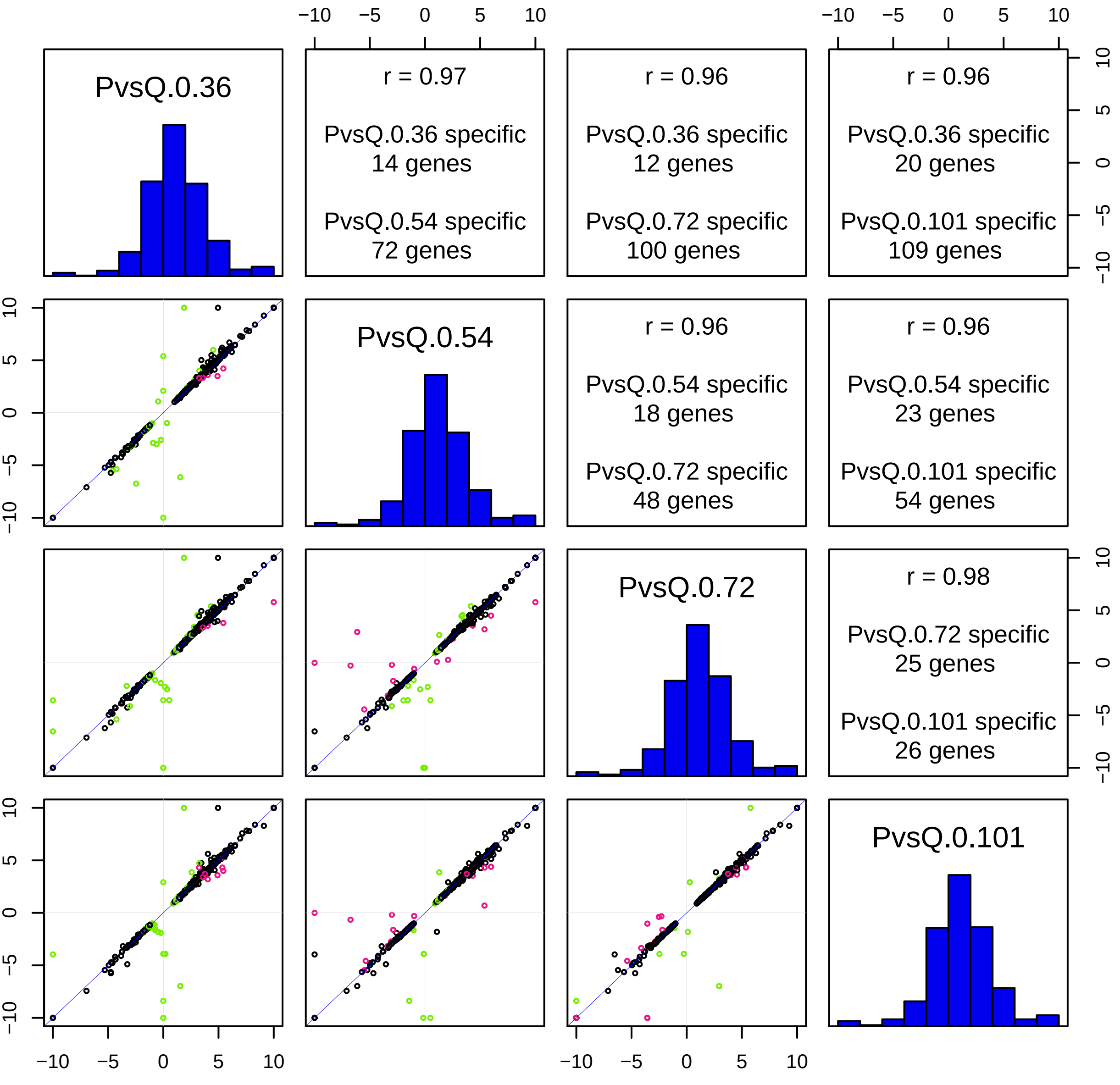
PvsQ.0.36
r = 0.97
PvsQ.0.36 specific
14 genes
PvsQ.0.54 specific
72 genes
r = 0.96
PvsQ.0.36 specific
12 genes
PvsQ.0.72 specific
100 genes
r = 0.96
PvsQ.0.36 specific
20 genes
PvsQ.0.101 specific
109 genes
PvsQ.0.54
r = 0.96
PvsQ.0.54 specific
18 genes
PvsQ.0.72 specific
48 genes
r = 0.96
PvsQ.0.54 specific
23 genes
PvsQ.0.101 specific
54 genes
PvsQ.0.72
r = 0.98
PvsQ.0.72 specific
25 genes
PvsQ.0.101 specific
26 genes
PvsQ.0.101
−10
−5
0
5
10

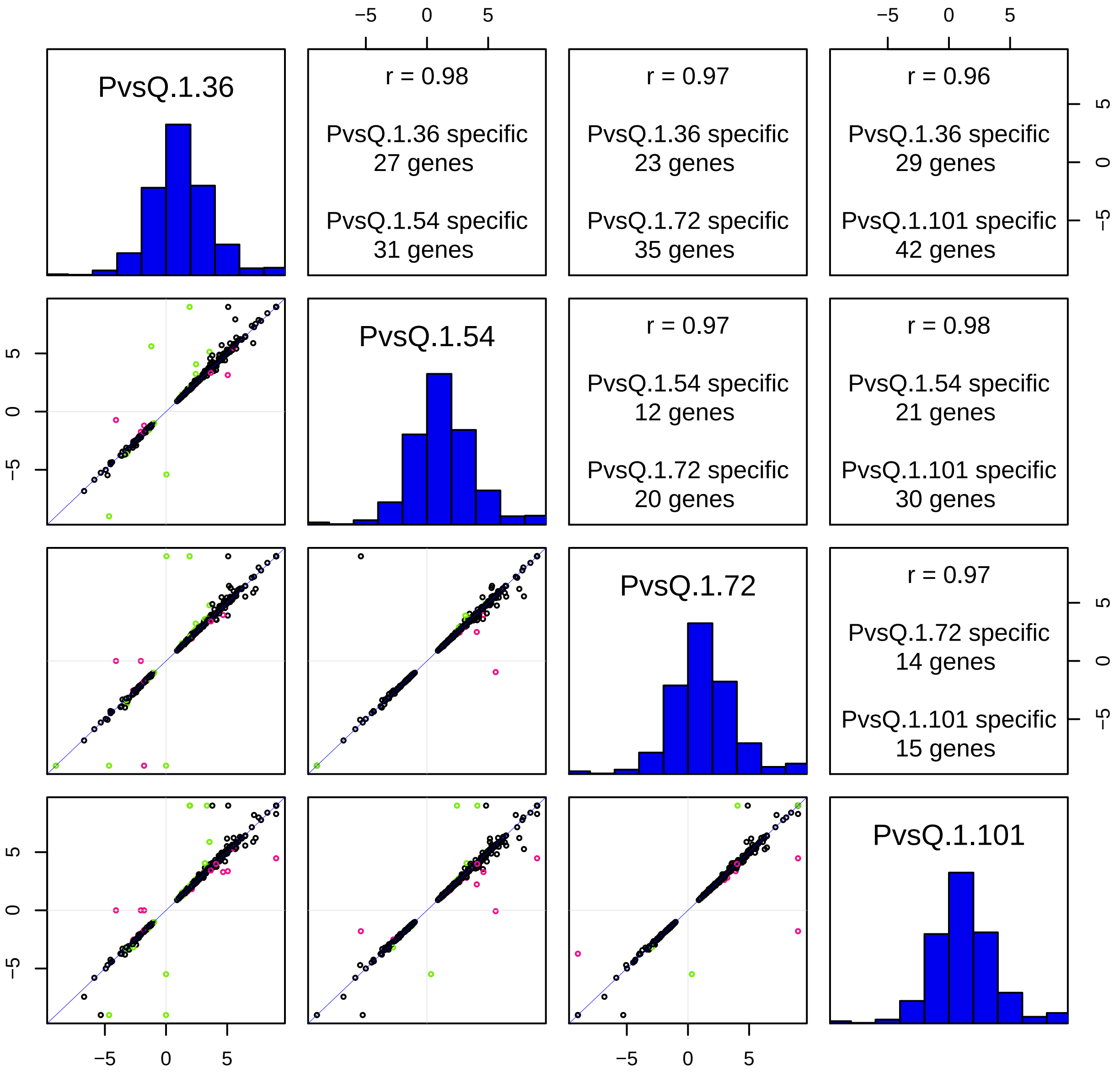
PvsQ.1.36
r = 0.98
PvsQ.1.36 specific
27 genes
PvsQ.1.54 specific
31 genes
r = 0.97
PvsQ.1.36 specific
23 genes
PvsQ.1.72 specific
35 genes
r = 0.96
PvsQ.1.36 specific
29 genes
PvsQ.1.101 specific
42 genes
PvsQ.1.54
r = 0.97
PvsQ.1.54 specific
12 genes
PvsQ.1.72 specific
20 genes
r = 0.98
PvsQ.1.54 specific
21 genes
PvsQ.1.101 specific
30 genes
PvsQ.1.72
r = 0.97
PvsQ.1.72 specific
14 genes
PvsQ.1.101 specific
15 genes
PvsQ.1.101
−5
0
5

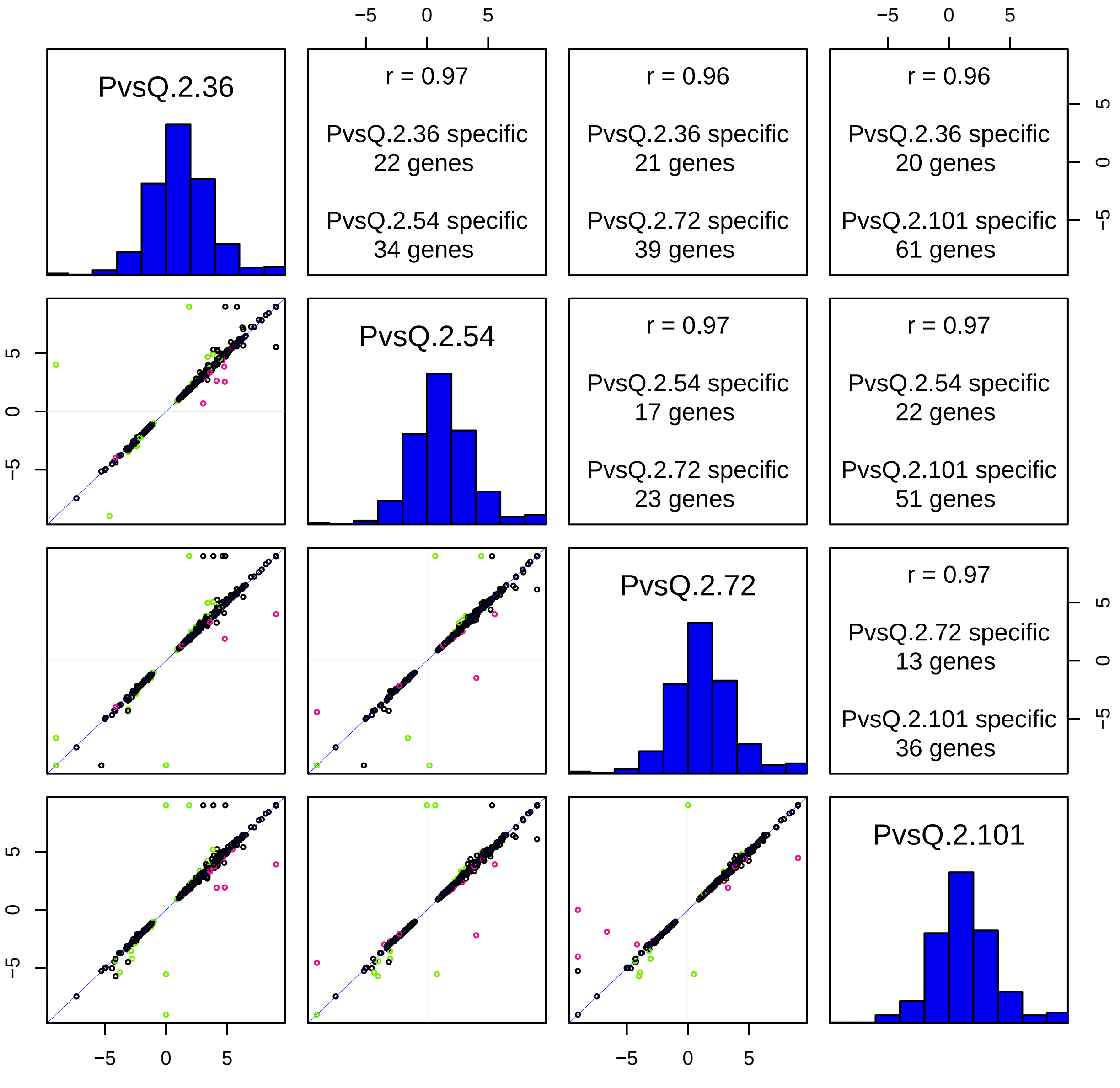

PvsQ.2.36
r = 0.97
PvsQ.2.36 specific
22 genes
PvsQ.2.54 specific
34 genes
r = 0.96
PvsQ.2.36 specific
21 genes
PvsQ.2.72 specific
39 genes
r = 0.96
PvsQ.2.36 specific
20 genes
PvsQ.2.101 specific
61 genes
PvsQ.2.54
r = 0.97
PvsQ.2.54 specific
17 genes
PvsQ.2.72 specific
23 genes
r = 0.97
PvsQ.2.54 specific
22 genes
PvsQ.2.101 specific
51 genes
PvsQ.2.72
r = 0.97
PvsQ.2.72 specific
13 genes
PvsQ.2.101 specific
36 genes
PvsQ.2.101
−5
0
5

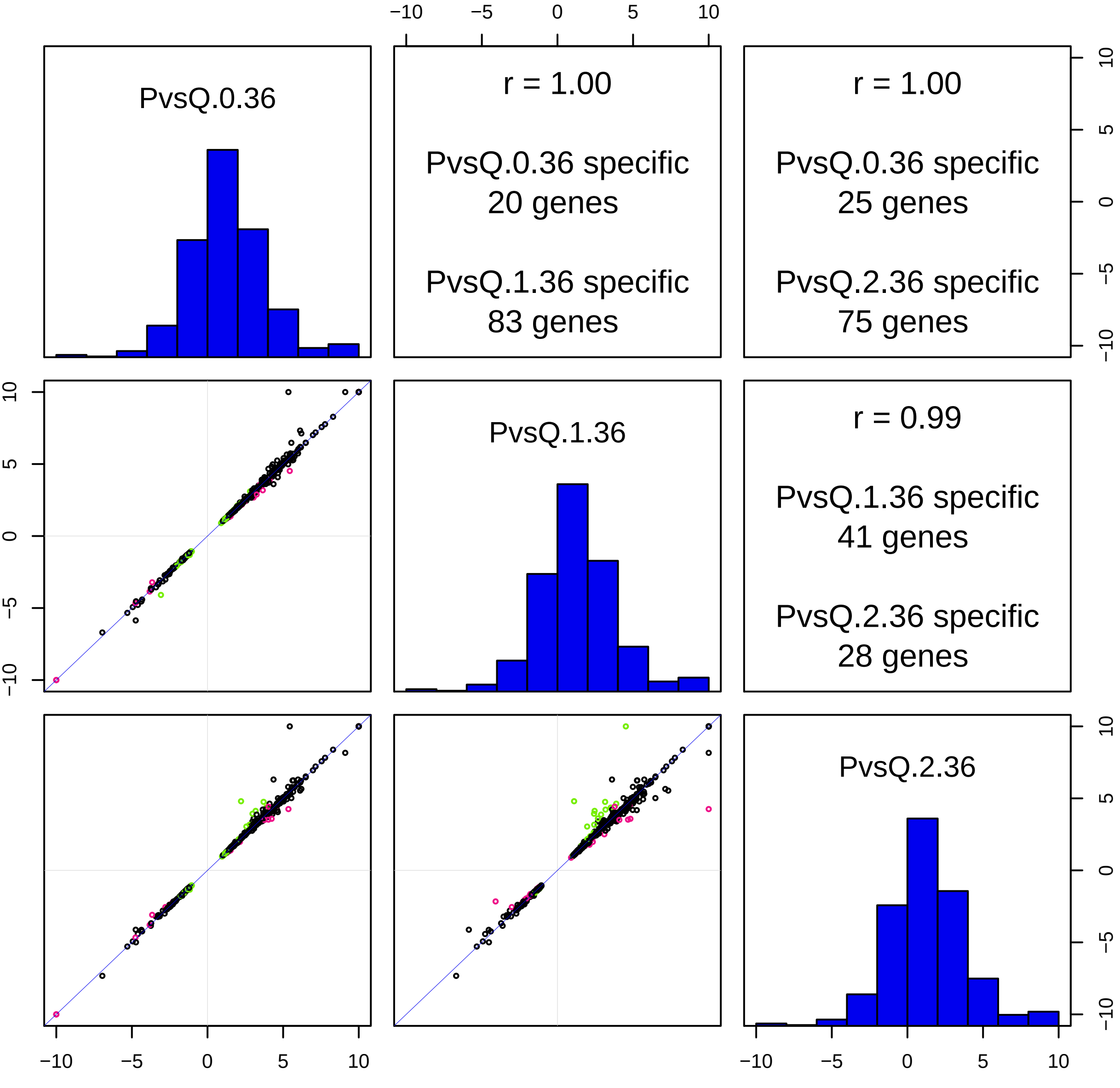

PvsQ.0.36
r = 1.00
PvsQ.0.36 specific
20 genes
PvsQ.1.36 specific
83 genes
r = 1.00
PvsQ.0.36 specific
25 genes
PvsQ.2.36 specific
75 genes
PvsQ.1.36
r = 0.99
PvsQ.1.36 specific
41 genes
PvsQ.2.36 specific
28 genes
PvsQ.2.36
−10
−5
0
5
10

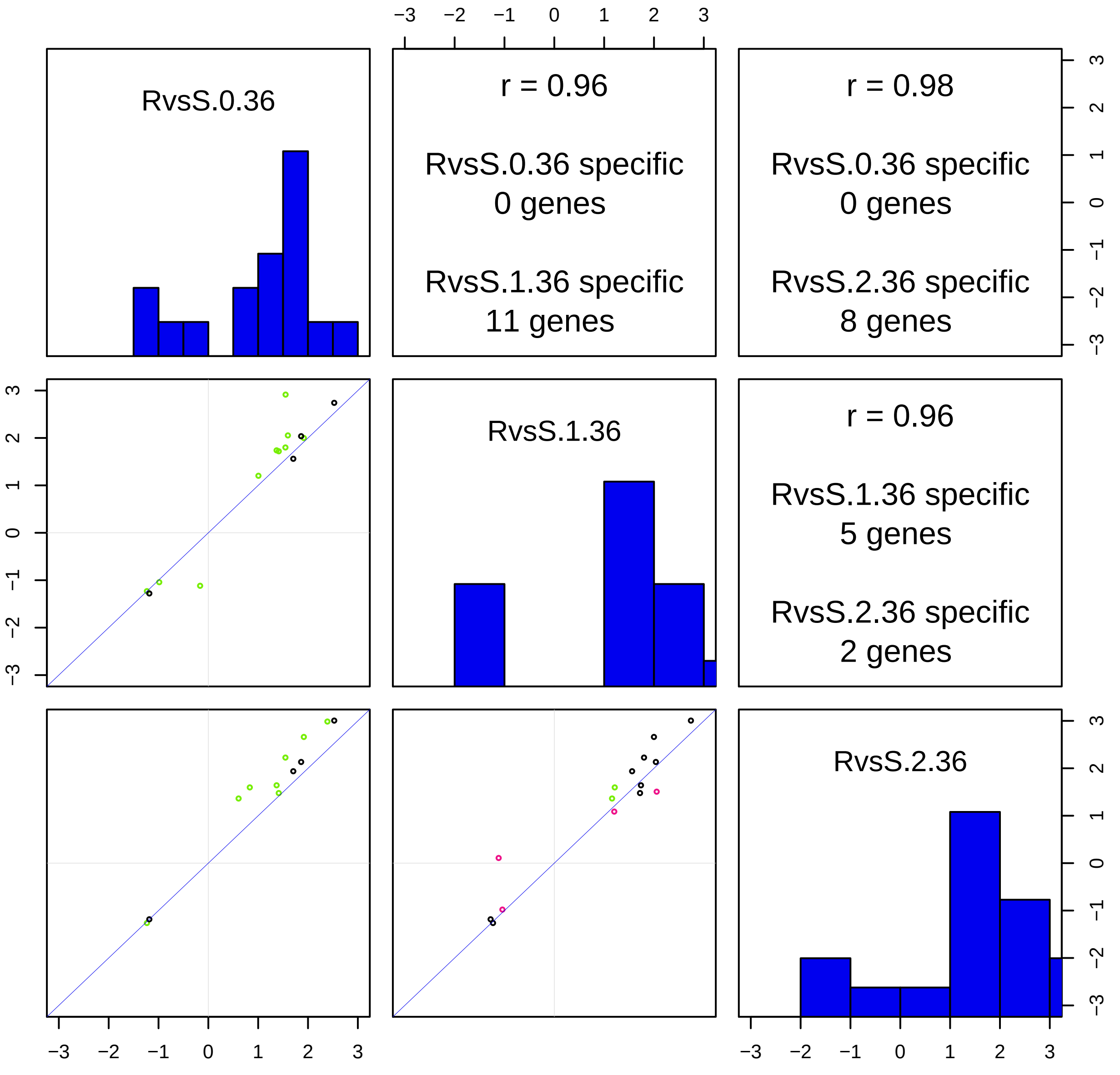

RvsS.0.36
r = 0.96
RvsS.0.36 specific
0 genes
RvsS.1.36 specific
11 genes
r = 0.98
RvsS.0.36 specific
0 genes
RvsS.2.36 specific
8 genes
RvsS.1.36
r = 0.96
RvsS.1.36 specific
5 genes
RvsS.2.36 specific
2 genes
RvsS.2.36
−3 −2 −1 0 1 2 3

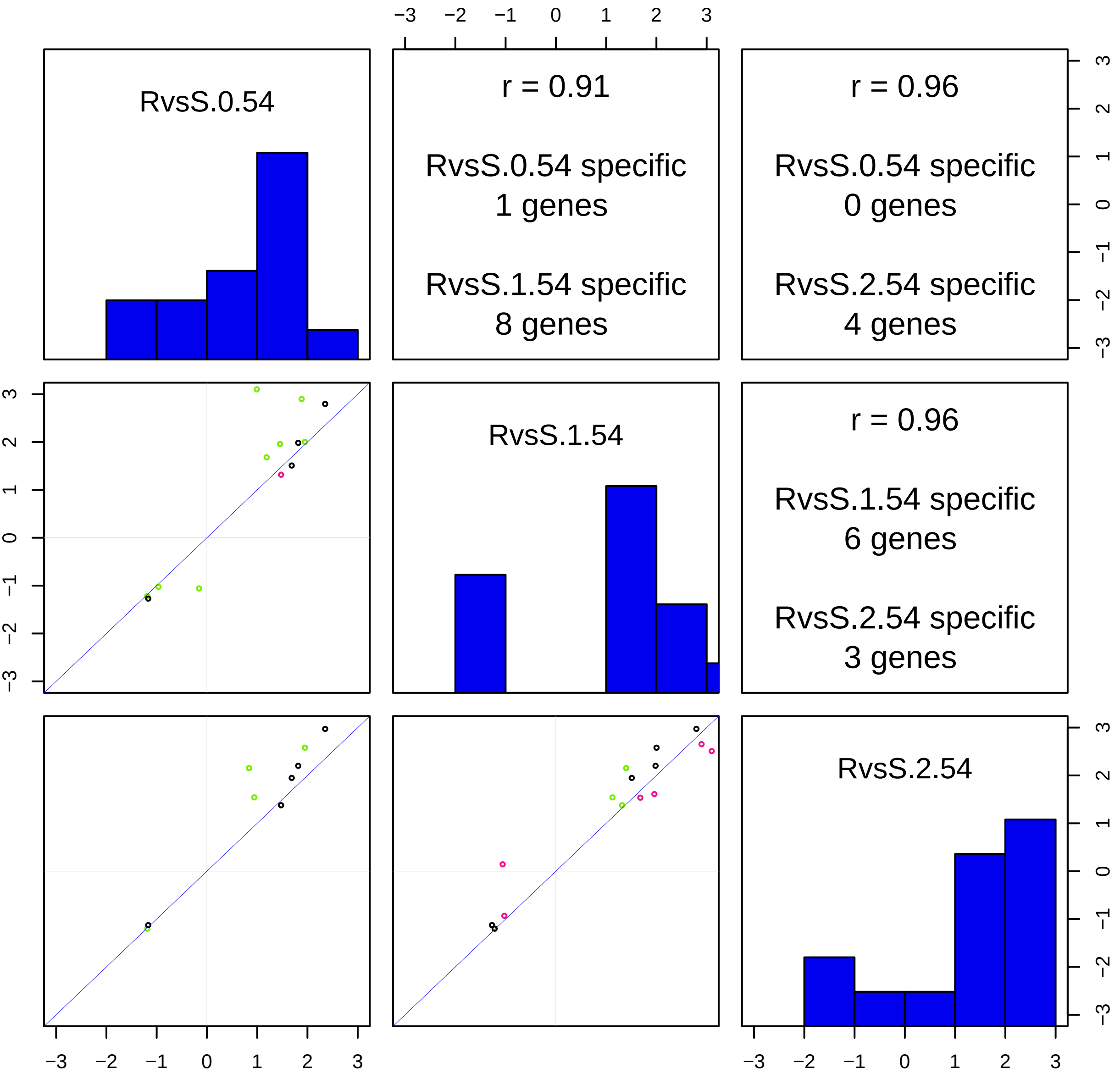
RvsS.0.54
r = 0.91
RvsS.0.54 specific
1 genes
RvsS.1.54 specific
8 genes
r = 0.96
RvsS.0.54 specific
0 genes
RvsS.2.54 specific
4 genes
RvsS.1.54
r = 0.96
RvsS.1.54 specific
6 genes
RvsS.2.54 specific
3 genes
RvsS.2.54
−3 −2 −1 0 1 2 3

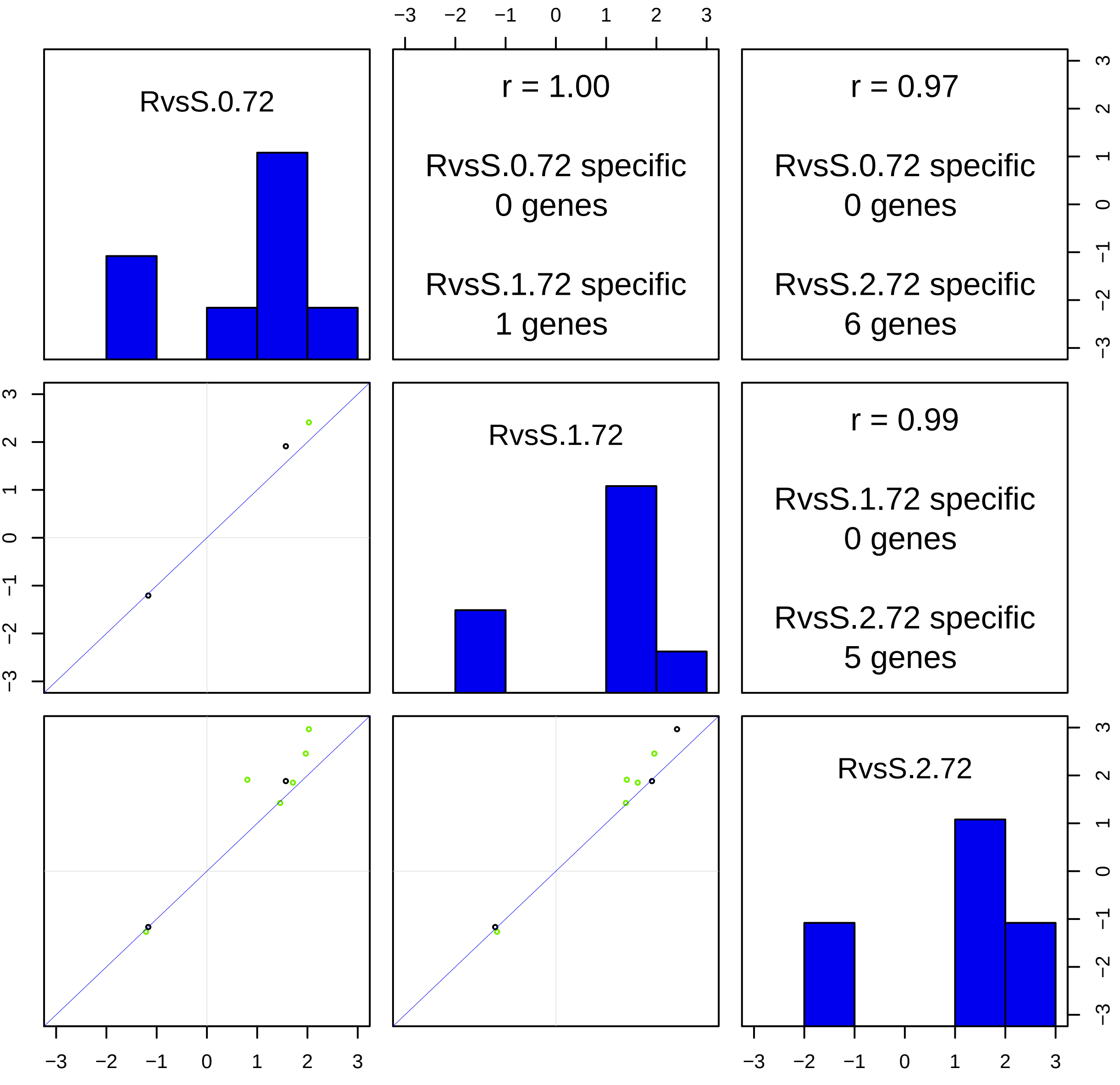
RvsS.0.72
r = 1.00
RvsS.0.72 specific
0 genes
RvsS.1.72 specific
1 genes
r = 0.97
RvsS.0.72 specific
0 genes
RvsS.2.72 specific
6 genes
RvsS.1.72
r = 0.99
RvsS.1.72 specific
0 genes
RvsS.2.72 specific
5 genes
RvsS.2.72
−3 −2 −1 0 1 2 3

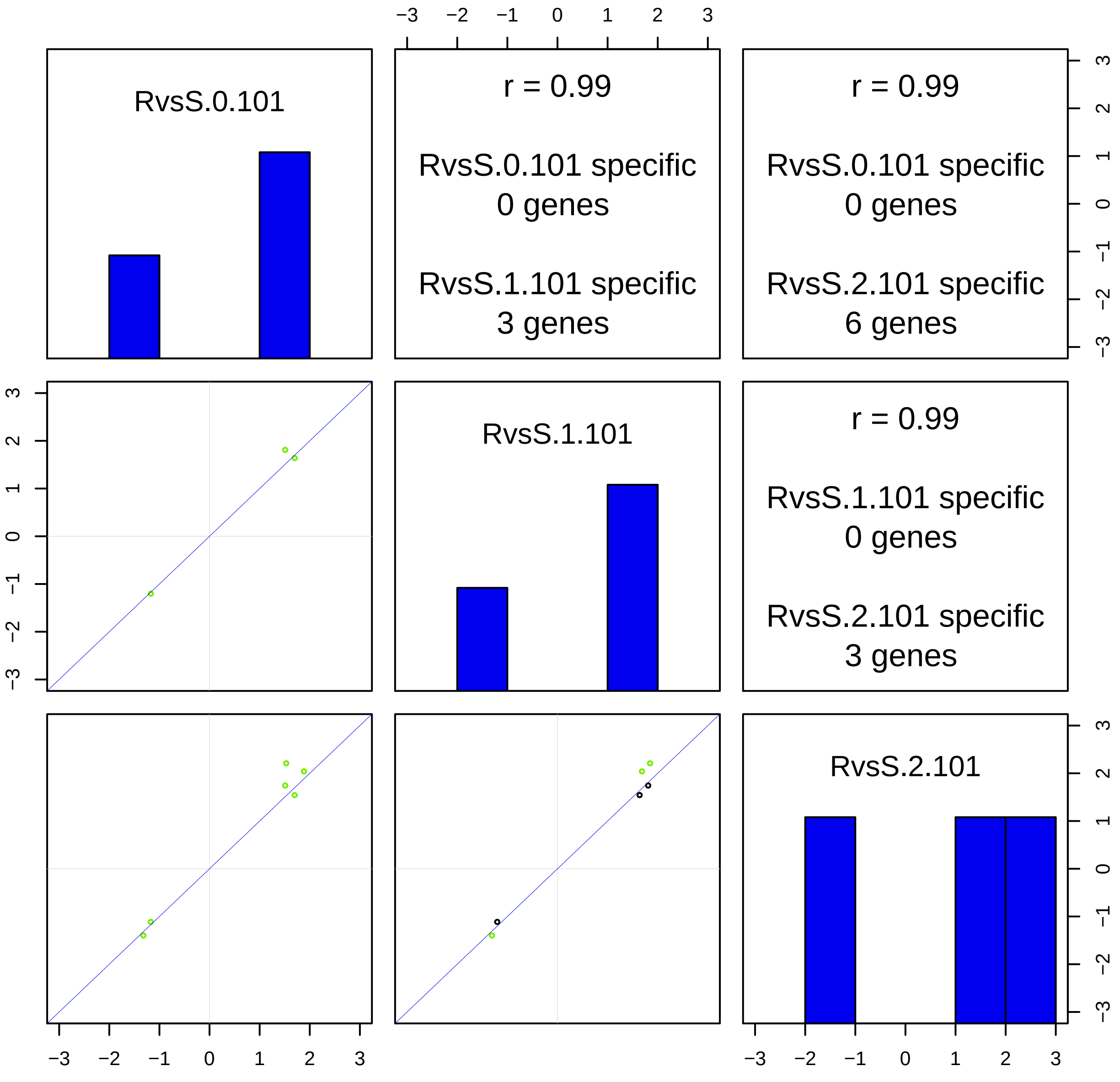

RvsS.0.101
r = 0.99
RvsS.0.101 specific
0 genes
RvsS.1.101 specific
3 genes
r = 0.99
RvsS.0.101 specific
0 genes
RvsS.2.101 specific
6 genes
RvsS.1.101
r = 0.99
RvsS.1.101 specific
0 genes
RvsS.2.101 specific
3 genes
RvsS.2.101
−3 −2 −1 0 1 2 3

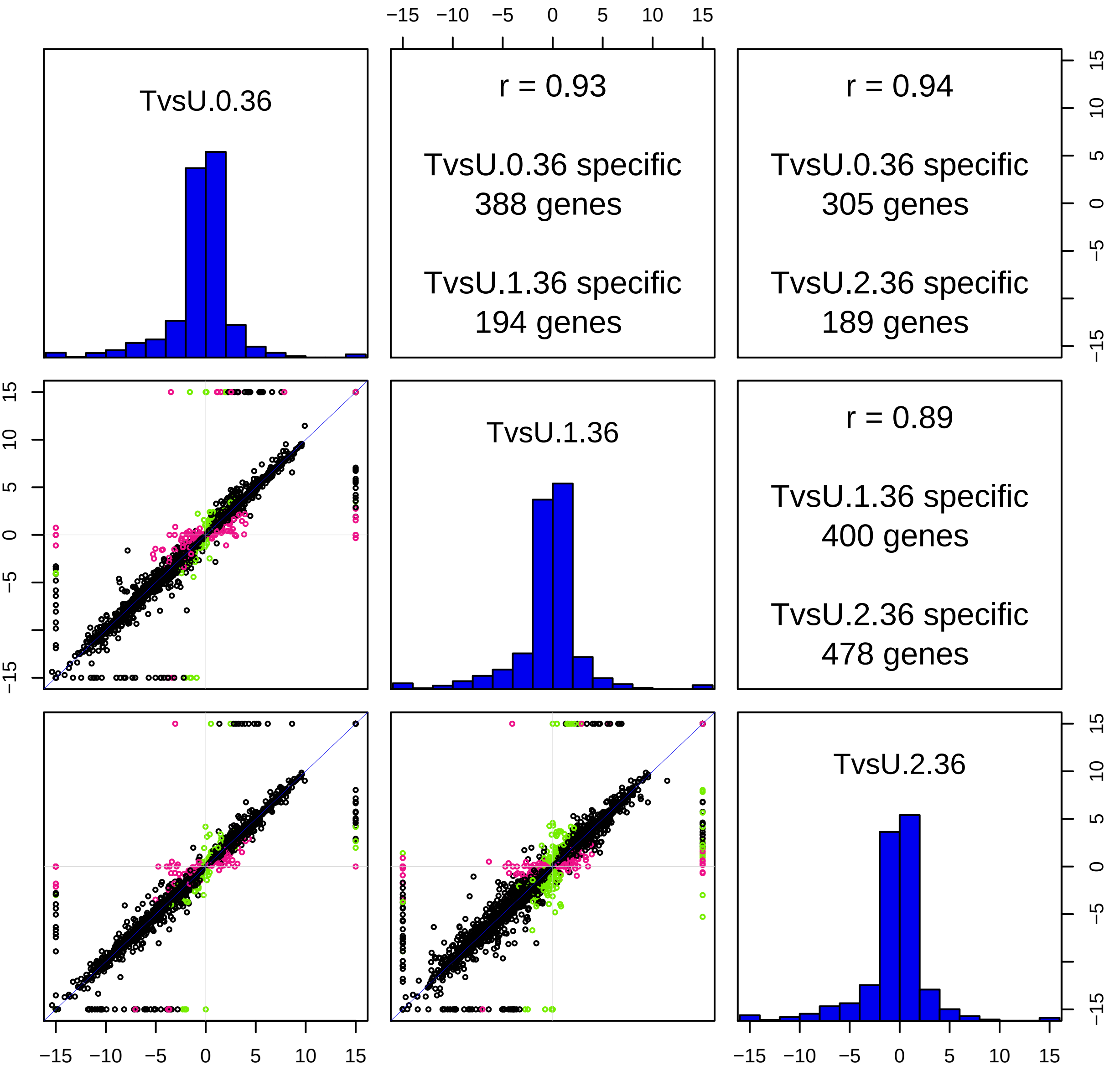
TvsU.0.36
r = 0.93
TvsU.0.36 specific
388 genes
TvsU.1.36 specific
194 genes
r = 0.94
TvsU.0.36 specific
305 genes
TvsU.2.36 specific
189 genes
TvsU.1.36
r = 0.89
TvsU.1.36 specific
400 genes
TvsU.2.36 specific
478 genes
TvsU.2.36
−15
−10
−5
0
5
10
15

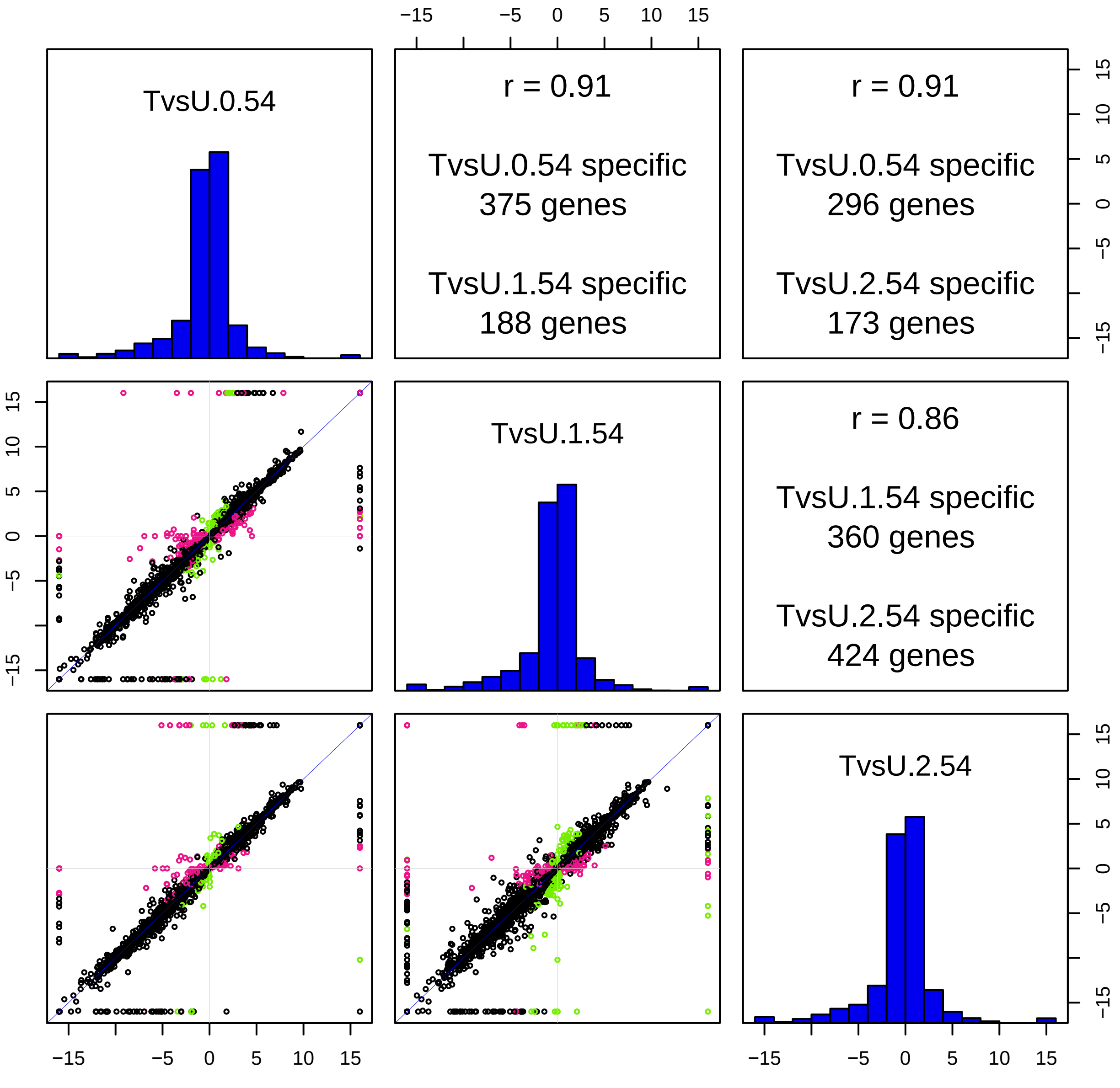

TvsU.0.54
r = 0.91
TvsU.0.54 specific
375 genes
TvsU.1.54 specific
188 genes
r = 0.91
TvsU.0.54 specific
296 genes
TvsU.2.54 specific
173 genes
TvsU.1.54
r = 0.86
TvsU.1.54 specific
360 genes
TvsU.2.54 specific
424 genes
TvsU.2.54
−15
−5
0
5
10
15

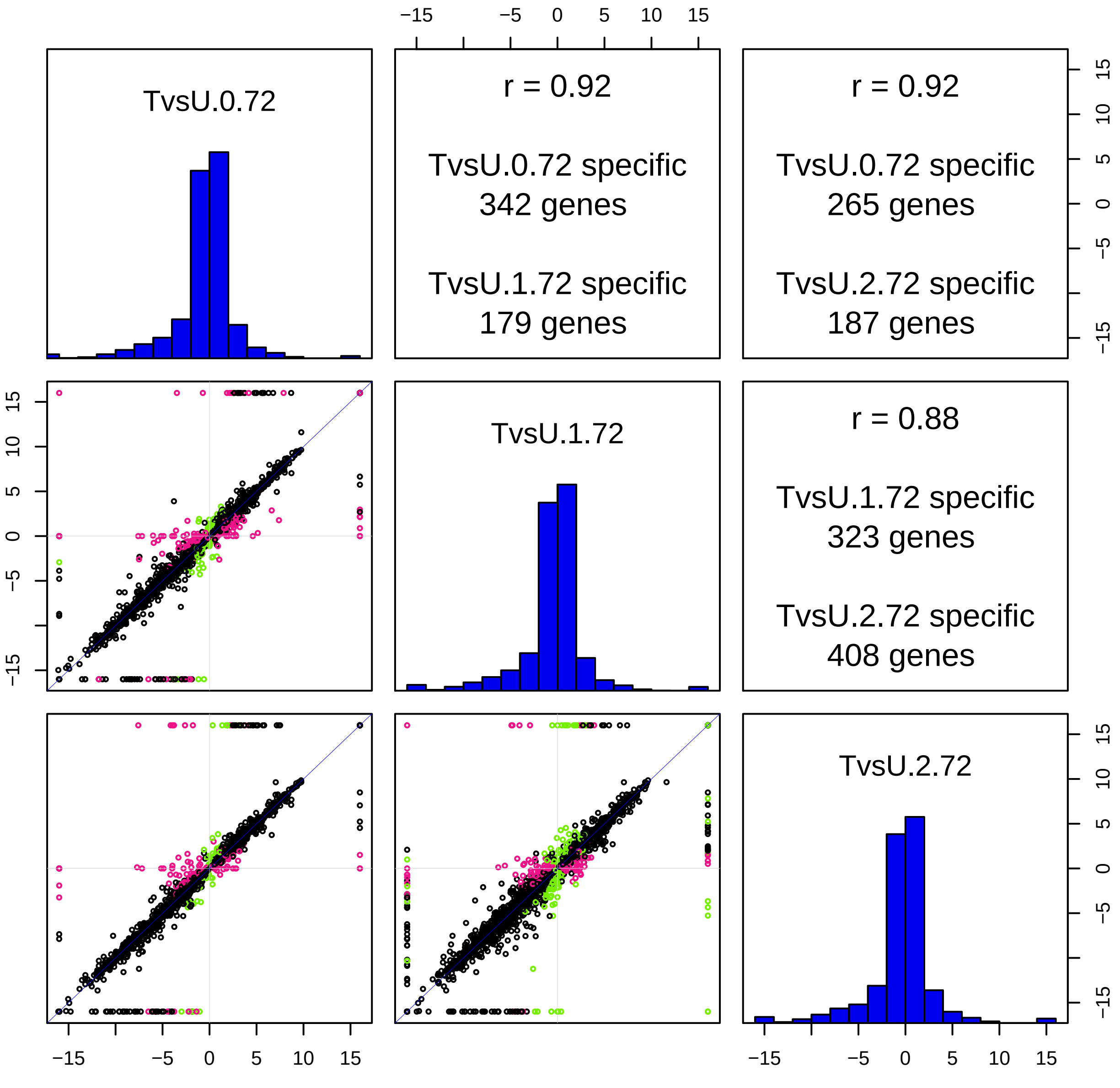

TvsU.0.72
r = 0.92
TvsU.0.72 specific
342 genes
TvsU.1.72 specific
179 genes
r = 0.92
TvsU.0.72 specific
265 genes
TvsU.2.72 specific
187 genes
TvsU.1.72
r = 0.88
TvsU.1.72 specific
323 genes
TvsU.2.72 specific
408 genes
TvsU.2.72
−15
−5
0
5
10
15

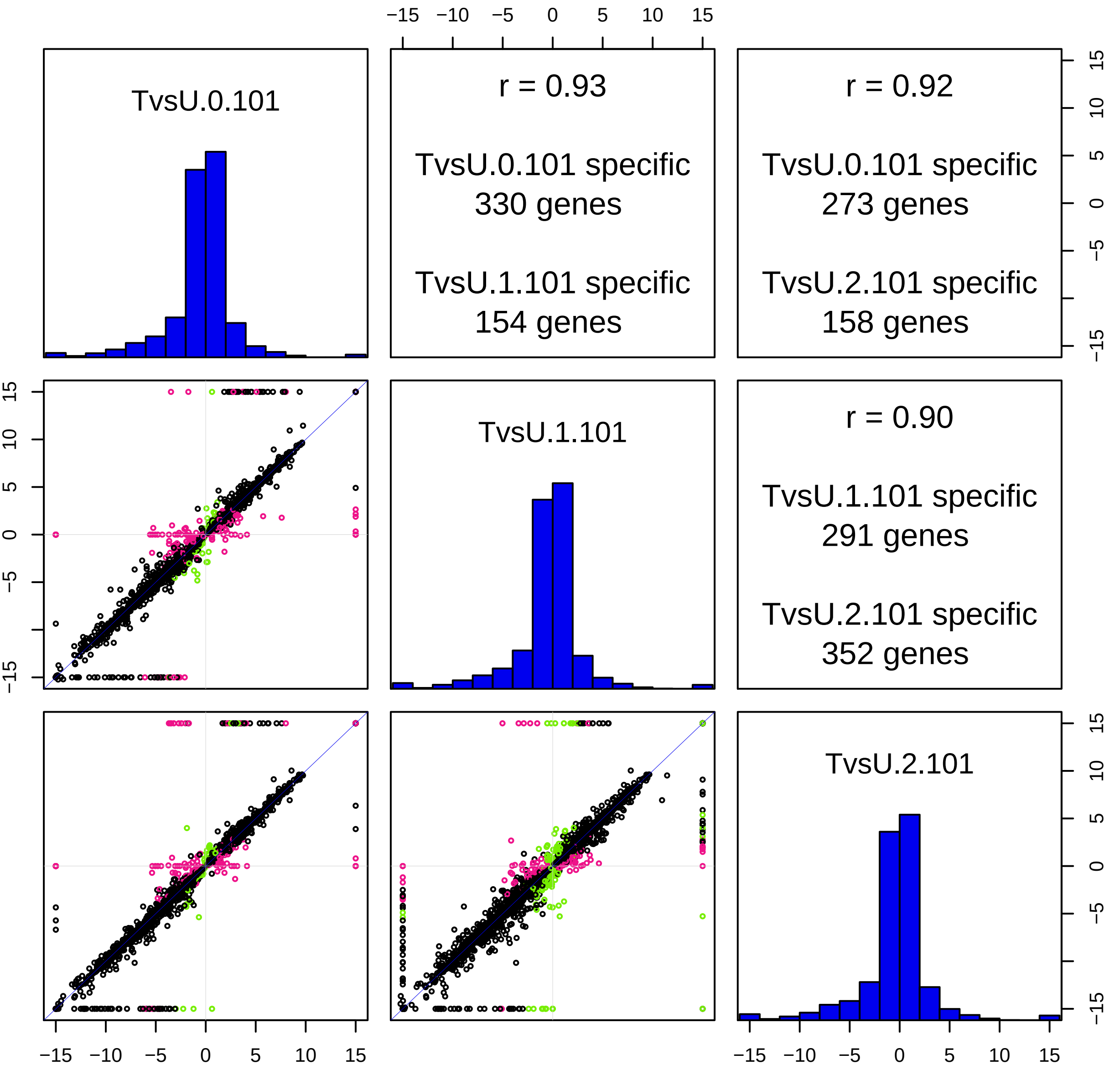
TvsU.0.101
r = 0.93
TvsU.0.101 specific
330 genes
TvsU.1.101 specific
154 genes
r = 0.92
TvsU.0.101 specific
273 genes
TvsU.2.101 specific
158 genes
TvsU.1.101
r = 0.90
TvsU.1.101 specific
291 genes
TvsU.2.101 specific
352 genes
TvsU.2.101
−15 −10 −5 0 5 10 15

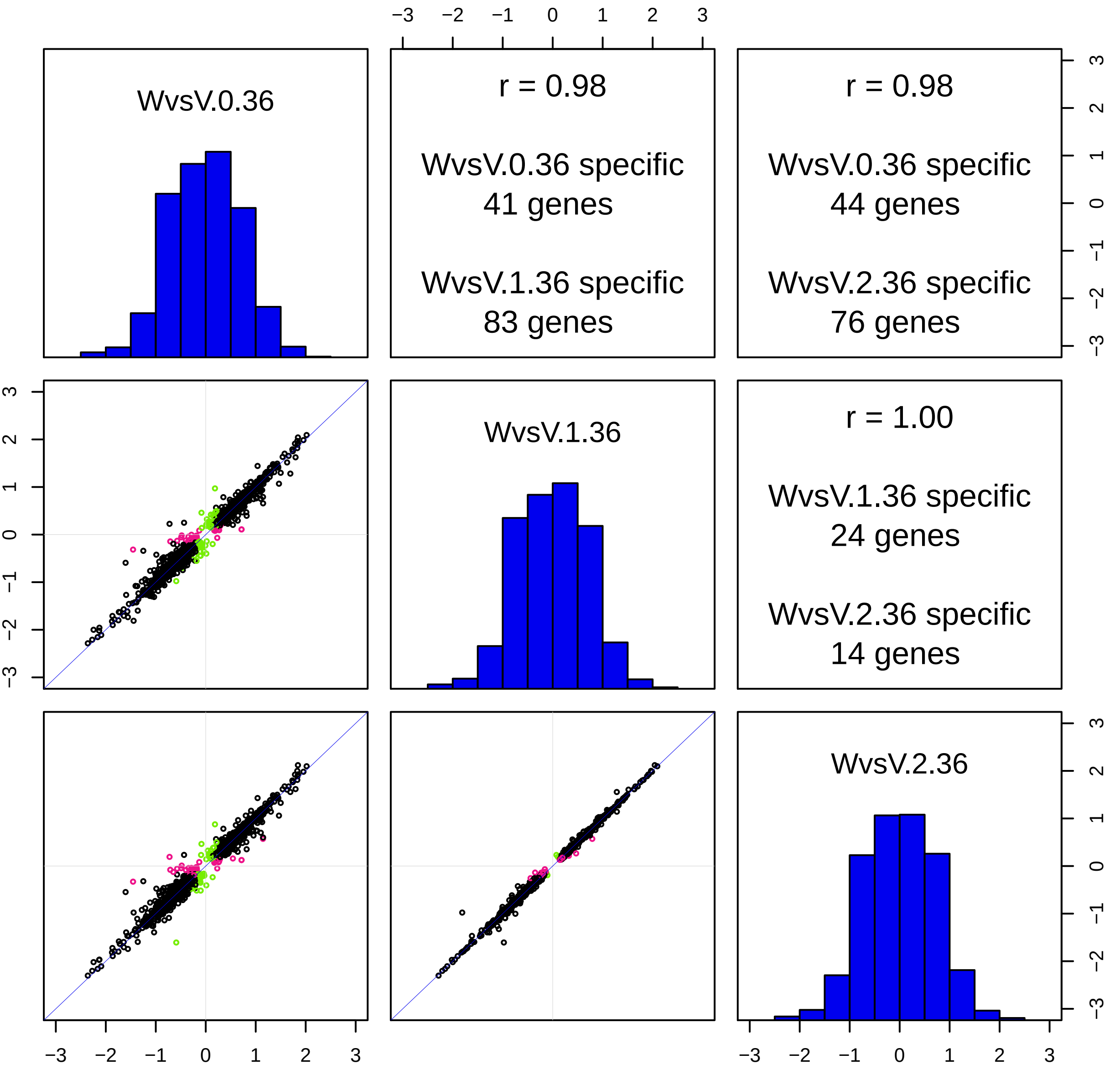
WvsV.0.36
r = 0.98
WvsV.0.36 specific
41 genes
WvsV.1.36 specific
83 genes
r = 0.98
WvsV.0.36 specific
44 genes
WvsV.2.36 specific
76 genes
WvsV.1.36
r = 1.00
WvsV.1.36 specific
24 genes
WvsV.2.36 specific
14 genes
WvsV.2.36
−3
−2
−1
0
1
2
3

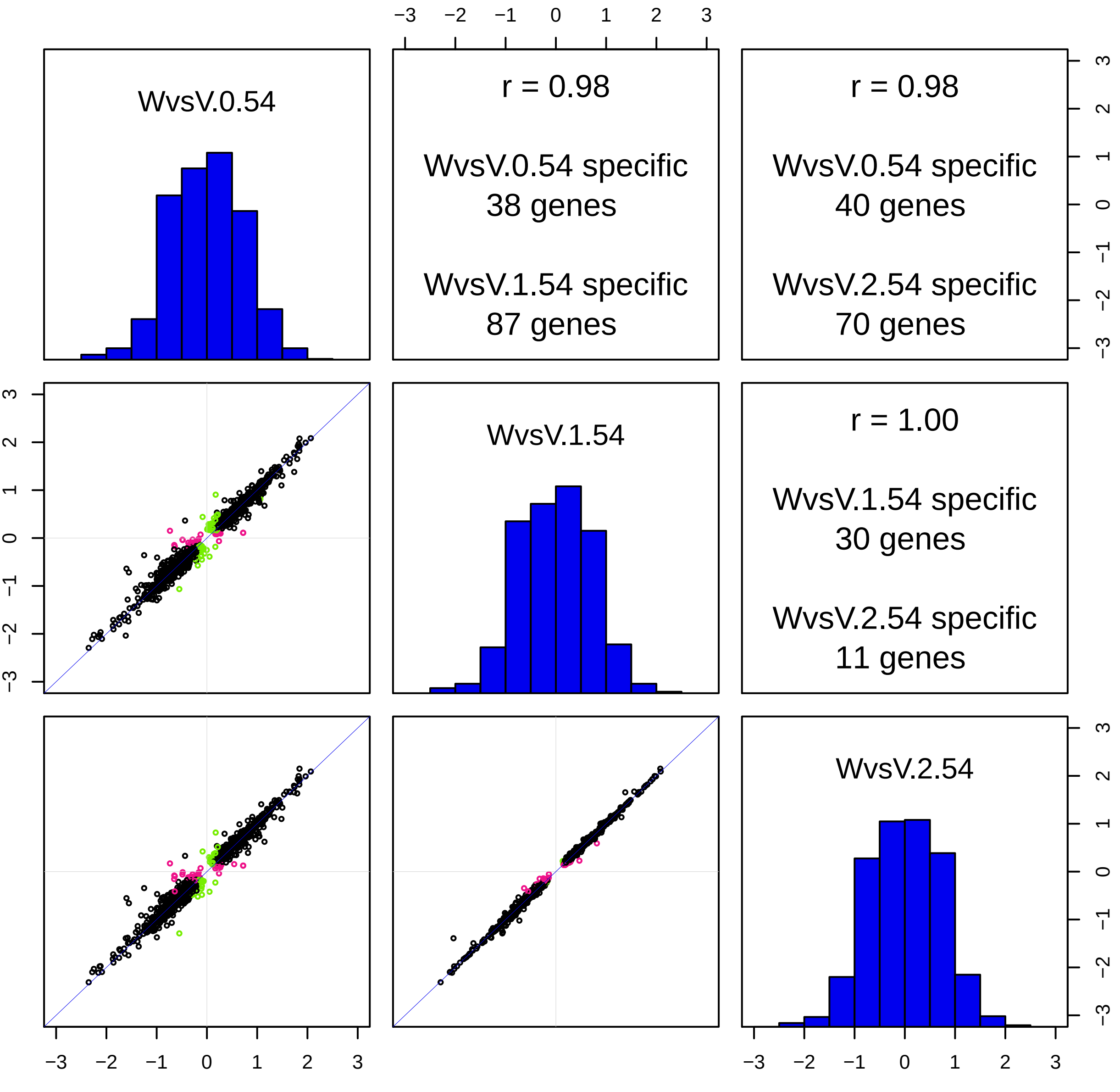

WvsV.0.54
r = 0.98
WvsV.0.54 specific
38 genes
WvsV.1.54 specific
87 genes
r = 0.98
WvsV.0.54 specific
40 genes
WvsV.2.54 specific
70 genes
WvsV.1.54
r = 1.00
WvsV.1.54 specific
30 genes
WvsV.2.54 specific
11 genes
WvsV.2.54
−3 −2 −1 0 1 2 3

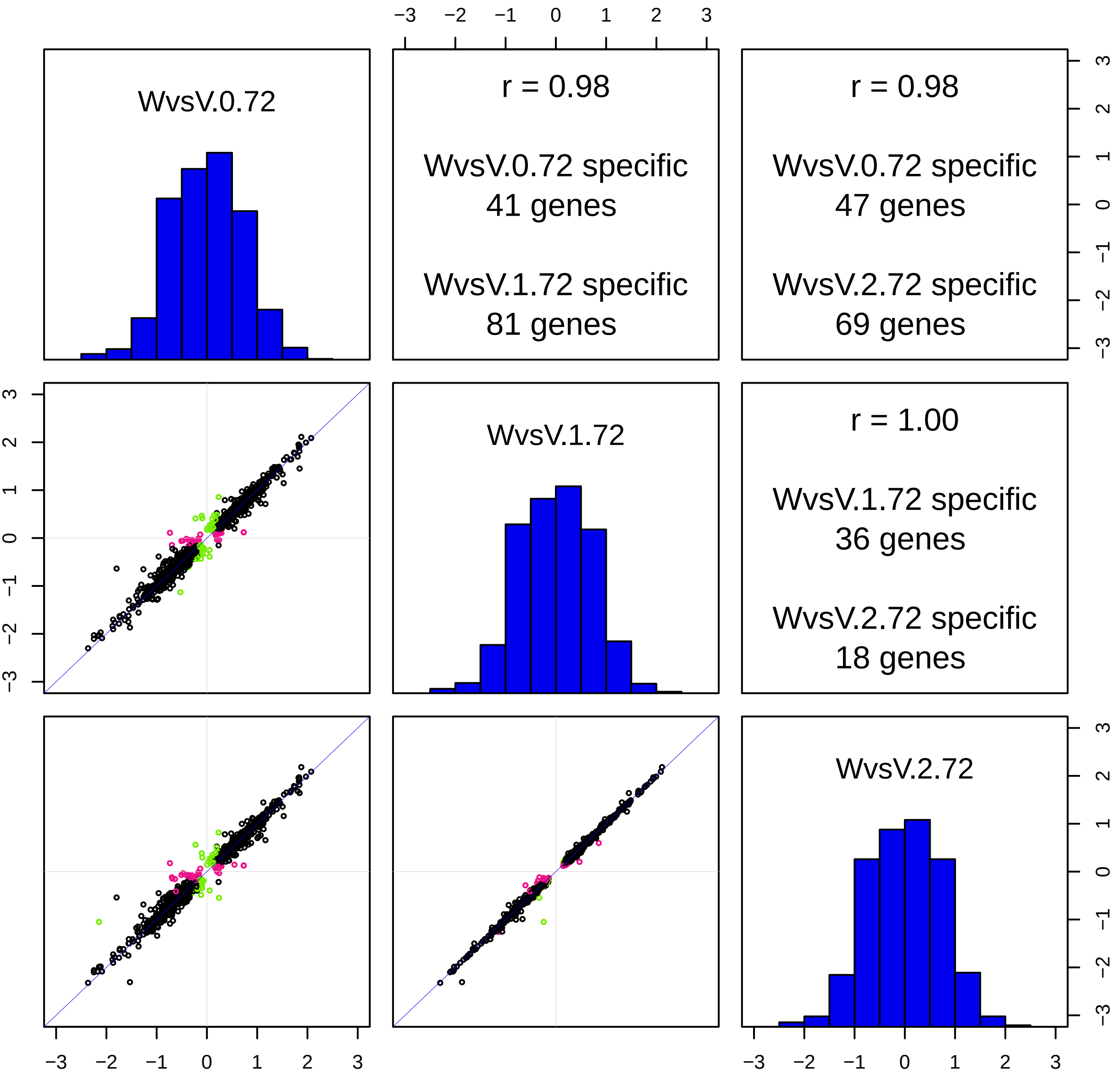
WvsV.0.72
r = 0.98
WvsV.0.72 specific
41 genes
WvsV.1.72 specific
81 genes
r = 0.98
WvsV.0.72 specific
47 genes
WvsV.2.72 specific
69 genes
WvsV.1.72
r = 1.00
WvsV.1.72 specific
36 genes
WvsV.2.72 specific
18 genes
WvsV.2.72
−3
−2
−1
0
1
2
3

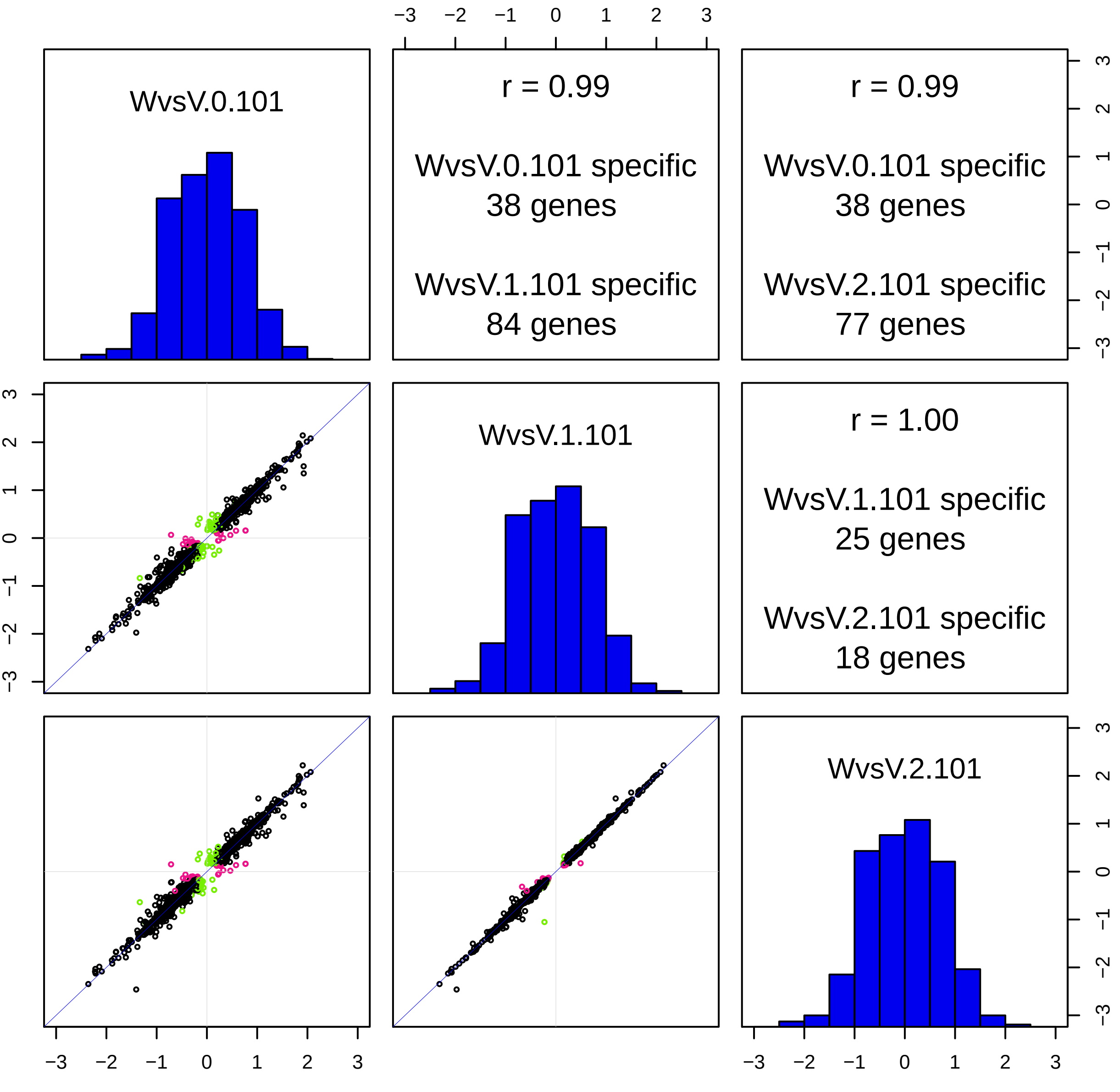
WvsV.0.101
r = 0.99
WvsV.0.101 specific
38 genes
WvsV.1.101 specific
84 genes
r = 0.99
WvsV.0.101 specific
38 genes
WvsV.2.101 specific
77 genes
WvsV.1.101
r = 1.00
WvsV.1.101 specific
25 genes
WvsV.2.101 specific
18 genes
WvsV.2.101
−3 −2 −1 0 1 2 3

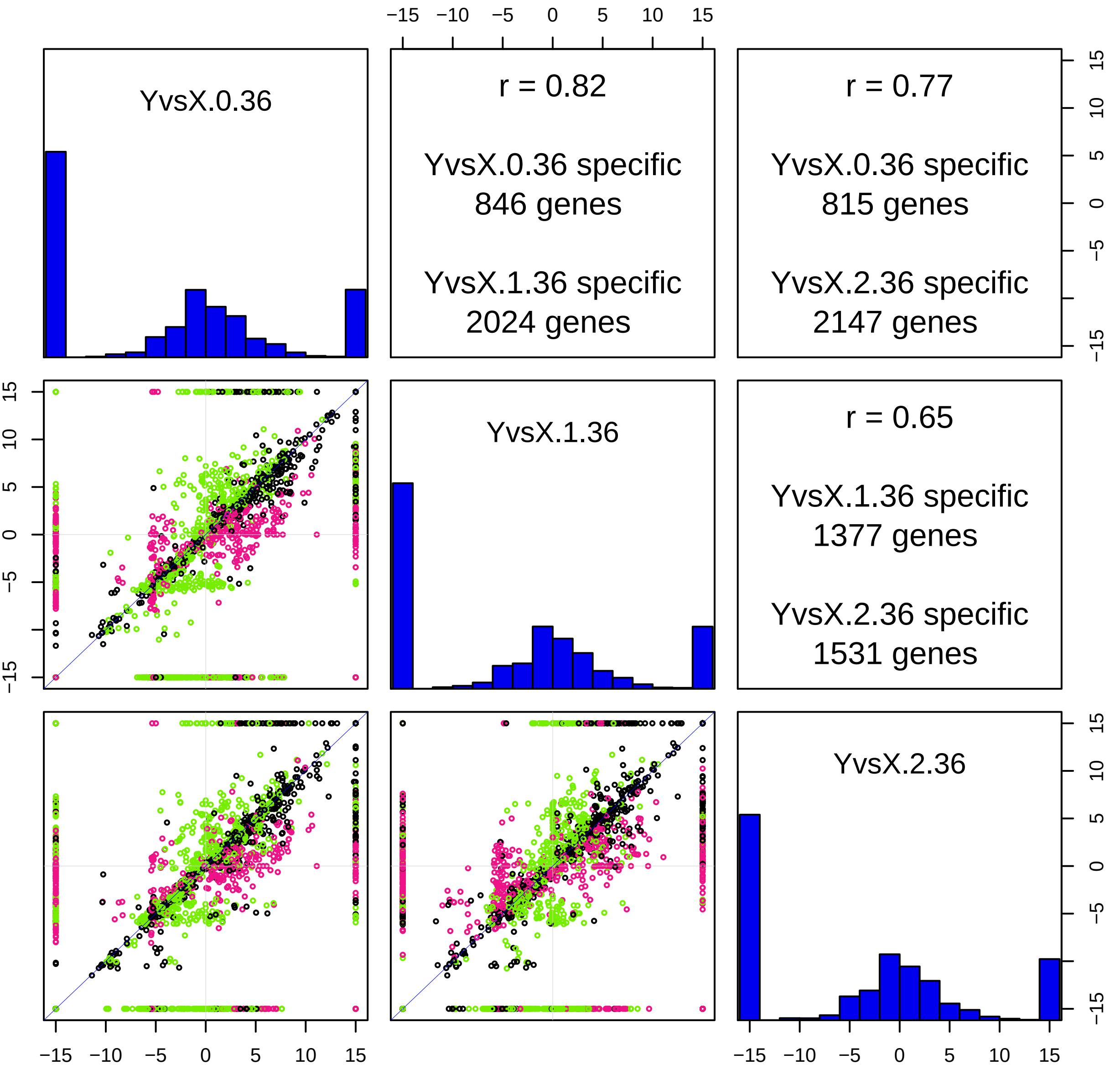

YvsX.0.36
r = 0.82
YvsX.0.36 specific
846 genes
YvsX.1.36 specific
2024 genes
r = 0.77
YvsX.0.36 specific
815 genes
YvsX.2.36 specific
2147 genes
YvsX.1.36
r = 0.65
YvsX.1.36 specific
1377 genes
YvsX.2.36 specific
1531 genes
YvsX.2.36
−15 −10 −5 0 5 10 15

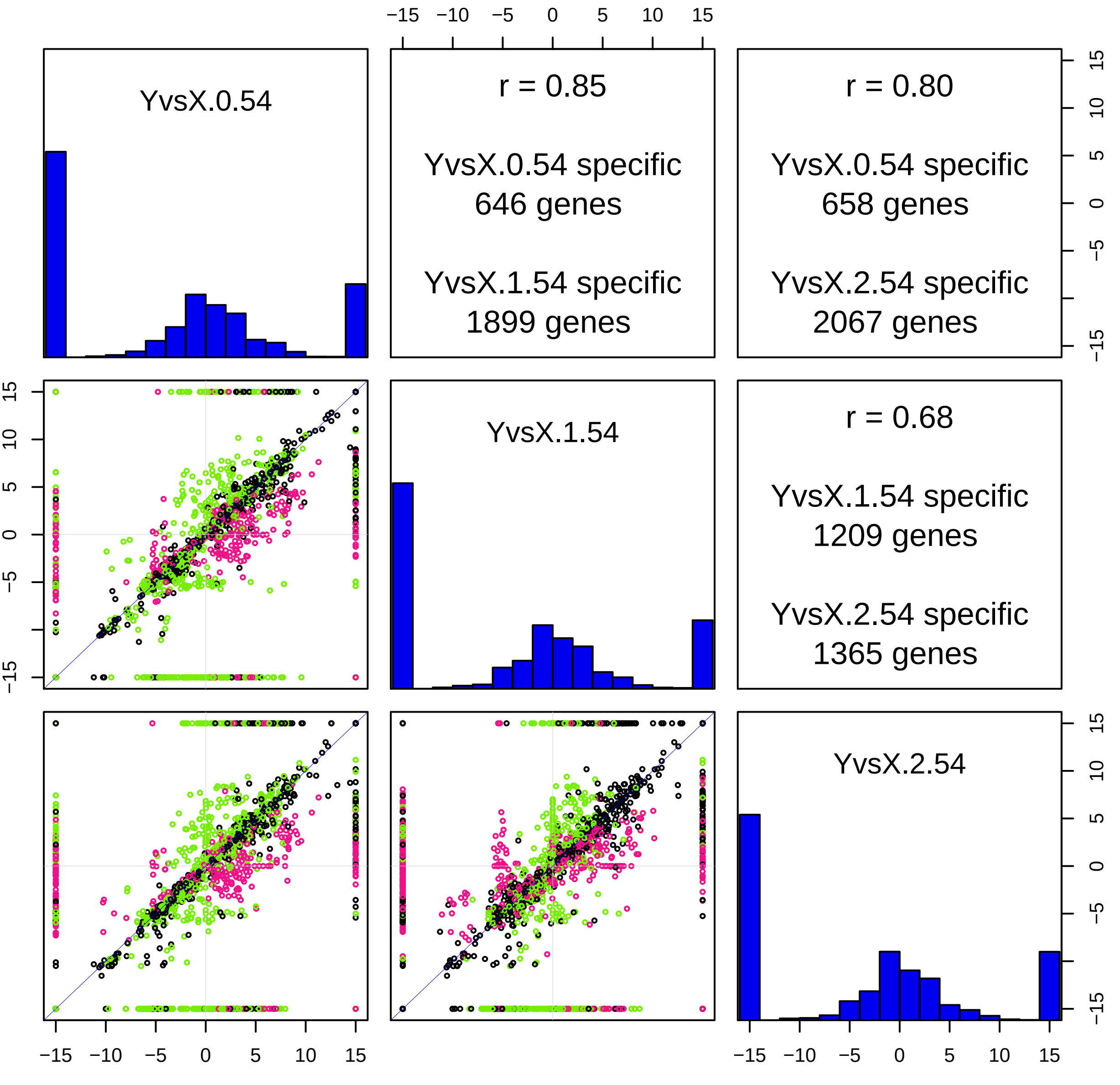

YvsX.0.54
r = 0.85
YvsX.0.54 specific
646 genes
YvsX.1.54 specific
1899 genes
r = 0.80
YvsX.0.54 specific
658 genes
YvsX.2.54 specific
2067 genes
YvsX.1.54
r = 0.68
YvsX.1.54 specific
1209 genes
YvsX.2.54 specific
1365 genes
YvsX.2.54
−15 −10 −5 0 5 10 15

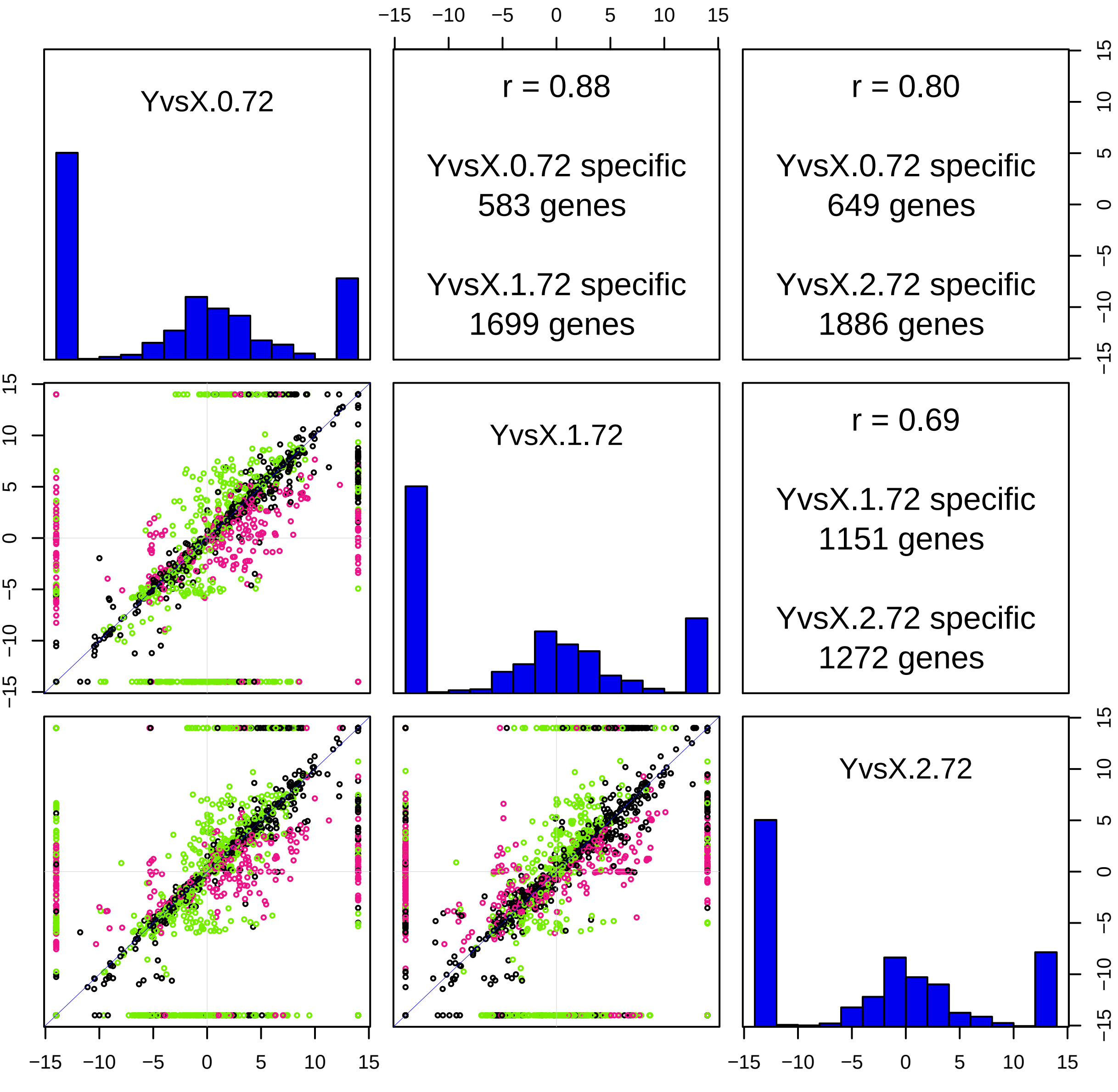

YvsX.0.72
r = 0.88
YvsX.0.72 specific
583 genes
YvsX.1.72 specific
1699 genes
r = 0.80
YvsX.0.72 specific
649 genes
YvsX.2.72 specific
1886 genes
YvsX.1.72
r = 0.69
YvsX.1.72 specific
1151 genes
YvsX.2.72 specific
1272 genes
YvsX.2.72
−15
−10
−5
0
5
10
15

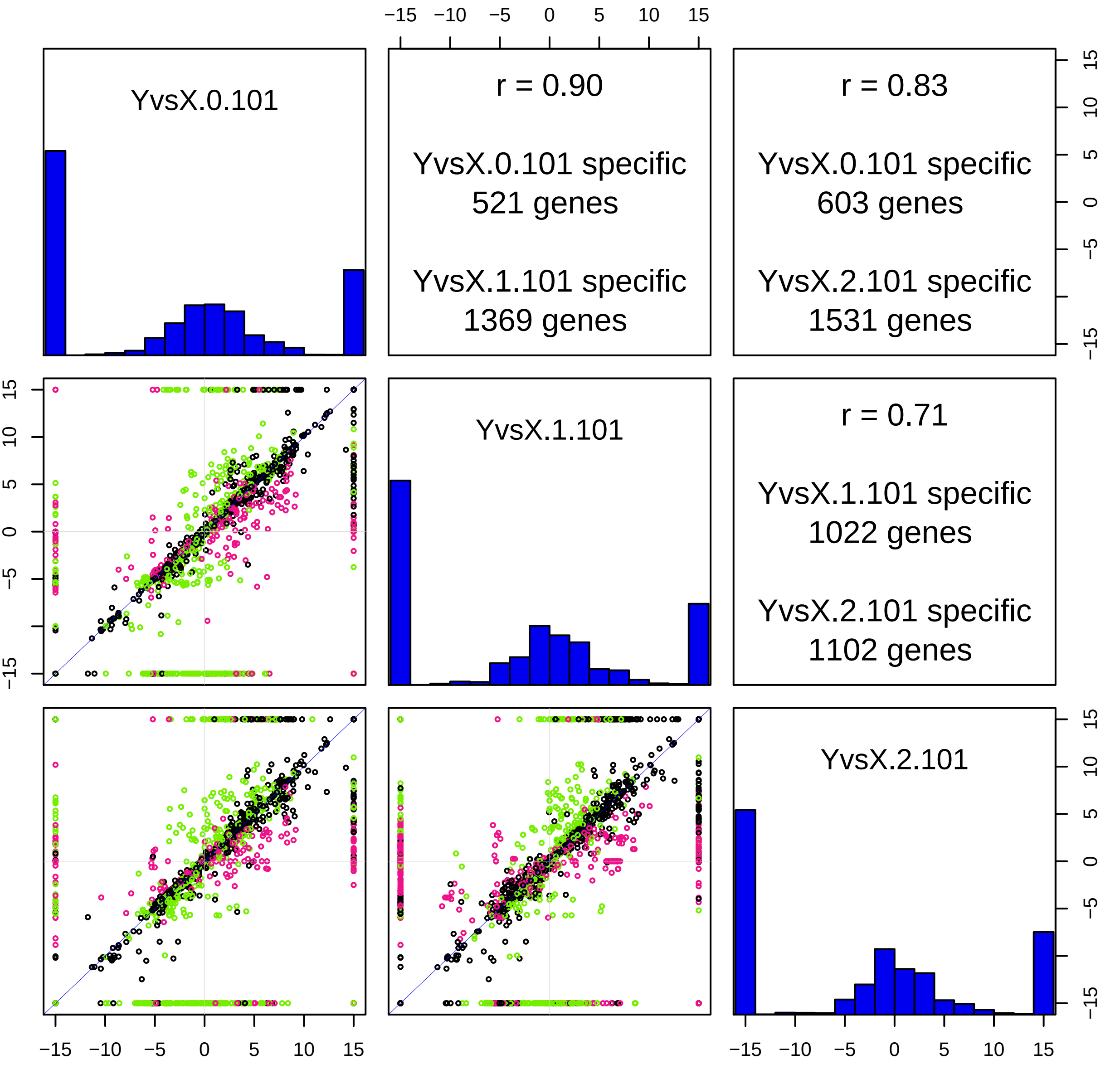
YvsX.0.101
r = 0.90
YvsX.0.101 specific
521 genes
YvsX.1.101 specific
1369 genes
r = 0.83
YvsX.0.101 specific
603 genes
YvsX.2.101 specific
1531 genes
YvsX.1.101
r = 0.71
YvsX.1.101 specific
1022 genes
YvsX.2.101 specific
1102 genes
YvsX.2.101
−15 −10 −5 0 5 10 15

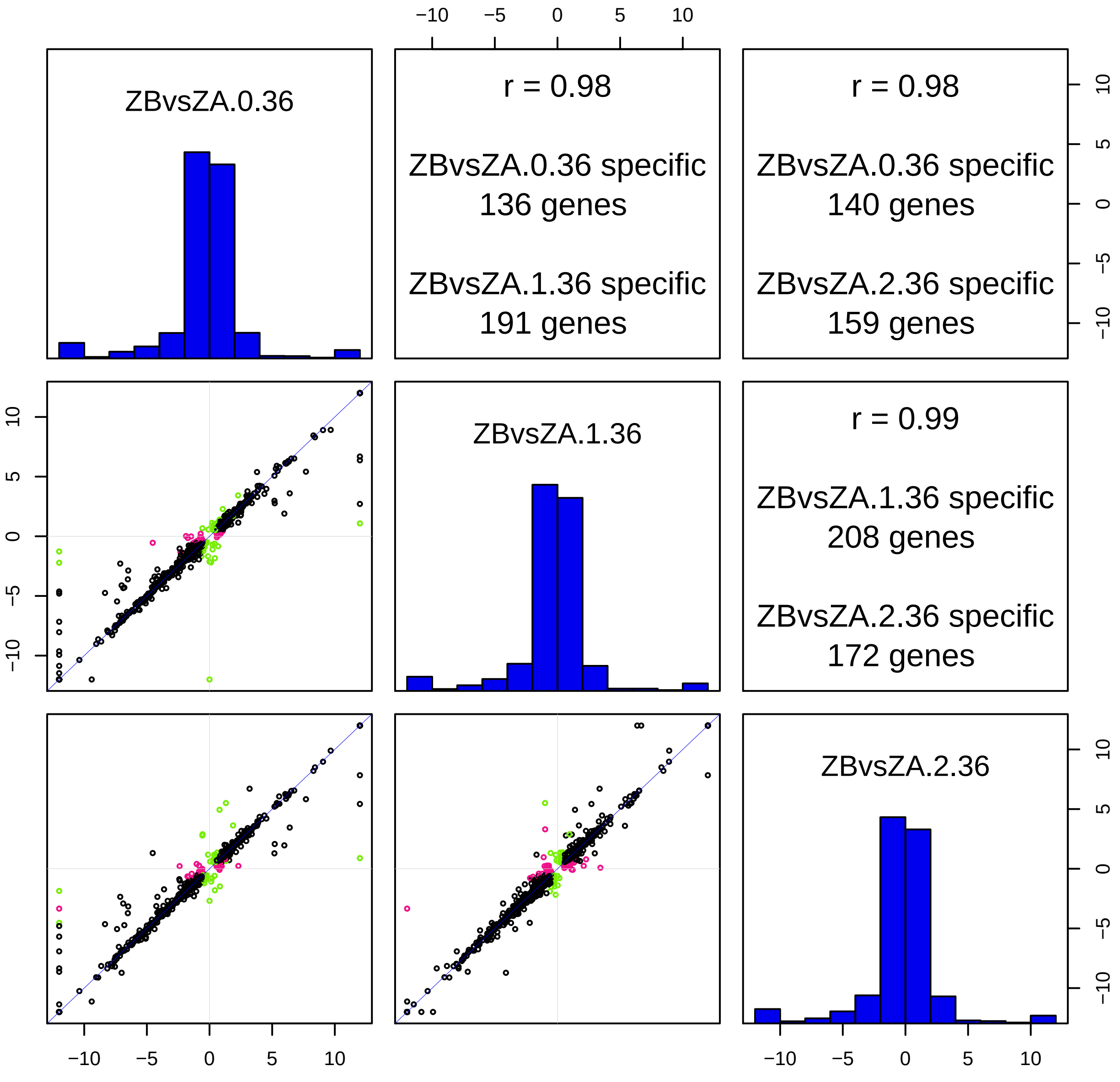

ZBvsZA.0.36
r = 0.98
ZBvsZA.0.36 specific
136 genes
ZBvsZA.1.36 specific
191 genes
r = 0.98
ZBvsZA.0.36 specific
140 genes
ZBvsZA.2.36 specific
159 genes
ZBvsZA.1.36
r = 0.99
ZBvsZA.1.36 specific
208 genes
ZBvsZA.2.36 specific
172 genes
ZBvsZA.2.36
−10
−5
0
5
10

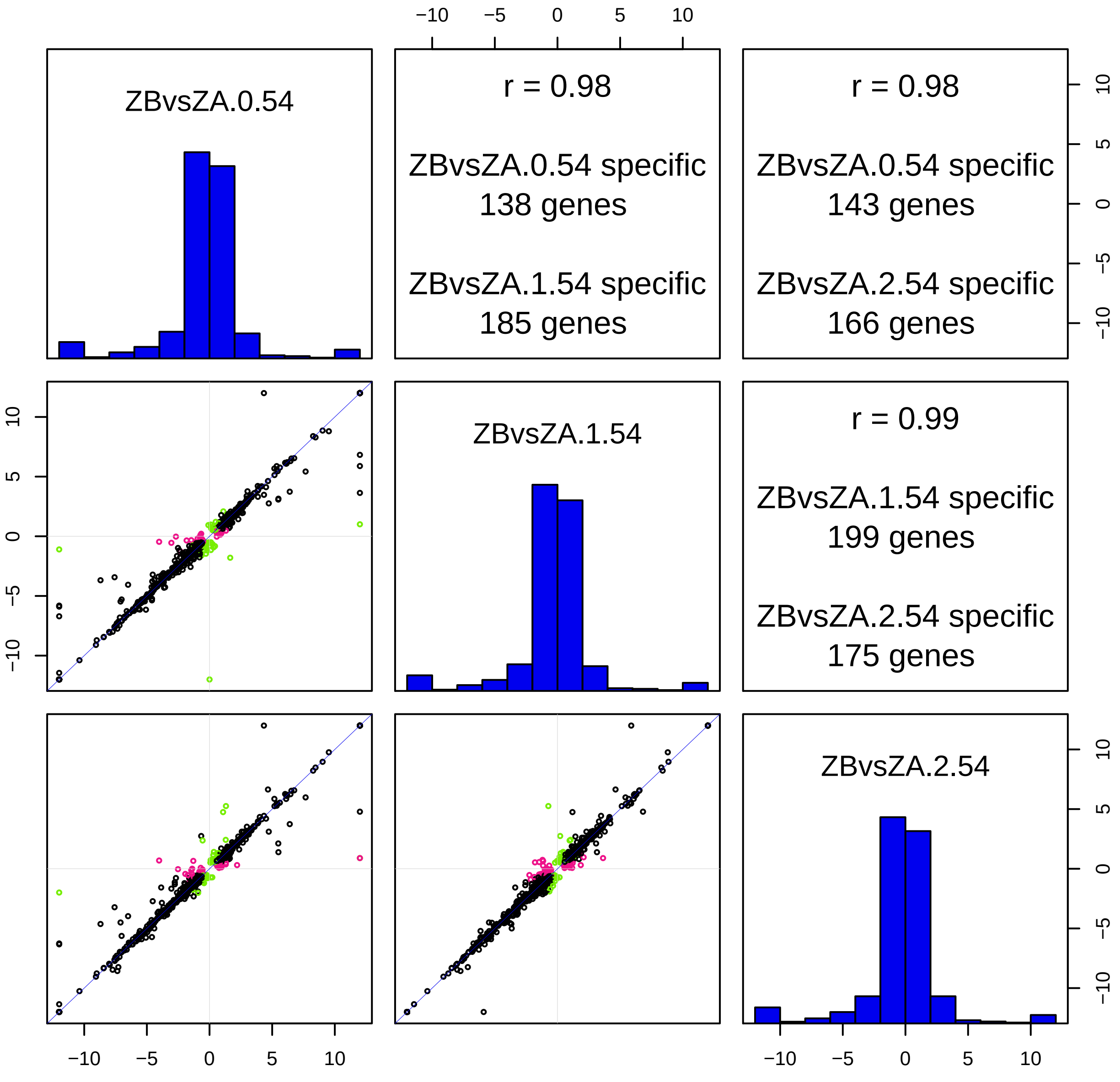

ZBvsZA.0.54
r = 0.98
ZBvsZA.0.54 specific
138 genes
ZBvsZA.1.54 specific
185 genes
r = 0.98
ZBvsZA.0.54 specific
143 genes
ZBvsZA.2.54 specific
166 genes
ZBvsZA.1.54
r = 0.99
ZBvsZA.1.54 specific
199 genes
ZBvsZA.2.54 specific
175 genes
ZBvsZA.2.54
−10
−5
0
5
10

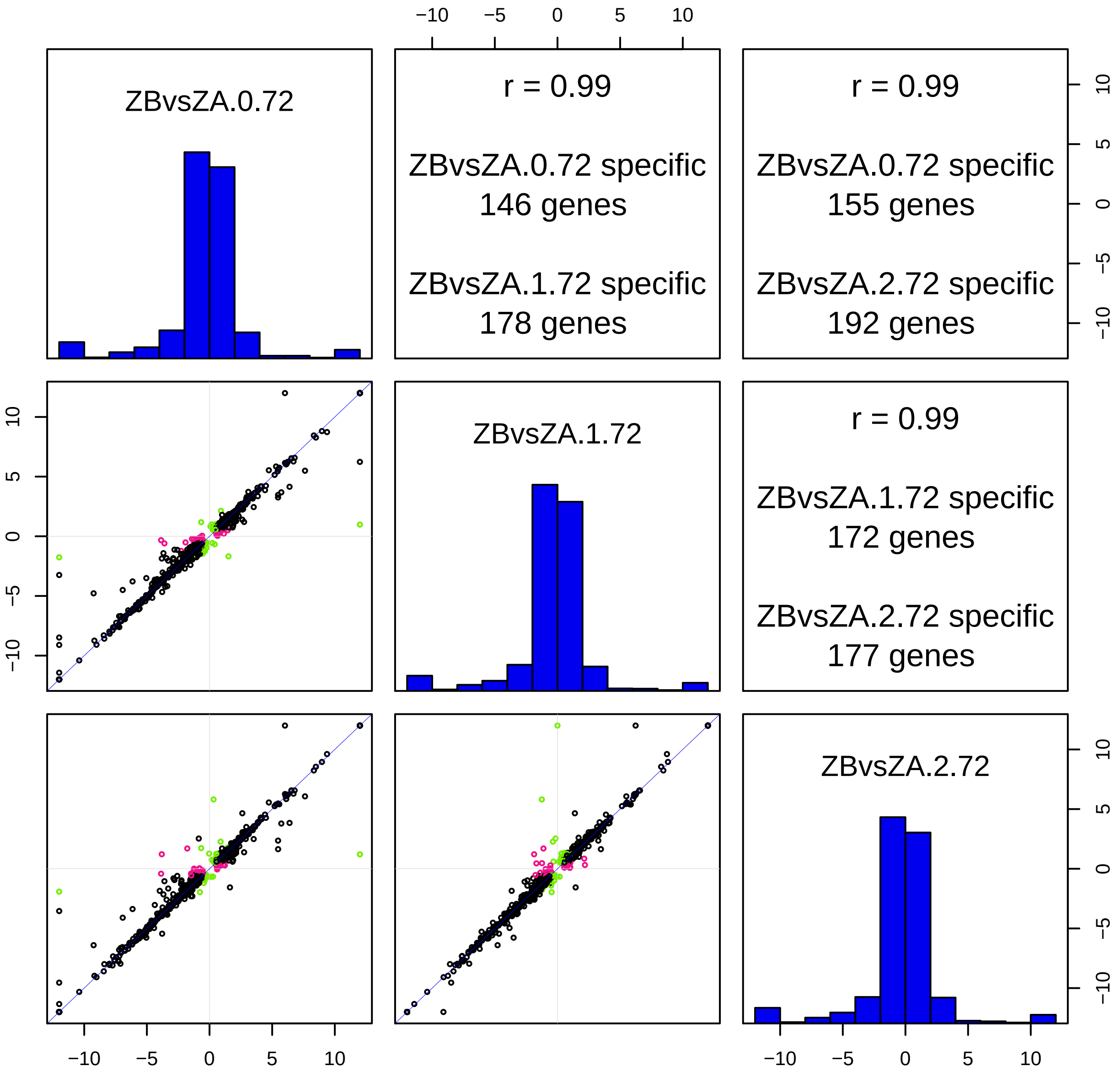
ZBvsZA.0.72
r = 0.99
ZBvsZA.0.72 specific
146 genes
ZBvsZA.1.72 specific
178 genes
r = 0.99
ZBvsZA.0.72 specific
155 genes
ZBvsZA.2.72 specific
192 genes
ZBvsZA.1.72
r = 0.99
ZBvsZA.1.72 specific
172 genes
ZBvsZA.2.72 specific
177 genes
ZBvsZA.2.72
−10
−5
0
5
10

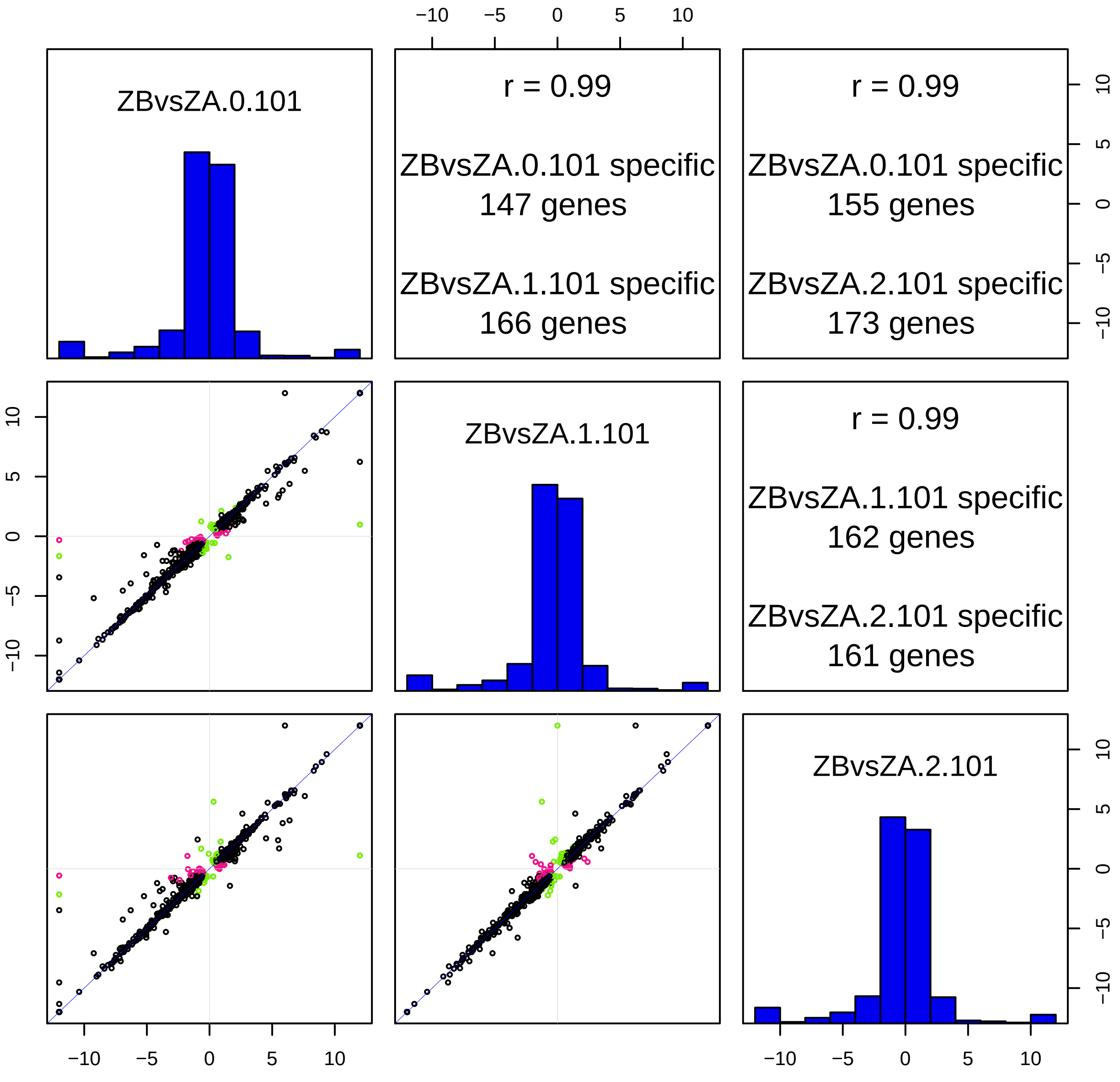

ZBvsZA.0.101
r = 0.99
ZBvsZA.0.101 specific
147 genes
ZBvsZA.1.101 specific
166 genes
r = 0.99
ZBvsZA.0.101 specific
155 genes
ZBvsZA.2.101 specific
173 genes
ZBvsZA.1.101
r = 0.99
ZBvsZA.1.101 specific
162 genes
ZBvsZA.2.101 specific
161 genes
ZBvsZA.2.101
−10
−5
0
5
10

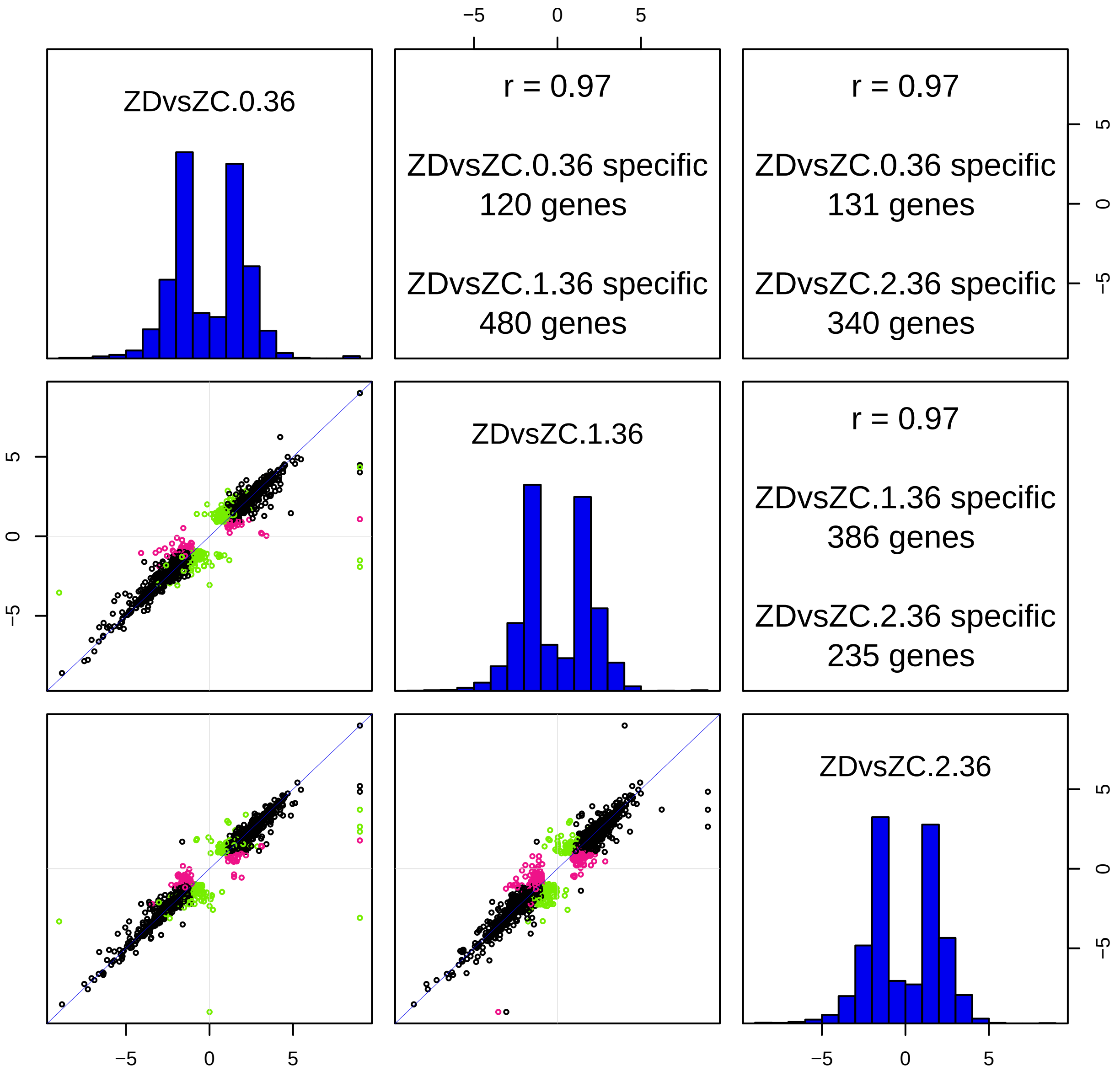
ZDvsZC.0.36
r = 0.97
ZDvsZC.0.36 specific
120 genes
ZDvsZC.1.36 specific
480 genes
r = 0.97
ZDvsZC.0.36 specific
131 genes
ZDvsZC.2.36 specific
340 genes
ZDvsZC.1.36
r = 0.97
ZDvsZC.1.36 specific
386 genes
ZDvsZC.2.36 specific
235 genes
ZDvsZC.2.36
−5
0
5

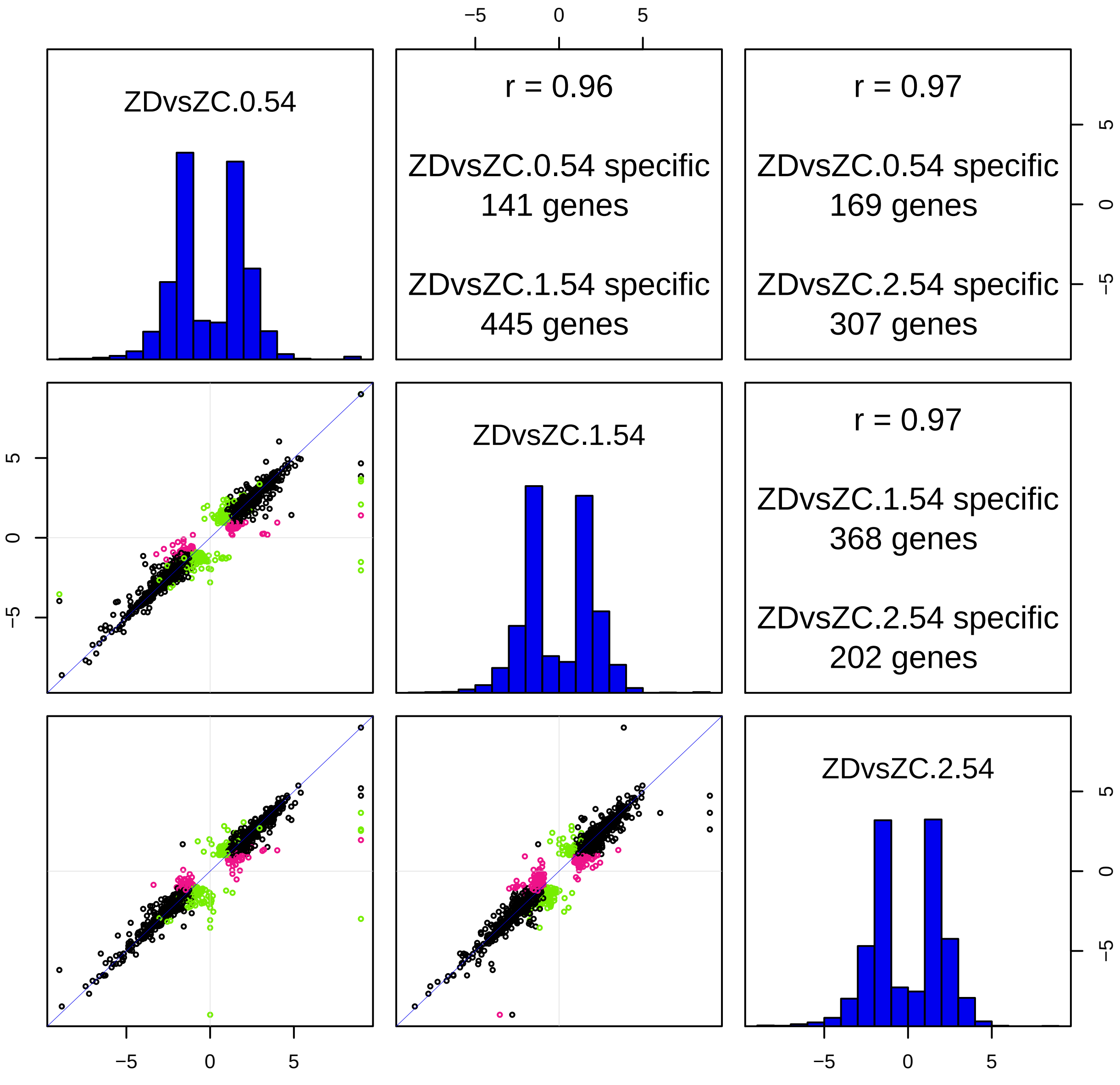
ZDvsZC.0.54
r = 0.96
ZDvsZC.0.54 specific
141 genes
ZDvsZC.1.54 specific
445 genes
r = 0.97
ZDvsZC.0.54 specific
169 genes
ZDvsZC.2.54 specific
307 genes
ZDvsZC.1.54
r = 0.97
ZDvsZC.1.54 specific
368 genes
ZDvsZC.2.54 specific
202 genes
ZDvsZC.2.54
−5
0
5

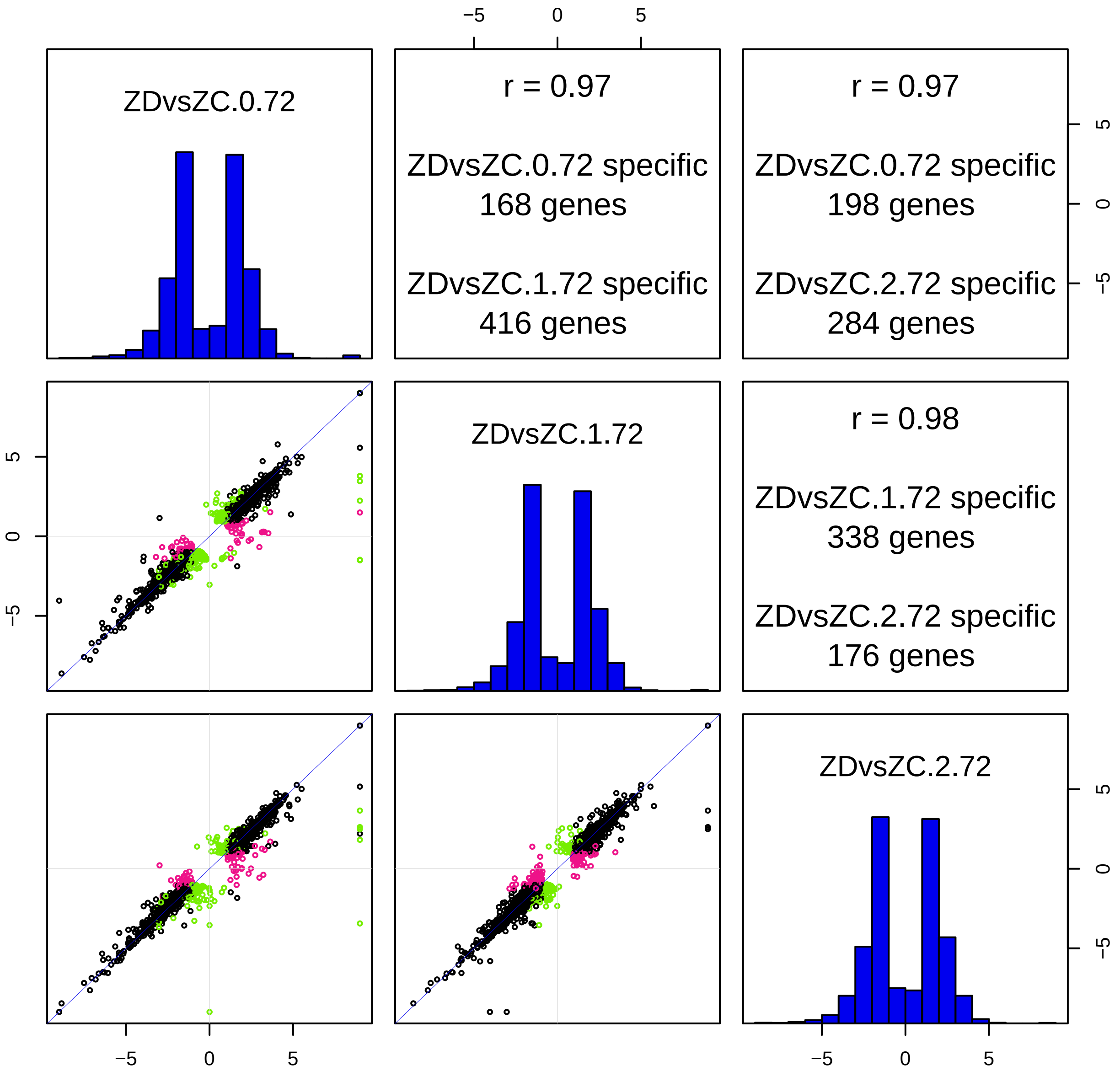
ZDvsZC.0.72
r = 0.97
ZDvsZC.0.72 specific
168 genes
ZDvsZC.1.72 specific
416 genes
r = 0.97
ZDvsZC.0.72 specific
198 genes
ZDvsZC.2.72 specific
284 genes
ZDvsZC.1.72
r = 0.98
ZDvsZC.1.72 specific
338 genes
ZDvsZC.2.72 specific
176 genes
ZDvsZC.2.72
−5
0
5

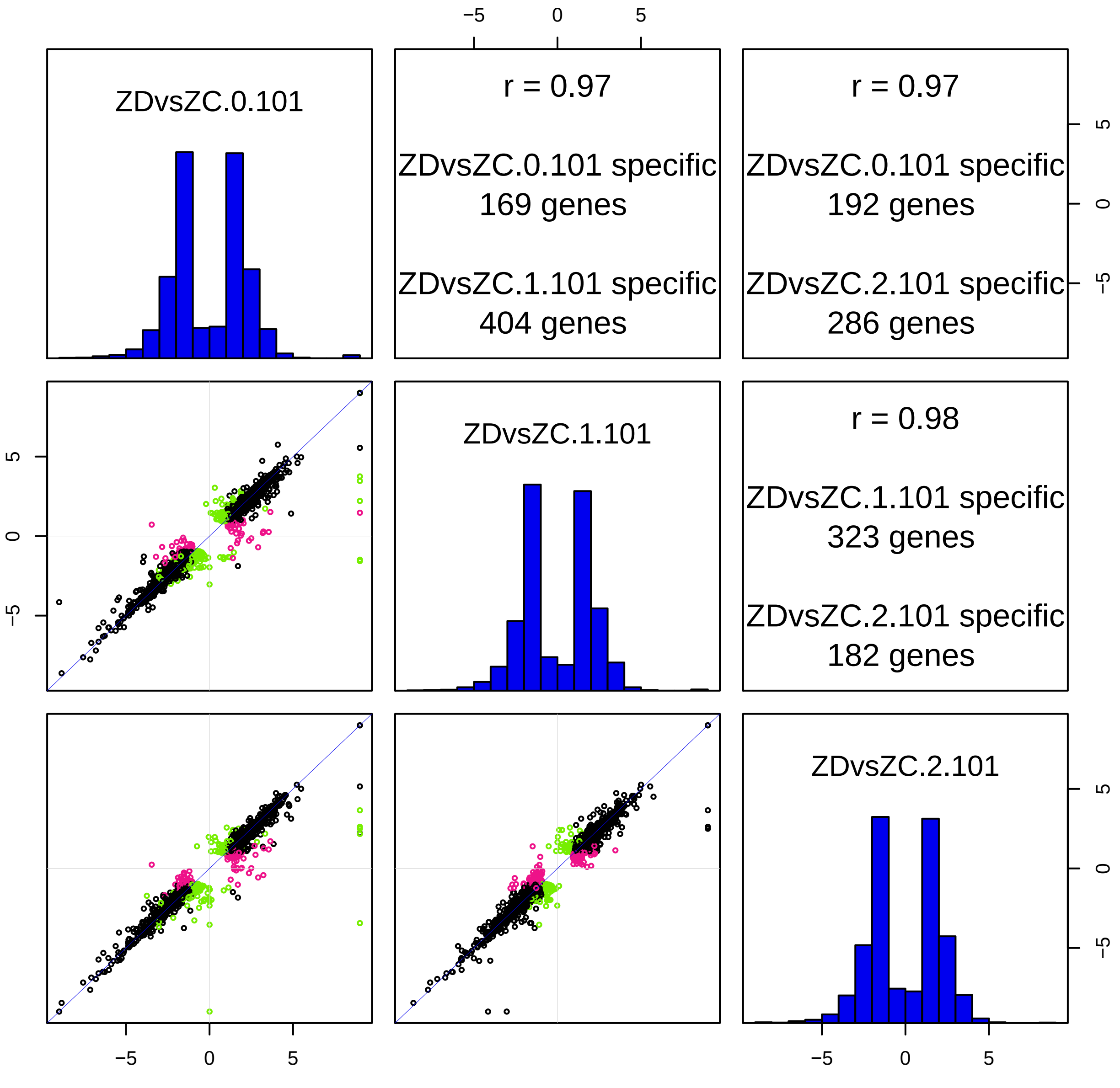

ZDvsZC.0.101
r = 0.97
ZDvsZC.0.101 specific
169 genes
ZDvsZC.1.101 specific
404 genes
r = 0.97
ZDvsZC.0.101 specific
192 genes
ZDvsZC.2.101 specific
286 genes
ZDvsZC.1.101
r = 0.98
ZDvsZC.1.101 specific
323 genes
ZDvsZC.2.101 specific
182 genes
ZDvsZC.2.101
−5
0
5

Additional File 31. Compendium of figures for *Es. coli* data with results separated by read pairing status using the BWA MEM aligner. A heatmap with hierarchical clustering with statistical support is shown on page 1 with the condition denoted according to letter code from Supplementary Table 2, followed by the replicate designation and the read length. A PCA plot is shown on page 2 where the conditions are denoted by the shape (circle, DMEM; triangle, LB) and the read length by the color (green, 36 bp; blue, 54 bp; magenta, 72 bp; purple, 101 bp). On both pages, results are shown in the three panels for (A) paired end reads, (B) first-in-pair single end reads, and (C) second-in-pair single reads. To compare the results from BWA MEM to those with Bowtie, these figures would be compared to Additional File 6.

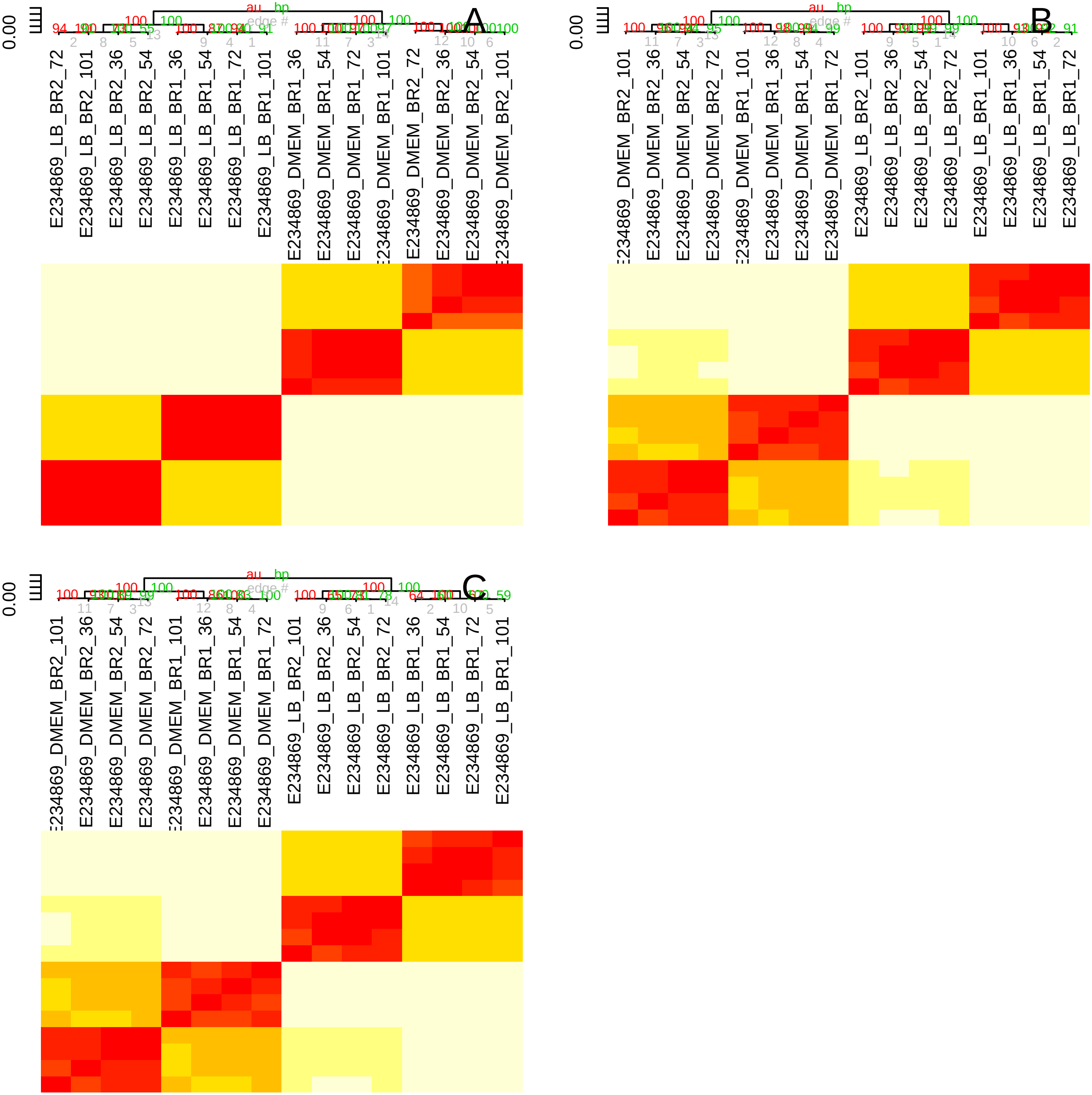

A
au
bp
edge #
0.00
E234869_LB_BR2_72
E234869_LB_BR2_101
E234869_LB_BR2_36
E234869_LB_BR2_54
E234869_LB_BR1_36
E234869_LB_BR1_54
E234869_LB_BR1_72
E234869_LB_BR1_101
E234869_DMEM_BR1_36
E234869_DMEM_BR1_54
E234869_DMEM_BR1_72
E234869_DMEM_BR1_101
E234869_DMEM_BR2_72
E234869_DMEM_BR2_36
E234869_DMEM_BR2_54
E234869_DMEM_BR2_101
B
au
bp
edge #
0.00
E234869_DMEM_BR2_101
E234869_DMEM_BR2_36
E234869_DMEM_BR2_54
E234869_DMEM_BR2_72
E234869_DMEM_BR1_101
E234869_DMEM_BR1_36
E234869_DMEM_BR1_54
E234869_DMEM_BR1_72
E234869_LB_BR2_101
E234869_LB_BR2_36
E234869_LB_BR2_54
E234869_LB_BR2_72
E234869_LB_BR1_101
E234869_LB_BR1_36
E234869_LB_BR1_54
E234869_LB_BR1_72
C
au
bp
edge #
0.00
E234869_DMEM_BR2_101
E234869_DMEM_BR2_36
E234869_DMEM_BR2_54
E234869_DMEM_BR2_72
E234869_DMEM_BR1_101
E234869_DMEM_BR1_36
E234869_DMEM_BR1_54
E234869_DMEM_BR1_72
E234869_LB_BR2_101
E234869_LB_BR2_36
E234869_LB_BR2_54
E234869_LB_BR2_72
E234869_LB_BR1_36
E234869_LB_BR1_54
E234869_LB_BR1_72
E234869_LB_BR1_101

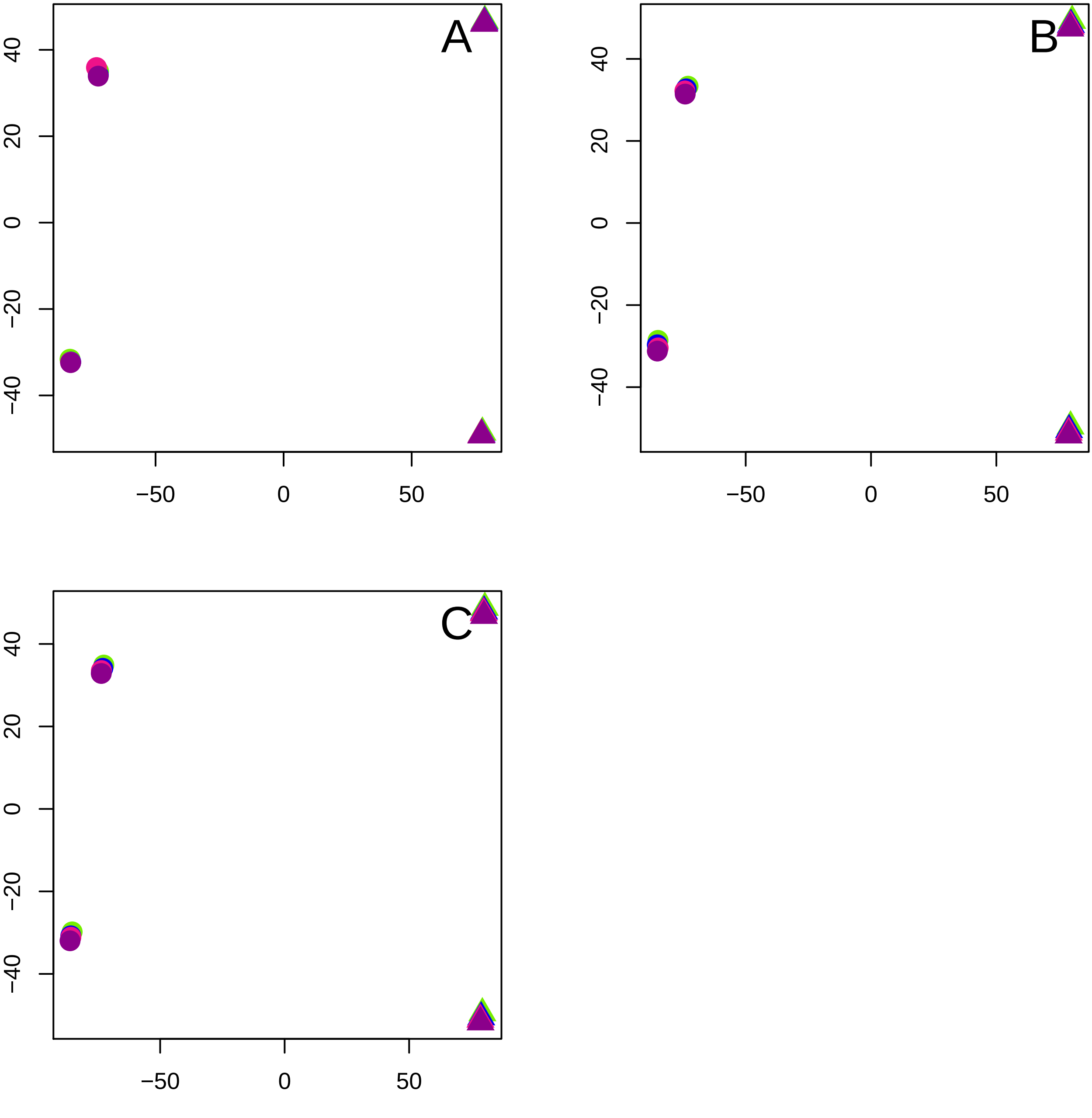

A
B
C
40
20
0
−20
−40
−50
0
50

Additional File 32. Compendium of figures for data from the *Wolbachia* endosymbiont *w*Bm in adult male and adult female *B. malayi* hosts with results separated by read pairing status using the BWA MEM aligner. A heatmap with hierarchical clustering with statistical support is shown on page 1 with the condition denoted according to letter code from Supplementary Table 2, followed by the replicate designation and the read length. A PCA plot is shown on page 2 where the conditions are denoted by the shape and the read length by the color (green, 36 bp; blue, 54 bp; magenta, 72 bp; purple, 101 bp). On both pages, results are shown in the three panels for (A) paired end reads, (B) first-in-pair single end reads, and (C) second-in-pair single reads. To compare the results from BWA MEM to those with Bowtie, these figures would be compared to Additional File 9.

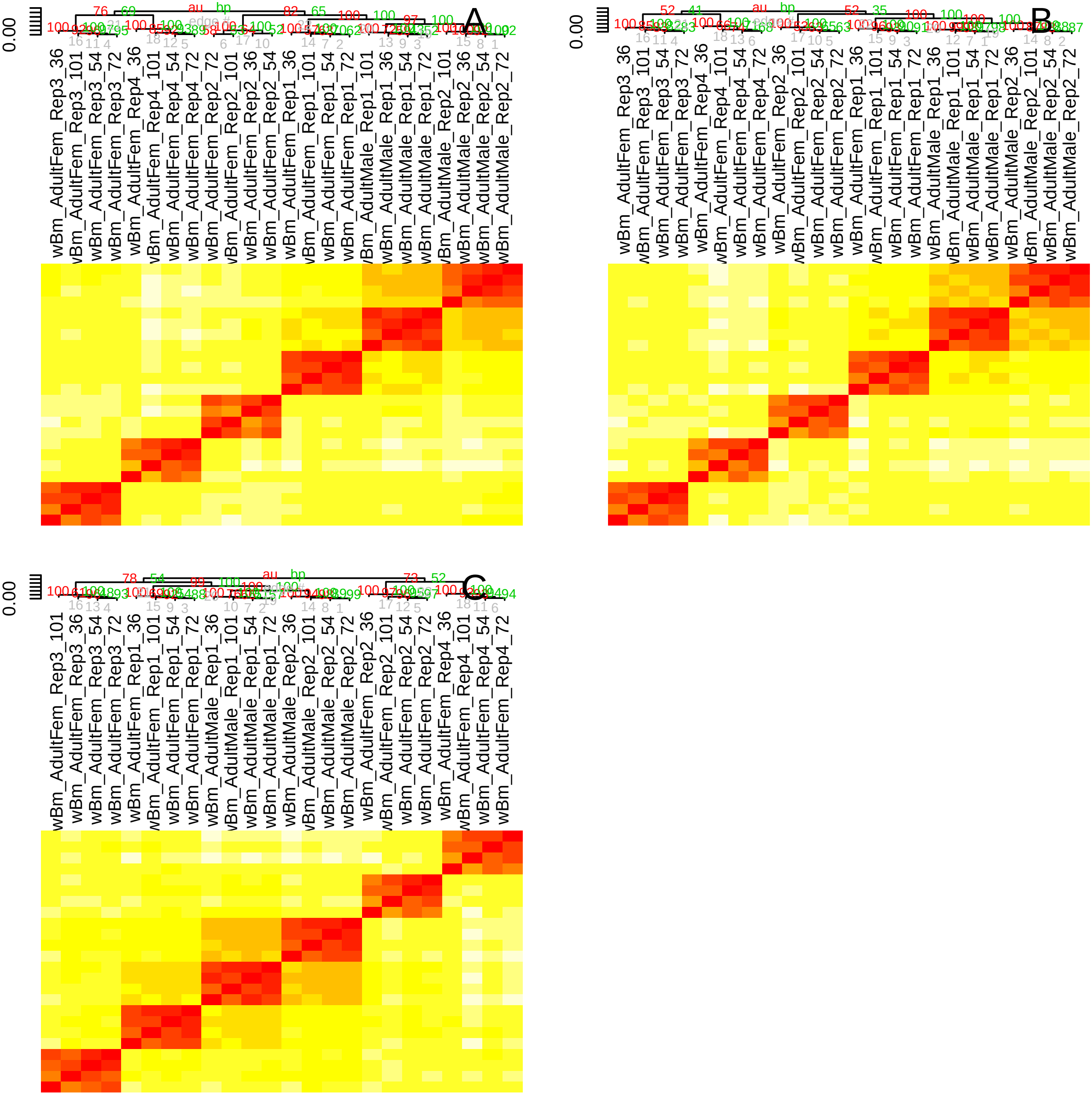
A
B
C
au
bp
edge #
0.00
wBm_AdultFem_Rep3_36
wBm_AdultFem_Rep3_101
wBm_AdultFem_Rep3_54
wBm_AdultFem_Rep3_72
wBm_AdultFem_Rep4_36
wBm_AdultFem_Rep4_101
wBm_AdultFem_Rep4_54
wBm_AdultFem_Rep4_72
wBm_AdultFem_Rep2_72
wBm_AdultFem_Rep2_101
wBm_AdultFem_Rep2_36
wBm_AdultFem_Rep2_54
wBm_AdultFem_Rep1_36
wBm_AdultFem_Rep1_101
wBm_AdultFem_Rep1_54
wBm_AdultFem_Rep1_72
wBm_AdultMale_Rep1_101
wBm_AdultMale_Rep1_36
wBm_AdultMale_Rep1_54
wBm_AdultMale_Rep1_72
wBm_AdultMale_Rep2_101
wBm_AdultMale_Rep2_36
wBm_AdultMale_Rep2_54
wBm_AdultMale_Rep2_72

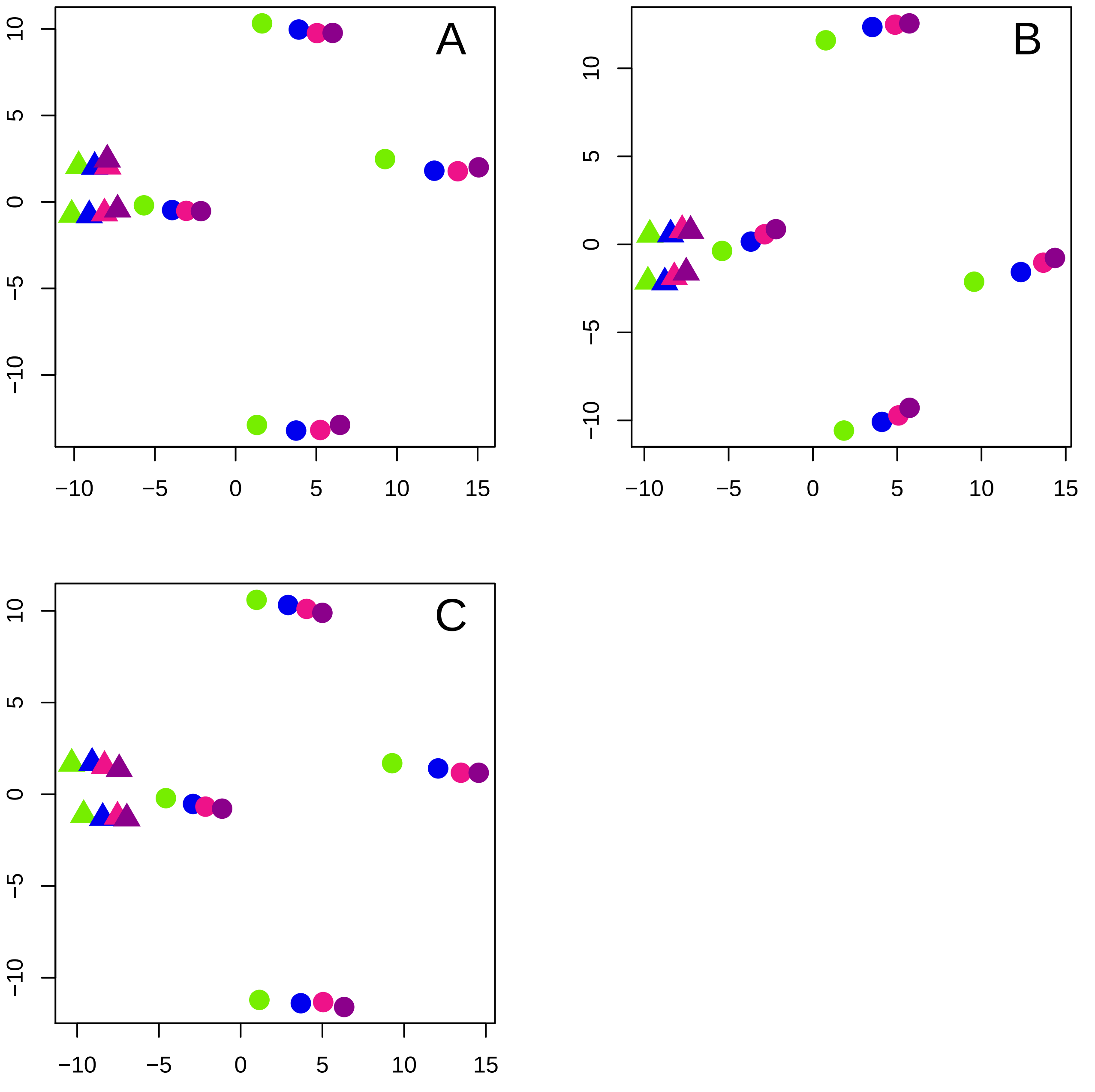

A
B
C
10
5
0
−5
−10
15

Additional File 33. Compendium of figures for *Es. coli* data with results separated by read length using the BWA MEM aligner. A heatmap with hierarchical clustering with statistical support is shown on page 1 with the condition denoted according to letter code from Supplementary Table 2, followed by the replicate designation and the pairing status such that (0) paired reads, (1) first-in-read single end read, and (2) second-in-read single end read. A PCA plot is shown on page 2 where the conditions are denoted by the shape (circle, DMEM; triangle, LB) and the pairing status by the color (green, paired end; blue, first-in-pair single end read; magenta, second-in-pair single end read). On both pages, results are shown in the four panels: (A) 36-bp reads, (B) 54-bp reads, (C) 72-bp reads, and (D) 101-bp reads. To compare the results from BWA MEM to those with Bowtie, these figures would be compared to Additional File 20.

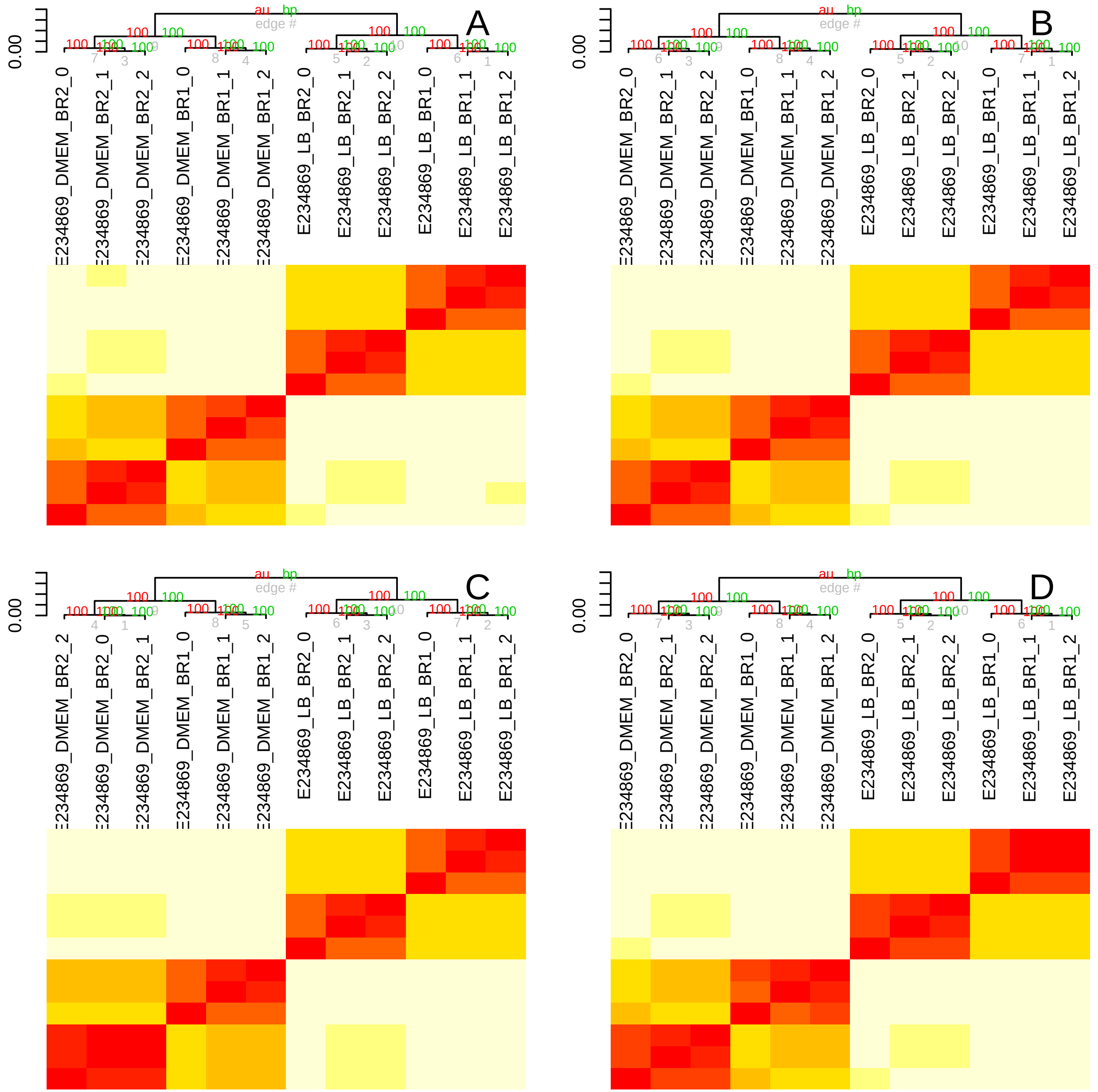
A
au
bp
edge #
0.00
E234869_DMEM_BR2_0
E234869_DMEM_BR2_1
E234869_DMEM_BR2_2
E234869_DMEM_BR1_0
E234869_DMEM_BR1_1
E234869_DMEM_BR1_2
E234869_LB_BR2_0
E234869_LB_BR2_1
E234869_LB_BR2_2
E234869_LB_BR1_0
E234869_LB_BR1_1
E234869_LB_BR1_2
B
au
bp
edge #
0.00
E234869_DMEM_BR2_0
E234869_DMEM_BR2_1
E234869_DMEM_BR2_2
E234869_DMEM_BR1_0
E234869_DMEM_BR1_1
E234869_DMEM_BR1_2
E234869_LB_BR2_0
E234869_LB_BR2_1
E234869_LB_BR2_2
E234869_LB_BR1_0
E234869_LB_BR1_1
E234869_LB_BR1_2
C
au
bp
edge #
0.00
E234869_DMEM_BR2_2
E234869_DMEM_BR2_0
E234869_DMEM_BR2_1
E234869_DMEM_BR1_0
E234869_DMEM_BR1_1
E234869_DMEM_BR1_2
E234869_LB_BR2_0
E234869_LB_BR2_1
E234869_LB_BR2_2
E234869_LB_BR1_0
E234869_LB_BR1_1
E234869_LB_BR1_2
D
au
bp
edge #
0.00
E234869_DMEM_BR2_0
E234869_DMEM_BR2_1
E234869_DMEM_BR2_2
E234869_DMEM_BR1_0
E234869_DMEM_BR1_1
E234869_DMEM_BR1_2
E234869_LB_BR2_0
E234869_LB_BR2_1
E234869_LB_BR2_2
E234869_LB_BR1_0
E234869_LB_BR1_1
E234869_LB_BR1_2

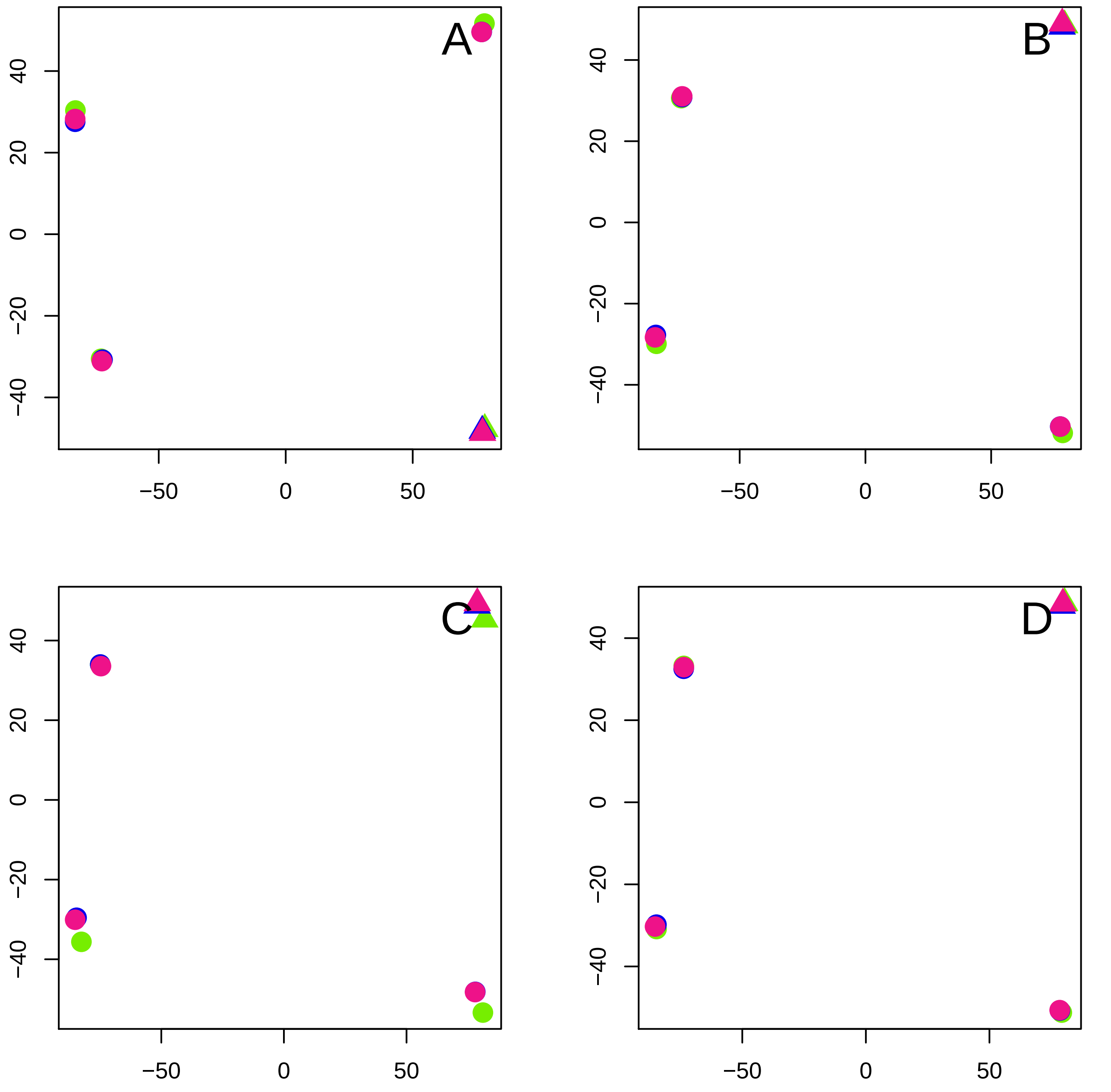

A
B
C
D
40
20
0
−20
−40
−50
0
50

Additional File 34. Compendium of figures for data from the *Wolbachia* endosymbiont *w*Bm in adult male and adult female *B. malayi* hosts with results separated by read length using the BWA MEM aligner. A heatmap with hierarchical clustering with statistical support is shown on page 1 with the condition denoted according to letter code from Supplementary Table 2, followed by the replicate designation and the pairing status such that (0) paired reads, (1) first-in-read single end read, and (2) second-in-read single end read. A PCA plot is shown on page 2 where the conditions are denoted by the shape and the pairing status by the color (green, paired end; blue, first-in-pair single end read; magenta, second-in-pair single end read). On both pages, results are shown in the four panels: (A) 36-bp reads, (B) 54-bp reads, (C) 72-bp reads, and (D) 101-bp reads. To compare the results from BWA MEM to those with Bowtie, these figures would be compared to Additional File 23.

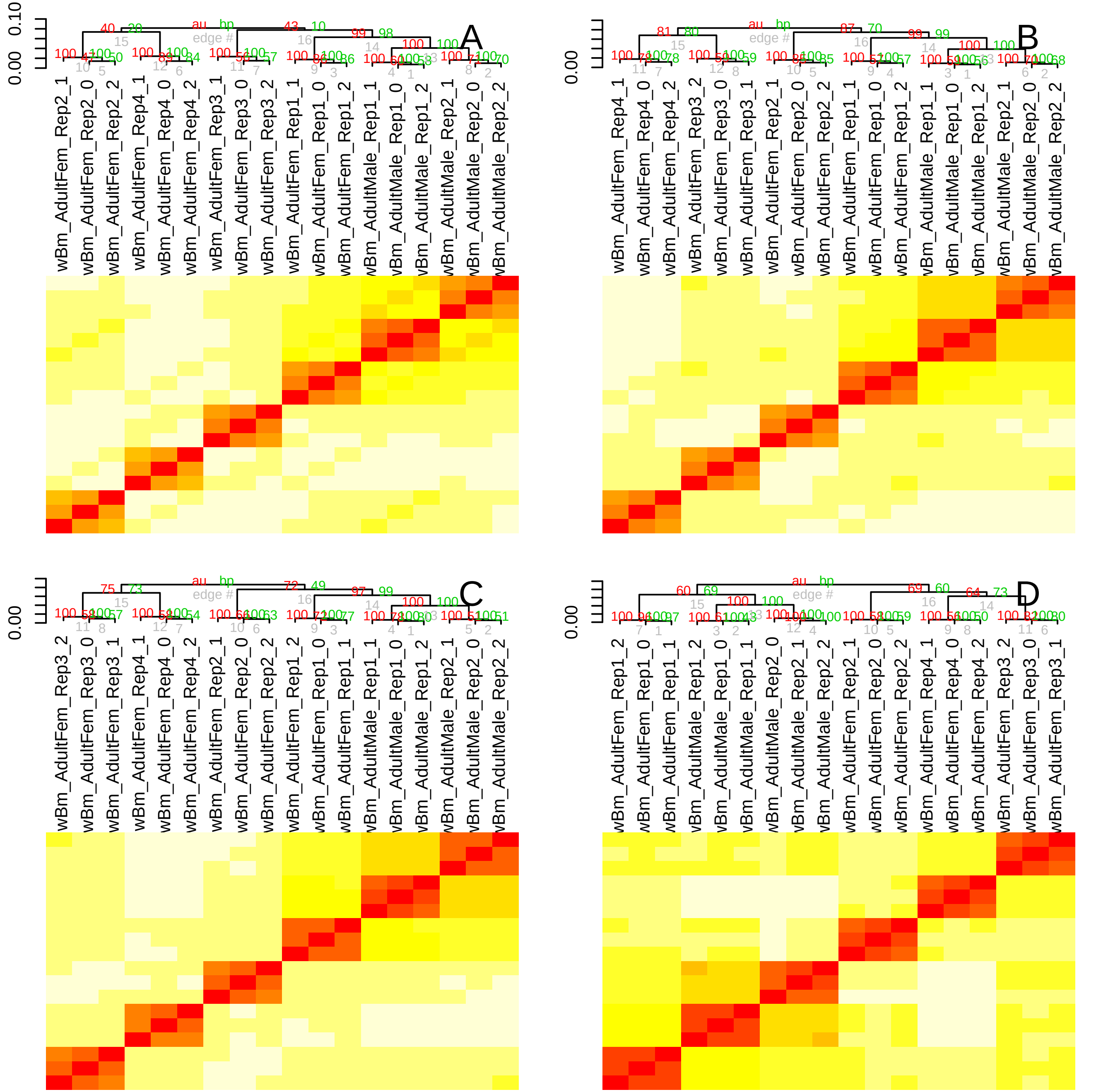
A
0.00
0.10
au
bp
edge #
wBm_AdultFem_Rep2_1
wBm_AdultFem_Rep2_0
wBm_AdultFem_Rep2_2
wBm_AdultFem_Rep4_1
wBm_AdultFem_Rep4_0
wBm_AdultFem_Rep4_2
wBm_AdultFem_Rep3_1
wBm_AdultFem_Rep3_0
wBm_AdultFem_Rep3_2
wBm_AdultFem_Rep1_1
wBm_AdultFem_Rep1_0
wBm_AdultFem_Rep1_2
wBm_AdultMale_Rep1_1
wBm_AdultMale_Rep1_0
wBm_AdultMale_Rep1_2
wBm_AdultMale_Rep2_1
wBm_AdultMale_Rep2_0
wBm_AdultMale_Rep2_2
B
0.00
au
bp
edge #
wBm_AdultFem_Rep4_1
wBm_AdultFem_Rep4_0
wBm_AdultFem_Rep4_2
wBm_AdultFem_Rep3_2
wBm_AdultFem_Rep3_0
wBm_AdultFem_Rep3_1
wBm_AdultFem_Rep2_1
wBm_AdultFem_Rep2_0
wBm_AdultFem_Rep2_2
wBm_AdultFem_Rep1_1
wBm_AdultFem_Rep1_0
wBm_AdultFem_Rep1_2
wBm_AdultMale_Rep1_1
wBm_AdultMale_Rep1_0
wBm_AdultMale_Rep1_2
wBm_AdultMale_Rep2_1
wBm_AdultMale_Rep2_0
wBm_AdultMale_Rep2_2
C
0.00
au
bp
edge #
wBm_AdultFem_Rep3_2
wBm_AdultFem_Rep3_0
wBm_AdultFem_Rep3_1
wBm_AdultFem_Rep4_1
wBm_AdultFem_Rep4_0
wBm_AdultFem_Rep4_2
wBm_AdultFem_Rep2_1
wBm_AdultFem_Rep2_0
wBm_AdultFem_Rep2_2
wBm_AdultFem_Rep1_2
wBm_AdultFem_Rep1_0
wBm_AdultFem_Rep1_1
wBm_AdultMale_Rep1_1
wBm_AdultMale_Rep1_0
wBm_AdultMale_Rep1_2
wBm_AdultMale_Rep2_1
wBm_AdultMale_Rep2_0
wBm_AdultMale_Rep2_2
D
0.00
au
bp
edge #
wBm_AdultFem_Rep1_2
wBm_AdultFem_Rep1_0
wBm_AdultFem_Rep1_1
wBm_AdultMale_Rep1_2
wBm_AdultMale_Rep1_0
wBm_AdultMale_Rep1_1
wBm_AdultMale_Rep2_0
wBm_AdultMale_Rep2_1
wBm_AdultMale_Rep2_2
wBm_AdultFem_Rep2_1
wBm_AdultFem_Rep2_0
wBm_AdultFem_Rep2_2
wBm_AdultFem_Rep4_1
wBm_AdultFem_Rep4_0
wBm_AdultFem_Rep4_2
wBm_AdultFem_Rep3_2
wBm_AdultFem_Rep3_0
wBm_AdultFem_Rep3_1

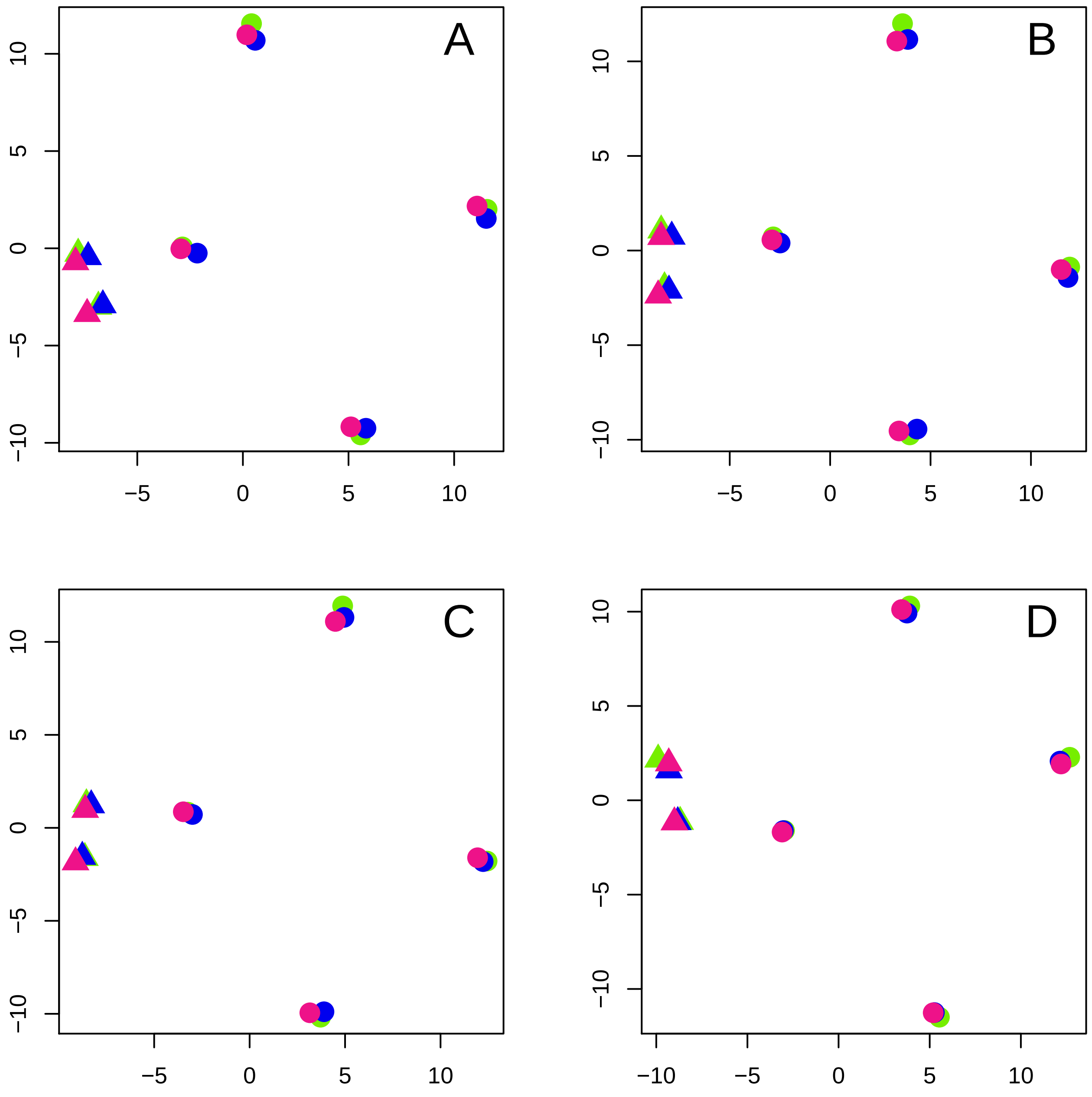
A
B
C
D

Additional File 35. Compendium of scatterplots for all data sets aligned with BWA MEM with results aggregated by read length. The differentially expressed genes identified using an adjusted p-value (FDR) cutoff ≤0.05 at varying read lengths within a dataset were compared using Pearson's correlation implemented in the R statistical tool and illustrated as a matrix of scatterplots. The diagonal represents the histogram of log-transformed fold-changes within the comparison. The lower plots represent the correlation between comparisons with singleton DEGs identified for comparisons on the x-axis (pink) and y-axis (green). Genes with FDR > 0.05 in both comparisons are not shown. The upper portion of the plot lists the corresponding Pearson's correlation coefficient and the number of singleton DEGs identified in each comparison. Each scatterplot is labeled by the comparison according to the letter code from Supplementary Table 2. A separate plot is shown for paired reads (labelled "0"), first read in pair (labelled "1"), and second read in pair (labelled "2").

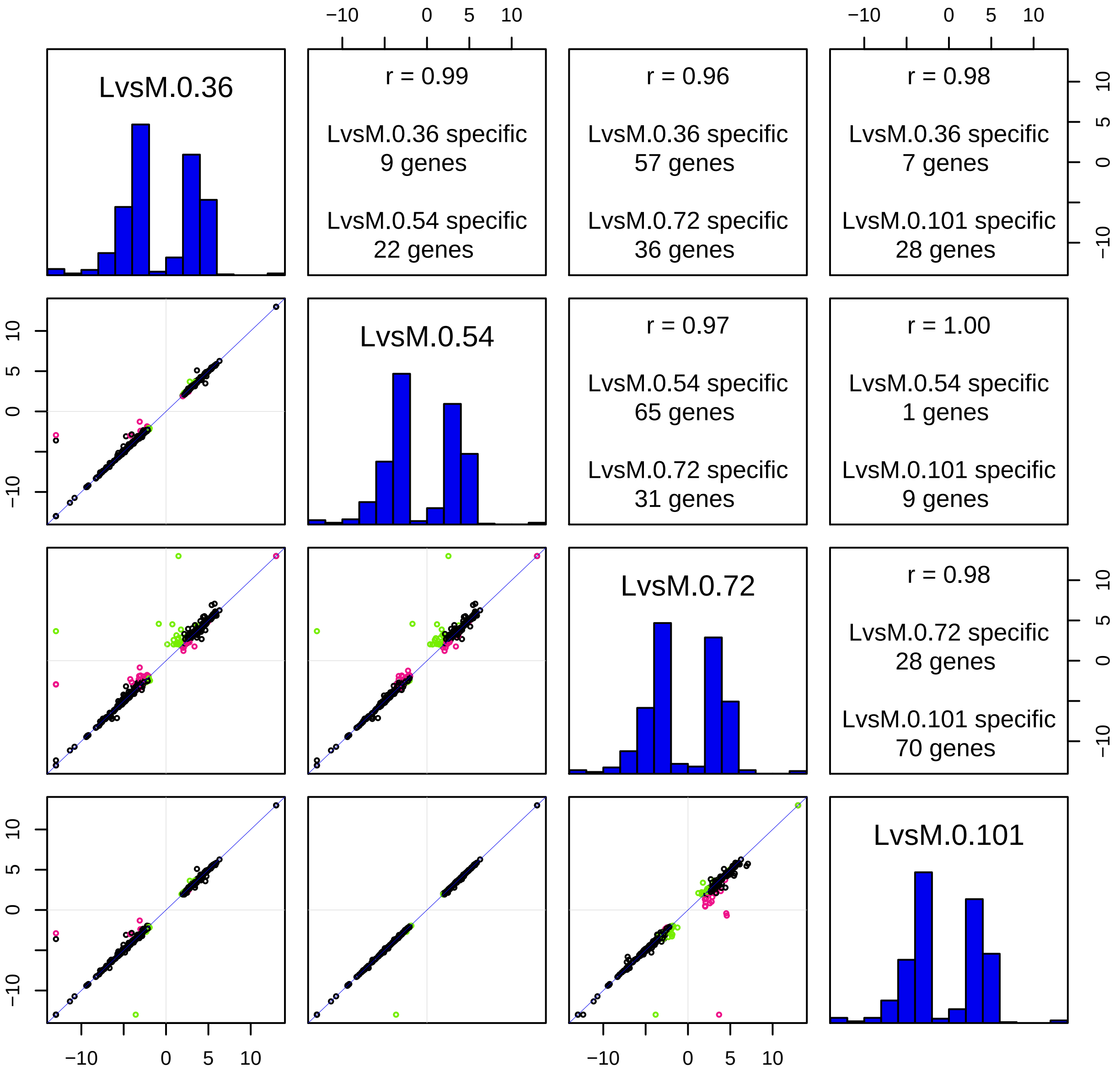
LvsM.0.36
r = 0.99
LvsM.0.36 specific
9 genes
LvsM.0.54 specific
22 genes
r = 0.96
LvsM.0.36 specific
57 genes
LvsM.0.72 specific
36 genes
r = 0.98
LvsM.0.36 specific
7 genes
LvsM.0.101 specific
28 genes
LvsM.0.54
r = 0.97
LvsM.0.54 specific
65 genes
LvsM.0.72 specific
31 genes
r = 1.00
LvsM.0.54 specific
1 genes
LvsM.0.101 specific
9 genes
LvsM.0.72
r = 0.98
LvsM.0.72 specific
28 genes
LvsM.0.101 specific
70 genes
LvsM.0.101
−10
0
5
10

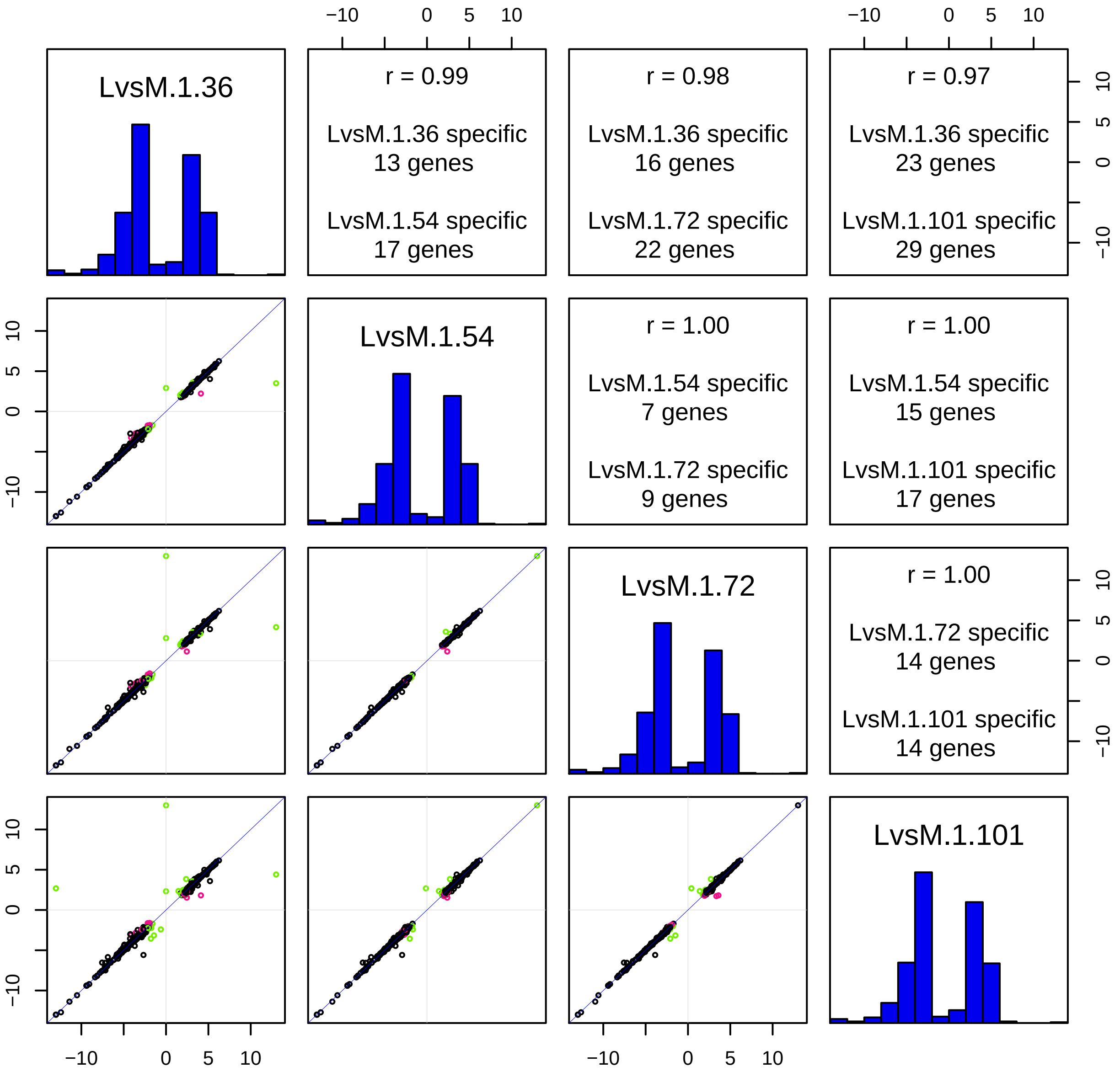

LvsM.1.36
r = 0.99
LvsM.1.36 specific
13 genes
LvsM.1.54 specific
17 genes
r = 0.98
LvsM.1.36 specific
16 genes
LvsM.1.72 specific
22 genes
r = 0.97
LvsM.1.36 specific
23 genes
LvsM.1.101 specific
29 genes
LvsM.1.54
r = 1.00
LvsM.1.54 specific
7 genes
LvsM.1.72 specific
9 genes
r = 1.00
LvsM.1.54 specific
15 genes
LvsM.1.101 specific
17 genes
LvsM.1.72
r = 1.00
LvsM.1.72 specific
14 genes
LvsM.1.101 specific
14 genes
LvsM.1.101
−10
0
5
10

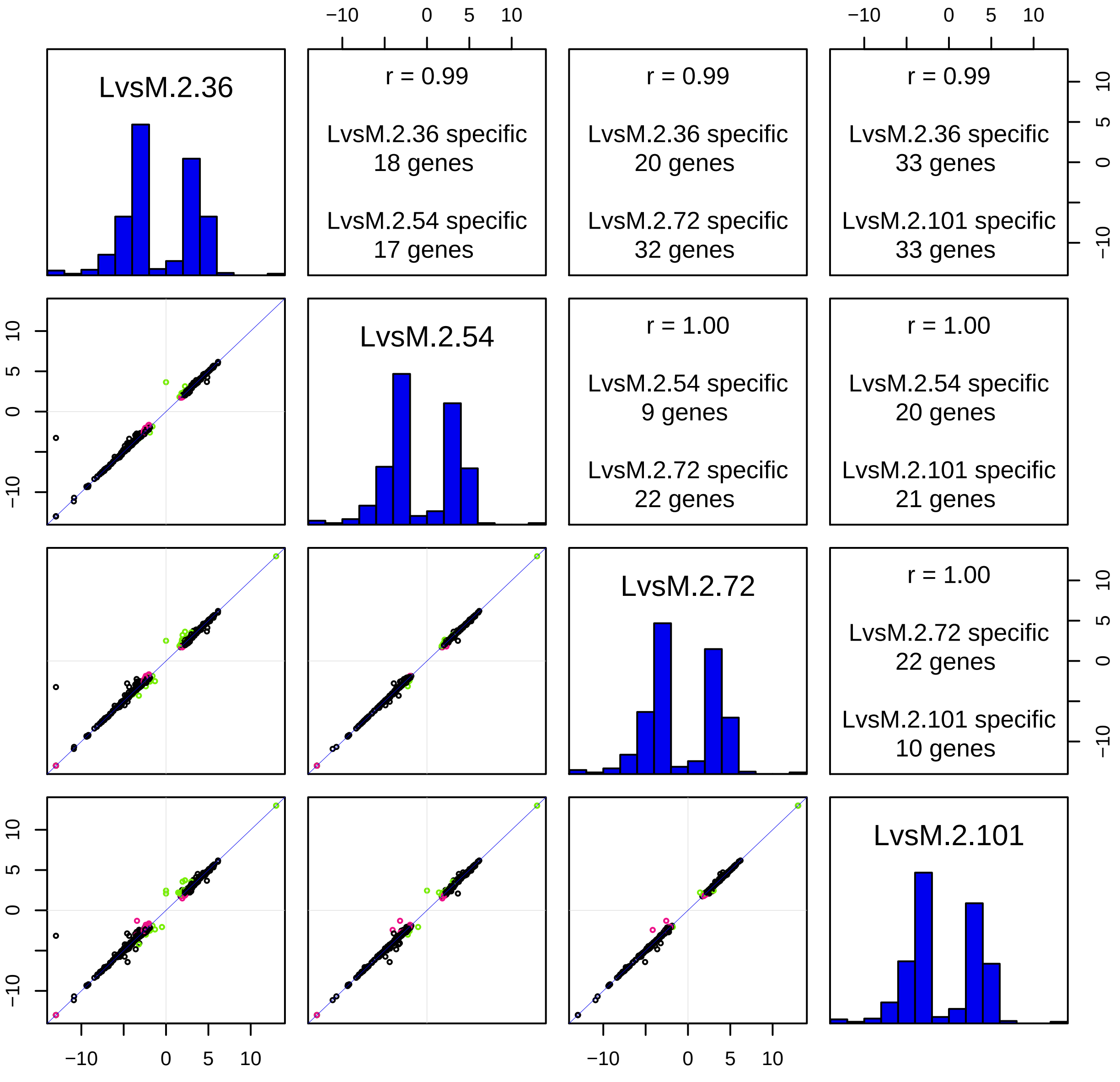
LvsM.2.36
r = 0.99
LvsM.2.36 specific
18 genes
LvsM.2.54 specific
17 genes
r = 0.99
LvsM.2.36 specific
20 genes
LvsM.2.72 specific
32 genes
r = 0.99
LvsM.2.36 specific
33 genes
LvsM.2.101 specific
33 genes
LvsM.2.54
r = 1.00
LvsM.2.54 specific
9 genes
LvsM.2.72 specific
22 genes
r = 1.00
LvsM.2.54 specific
20 genes
LvsM.2.101 specific
21 genes
LvsM.2.72
r = 1.00
LvsM.2.72 specific
22 genes
LvsM.2.101 specific
10 genes
LvsM.2.101
−10
0
5
10

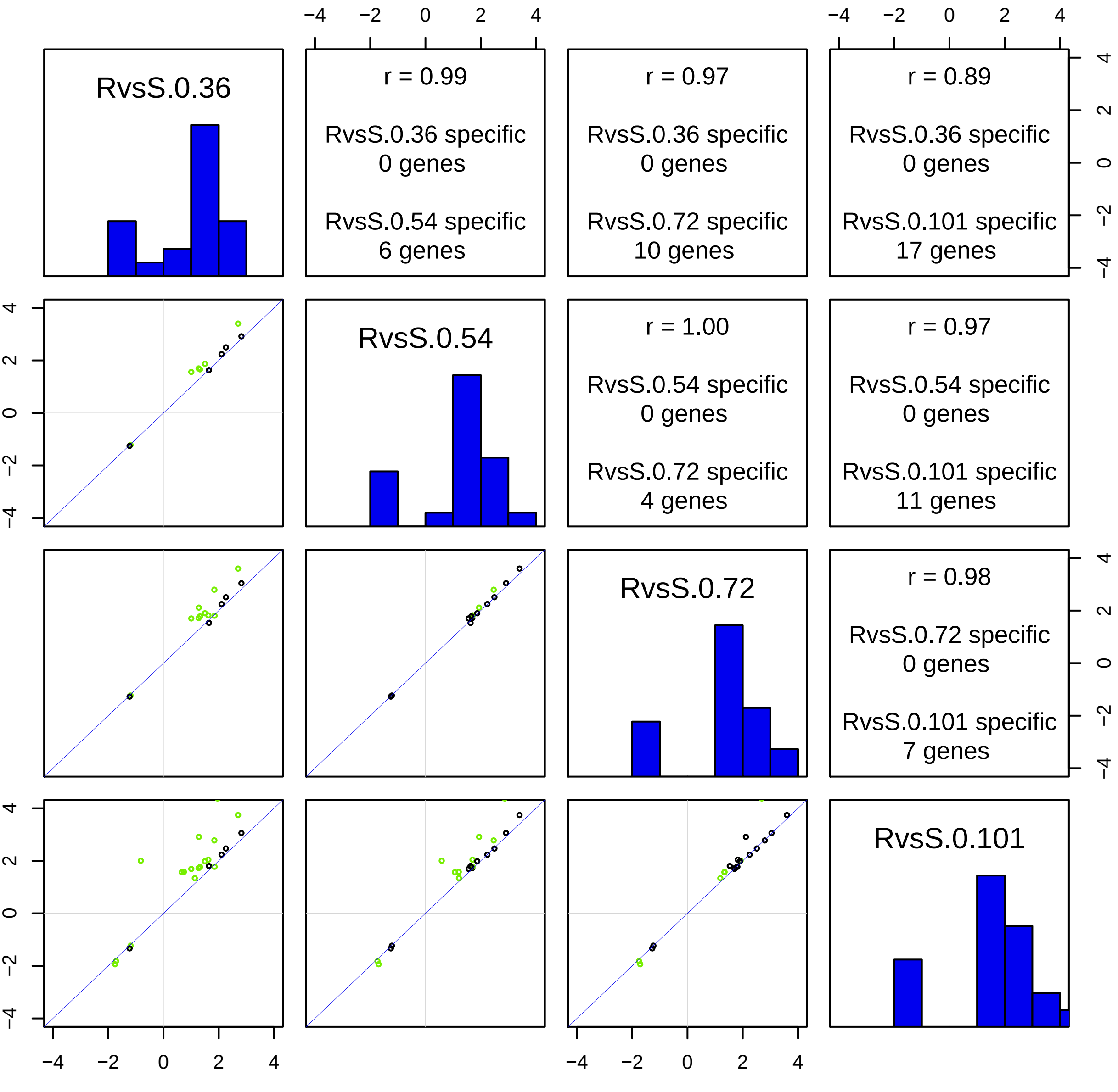

RvsS.0.36
r = 0.99
RvsS.0.36 specific
0 genes
RvsS.0.54 specific
6 genes
r = 0.97
RvsS.0.36 specific
0 genes
RvsS.0.72 specific
10 genes
r = 0.89
RvsS.0.36 specific
0 genes
RvsS.0.101 specific
17 genes
RvsS.0.54
r = 1.00
RvsS.0.54 specific
0 genes
RvsS.0.72 specific
4 genes
r = 0.97
RvsS.0.54 specific
0 genes
RvsS.0.101 specific
11 genes
RvsS.0.72
r = 0.98
RvsS.0.72 specific
0 genes
RvsS.0.101 specific
7 genes
RvsS.0.101
−4 −2 0 2 4

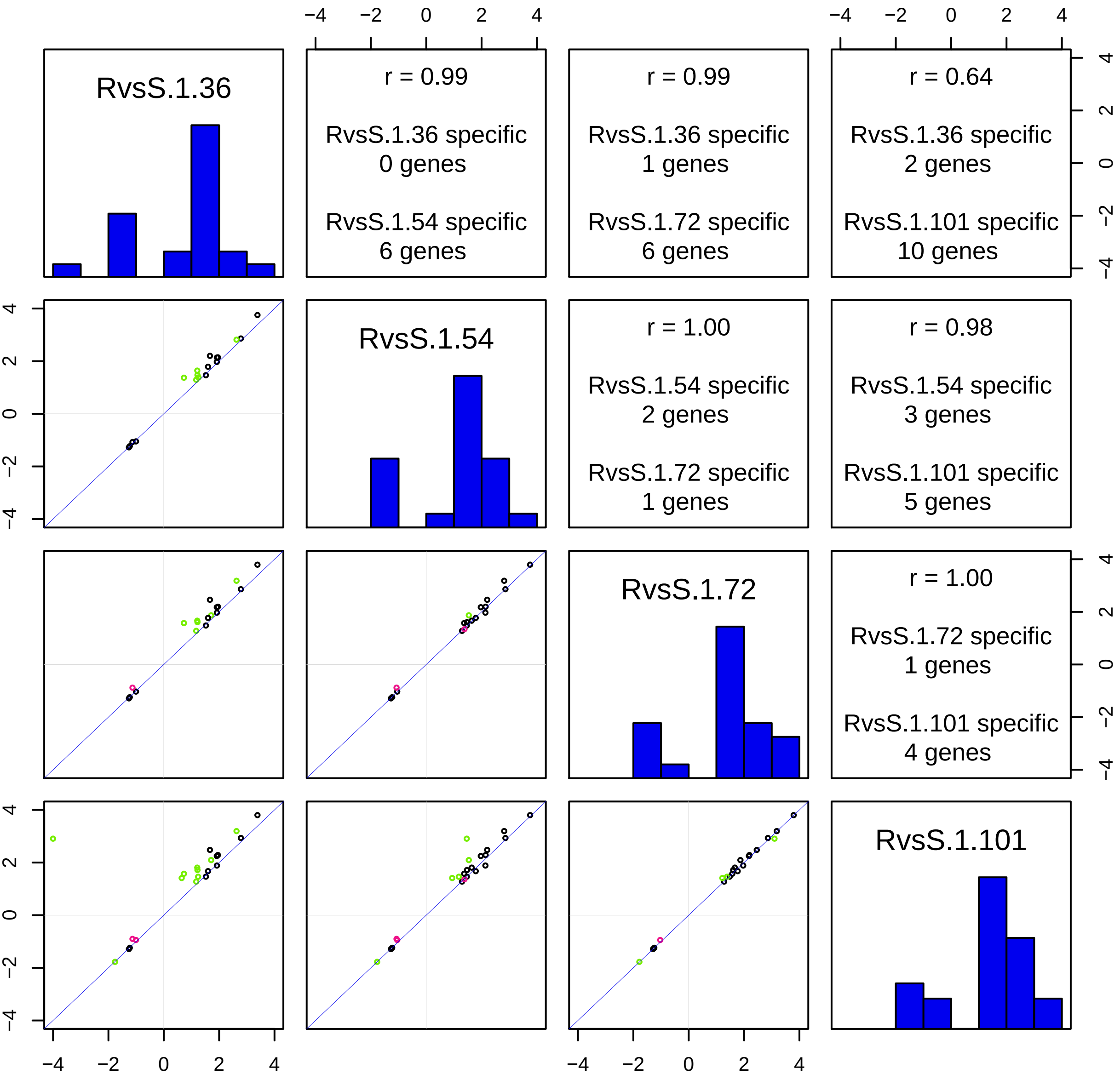

RvsS.1.36
r = 0.99
RvsS.1.36 specific
0 genes
RvsS.1.54 specific
6 genes
r = 0.99
RvsS.1.36 specific
1 genes
RvsS.1.72 specific
6 genes
r = 0.64
RvsS.1.36 specific
2 genes
RvsS.1.101 specific
10 genes
RvsS.1.54
r = 1.00
RvsS.1.54 specific
2 genes
RvsS.1.72 specific
1 genes
r = 0.98
RvsS.1.54 specific
3 genes
RvsS.1.101 specific
5 genes
RvsS.1.72
r = 1.00
RvsS.1.72 specific
1 genes
RvsS.1.101 specific
4 genes
RvsS.1.101
−4
−2
0
2
4

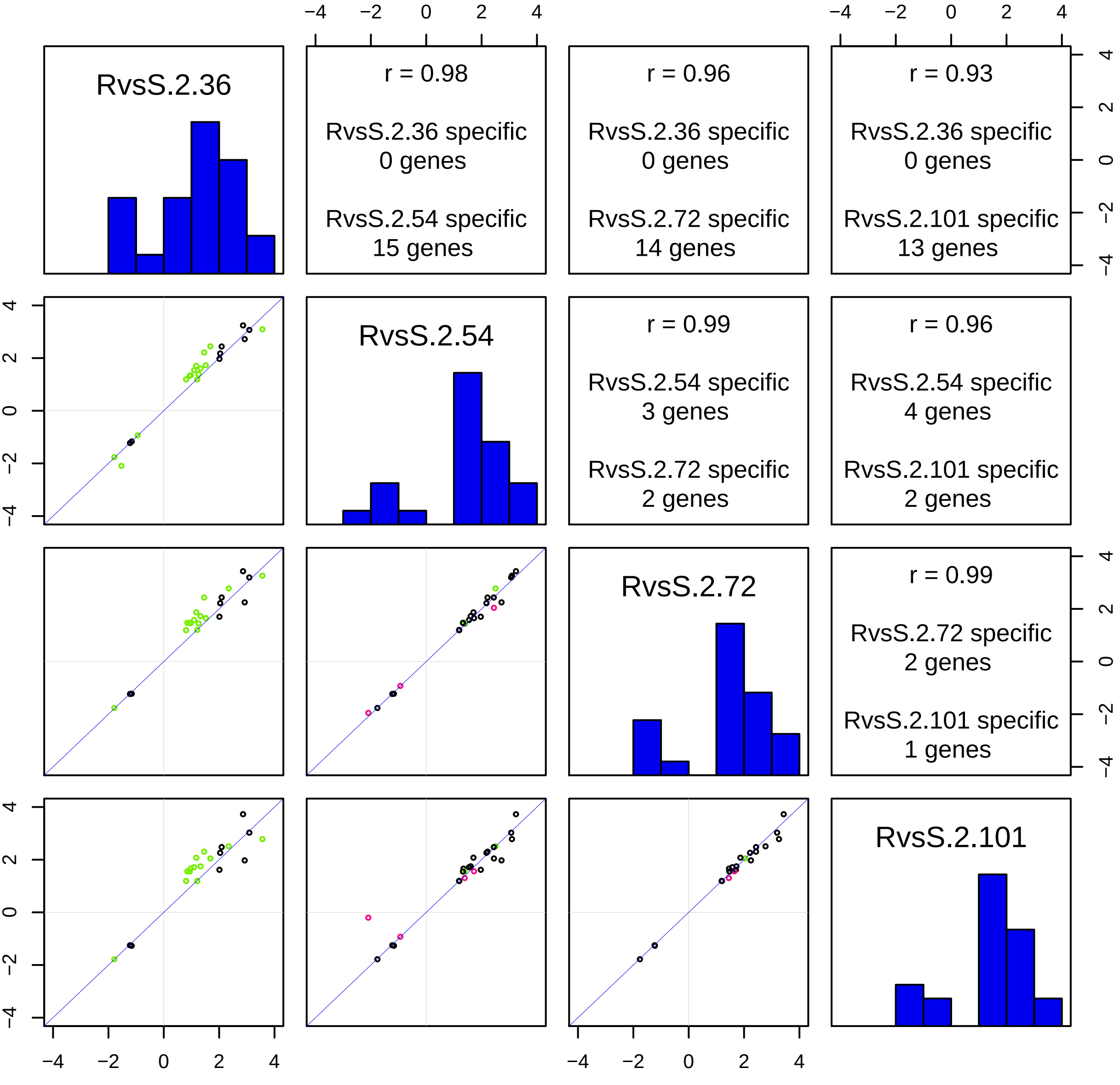
RvsS.2.36
r = 0.98
RvsS.2.36 specific
0 genes
RvsS.2.54 specific
15 genes
r = 0.96
RvsS.2.36 specific
0 genes
RvsS.2.72 specific
14 genes
r = 0.93
RvsS.2.36 specific
0 genes
RvsS.2.101 specific
13 genes
RvsS.2.54
r = 0.99
RvsS.2.54 specific
3 genes
RvsS.2.72 specific
2 genes
r = 0.96
RvsS.2.54 specific
4 genes
RvsS.2.101 specific
2 genes
RvsS.2.72
r = 0.99
RvsS.2.72 specific
2 genes
RvsS.2.101 specific
1 genes
RvsS.2.101

Additional File 36. Compendium of scatterplots for all data sets aligned with BWA MEM with results aggregated by read pairing. The differentially expressed genes identified using an adjusted p-value (FDR) cutoff ≤0.05 at varying read lengths within a dataset were compared using Pearson's correlation implemented in the R statistical tool and illustrated as a matrix of scatterplots. The diagonal represents the histogram of log-transformed fold-changes within the comparison. The lower plots represent the correlation between comparisons with singleton DEGs identified for comparisons on the x-axis (pink) and y-axis (green). Genes with FDR > 0.05 in both comparisons are not shown. The upper portion of the plot lists the corresponding Pearson's correlation coefficient and the number of singleton DEGs identified in each comparison. Each scatterplot is labeled by the comparison according to the letter code from Supplementary Table 2. A separate plot is shown for the various read lengths.

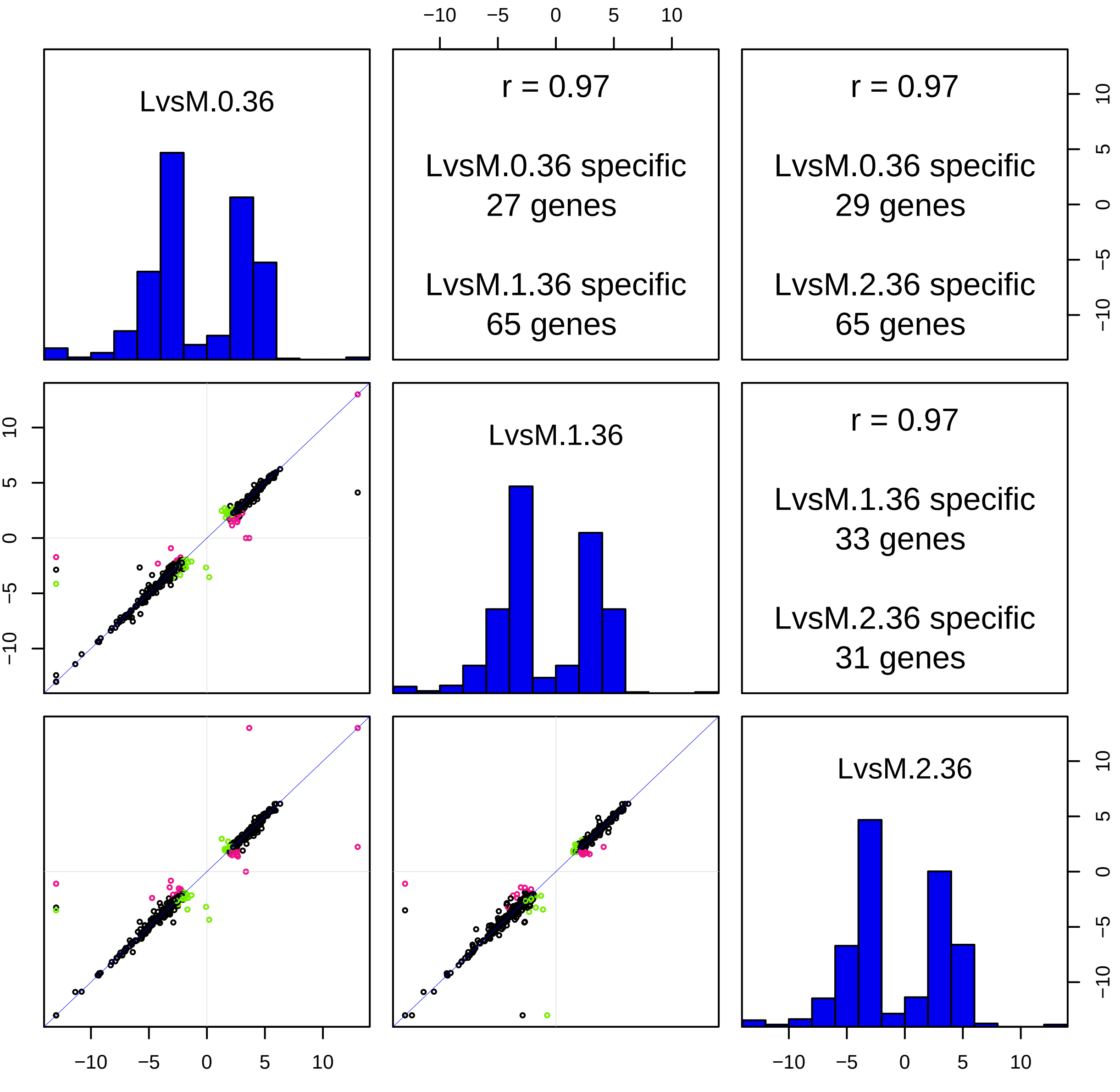
LvsM.0.36
r = 0.97
LvsM.0.36 specific
27 genes
LvsM.1.36 specific
65 genes
r = 0.97
LvsM.0.36 specific
29 genes
LvsM.2.36 specific
65 genes
LvsM.1.36
r = 0.97
LvsM.1.36 specific
33 genes
LvsM.2.36 specific
31 genes
LvsM.2.36
−10
−5
0
5
10

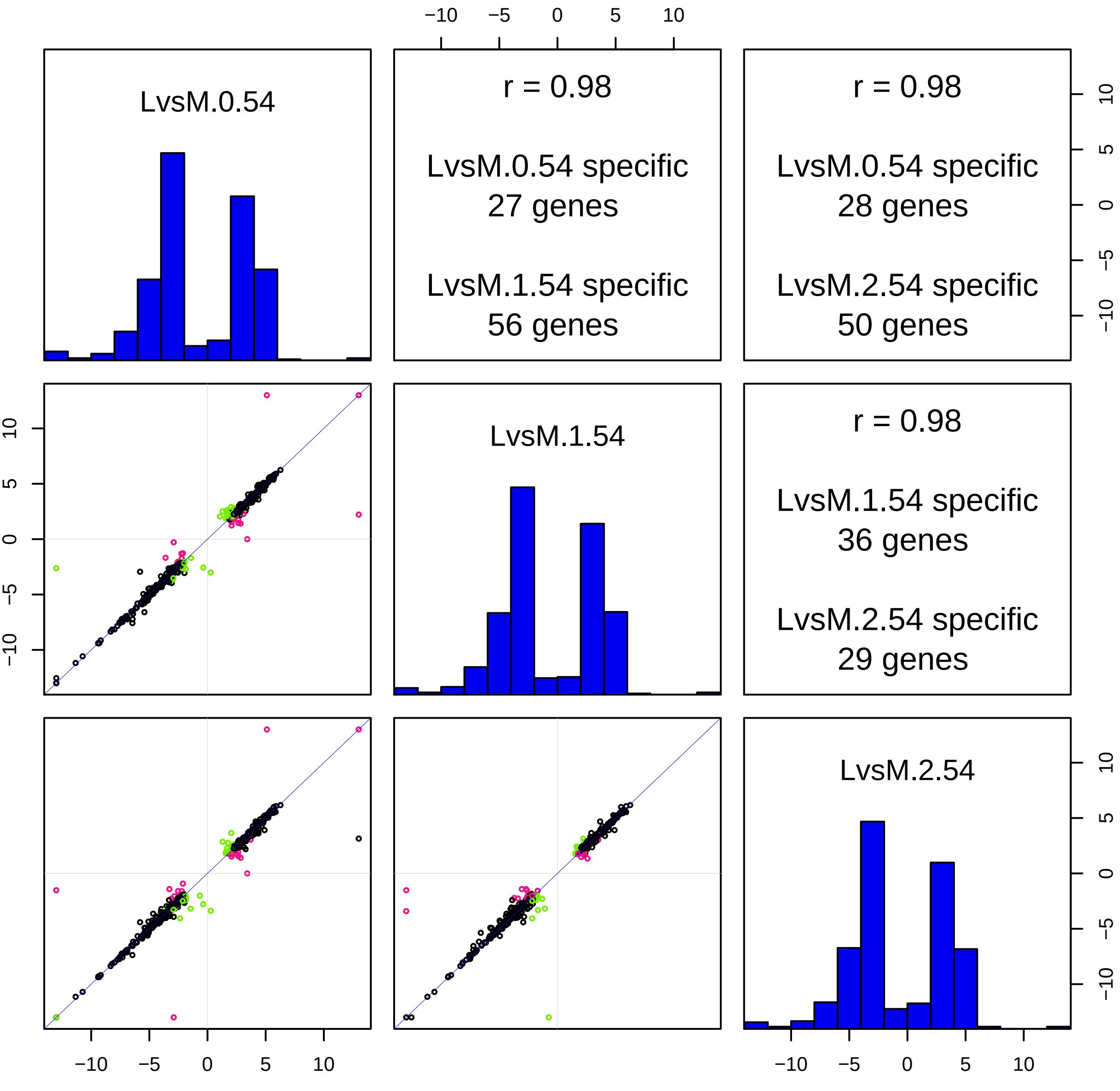
LvsM.0.54
r = 0.98
LvsM.0.54 specific
27 genes
LvsM.1.54 specific
56 genes
r = 0.98
LvsM.0.54 specific
28 genes
LvsM.2.54 specific
50 genes
LvsM.1.54
r = 0.98
LvsM.1.54 specific
36 genes
LvsM.2.54 specific
29 genes
LvsM.2.54
−10
−5
0
5
10

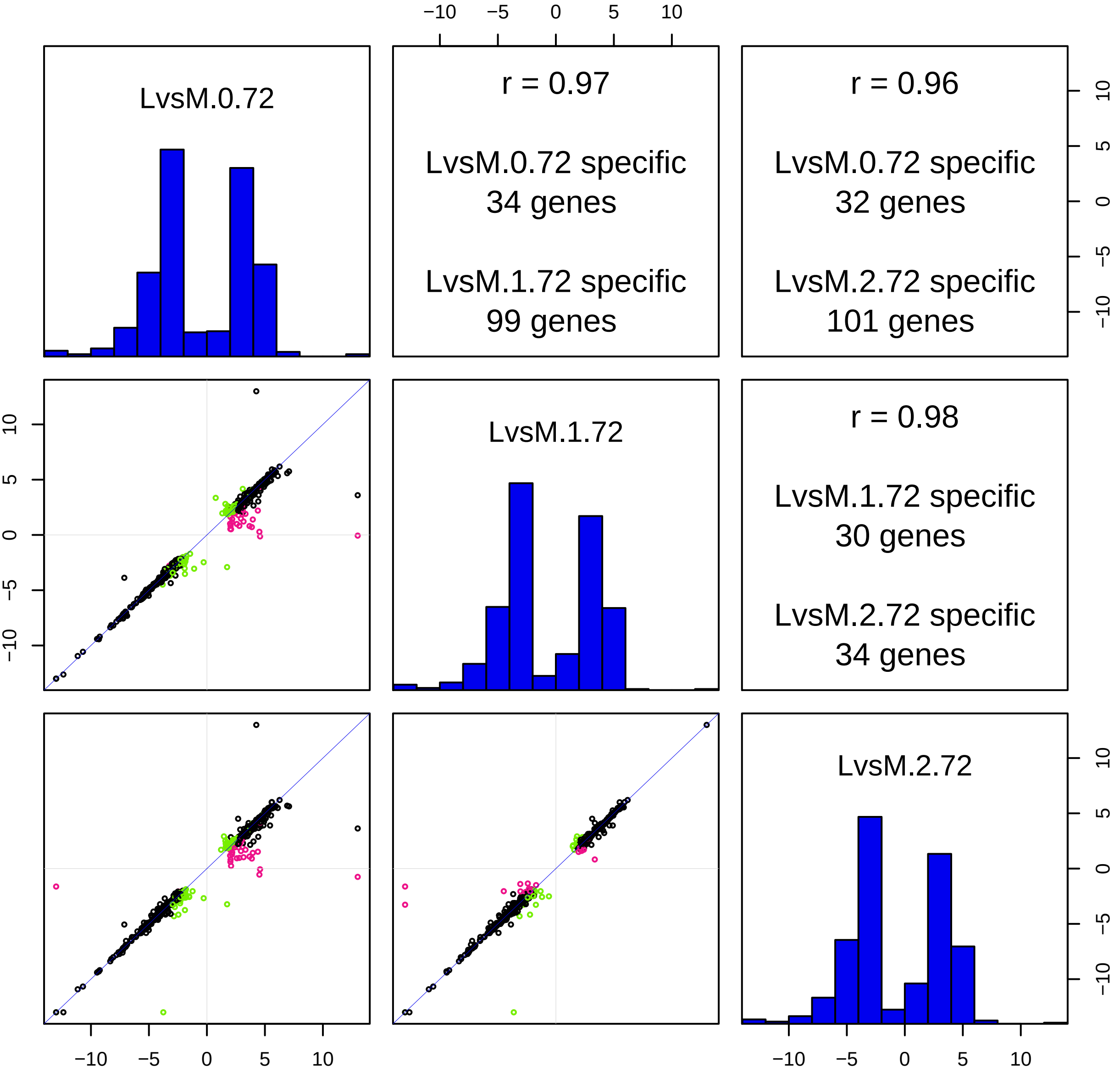

LvsM.0.72
r = 0.97
LvsM.0.72 specific
34 genes
LvsM.1.72 specific
99 genes
r = 0.96
LvsM.0.72 specific
32 genes
LvsM.2.72 specific
101 genes
LvsM.1.72
r = 0.98
LvsM.1.72 specific
30 genes
LvsM.2.72 specific
34 genes
LvsM.2.72
−10 −5 0 5 10

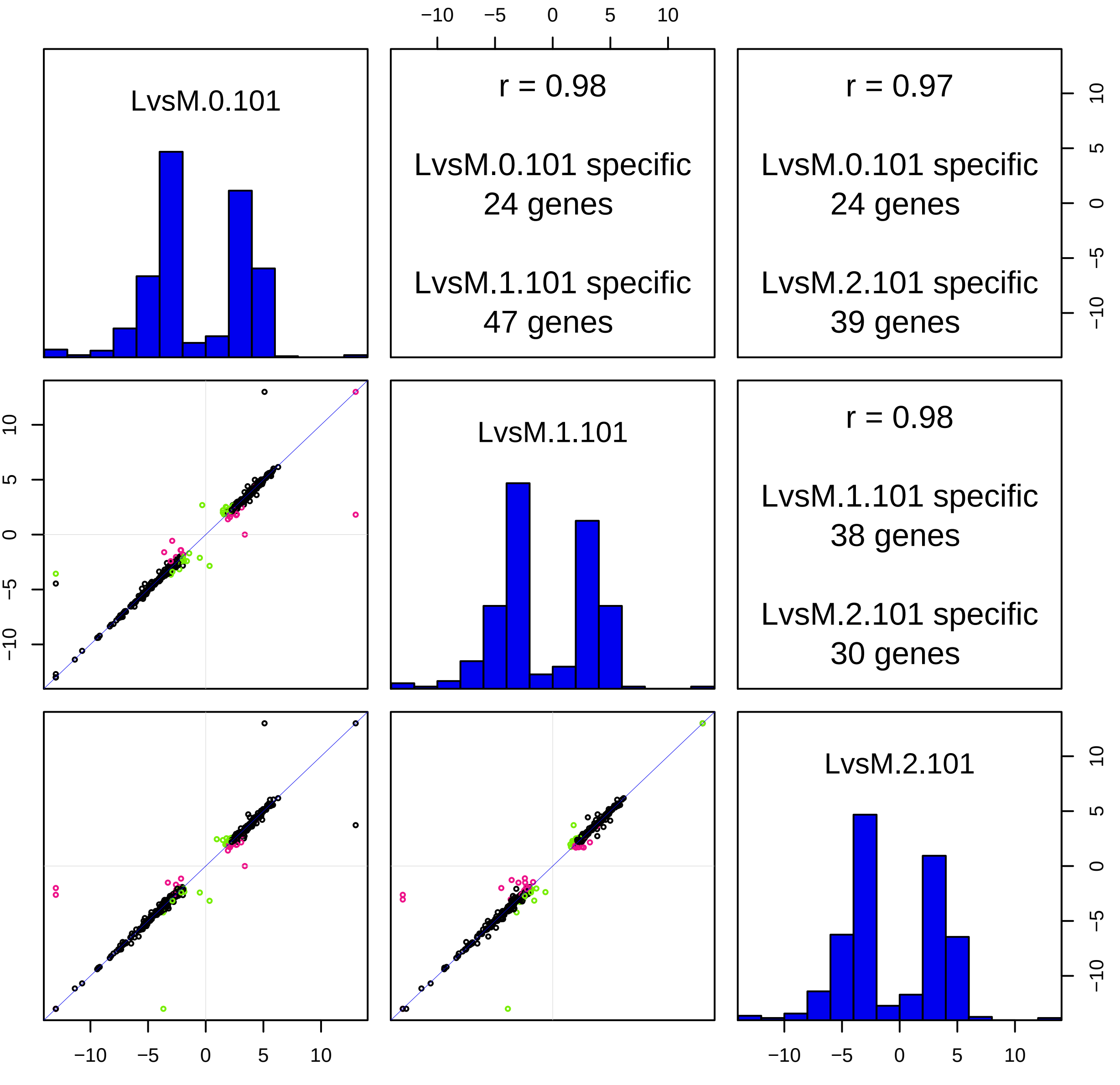
LvsM.0.101
r = 0.98
LvsM.0.101 specific
24 genes
LvsM.1.101 specific
47 genes
r = 0.97
LvsM.0.101 specific
24 genes
LvsM.2.101 specific
39 genes
LvsM.1.101
r = 0.98
LvsM.1.101 specific
38 genes
LvsM.2.101 specific
30 genes
LvsM.2.101
−10
−5
0
5
10

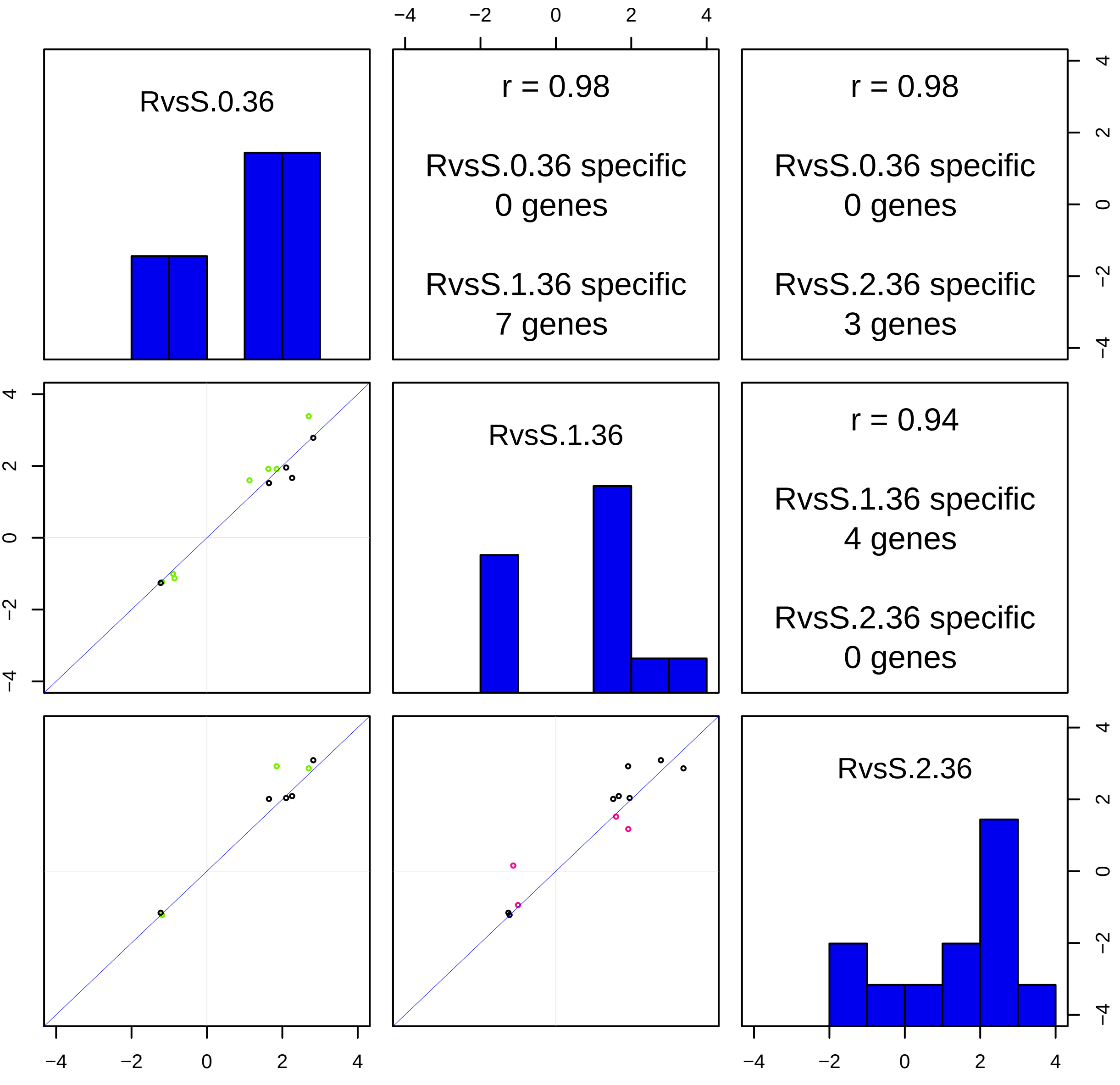
RvsS.0.36
r = 0.98
RvsS.0.36 specific
0 genes
RvsS.1.36 specific
7 genes
r = 0.98
RvsS.0.36 specific
0 genes
RvsS.2.36 specific
3 genes
RvsS.1.36
r = 0.94
RvsS.1.36 specific
4 genes
RvsS.2.36 specific
0 genes
RvsS.2.36
−4
−2
0
2
4

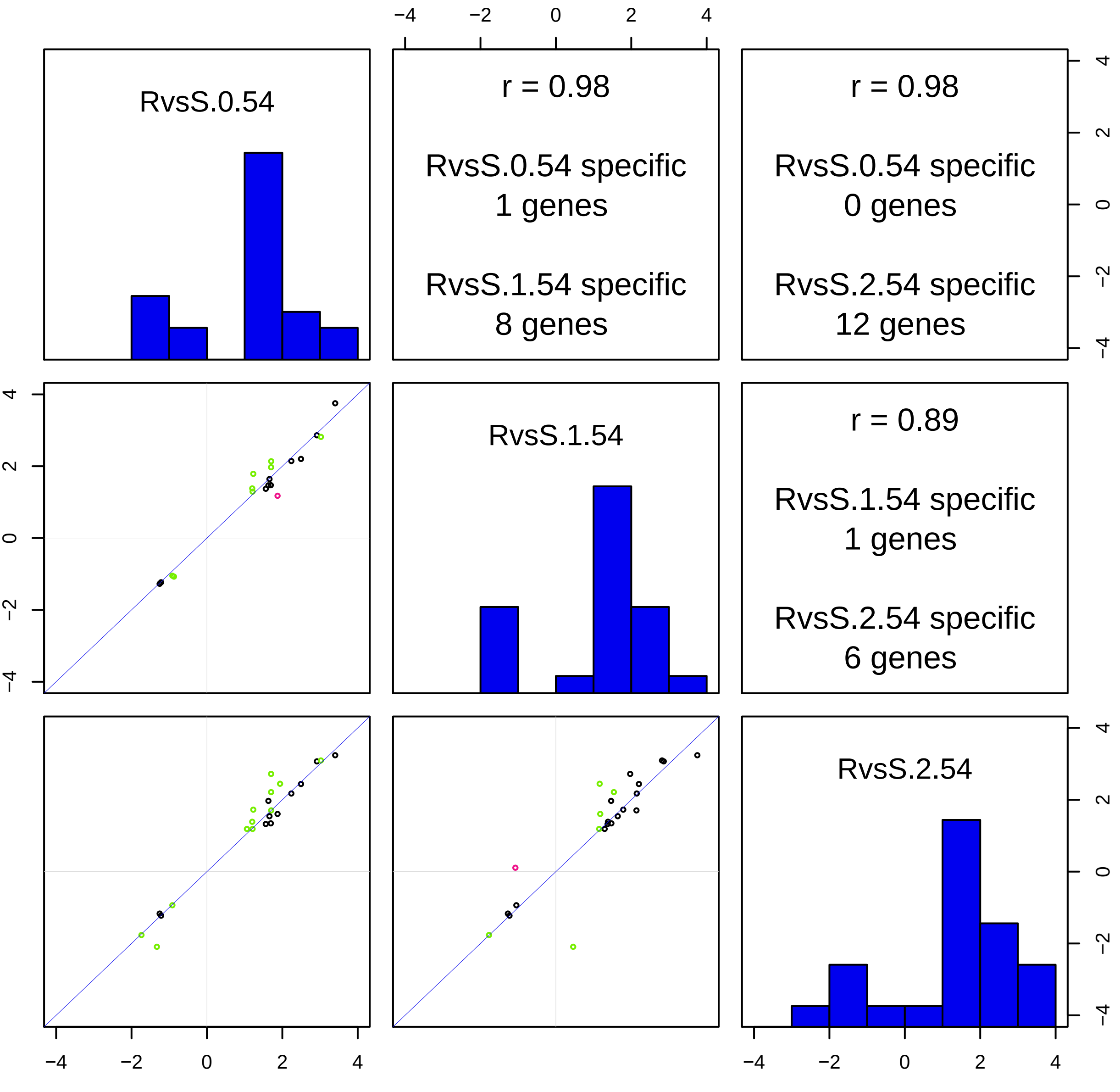

RvsS.0.54
r = 0.98
RvsS.0.54 specific
1 genes
RvsS.1.54 specific
8 genes
r = 0.98
RvsS.0.54 specific
0 genes
RvsS.2.54 specific
12 genes
RvsS.1.54
r = 0.89
RvsS.1.54 specific
1 genes
RvsS.2.54 specific
6 genes
RvsS.2.54
−4
−2
0
2
4

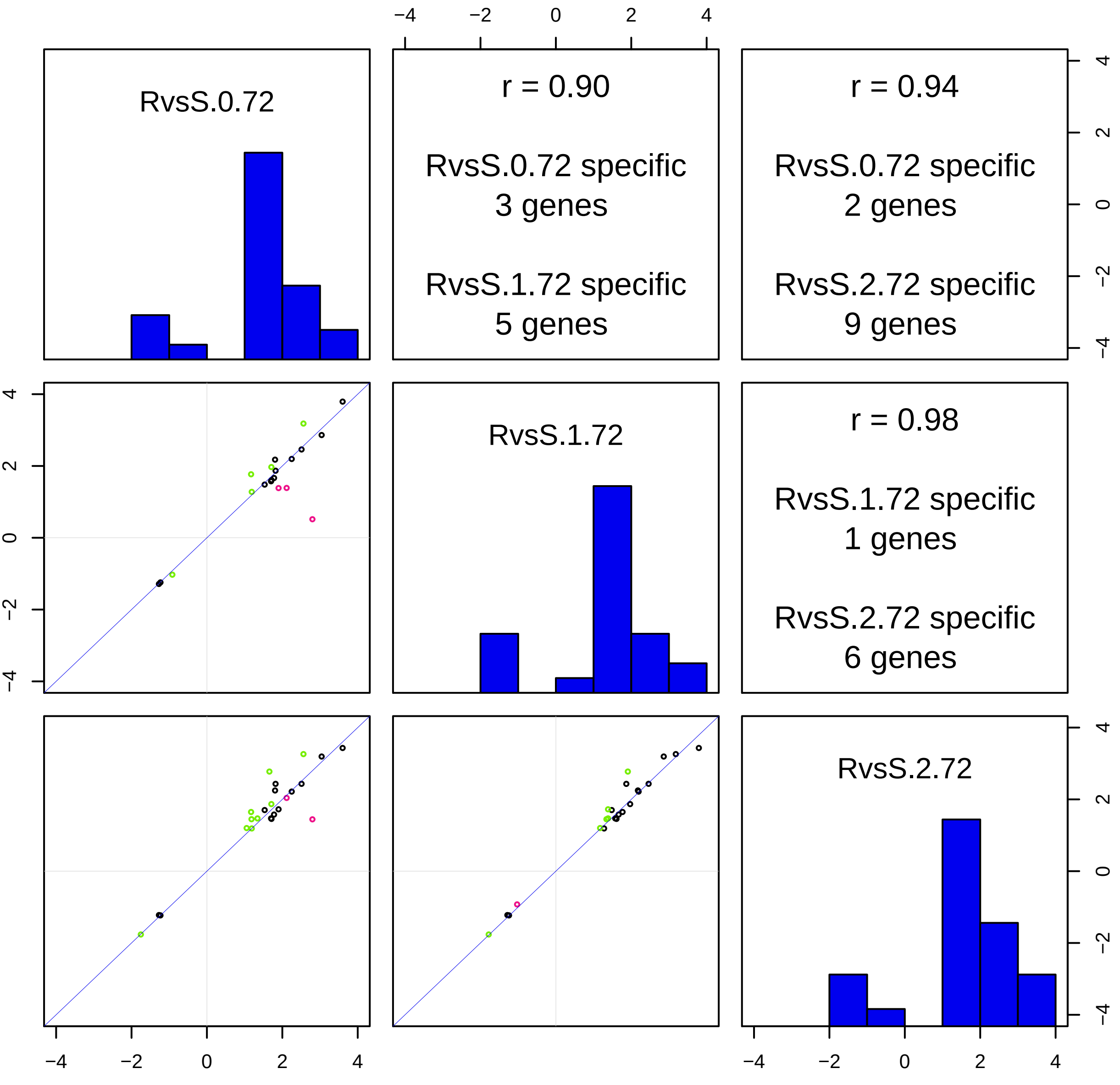
RvsS.0.72
r = 0.90
RvsS.0.72 specific
3 genes
RvsS.1.72 specific
5 genes
r = 0.94
RvsS.0.72 specific
2 genes
RvsS.2.72 specific
9 genes
RvsS.1.72
r = 0.98
RvsS.1.72 specific
1 genes
RvsS.2.72 specific
6 genes
RvsS.2.72
−4
−2
0
2
4

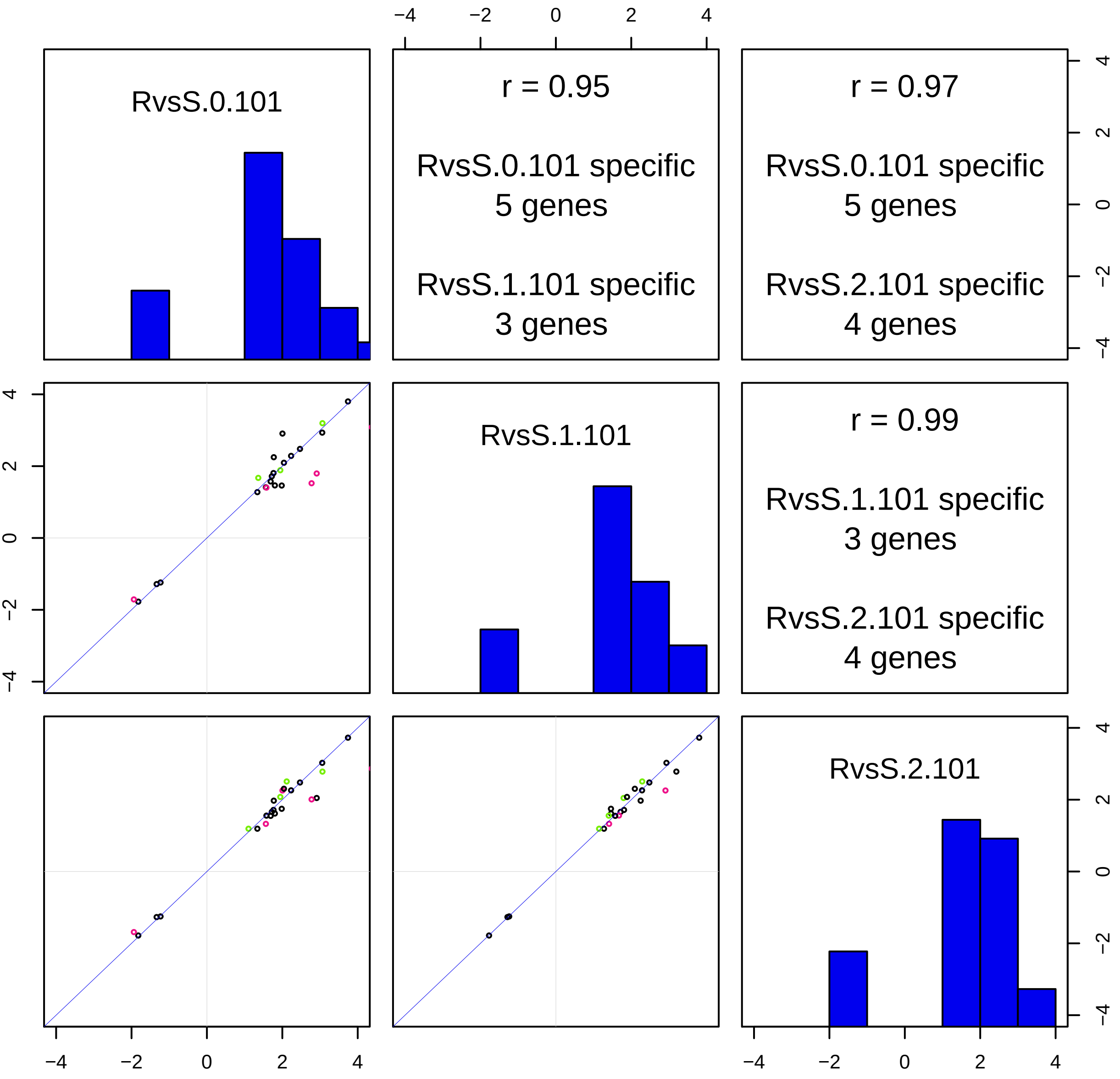
RvsS.0.101
r = 0.95
RvsS.0.101 specific
5 genes
RvsS.1.101 specific
3 genes
r = 0.97
RvsS.0.101 specific
5 genes
RvsS.2.101 specific
4 genes
RvsS.1.101
r = 0.99
RvsS.1.101 specific
3 genes
RvsS.2.101 specific
4 genes
RvsS.2.101
−4
−2
0
2
4
